# Si−H Activation via Dynamic Permutational Isomerism: A Ligand‐Directed Route to Dehydrogenative Coupling

**DOI:** 10.1002/anie.202517017

**Published:** 2025-09-30

**Authors:** Manuel Kümper, Franz F. Westermair, Tobias Götz, Ruth M. Gschwind, Jonathan O. Bauer

**Affiliations:** ^1^ Faculty of Chemistry and Pharmacy Institute of Inorganic Chemistry University of Regensburg Universitätsstraße 31 D‐93053 Regensburg Germany; ^2^ Faculty of Chemistry and Pharmacy Institute of Organic Chemistry University of Regensburg Universitätsstraße 31 D‐93053 Regensburg Germany

**Keywords:** Dehydrogenative coupling, Dynamic isomerism, NMR spectroscopy, Pentacoordinate intermediates, Silicon

## Abstract

Dehydrogenative coupling (DHC) of hydridosilanes with silanols under metal‐free conditions provides a sustainable route to Si─O bond formation. Yet, the mechanistic origin of hydrogen release in such systems has remained unclear. Here, we show that dynamic permutational isomerism of pentacoordinate silicon intermediates is a key prerequisite for Si─H activation and H_2_ release. Using sterically tailored diaminohydridosilanes, we demonstrate that only ligands enabling access to axial hydride configurations facilitate Si─O coupling with productive H_2_ elimination. In contrast, N–*tert*‐butyl substitution locks the hydride in the equatorial position and diverts reactivity toward Si─N bond cleavage. Multinuclear variable‐temperature NMR spectroscopy, combined with quantum chemical calculations, reveals an equilibrium between equatorial and axial hydride configurations, enabling Berry pseudorotation and hydrogen evolution. These findings provide a mechanistic rationale for H_2_ release in hydridosilicates and establish ligand‐directed isomerism as a general design principle for selective, metal‐free Si─H activation.

## Graphical abstract

Ligand‐controlled dynamic permutational isomerism in pentacoordinate silicon intermediates enables metal‐free dehydrogenative Si─O coupling. The accessibility of axial hydride positions dictates whether Si─H bond activation or Si─N bond cleavage occurs, providing a mechanistic framework for understanding hydrogen release and coupling processes in silicon chemistry.

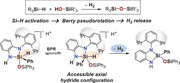

## Introduction

Dehydrogenative coupling (DHC) has emerged as a fundamental strategy in modern synthetic chemistry, offering outstanding atom economy, operational simplicity, and environmental sustainability.^[^ ^1^, ^2^, ^3^, ^4^, ^5^, ^6^, ^7^, ^8^, ^9^, ^10^, ^11^, ^12^, ^13^, ^14^, ^15^, ^16^, ^17^, ^18^ ^]^ Metal‐free variants, in particular, have attracted increasing attention as they offer an alternative approach to the formation of C–X bonds (X = C, N, O, S), aligning with the principles of green and efficient synthesis. ^[^ ^19^, ^20^, ^21^, ^22^, ^23^, ^24^, ^25^, ^26^, ^27^ ^]^ Within the realm of silicon chemistry, dehydrogenative coupling of hydridosilanes with protic nucleophiles such as water, silanols, alcohols, amines, or thiols represents an extremely versatile methodology for the synthesis of valuable functionalized silicon‐based compounds, thus leading to silanols,^[^ ^28^ ^]^ siloxanes,^[^ ^29^ ^]^ silyl ethers,^[^ ^30^, ^31^, ^32^, ^33^, ^34^ ^]^ aminosilanes,^[^ ^35^, ^36^, ^37^ ^]^ and thiosilanes^[^ ^38^, ^39^, ^40^, ^41^, ^42^ ^]^ in a convenient and atom‐economic manner. Among the dehydrogenative coupling strategies, even asymmetric catalytic variants toward silicon‐stereogenic compound libraries have been developed in recent years,^[^ ^43^, ^44^, ^45^, ^46^, ^47^, ^48^, ^49^, ^50^ ^]^ which are of great relevance for materials science^[^ ^51^ ^]^ and pharmaceutical development.^[^ ^52^, ^53^, ^54^, ^55^ ^]^

Despite the synthetic utility and the ever‐expanding substrate scope of these transformations, key mechanistic questions remain unresolved. In this regard, the involvement of permutation processes in pentacoordinate silicon intermediates consistently contributes to a more precise understanding of substitution mechanisms.^[^ ^56^, ^57^, ^58^, ^59^, ^60^, ^61^, ^62^, ^63^, ^65^, ^66^, ^67^ ^]^ In recent years, increasing attention has also been directed toward the structural dynamics of tetrahedral p‐block element hydrides, highlighting the significance of structural fluxionality in substitution processes.^[^ ^68^, ^69^ ^]^

**Figure 1:**
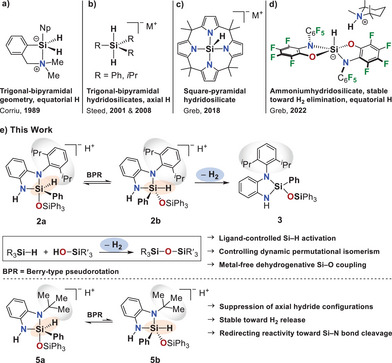
a) Trigonal‐bipyramidal silicon species with equatorial hydride positioning reported by Corriu.^[^ ^76^ ^]^ b) Hydridosilicates with axial hydride positioning as reported by Steed.^[^ ^80^, ^81^ ^]^ c,d) Square‐pyramidal and trigonal‐bipyramidal hydridosilicates reported by Greb;^[^ ^83^, ^87^ ^]^ the ammonium salt (d) features an equatorial hydride and remains inert toward H_2_ release.^[^ ^87^ ^]^ e) This work: Ligand‐controlled Si─H activation via dynamic permutational isomerism enables selective H_2_ release in the Dipp‐substituted system by accessing axial hydride isomers (Dipp = 2,6‐di‐*iso*‐propylphenyl).

Early mechanistic studies suggest that dehydrogenative coupling reactions are initiated by nucleophilic attack on the silicon center of a tetravalent hydridosilane, proceeding via a pentacoordinate, anionic silicon intermediate with trigonal‐bipyramidal geometry.^[^ ^70^, ^71^ ^]^ It is generally assumed that nucleophilic attack on tetravalent silicon species typically proceeds via an axial approach *anti* to an electron withdrawing, apicophilic substituent,^[^ ^72^, ^73^, ^74^ ^]^ with the hydride ligands preferentially adopting equatorial positions due to their low apicophilicity (Figure 1a)^[^ ^75^, ^76^, ^77^, ^78^, ^79^ ^]^ In subsequent years, however, Steed and Okuda reported pentacoordinate species with hydride ligands occupying axial positions in trigonal‐bipyramidal geometries.^[^ ^80^, ^81^, ^82^ ^]^ These findings stood in marked contrast to Corriu's earlier results and reignited the debate concerning the factors that determine the positioning of hydride ligands in such systems. (Figure 1b).

Recent studies by Greb have largely questioned the established picture of reactivity and Lewis acidity in silicon chemistry.^[^ ^83^, ^84^, ^85^, ^86^, ^87^, ^88^ ^]^ The exploration of exceptionally stable pentacoordinate hydridosilicates challenged the general validity of previously proposed substitution pathways and raised new questions concerning the stability of hydridosilicate intermediates and the reversibility of hydrogen activation (Figure 1c,d).^[^ ^83^, ^87^ ^]^ The isolated ammoniumhydridosilicate reported by Greb et al. is particularly noteworthy (Figure 1d).^[^ ^87^ ^]^ Despite the close spatial proximity of protic and hydridic hydrogen atoms, the compound exhibits a surprising inertness toward H_2_ elimination. A closer inspection of the molecular structure reveals that the hydride ligand in fact occupies an equatorial position of a trigonal bipyramid.^[^ ^87^ ^]^ This raises the critical question of how the positioning of hydrogen atoms in hydridosilicate intermediates dictates Si─H bond activation^[^ ^89^ ^]^ in dehydrogenative reaction pathways.

The aim of this work is to investigate for the first time the influence of dynamic permutational isomerism as a key prerequisite for the dehydrogenative coupling ability of hydridosilanes (Figure 1e). We demonstrate that modifying the ligand environment has a profound impact on the reaction outcome and can be exploited to selectively control reactivity. When using the sterically demanding Dipp substituent (Dipp = 2,6‐di‐*iso*‐propylphenyl), dehydrogenative coupling occurs. In contrast, incorporation of a *tert*‐butyl group into the ligand backbone results in an entirely different reaction pathway via ring opening, which preserves the Si─H bond and prevents H_2_ elimination.

Mechanistic investigations, combining single‐crystal X‐ray diffraction analysis, multinuclear variable‐temperature (VT) NMR spectroscopy, and quantum chemical calculations, show the presence of two pentacoordinate intermediates giving rise to separate reaction pathways. Our findings reveal a subtle yet critical interplay between ligand framework and permutational dynamics, offering unprecedented mechanistic insights into Si─H activation. We believe that these results underscore the importance of permutational dynamics in controlling hydrogen release reactions of tetravalent hydridosilanes and pave new avenues for transition‐state engineering in dehydrogenative coupling methodologies and for the rational development of reversible hydrogen storage systems using p‐block elements.

## Results and Discussion

### Divergent Reactivity Trends and Probing Steric Effects on Hydrogen Release

**Scheme 1:**
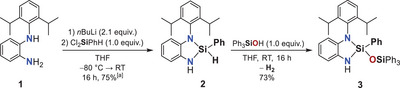
Synthesis of diaminohydridocyclosilane **2** from diaminoligand **1** and subsequent hydrogen‐releasing reaction with triphenylsilanol to form disiloxane **3**. [a] Yield determined by ^1^H NMR spectroscopy using hexamethylbenzene as internal standard.

To investigate the potential of diaminohydridosilanes for dehydrogenative coupling with silanols, we synthesized the five‐membered cyclic compound **2** via lithiation of diaminoligand **1**, followed by reaction with dichlorophenylsilane (Scheme 1).

**Figure 2:**
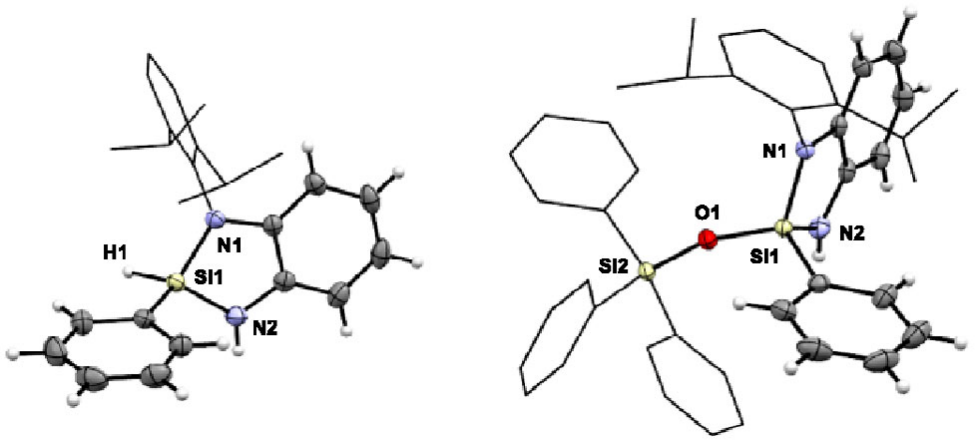
Molecular structures of compounds **2** (left) and **3** (right) in the crystal (displacement ellipsoids set at the 50% probability level). Dipp and Ph_3_SiO groups are shown in wireframe, and corresponding hydrogen atoms are omitted for clarity. Selected bond lengths / Å and angles/° of compound **2** (at 123 K): Si1–N1: 1.7413(4), Si1–N2: 1.7279(5), Si1–H1: 1.546(7), N1–Si1–N2: 91.32(2), N1–Si1–H1: 110.1(3), N2–Si1–H1: 118.8(3). Selected bond lengths / Å and angles/° of compound **3** (at 123 K): Si1–N1: 1.733(1), Si1–N2: 1.724(1), Si1–O1: 1.618(1), N1–Si1–N2: 91.81(6), N1–Si1–O1: 114.13(5), N2–Si1–O1: 115.86(6).^[^ ^90^ ^]^

Compound **2** was obtained in 75% yield and characterized by single‐crystal X‐ray diffraction analysis (Figure 2, left).^[^ ^90^ ^]^ The molecular structure reveals a five‐membered ring comprising two Si─N bonds and one Si─H bond. The Si─N bond lengths [1.7413(4) Å and 1.7279(5) Å] are consistent with related compounds in the literature,^[^ ^91^, ^92^, ^93^ ^]^ whereas the Si─H bond is significantly elongated at 1.546(7) Å compared to the previously reported analogue by Avent et al. [1.382(15) Å].^[^ ^91^ ^]^ The compressed N─Si─N bond angle [91.32(2)°] and the widened N─Si─H angles [118.8(3)° and 110.1(3)°] suggest a weakened Si─H bond, susceptible to cleavage by nucleophilic attack.

The reaction of compound **2** with triphenylsilanol in tetrahydrofuran (THF) proceeds smoothly under gas evolution to afford disiloxane **3** in excellent yield (Scheme 1). Single‐crystals of **3** were obtained from pentane and analyzed by single‐crystal X‐ray diffraction analysis, confirming the preservation of the cyclic backbone (Figure 2, right).^[^ ^90^ ^]^ Structural comparison between compounds **2** and **3** shows only minimal changes in the Si─N bond lengths [1.733(1) Å and 1.724(1) Å in **3**] and the N─Si─N angle [91.81(6)° in **3**]. The newly formed Si─O bond measures 1.618(1) Å, and the N─Si─O angles [114.13(5)° and 115.86(6)°] are in good agreement with stable siloxane architectures.^[^ ^93^ ^]^

**Scheme 2:**
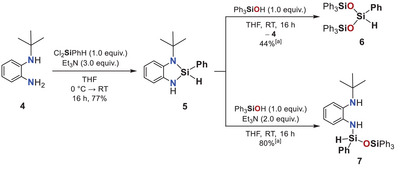
Synthesis of diaminohydridocyclosilane **5** from diaminoligand **4** and subsequent reaction with triphenylsilanol to give oligosiloxane **6** via a double Si─N bond cleavage (top). Under basic conditions, the reaction affords **7** with cleavage of a single Si─N bond (bottom). [a] Yield determined by ^1^H NMR spectroscopy using hexamethylbenzene as internal standard.

**Figure 3:**
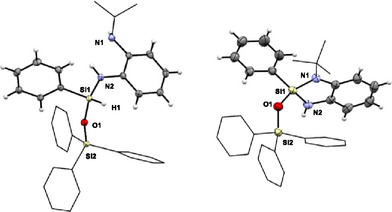
Molecular structures of compounds **7** (left) and **9** (right) in the crystal (displacement ellipsoids set at the 50% probability level). *tert*‐Butyl and Ph_3_SiO groups are shown in wireframe, and corresponding hydrogen atoms are omitted for clarity. Selected bond lengths / Å and angles/° of compound **7** (at 123 K): Si1–N1: 1.709(1), Si1–H1: 1.37(2), N2–Si1–O1: 113.60(5), N2–Si1–H1: 110.2(7). Selected bond lengths / Å and angles/° of compound **9** (at 123 K): Si1–N1: 1.734(2), Si1–N2: 1.719(1), Si1–O1: 1.625(1), N1–Si1–N2: 92.55(7), N1–Si1–O1: 116.67(7), N2–Si1–O1: 113.78(7).^[^ ^90^ ^]^

To assess the influence of steric bulk on hydrogen release, a *tert*‐butyl‐substituted analogue (**5**) was synthesized from ligand **4** (Scheme 2). Upon treatment with triphenylsilanol, trisiloxane **6** was obtained in 44% yield, resulting from the cleavage of two Si─N bonds in compound **5**. Conducting the same reaction in the presence of triethylamine suppressed excessive cleavage, affording the partially converted product **7** in 80% yield, in which one Si─N bond remains intact. Single‐crystal X‐ray structural analysis of **7** shows shorter Si─N [1.709(1) Å] and Si─H bonds [1.37(2) Å] compared to the cyclic Dipp‐patterned compound **2**, suggesting a strengthened Si─H bond and hence decreased reactivity toward hydrogen release (Figure 3, left).^[^ ^90^ ^]^

**Scheme 3:**
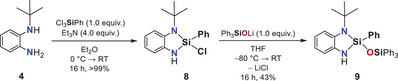
Synthesis of disiloxane **9** from diaminoligand **4**, involving cyclization with trichlorophenylsilane followed by reaction with lithium triphenylsilanolate.

An alternative route to disiloxane **9**, the *tert*‐butyl analogue of compound **3**, was achieved by conversion of the chloro derivative **8**, prepared in >99% yield from diamine **4** and PhSiCl_3_, with lithium triphenylsilanolate (Scheme 3). Single‐crystal X‐ray diffraction analysis of **9** shows Si─N and N─Si─O bond parameters similar to those in compound **3**, indicating that ring strain and geometry of the tetracoordinate species alone are insufficient to explain the lack of hydrogen release in the *tert*‐butyl‐substituted system (Figure 3, right).^[^ ^90^ ^]^ This prompted us to conduct a mechanistic investigation combining variable‐temperature ^1^H and ^29^Si NMR spectroscopy and quantum chemical calculations.

### NMR Studies of Pentacoordinate Silicon Intermediates

**Figure 4:**
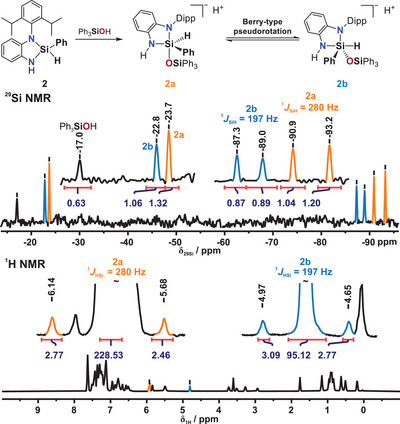
^29^Si NMR (top) and ^1^H NMR (bottom) spectra of the reaction mixture of **2** and Ph_3_SiOH in THF‐*d*_8_ at 180 K, showing new signals corresponding to intermediates **2a** (orange) and **2b** (blue).

^1^H and ^29^Si NMR experiments were conducted in the presence of Ph_3_SiOH over a temperature range of 180 K to 298 K (see also Section S3). At 180 K (Figure 4), two species (**2a** and **2b**) were observed in a close to 1:1 ratio, each exhibiting characteristic ^29^Si NMR chemical shifts and ^1^ *J*_Si–H_ coupling constants (280 Hz for **2a**; 197 Hz for **2b**). The differences suggest distinct coordination geometries, attributed to a pentacoordinate silicon center through coordination of a silanolate anion, with the hydride in either equatorial (**2a**) or axial (**2b**) position.

These findings agree with the chemical shift ranges previously reported for pentacoordinate hydridosilicate species,^[^ ^94^ ^]^ thereby supporting the structural assignments of **2a** and **2b**. Notably, the deshielding of the ^29^Si NMR signal relative to the parent compound **2** is stronger in **2b** than in **2a**. A pronounced difference in the ^1^ *J*_Si–H_ coupling constants (280 Hz for **2a** vs. 197 Hz for **2b**) further substantiates this distinction, suggesting greater s‐orbital character in the Si─H bond of **2a**.^[^ ^95^ ^]^ In contrast, the diminished ^1^ *J*_Si–H_ coupling constant for **2b** implies a reduced s‐orbital contribution compared to the starting material **2**. These observations are consistent with a model in which the Si─H bond occupies an equatorial position in **2a** and an axial position in **2b**, assuming a trigonal bipyramidal coordination geometry.

**Figure 5:**
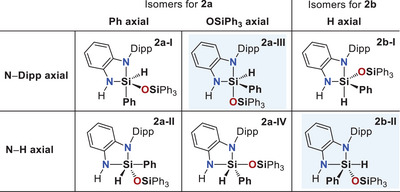
Structures of plausible anionic pentacoordinate silicon intermediates (charges are omitted for clarity). Based on the findings, the designated compound **2a** corresponds to structure **2a‐III** and compound **2b** to structure **2b‐II**. The coordination geometries of structures **2a** and **2b** are best described as slightly distorted yet clearly trigonal‐bipyramidal, with the exception of **2a‐IV**, which more closely adopts a basally distorted square‐pyramidal arrangement featuring the phenyl substituent at the apex (for details, see Section S6).^[^ ^96^ ^]^

In bidentate coordination scenarios where a five‐membered chelate ring coordinates the silicon center, steric effects typically lead to one donor atom being placed in an equatorial position and the other in an axial position (Figure 5). Given the comparably low electron‐withdrawing property of the phenyl substituent, it is plausible that the axial sites in the four conceivable pentacoordinate isomers of **2a** (hydride in equatorial position) are preferentially occupied by the more electronegative silanolate oxygen atom, as represented in structures **2a‐III** and **2a‐IV**. In contrast, arrangements **2a‐I** and **2a‐II**, in which the phenyl group occupies the axial position, are expected to be less stable. Additionally, in structure **2a‐III**, steric repulsion between the bulky N–Dipp and OSiPh_3_ substituents is minimized by maximizing their spatial separation. For the two conceivable isomers of **2b** (hydride in axial position), structure **2b‐II** is most plausible, as it minimizes steric repulsion by placing both the N–Dipp and OSiPh_3_ substituents in equatorial positions.

### Computational Analysis of the Permutational Isomerism of Pentacoordinate Silicon Intermediates

Quantum chemical calculations were performed to gain deeper insight into the geometric preferences and electronic structures of the proposed intermediates, thereby refining our mechanistic picture (see Section S4). Geometry optimizations and harmonic frequency analyses were carried out using the composite method PBEh‐3c^[^ ^97^ ^]^ (with D3BJ dispersion correction)^[^ ^98^, ^99^ ^]^ and gCP (geometrical counter‐poise correction).^[^ ^100^ ^]^ Solvation by THF was considered by applying the conductor‐like polarizable continuum model (CPCM).^[^ ^101^, ^102^ ^]^ Nuclear magnetic shielding tensors and scalar coupling constants were computed using the PBE0 functional^[^ ^103^ ^]^ in conjunction with the pcSseg‐2^[^ ^104^ ^]^ and pc*J*‐2^[^ ^105^ ^]^ basis sets, and with CPCM(THF) solvation. Benchmark calculations on [F_3_Si(CH_2_OAc)] (**10**), on the key starting materials (**2**, **5**, and Ph_3_SiOH), and on product **3** showed excellent agreement between computed and experimental data (see Section S4.2).

Relative Gibbs energies (Δ*G*) were evaluated for the six plausible anionic pentacoordinate silicon species derived from the corresponding ligand positioning (Figure 5). It was found that optimization of structure **2a‐I** consistently converged to **2b‐II**, thereby clearly excluding **2a‐I** as a distinct local minimum. The lowest‐energy structures were in fact identified as **2a‐III** (Δ*G*_calc_ = +1.2 kJmol^−1^), featuring axial N–Dipp and siloxy substituents, and **2b‐II** (Δ*G*_calc_ = 0.0 kJ mol^−1^), in which the NH group and the hydride substituent occupy the axial positions. All remaining isomers were calculated to be ≥ 12.0 kJ mol^−1^ higher in energy. Given the expected accuracy of these methods (errors typically > ±5 kJ mol^−1^), the results are in excellent agreement with the NMR spectroscopically observed equilibrium with near‐equal populations of **2a** and **2b**.

Furthermore, the calculated NMR parameters closely match the experimental data [calculated: *δ* (^29^Si) = −98.7 ppm and ^1^ *J*_Si–‍H_ = −245 Hz for **2a‐III**; *δ* (^29^Si) = −100.0 ppm and ^1^ *J*_Si–H_ = −187 Hz for **2b‐II**]. These results strongly support the proposed structural assignments. The calculated ^1^H NMR chemical shifts also align well with the proposed geometries.

The exact position of the proton (countercation) cannot be determined unambiguously owing to the dynamic solution environment. Moreover, proton complexation by ethereal solvents such as THF must be considered, a phenomenon that is well documented in the literature.^[^ ^106^ ^]^ This issue has recently been examined in detail for related systems.^[^ ^107^ ^]^

To evaluate possible neutral (zwitterionic) species, DFT calculations were performed for N‐protonated derivatives of **2a** and **2b**, arising from silanolate addition to silicon followed by protonation at nitrogen (see Section S4.3). Protonation at the N–Dipp substituent (**2c‐I–VI**) afforded structures that deviate strongly from pentacoordinate geometries, instead resembling ring‐opened species with a cleaved Si─N(Dipp) bond and tetrahedral silicon coordination.^[^ ^93^ ^]^ The corresponding calculated ^29^Si NMR chemical shifts (−27 to −44 ppm) are inconsistent with the experimental values, which clearly indicate pentacoordinate species. Such structures can therefore be excluded. In contrast, protonation at the NH group (**2d‐I**–**III**) resulted in elongated Si─NH_2_ bonds while largely preserving trigonal‐bipyramidal coordination (see Section S4.3). Among these structures, **2d‐II**, the NH protonated analogue of **2b‐II**, emerged as the only neutral pentacoordinate species, which is thermodynamically feasible and in reasonable agreement with the experimental NMR data of **2b**. It is conceivable that such a neutral, zwitterionic species plays a role in the isomerization process. Experiments with deuterated silanol (Ph_3_SiOD) indicate rapid NH/OD exchange in solution (see Section S3.6), suggesting that the proton can indeed reside on the amine function.

In addition, the same calculations were performed on the six analogous N–*tert*‐butyl‐substituted anionic pentacoordinate isomers, formed by nucleophilic attack of the silanolate anion on compound **5**, using the same computational protocol and nomenclature (see Section S4.3). The lowest‐energy structure (Δ*G*_calc_ = 0.0 kJ mol^−1^) features axially positioned N–*tert*‐butyl and siloxy groups and reflects the energetically favorable arrangement also found for the N–Dipp analogue **2a‐III**. In contrast, the *tert*‐butyl structure **5b‐II** corresponding to **2b‐II** with the hydride substituent in the axial position lies significantly higher in energy (Δ*G*_calc_ = +17.9 kJ mol^−1^). Other isomers, with axially located N–*tert*‐butyl and either the phenyl (Δ*G*_calc_ = +15.8 kJ mol^−1^) or hydride substituent (Δ*G*_calc_ = +26.4 kJ mol^−1^) in the other axial position, were also energetically disfavored, with the remaining conceivable structures even less stable. These findings suggest that species with an axially located hydride substituent, which is a prerequisite for hydrogen evolution, are not significantly populated for the *tert*‐butyl case, explaining the lack of hydrogen release for compound **5**. Instead, Si─N bond cleavage products (**6** and **7**) dominate, which is in line with the experimental results.

### Mechanistic Insights Into Pseudorotation and Hydrogen Release

**Figure 6:**
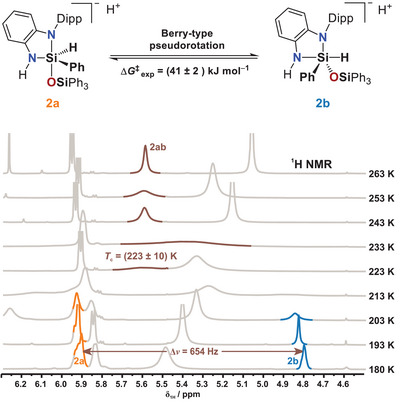
Variable‐temperature ^1^H NMR spectra (THF‐*d*_8_, 180–263 K) of the reaction of **2** with Ph_3_SiOH. Signals are assigned to **2a**, **2b**, and the averaged intermediate **2ab** based on ^29^Si satellites. The coalescence temperature of *T*_c_ = (223 ± 10) K and the Larmor frequency difference of Δ*ν* = 654 Hz result in an activation barrier for the Berry pseudorotation of Δ*G* ^‡^_BPR_ = (41 ± 2) kJ mol^−1^. The observed rate constant at the coalescence temperature was determined to be *k*_obs_ = 1450 s^−1^ (for details, see Section S3.3).

We carried out a series of further NMR experiments to gain additional insights into the reaction mechanism. Based on all these results, we propose that species **2a** represents the initially formed intermediate, formed upon nucleophilic attack of the silanolate anion, which occurs spatially opposite the sterically demanding N–Dipp group (Figure 6). The equatorially positioned hydride then undergoes Berry pseudorotation, giving rise to species **2b**, which subsequently releases hydrogen. To investigate this dynamic behavior, variable‐temperature ^1^H NMR experiments were performed. These revealed the presence of an averaged signal for **2a** and **2b**, designated as **2ab**, above 233 K (Figure 6). The observed coalescence temperature of *T*_c_ = (223 ± 10) K and the Larmor frequency difference of Δ*ν* = 654 Hz at low temperature resulted in an activation free energy for the Berry pseudorotation of Δ*G* ^‡^_BPR_ = (41 ± 2) kJ mol^−1^. At the coalescence temperature, the observed rate constant is *k*_obs_ = 1450 s^−1^.

**Figure 7:**
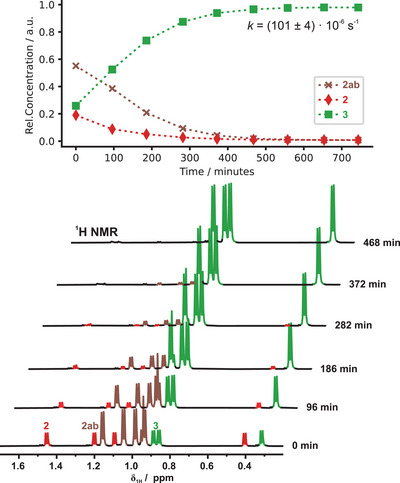
Aliphatic region of the ^1^H NMR spectra monitoring the reaction of **2** with Ph_3_SiOH in THF‐*d*_8_ at 298 K. Consumption of both **2** and intermediates **2ab** together with formation of product **3** is observed. A first‐order rate constant of *k* = (101 ± 4) × 10^−6^ s^−1^ was determined. *t* = 0 min marks the start of NMR monitoring; the sample was hand‐warmed and shaken for 5 min beforehand.

Further reaction monitoring at 298 K revealed transient ^1^H and ^29^Si NMR signals corresponding to a mixture of the presumed pentacoordinate species. From these data, a pseudo‐first‐order rate constant of *k* = (101 ± 4) × 10^−6^ s^−1^ was determined (Figure 7). Application of the Eyring–Polanyi equation afforded an activation free energy of Δ*G* ^‡^ = (95.8 ± 0.1) kJ mol^−1^ for the H_2_ elimination step (see Section S3.4). In agreement with the mechanistic model proposed by Eaborn and Jenkins,^[^ ^71^ ^]^ we conclude that hydrogen evolution constitutes the rate‐determining step, following a pre‐equilibrium between **2** and pentacoordinate intermediates **2a** and **2b**, with pseudorotation facilitating hydride transfer and subsequent H_2_ elimination. The close energetic proximity of **2a** and **2b** is also consistent with our quantum chemical calculations (see discussion above).

Additional support for this mechanistic scenario was obtained using Ph_3_SiOK as nucleophile, which was employed to probe the involvement of anionic intermediates (for details, see Section 3.5). At low temperatures (233 K), species **2a** predominates, with only trace amounts of **2b** detectable. Upon heating to 298 K, the equilibrium shifts toward an increased population of **2b**. The NMR parameters observed under basic conditions are nearly identical to those of **2a** and **2b** under neutral conditions, supporting their description as anionic pentacoordinate hydridosilicates formed by coordination of a silanolate anion to compound **2**. These observations further indicate that **2b** is thermodynamically slightly favored, while **2a** is likely the initially formed, kinetic species.

Low temperature NMR studies on the *tert*‐butyl‐substituted compound **5** in the presence of excess Ph_3_SiOH and triethylamine revealed no detectable intermediates, instead, full conversion to **7** was observed (see Section S3.7).

Overall, our combined spectroscopic and computational analysis demonstrates that electronic and steric parameters of the ligand critically govern hydrogen release in these systems. Moreover, these findings underscore the pivotal role of the N–Dipp group in stabilizing reactive pentacoordinate intermediates, uniquely enabling hydrogen release while preserving the integrity of the five‐membered ring.

## Conclusion

In summary, we have demonstrated that dynamic permutational isomerism of pentacoordinate silicon intermediates is a decisive factor for dehydrogenative Si─O coupling, enabling hydrogen evolution from diaminohydridosilanes under mild, metal‐free conditions. Through systematic ligand modification, NMR spectroscopy, and quantum chemical calculations, we revealed that the sterically demanding N–Dipp substituent facilitates a delicate equilibrium between equatorial and axial hydride positions, which is essential for hydrogen release via Berry‐type pseudorotation. In contrast, N–*tert*‐butyl substitution suppresses axial hydride configurations, thereby hindering H_2_ evolution and redirecting reactivity toward Si─N bond cleavage. These findings establish dynamic ligand–substrate interplay as a tunable approach for controlling reactivity in Si─H activation and dehydrogenative coupling, and they provide a general mechanistic framework for understanding hydrogen release in silicon chemistry: The accessibility of axial hydride configurations in trigonal‐bipyramidal intermediates emerges as a key prerequisite for productive H_2_ elimination. Our findings thus provide a clear rationale for the absence of hydrogen release in systems lacking accessible axial hydride isomers and dynamically viable permutation pathways, also offering a compelling explanation for the observed inertness of Greb's ammoniumhydridosilicate toward H_2_ evolution.^[^ ^87^ ^]^

Overall, our work significantly advances the fundamental understanding of Si─H bond activation and paves the way for the rational design of selective, metal‐free hydrogen transfer and coupling processes in main‐group chemistry. We further believe that these findings represent a significant step toward the development of reversible hydrogen storage systems based on p‐block elements. Future work will utilize these mechanistic principles for the development of catalytic systems exploiting dynamic control over reactive pentacoordinate intermediates in silicon chemistry.

## Supporting Information

The authors have cited additional references within the Supporting Information.^[^ ^108^, ^109^, ^110^, ^111^, ^112^, ^113^, ^114^, ^115^, ^116^, ^117^, ^118^, ^119^, ^120^, ^121^, ^122^, ^123^, ^124^, ^125^, ^126^, ^127^, ^128^, ^129^, ^130^, ^131^, ^132^, ^133^, ^134^, ^135^, ^136^, ^137^, ^138^, ^139^, ^140^, ^141^ ^]^

## Author Contributions

Synthesis, single‐crystal X‐ray diffraction analysis, and characterization of compounds were performed by M.K. and T.G. F.F.W. carried out VT‐NMR measurements, reaction monitoring, and quantum chemical calculations under the supervision of R.M.G. F.F.W., R.M.G., and J.O.B. analyzed and interpreted NMR/DFT data to gain mechanistic insights. J.O.B. supervised and coordinated the project and interpreted all the results. The manuscript was prepared by M.K. and J.O.B., with input from all other authors. The Supporting Information was prepared in collaboration with all authors.

## Conflict of Interests

The authors declare no conflict of interest.

## Supporting information



Supporting Information

## Data Availability

The data that support the findings of this study are available in the Supporting Information of this article.
