# Supplementary material for: Si−H Activation via Dynamic Permutational Isomerism: A Ligand‐Directed Route to Dehydrogenative Coupling
Source: Angew Chem Int Ed Engl. 2025 Sep 30;64(47):e202517017. doi: 10.1002/anie.202517017 (PMC12624319; doi:10.1002/anie.202517017)
Supplement: Supplementary file 1 — Supporting Information [file ANIE-64-e202517017-s001.pdf]

Supporting Information

Si−H Activation via Dynamic Permutational Isomerism: A Ligand-Directed Route to Dehydrogenative Coupling

Manuel Kümper,+[a] Franz F. Westermair,+[b] Tobias Götz,[a] Ruth M. Gschwind,[b] and Jonathan O. Bauer*[a]

[a] Faculty of Chemistry and Pharmacy, Institute of Inorganic Chemistry, University of Regensburg, Universitätsstraße 31, D-93053 Regensburg, Germany

[b] Faculty of Chemistry and Pharmacy, Institute of Organic Chemistry, University of Regensburg, Universitätsstraße 31, D-93053 Regensburg, Germany

+ These authors contributed equally to this work.

Corresponding author: jonathan.bauer@ur.de

Table of contents

1. General Remarks 2
2. Synthetic Procedures 3
2.1. Synthesis of Compound **2** 3
2.2. Synthesis of Compound **3** 7
2.3. Synthesis of Compound **5** 10
2.4. Synthesis of Compound **6** 15
2.5. Synthesis of Compound **7** 19
2.6. Synthesis of Compound **8** 23
2.7. Synthesis of Compound **9** 26
2.8 Synthesis of Triphenylsilanol-*d* 29
2.9 Reaction of Compound **2** with Triphenylsilanol-*d* 32
3. Mechanistic Investigations via NMR Spectroscopy 34
3.1. General Considerations 34
3.2. NMR-Measurement at 180 K 34
3.3. VT-Measurements from 180 K to 263 K: Berry Pseudorotation vs. Dissociative-Associative Mechanism 37
3.4. Reaction monitoring at 298 K 39
3.5. Measurements at 233 K and 298 K Under Highly Basic Conditions 42
3.6. Measurements of **2** with Triphenylsilanol-*d* 47
3.7. Measurements of **5** with Ph_3SiOH and Et_3N 50
4. DFT Calculations 51
4.1. General Considerations 51
4.2. Evaluation of the Method of Choice 51
4.3. Calculations of Compounds in the Systems Under Investigation 53
5. Single-Crystal X-Ray Diffraction Analysis 57
6. Cartesian Coordinates of Optimized Structures 66

1. General Remarks

All experiments were performed in an inert atmosphere of purified nitrogen by using standard Schlenk techniques or an MBraun Unilab 1200/780 glovebox. Glassware was heated at 140 °C prior to use. Dichloromethane (DCM), diethyl ether (Et_2O), *n*-hexane, tetrahydrofuran (THF) and *n*-pentane were dried and degassed with an MBraun SP800 solvent purification system. n-Butyllithium (2.5 M solution in hexane, Merck KGaA), dichlorophenylsilane (96%, abcr GmbH), trichlorophenylsilane (97%, Alfa Aesar), chlorotriphenylsilane (96%, Merck KGaA), deuterium oxide (99.9%, Merck KGaA), hexamethylbenzene (HMB; 99%, Merck KGaA) and triphenylsilanol (98%, Merck KGaA) were used as received without further purification. Triethylamine (≥ 99%, Merck KGaA) was heated at reflux over CaH_2 and distilled prior to use. Compounds **1** and **4** were synthesized according to a previously published procedure.[93,108–110] C_6D_6 (≥ 99.6%, Merck) and THF-d_8 (99.5%, Eurisotop) were degassed and dried over molecular sieve (3 Å) prior to use. NMR spectra were either recorded using a Bruker Avance 400 (400.13 MHz) or a Bruker Avance III HD 400 (400.13 MHz) at 298 K. Chemical shifts (δ) are reported in parts per million (ppm). ^1H and $^{13}C\{^1H\}$ NMR spectra are referenced to tetramethylsilane ($SiMe_4$, δ = 0.0 ppm) as external standard, with the deuterium signal of the solvent serving as internal lock and the residual solvent signal as an additional reference. For the assignment of the multiplicities, the following abbreviations are used: s = singlet, d = doublet, t = triplet, sept = septet, m = multiplet. For simplicity, multiplets of order higher than one are described by approximating them to the closest first-order type. Elemental analyses were performed on a Vario MICRO cube apparatus. High-resolution mass spectrometry was carried out on a Jeol AccuTOF GCX and an Agilent Q-TOF 6540 UHD spectrometer.

2. Synthetic Procedures

2.1. Synthesis of Compound 2

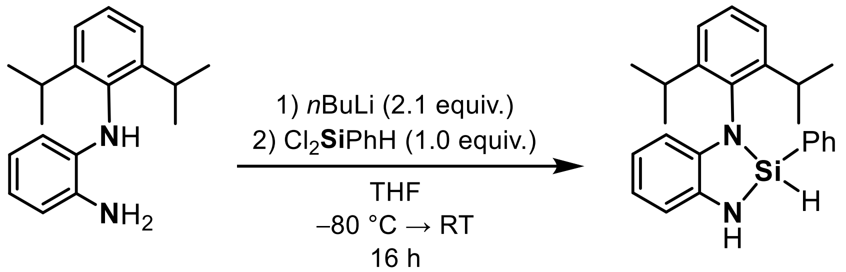

1 2

Compound **1** was prepared according to previously published literature procedures.[93,108,109] Crystals suitable for single-crystal X-ray diffraction analysis were obtained after recrystallization in diethyl ether at room temperature.

Compound **1** (2.68 g, 10.0 mmol, 1.0 equiv.) was dissolved in THF (50 mL) and cooled to –80 °C. *n*-Butyllithium (8.0 mL of a 2.5 M solution in hexane, 20.0 mmol, 2.0 equiv.) was added dropwise. The solution was stirred for 10 minutes while warming up to room temperature. The resulting yellow suspension was cooled to –80 °C again. Dichlorophenylsilane (1.46 mL, 10.0 mmol, 1.0 equiv.) was added via syringe and the reaction mixture was allowed to warm up to room temperature in 16 hours. Then, all volatiles were removed *in vacuo*. The residue was extracted with *n*-pentane (3 × 5 mL) and the remaining solids filtered off via filter cannula. Colorless crystals suitable for single-crystal X-ray diffraction analysis were obtained after storing the combined filtrates at –20 °C overnight (Isolated crystalline Yield: 535 mg, 1.4 mmol, 14%).

In an analogous experiment, hexamethylbenzene (0.5 equiv.) was added as an internal standard before purification and an aliquot was taken and analyzed via ^{1}H NMR spectroscopy, revealing an NMR-based yield of the reaction of 75%.

^{1}H NMR (400 MHz, C_6D_6, 298 K): δ = 0.37 [d, 3H, $^{3}J_{H\text{-}H}$ = 6.7 Hz, CH(C*H*$_3$)$_2$], 1.03 [d, 6H, $^{3}J_{H\text{-}H}$ = 6.8 Hz, CH(C*H*$_3$)$_2$], 1.31 [d, 3H, $^{3}J_{H\text{-}H}$ = 6.8 Hz, CH(C*H*$_3$)$_2$], 3.02 [sept, 1H, $^{3}J_{H\text{-}H}$ = 6.8 Hz, C*H*(CH$_3$)$_2$], 3.16 [s, 1H, N*H*], 3.32 [sept, 1H, $^{3}J_{H\text{-}H}$ = 6.8 Hz, C*H*(CH$_3$)$_2$], 6.14 [s, 1H, Si*H*], 6.13–6.17 [m, 1H, *H*$_{Ar}$], 6.59–6.62 [m, 1H, *H*$_{Ar}$], 6.63–6.69 [m, 1H, *H*$_{Ar}$], 6.76–6.82 [m, 1H, *H*$_{Ar}$], 7.00–7.09 [m, 3H, *H*$_{Ar}$], 7.12–7.18 [m, 2H, *H*$_{Ar}$], 7.36–7.41 [m, 2H, *H*$_{Ar}$]. **^{13}C{^{1}H} NMR** (101 MHz, C_6D_6, 298 K): δ = 23.6 [s, CH*C*H$_3$], 24.8 [s, CH*C*H$_3$], 25.0 [s, CH*C*H$_3$], 25.5 [s, CH*C*H$_3$], 28.2 [s, *C*HCH$_3$], 28.5 [s, *C*HCH$_3$], 110.3 [s, *C*H$_{Ar}$], 111.3 [s, *C*H$_{Ar}$], 118.5 [s, *C*H$_{Ar}$], 118.7 [s, *C*H$_{Ar}$], 124.6 [s, *C*H$_{Ar}$], 124.9 [s, *C*H$_{Ar}$], 128.1 [s, *C*H$_{Ar}$], 128.3 [s, *C*H$_{Ar}$], 131.6 [s, *C*H$_{Ar}$], 134.1 [s, *C*$_{Ar}$], 134.4 [s, *C*$_{Ar}$], 135.1 [s, *C*H$_{Ar}$], 138.3 [s, *C*$_{Ar}$], 141.3 [s, *C*$_{Ar}$], 148.6 [s, *C*$_{Ar}$], 149.3 [s, *C*$_{Ar}$]. **^{29}Si{^{1}H} NMR** (79 MHz, C_6D_6, 298 K): δ = −13.8 [s, *Si*]. **^{29}Si NMR** (79 MHz, C_6D_6, 298 K): δ = −13.8 [dm, $^{1}J_{Si\text{-}H}$ = 233.2 Hz, *Si*]. **CHN Analysis** $C_{24}H_{28}N_2Si$: calculated: C 77.37, H 7.58, N 7.52, Si 7.54; found: C 77.38, H 7.17, N 7.30. **HR-MS(FD+)**, calculated m/z for $C_{24}H_{28}N_2Si$ [M+H^{+}]: 372.20239; found: 372.20163.

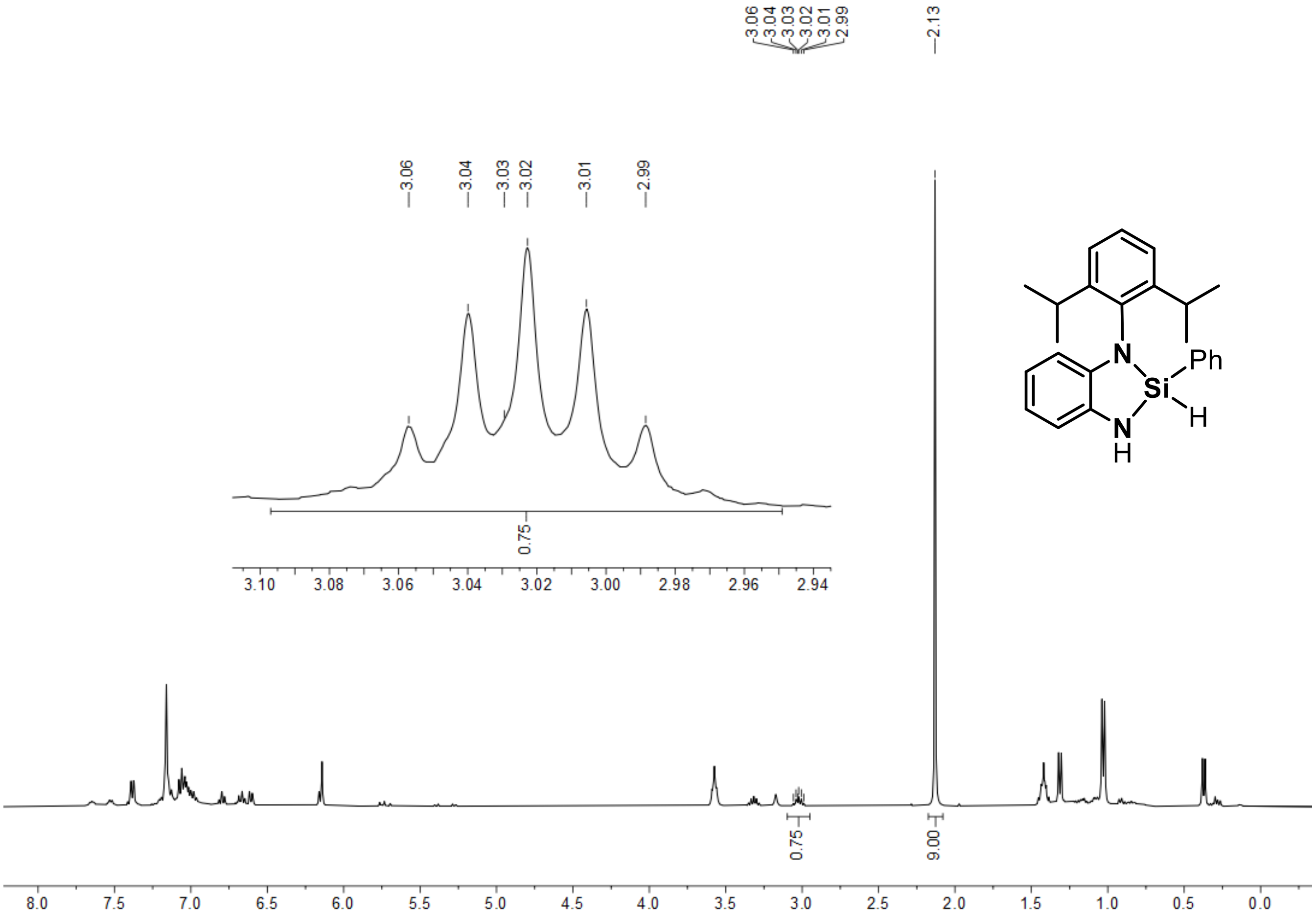

Figure S1. ^{1}H NMR spectrum (C_6D_6, 298 K) of the crude reaction mixture of compound **2** with HMB as Internal Standard.

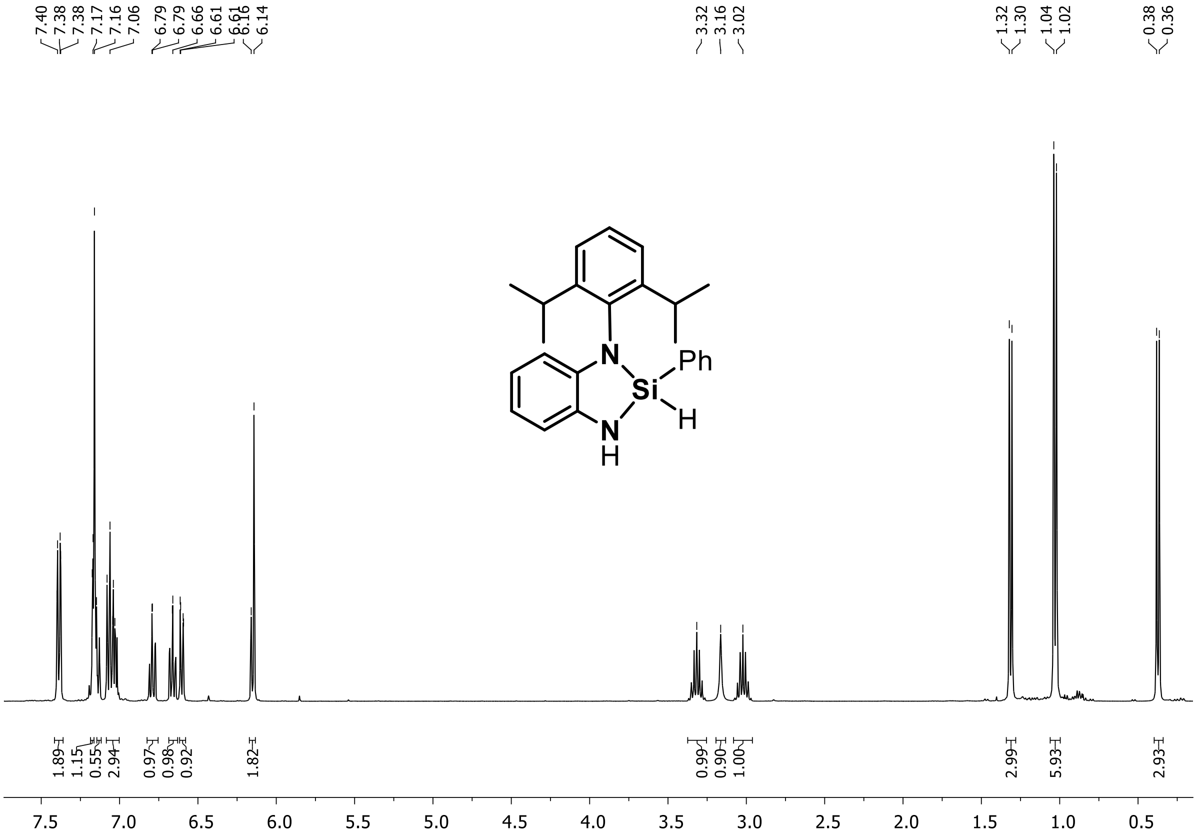

Figure S2. ^{1}H NMR spectrum (C_6D_6, 298 K) of **2**.

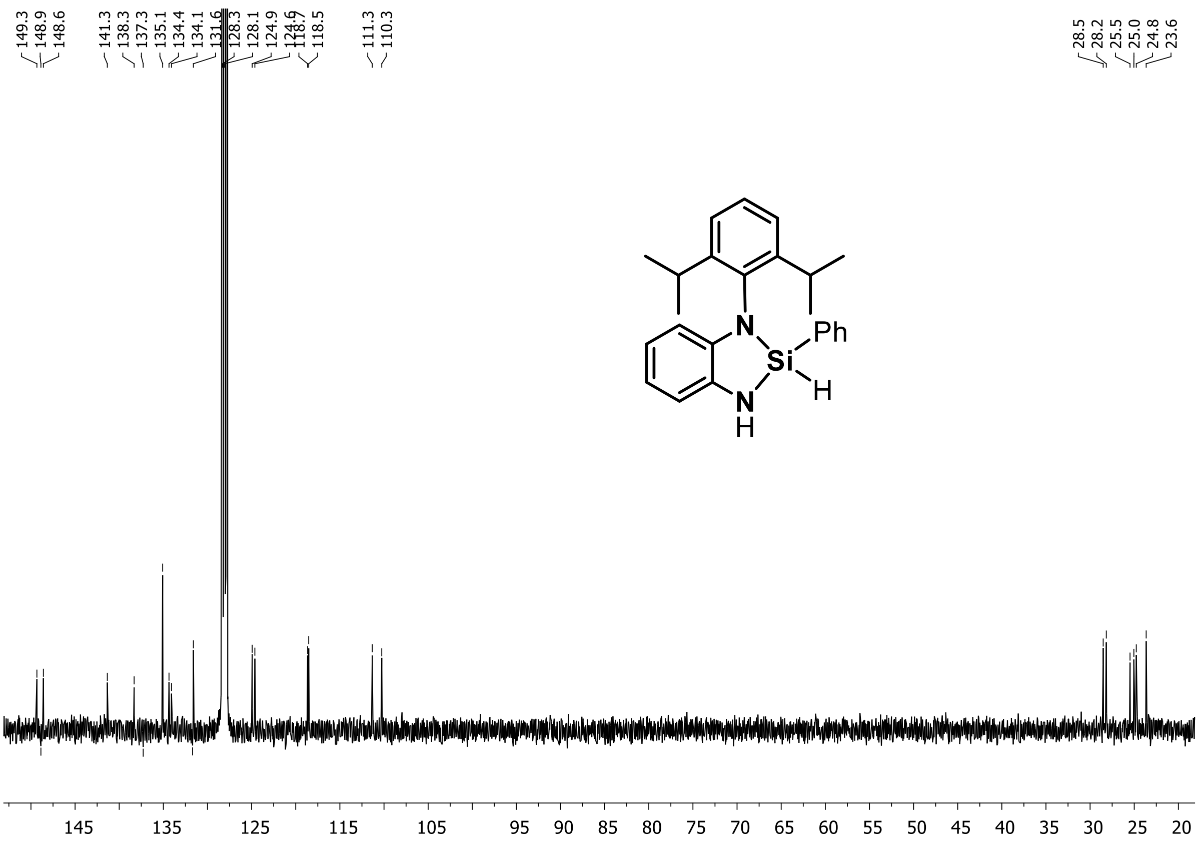

Figure S3. $^{13}C\{^1H\}$ NMR spectrum (C_6D_6, 298 K) of **2**.

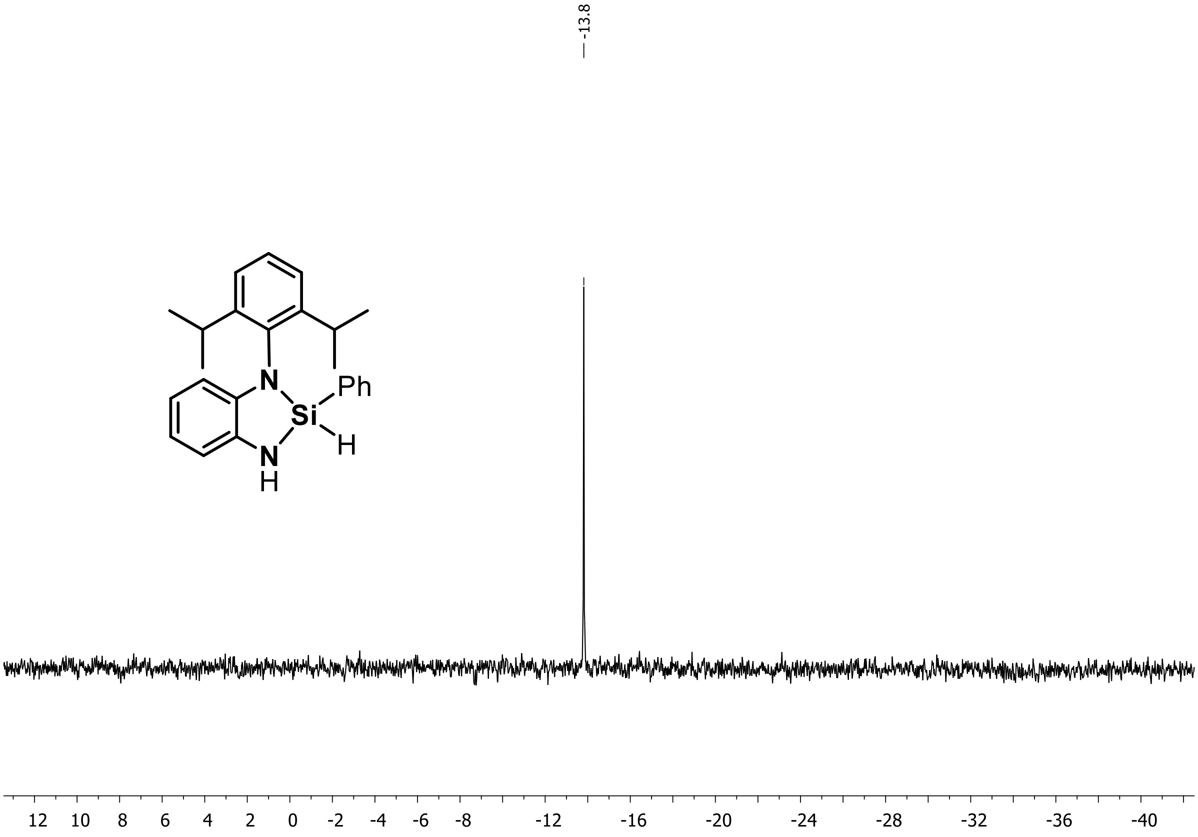

Figure S4. $^{29}Si\{^1H\}$ NMR spectrum (C_6D_6, 298 K) of **2**.

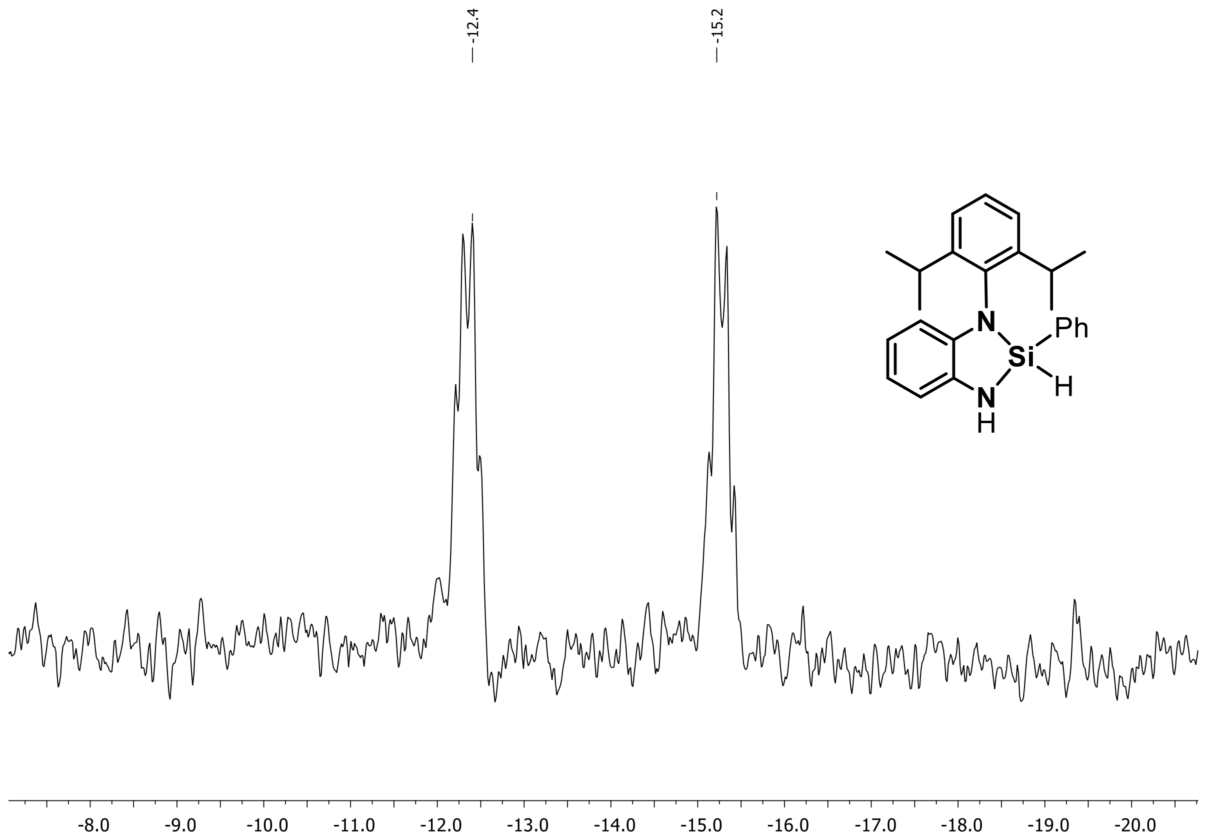

Figure S5. ^{29}Si NMR spectrum (C_6D_6, 298 K) of **2**.

2.2. Synthesis of Compound 3

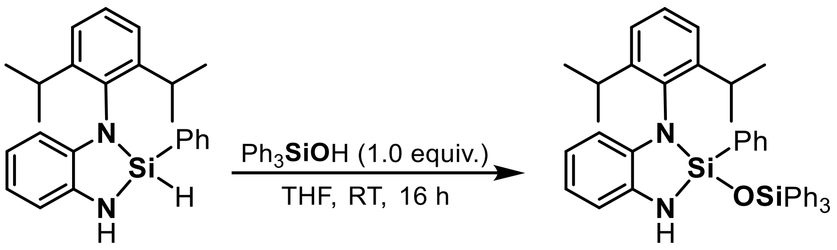

2 **3**

Compound **2** (373 mg, 1.0 mmol, 1.0 equiv.) and triphenylsilanol (276 mg, 1.0 mmol, 1.0 equiv.) were dissolved in THF (20 mL) and stirred at room temperature for 16 hours. Next, all volatiles were removed *in vacuo*. The residue was washed with *n*-pentane to afford compound **3** as a white solid (471 mg, 0.73 mmol, 73%). Crystals suitable for single-crystal X-ray diffraction analysis were obtained by recrystallization from *n*-pentane.

^{1}H NMR (400 MHz, C_6D_6, 298 K): δ = 0.32 [d, 3H, $^3J_{H\text{-}H}$ = 6.8 Hz, CH(C*H*)$_3$], 0.91 [d, 3H, $^3J_{H\text{-}H}$ = 6.8 Hz, CH(C*H*)$_3$], 0.93 [d, 3H, $^3J_{H\text{-}H}$ = 6.8 Hz, CH(C*H*)$_3$], 0.99 [d, 3H, $^3J_{H\text{-}H}$ = 6.8 Hz, CH(C*H*)$_3$], 2.83 [sept, 1H, $^3J_{H\text{-}H}$ = 6.8 Hz, C*H*(CH)$_3$], 3.62 [sept, 1H, $^3J_{H\text{-}H}$ = 6.8 Hz, C*H*(CH)$_3$], 3.84 [s, 1H, N*H*], 6.15 [d, 1H, $^3J_{H\text{-}H}$ = 7.6 Hz, C*H*$_{Ar}$], 6.47 [dd, 1H, $^3J_{H\text{-}H}$ = 7.5 Hz, $^4J_{H\text{-}H}$ = 1.1 Hz, C*H*$_{Ar}$], 6.68 [td, 1H $^3J_{H\text{-}H}$ = 7.6 Hz, $^4J_{H\text{-}H}$ = 1.3 Hz, C*H*$_{Ar}$], 6.80 [td, 1H $^3J_{H\text{-}H}$ = 7.5 Hz, $^4J_{H\text{-}H}$ = 1.2 Hz, C*H*$_{Ar}$], 7.00–7.13 [m, 13H, C*H*$_{Ar}$], 7.17–7.21 [m, 2H, C*H*$_{Ar}$], 7.61–7.65 [m, 6H, C*H*$_{Ar}$], 7.69–7.72 [m, 2H, C*H*$_{Ar}$]. **^{13}C{^{1}H} NMR** (101 MHz, C_6D_6, 298 K): δ = 23.4 [s, CH(*C*H$_3$)$_2$], 24.7 [s, CH(*C*H$_3$)$_2$], 25.5 [s, CH(*C*H$_3$)$_2$], 25.7 [s, CH(*C*H$_3$)$_2$], 28.3 [s, *C*H(CH$_3$)$_2$], 28.4 [s, *C*H(CH$_3$)$_2$], 111.0 [s, *C*H$_{Ar}$], 111.2 [s, *C*H$_{Ar}$], 118.7 [s, *C*H$_{Ar}$], 118.8 [s, *C*H$_{Ar}$], 124.5 [s, *C*H$_{Ar}$], 124.9 [s, *C*H$_{Ar}$], 127.9 [s, *C*H$_{Ar}$], 130.4 [s, *C*H$_{Ar}$], 130.9 [s, *C*H$_{Ar}$], 132.7 [s, *C*$_{Ar}$],, 134.4 [s, *C*$_{Ar}$], 135.5 [s, *C*$_{Ar}$], 135.6 [s, *C*H$_{Ar}$], 135.9 [s, *C*H$_{Ar}$], 136.3 [s, *C*$_{Ar}$], 140.1 [s, *C*$_{Ar}$], 148.7 [s, *C*$_{Ar}$], 149.5 [s, *C*$_{Ar}$]. **^{29}Si{^{1}H} NMR** (79 MHz, C_6D_6, 298 K): δ = −38.7 [s, *Si*N], −16.6 [s, *Si*Ph$_3$]. **CHN Analysis** $C_{42}H_{42}N_2OSi$: calculated: C 77.97, H 6.54, N 4.33, O 2.47, Si 8.68; found: C 78.25, H 6.65, N 4.19. **HR-MS(FD+)**, calculated m/z for $C_{42}H_{42}N_2OSi$ [M+H$^+$]: 646.28302; found: 646.28402.

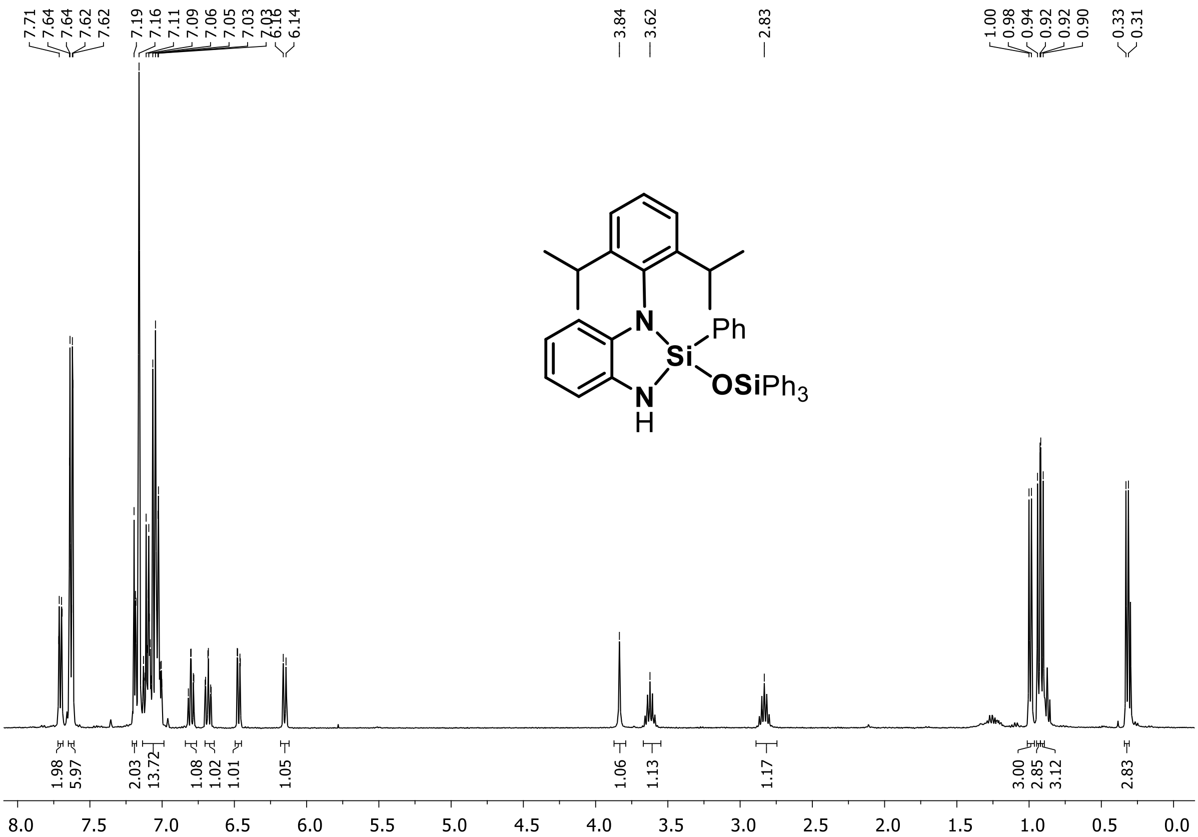

Figure S6. ^{1}H NMR spectrum (C_6D_6, 298 K) of **3**.

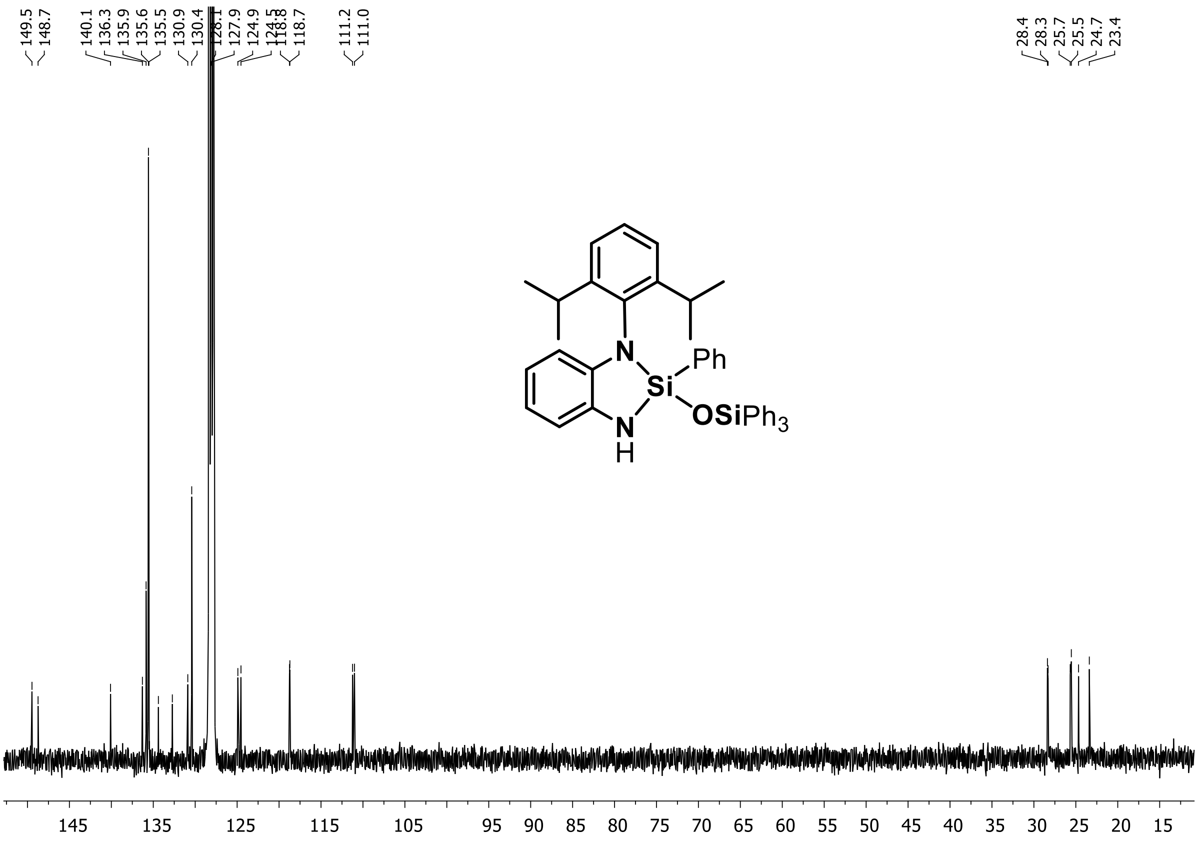

Figure S7. $^{13}C\{^1H\}$ NMR spectrum (C_6D_6, 298 K) of **3**.

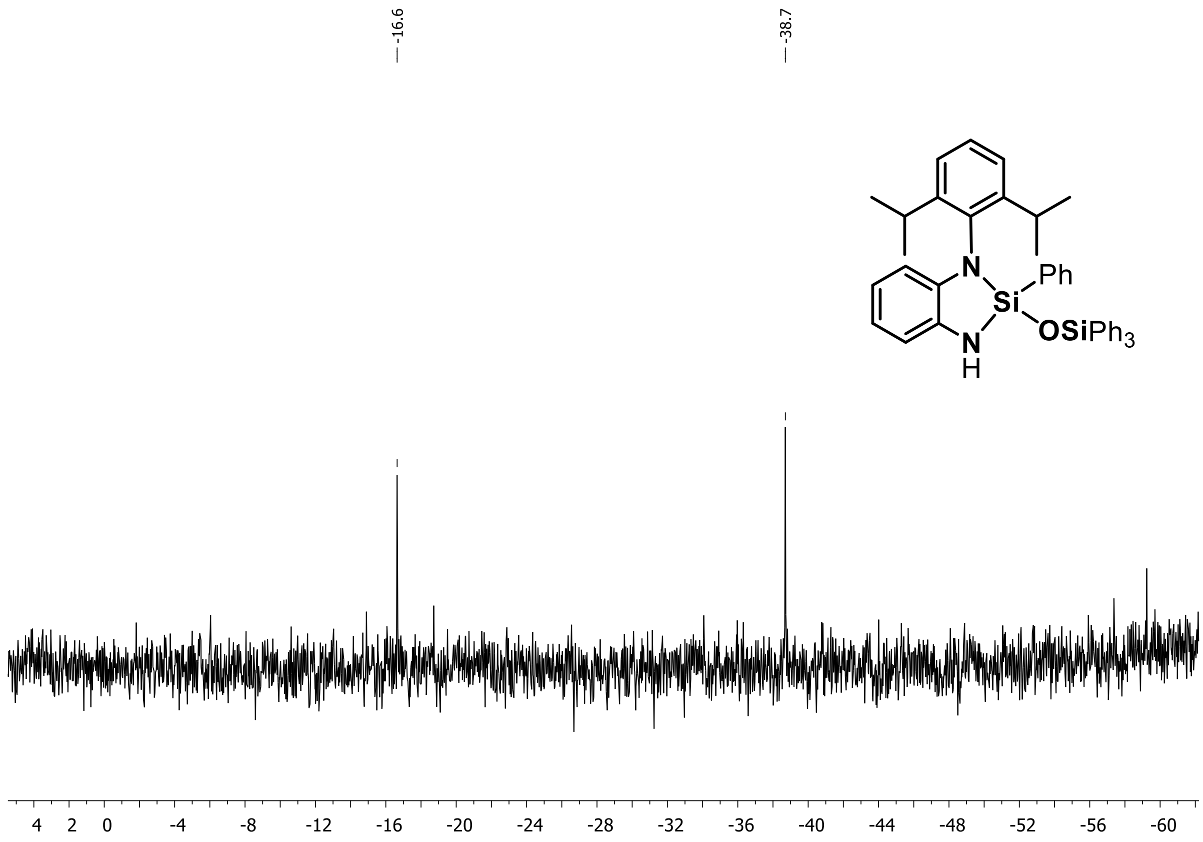

Figure S8. $^{29}Si\{^1H\}$ NMR spectrum (C_6D_6, 298 K) of **3**.

2.3. Synthesis of Compound 5

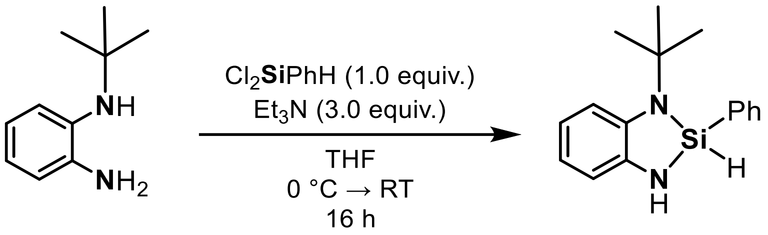

4 **5**

Compound **4** was prepared according to previously published literature procedures.[93,109,110]

Compound **4** (1.48 g, 9.01 mmol, 1.0 equiv.) and triethylamine (3.77 mL, 27.0 mmol, 3.0 equiv.) were dissolved in THF (35 mL) and cooled to 0 °C. Dichlorophenylsilane (1.32 mL, 9.0 mmol, 1.0 equiv.) was added via syringe and the mixture was allowed to warm up to room temperature in 16 hours while stirring. The formed precipitate was filtered off via filter cannula and further extracted with diethyl ether (2 × 6 mL). The phases were combined and all volatiles removed *in vacuo*. The residue was extracted with *n*-pentane (3 × 5 mL) and the remaining solids filtered off via filter cannula. The filtrates were combined and dried *in vacuo* to afford compound **5** as a beige wax (1.86 g, 6.9 mmol, 77%). For further purification, Kugelrohr distillation (140 °C oven temperature, $4.0 \cdot 10^{-3}$ mbar) can be employed, yielding compound **5** as a colorless wax.

^{1}H NMR (400 MHz, C_6D_6, 298 K): δ = 1.30 [s, 9H, CHCH_3], 2.98 [s, 1H, N*H*], 6.14 [s, 1H, Si*H*], 6.47–6.51 [dd, 1H, $^{3}J_{H\text{-}H}$ = 7.1 Hz, $^{4}J_{H\text{-}H}$ = 1.8 Hz, H_{Ar}], 6.77–6.87 [td, 2H, $^{3}J_{H\text{-}H}$ = 6.9 Hz, $^{4}J_{H\text{-}H}$ = 1.6 Hz, H_{Ar}], 6.93–6.98 [m, 1H, $^{3}J_{H\text{-}H}$ = 7.5 Hz, H_{Ar}], 7.10–7.15 [m, 2H, H_{Ar}], 7.16–7.19 [m, 1H, H_{Ar}], 7.46–7.51 [m, 2H, H_{Ar}]. **^{13}C{^{1}H} NMR** (101 MHz, C_6D_6, 298 K): δ = 29.6 [s, C(CH$_3$)$_3$], 52.5 [s, C(CH$_3$)$_3$], 111.3 [s, CH$_{Ar}$], 112.9 [s, CH$_{Ar}$], 117.7 [s, CH$_{Ar}$], 117.9 [s, CH$_{Ar}$], 128.5 [s, CH$_{Ar}$], 131.1 [s, CH$_{Ar}$], 134.0 [s, CH$_{Ar}$], 138.1 [s, C_{Ar}], 138.4 [s, C_{Ar}], 140.7 [s, C_{Ar}]. **^{29}Si{^{1}H} NMR** (79 MHz, C_6D_6, 298 K): δ = −18.0 [s, *Si*]. **^{29}Si NMR** (79 MHz, C_6D_6, 298 K): δ = −18.4 [dm, $^{1}J_{SiH}$ = 233.0 Hz, *Si*]. **CHN Analysis** $C_{16}H_{20}N_2Si$ • 0.1 DCM: calculated: C 69.83, H 7.35, N 10.12; found: C 69.87, H 7.52, N 10.05. **HR-MS(EI+)**, calculated m/z for $C_{16}H_{20}N_2Si$ [M+H^{+}]: 268.13903; found: 268.13984.

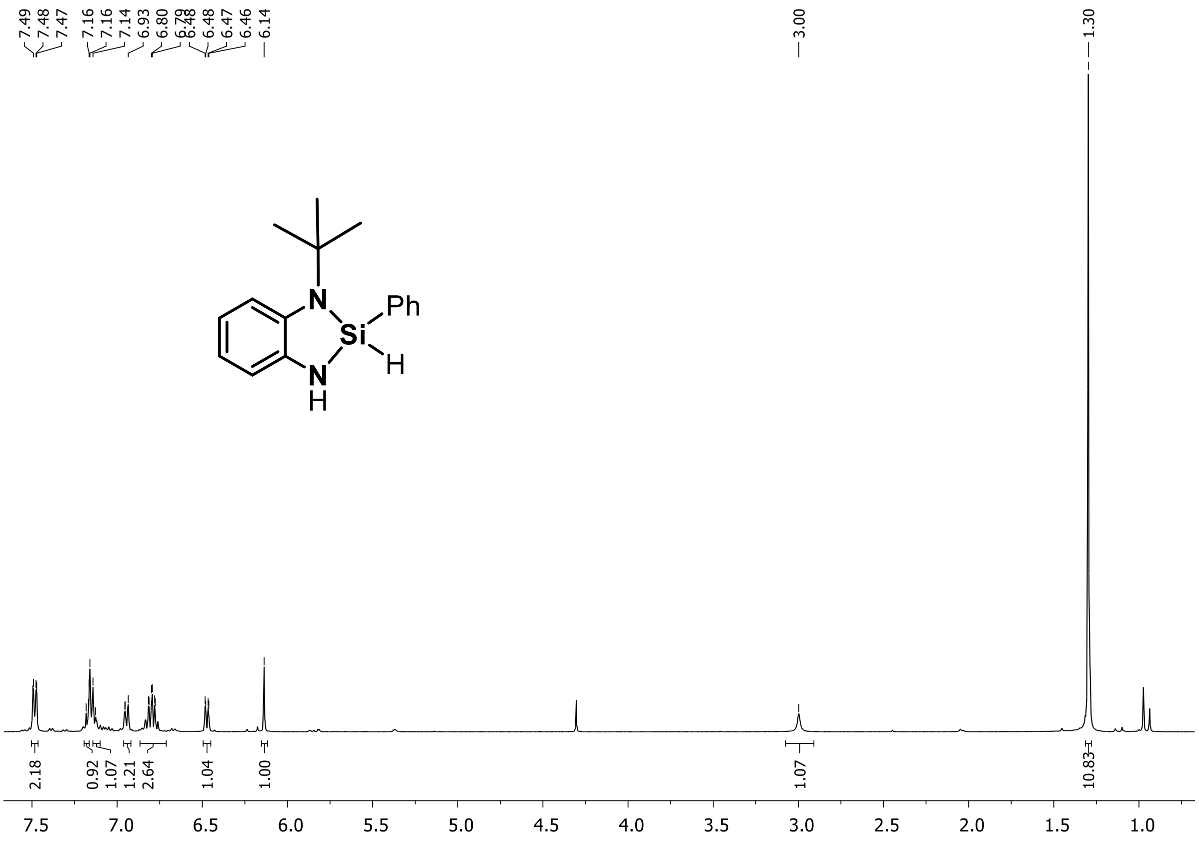

Figure S9. ^{1}H NMR spectrum (C_6D_6, 298 K) of **5** before distillation.

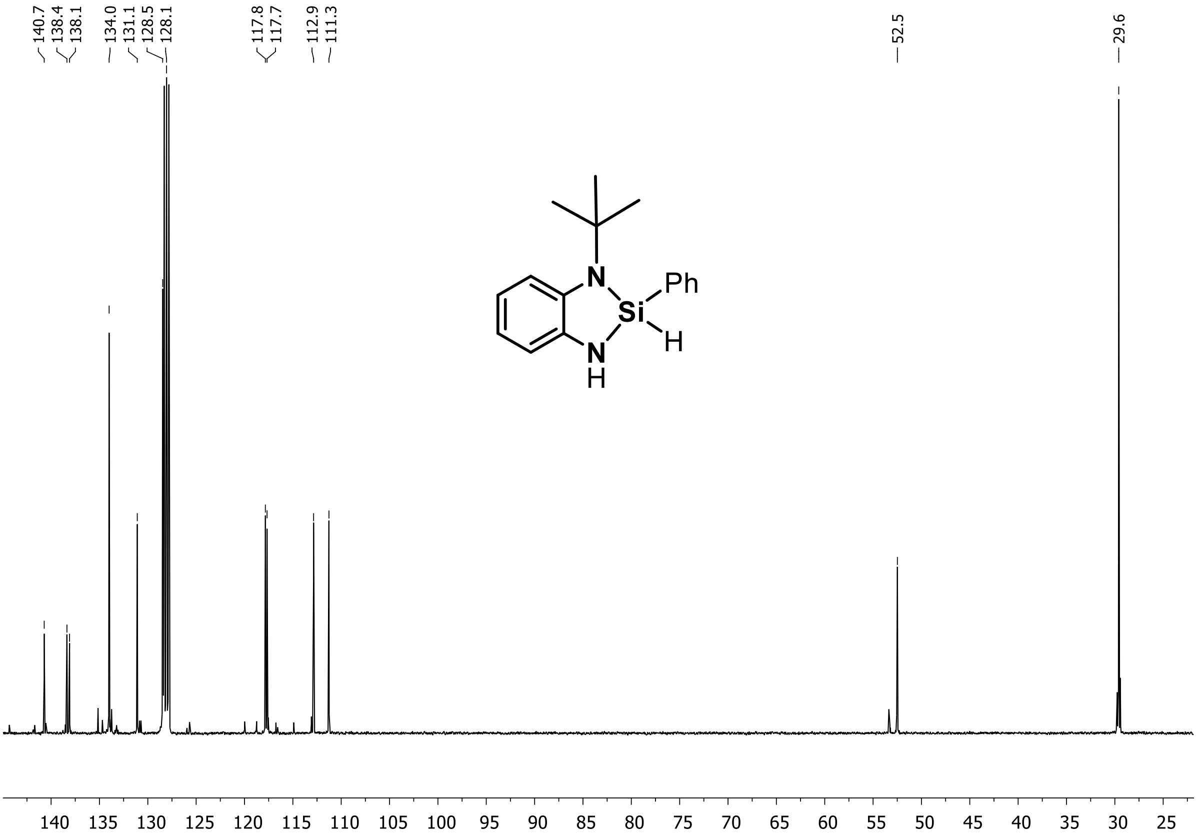

Figure S10. $^{13}C\{^{1}H\}$ NMR spectrum (C_6D_6, 298 K) of **5** before distillation.

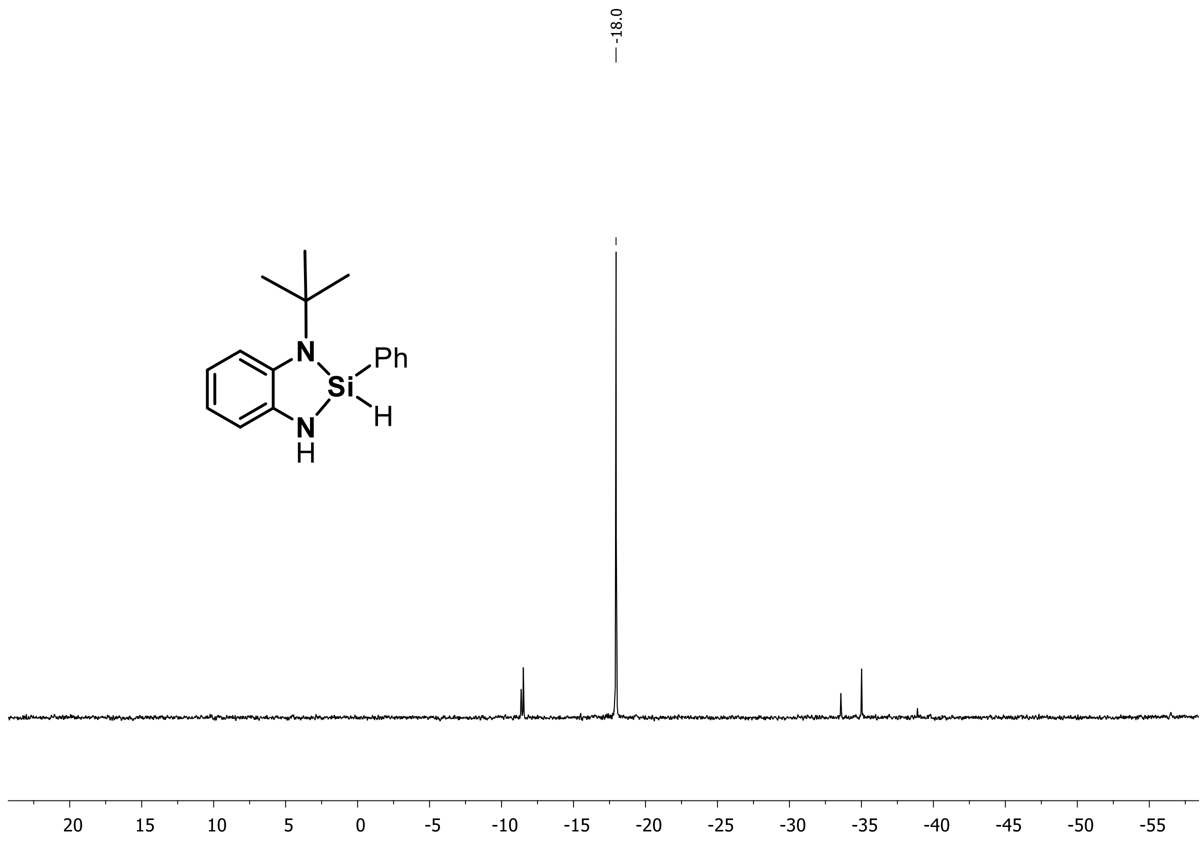

Figure S11. $^{29}Si\{^1H\}$ NMR spectrum (C_6D_6, 298 K) of **5** before distillation.

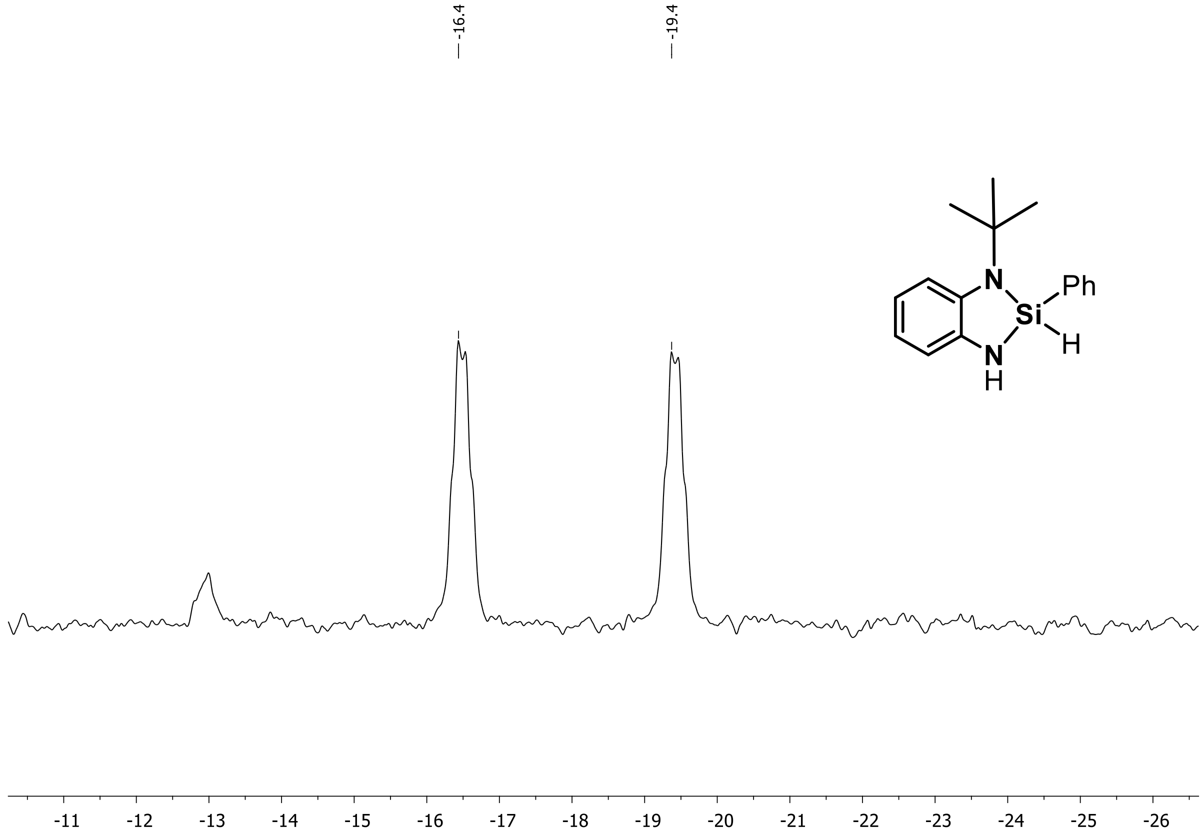

Figure S12. ^{29}Si NMR spectrum (C_6D_6, 298 K) of **5** before distillation.

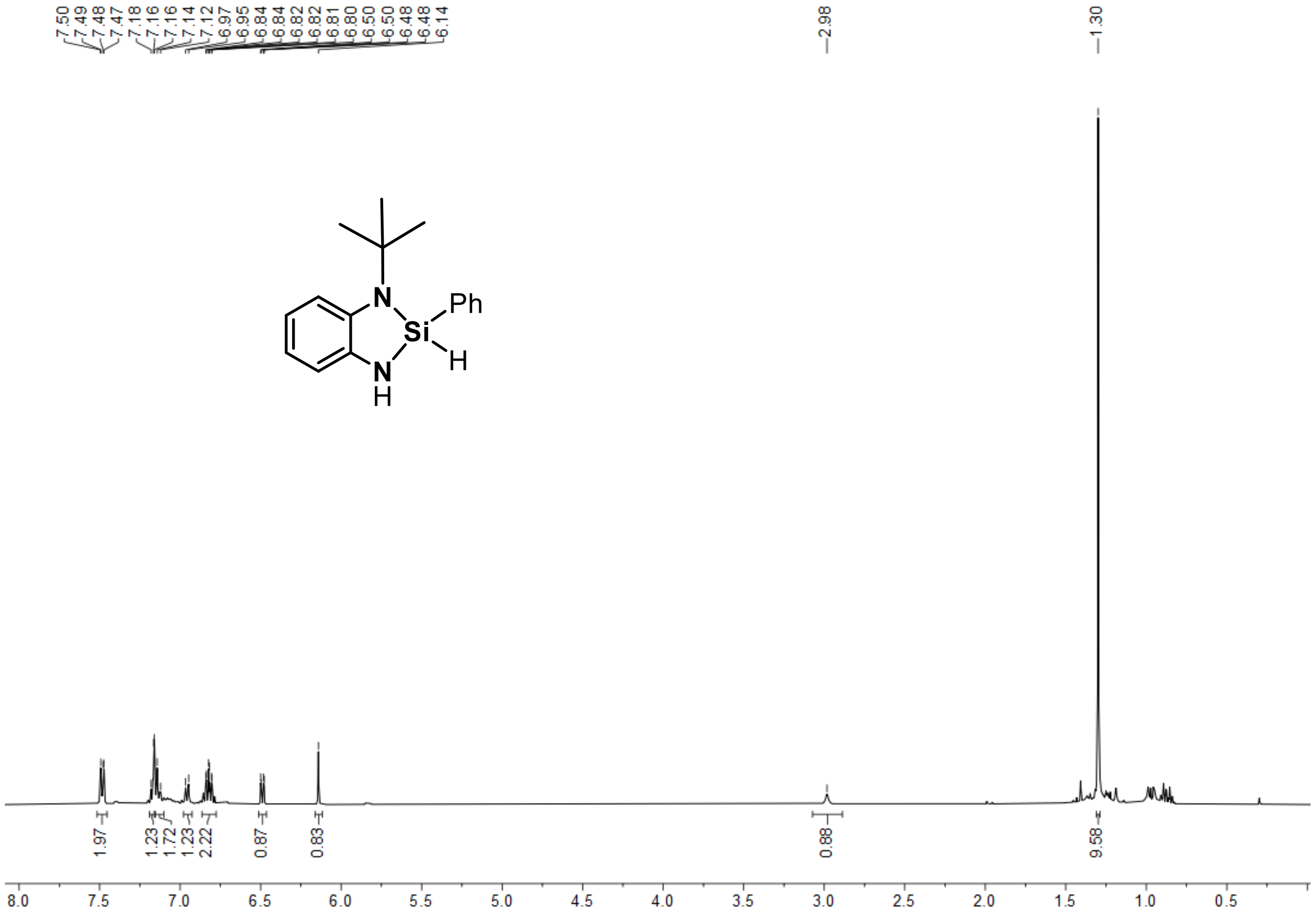

Figure S13. ^{1}H NMR spectrum (C_6D_6, 298 K) of **5**.

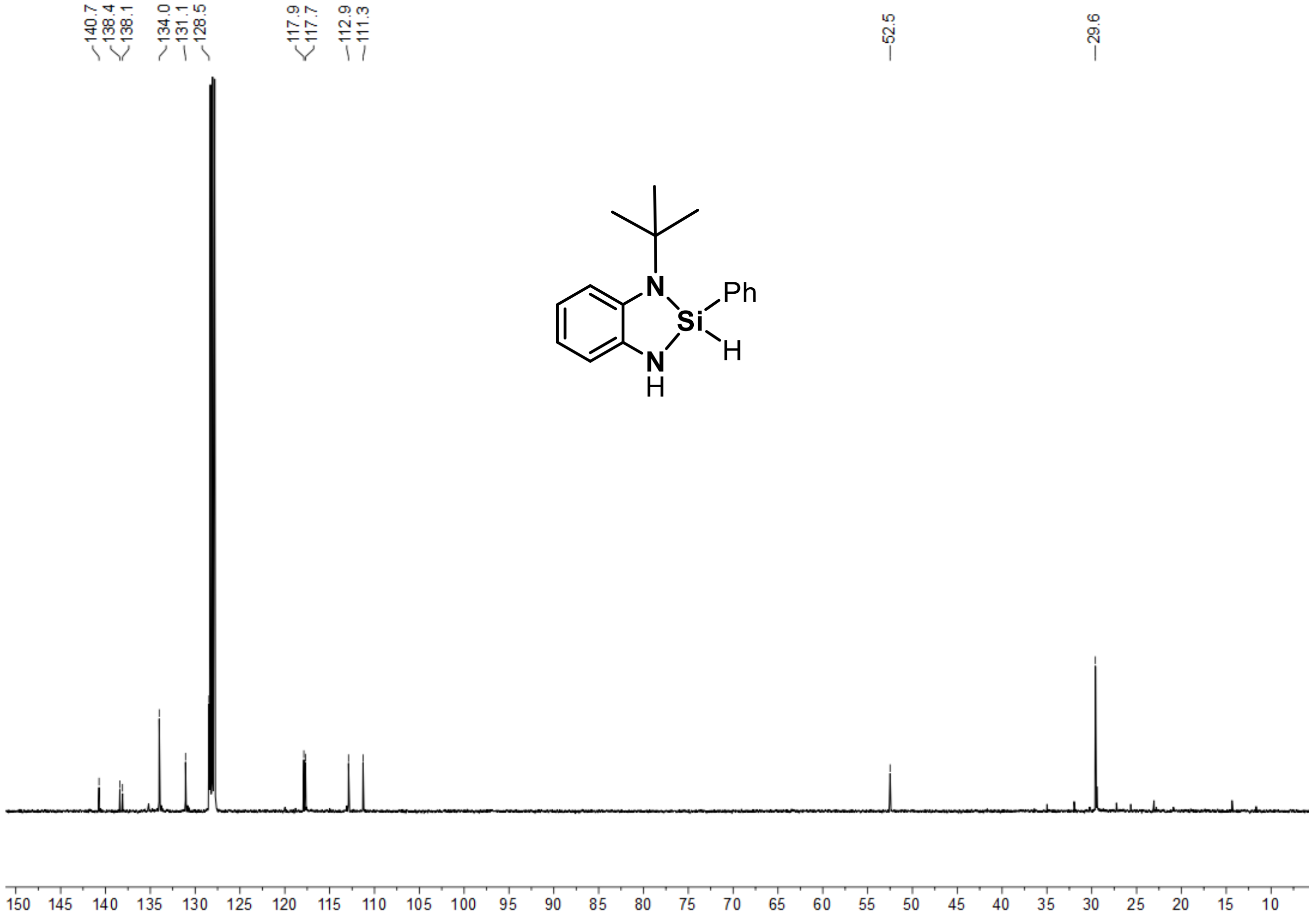

Figure S14. $^{13}C\{^{1}H\}$ NMR spectrum (C_6D_6, 298 K) of **5**.

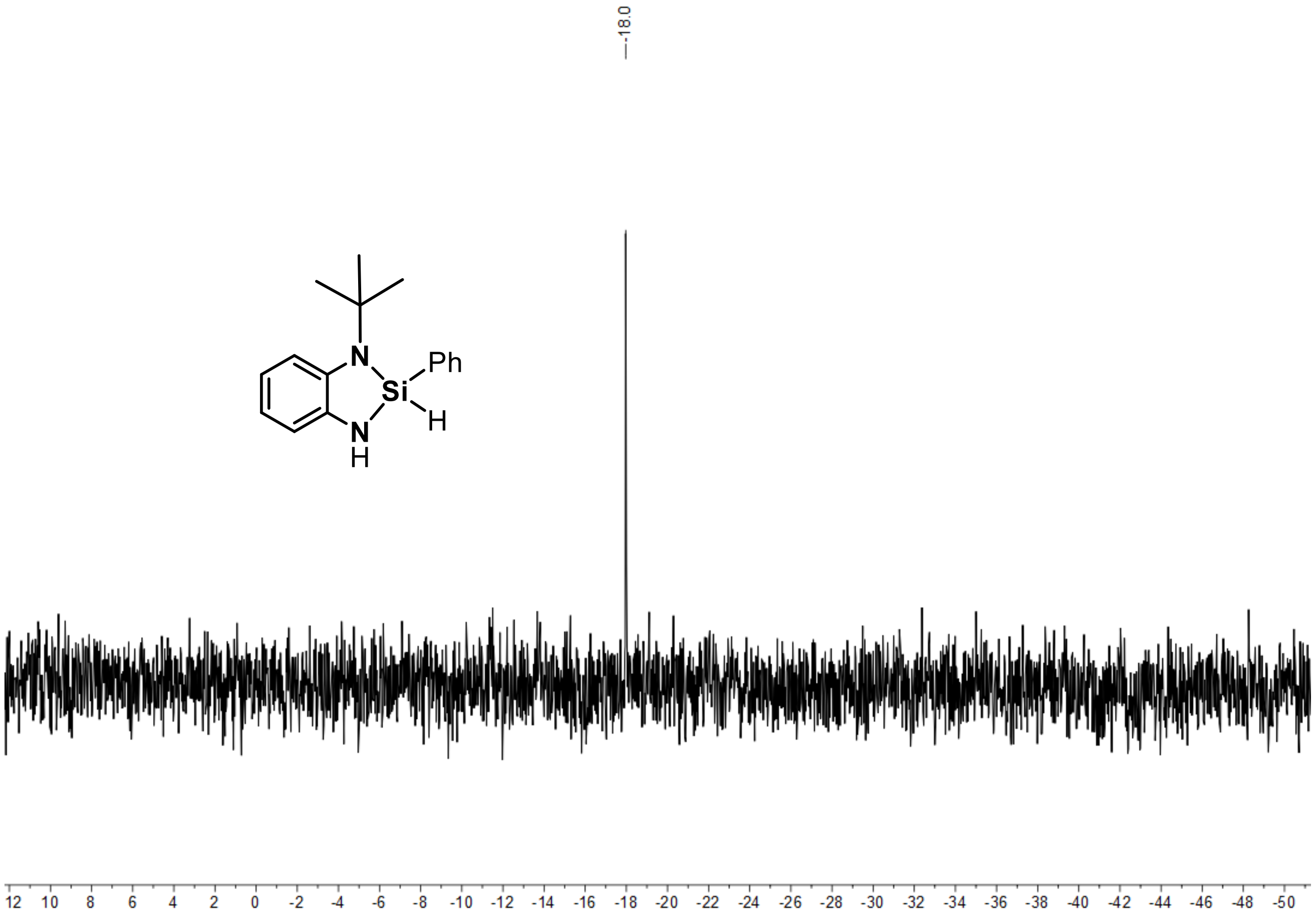

Figure S15. $^{29}Si\{^{1}H\}$ NMR spectrum (C_6D_6, 298 K) of **5**.

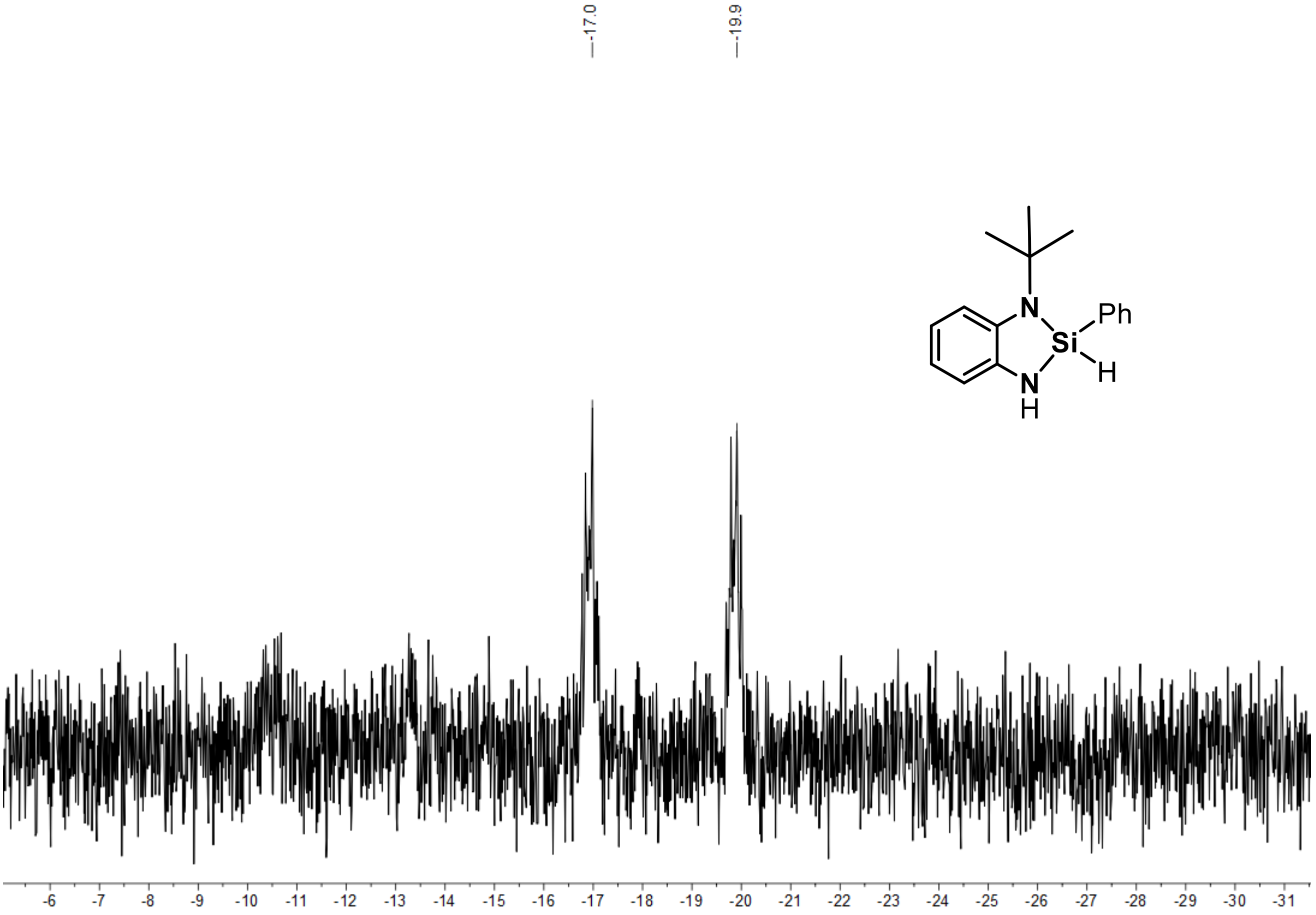

Figure S16. ^{29}Si NMR spectrum (C_6D_6, 298 K) of **5**.

2.4. Synthesis of Compound 6

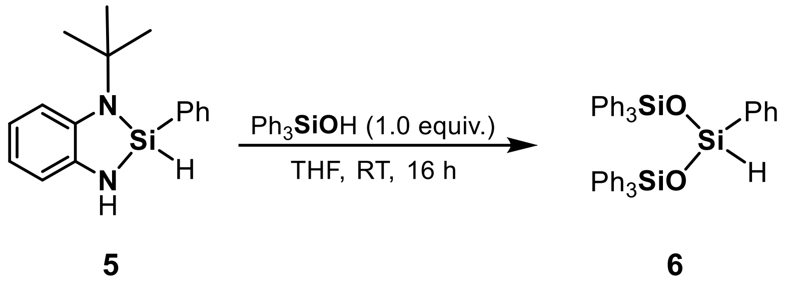

Compound **5** (1.34 g, 5.0 mmol, 1.0 equiv.) and triphenylsilanol (1.38 g, 5.0 mmol, 1.0 equiv.) were dissolved in THF (50 mL) and stirred at room temperature for 16 hours. Next, all volatiles were removed *in vacuo*. The residue was extracted with *n*-pentane (50 mL) and the solids filtered off via filter cannula. Crystals suitable for single-crystal X-ray diffraction analysis were obtained by storing the filtrate at 8 °C for one day. The crystals were isolated via filter cannula and washed with *n*-pentane to afford compound **6** as an off-white crystalline solid (Isolated Yield: 680 mg, 1.0 mmol, 20%).

In an analogous experiment, hexamethylbenzene (0.5 equiv.) was added as an internal standard before purification and an aliquot was taken and analyzed via ^{1}H NMR spectroscopy, revealing an NMR-based yield of the reaction of 44% (Theoretical Yield: 50%).

^{1}H NMR (400 MHz, C_6D_6, 298 K): δ = 5.80 [s, 1H, Si*H*], 6.97–7.15 [m, 20H, H_{Ar}], 7.17–7.19 [m, 1H, H_{Ar}], 7.57–7.61 [m, 2H, H_{Ar}], 7.63–7.68 [m, 12H, H_{Ar}]. **^{13}C{^{1}H} NMR** (101 MHz, C_6D_6, 298 K): δ = 138.2 [s, CH_{Ar}], 130.2 [s, CH_{Ar}], 130.7 [s, CH_{Ar}], 133.8 [s, CH_{Ar}], 135.6 [s, CH_{Ar}], 135.9 [s, C_{Ar}]. **^{29}Si{^{1}H} NMR** (79 MHz, C_6D_6, 298 K): δ = −46.6 [s, *Si*H], −17.9 [s, *Si*Ph$_3$]. **^{29}Si NMR** (79 MHz, C_6D_6, 298 K): δ = −46.2 [dm, $^1J_{SiH}$ = 250.5 Hz, *Si*H], −17.4 [m, *Si*Ph$_3$]. **CHN Analysis** $C_{42}H_{36}O_2Si_3$: calculated: C 76.78, H 5.52, O 4.87, Si 12.82; found: C 76.68, H 5.43. **HR-MS(FD+)**, calculated m/z for $C_{42}H_{36}O_2Si_3$ [M+H$^+$]: 656.20176; found: 656.20401.

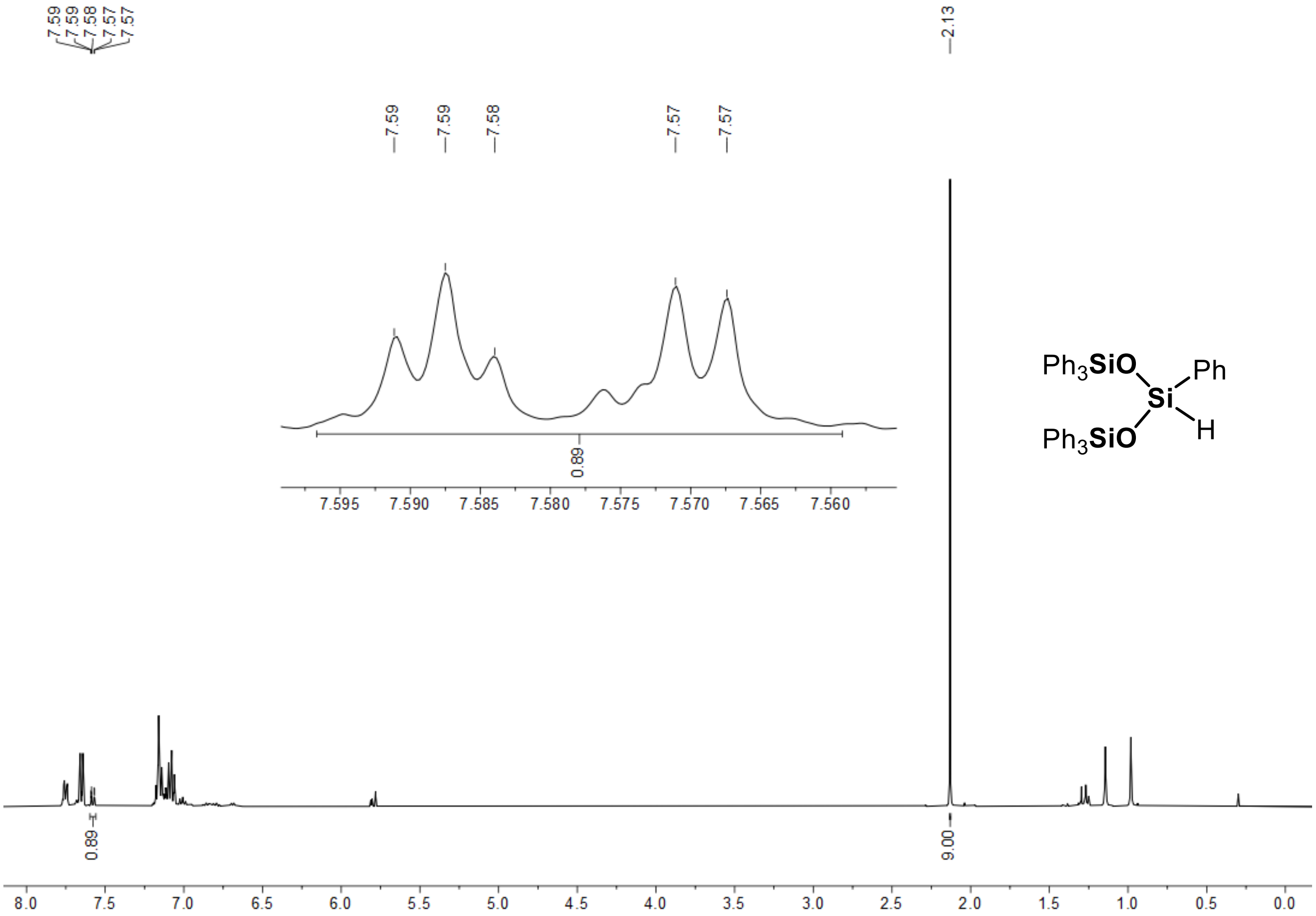

Figure S17. ^{1}H NMR spectrum (C_6D_6, 298 K) of the crude reaction mixture of compound **6** with HMB as internal standard.

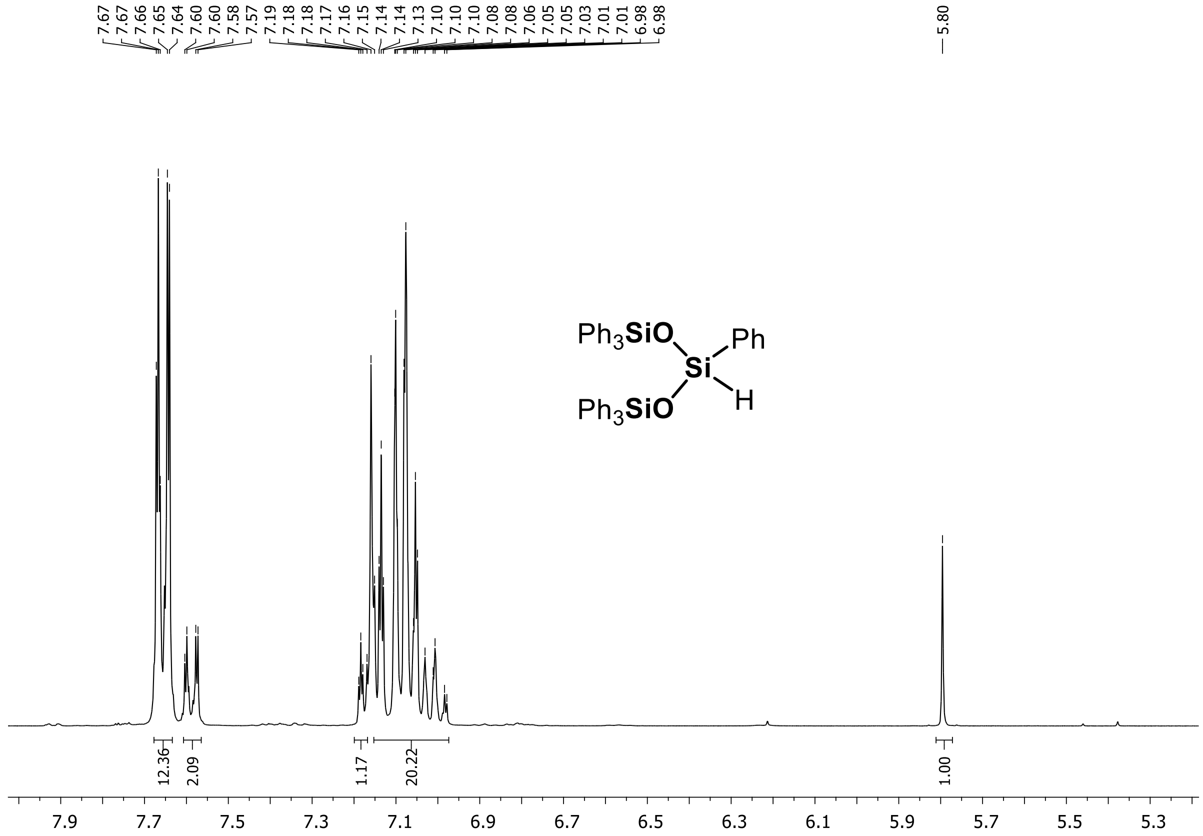

Figure S18. ^{1}H NMR spectrum (C_6D_6, 298 K) of **6**.

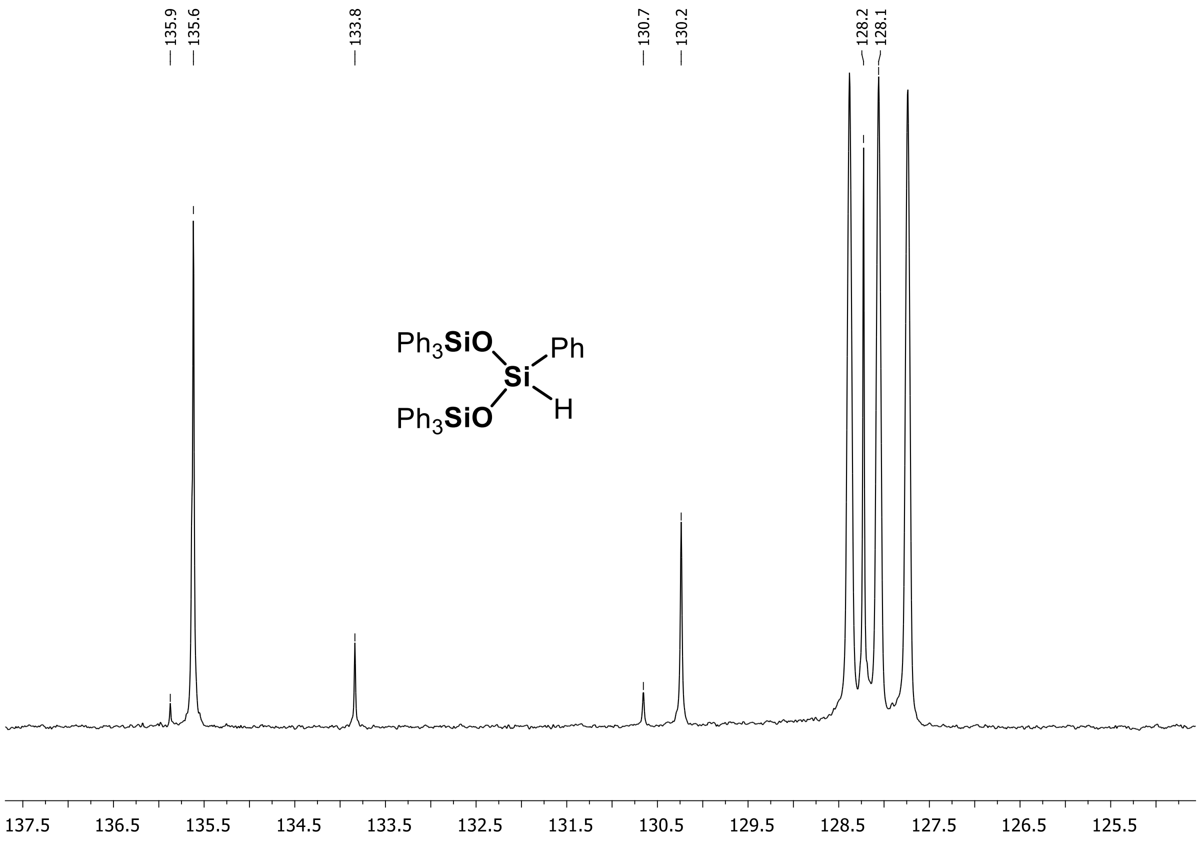

Figure S19. $^{13}C\{^{1}H\}$ NMR spectrum (C_6D_6, 298 K) of **6**.

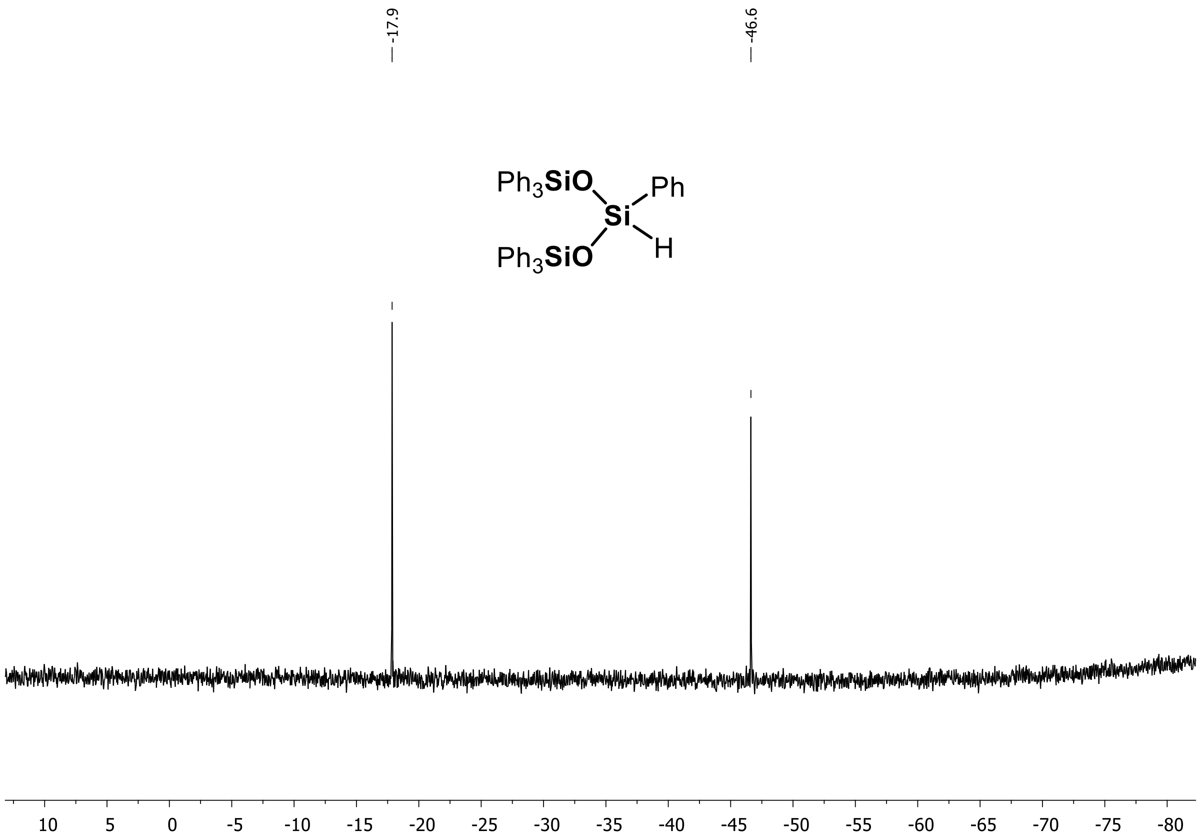

Figure S20. $^{29}Si\{^{1}H\}$ NMR spectrum (C_6D_6, 298 K) of **6**.

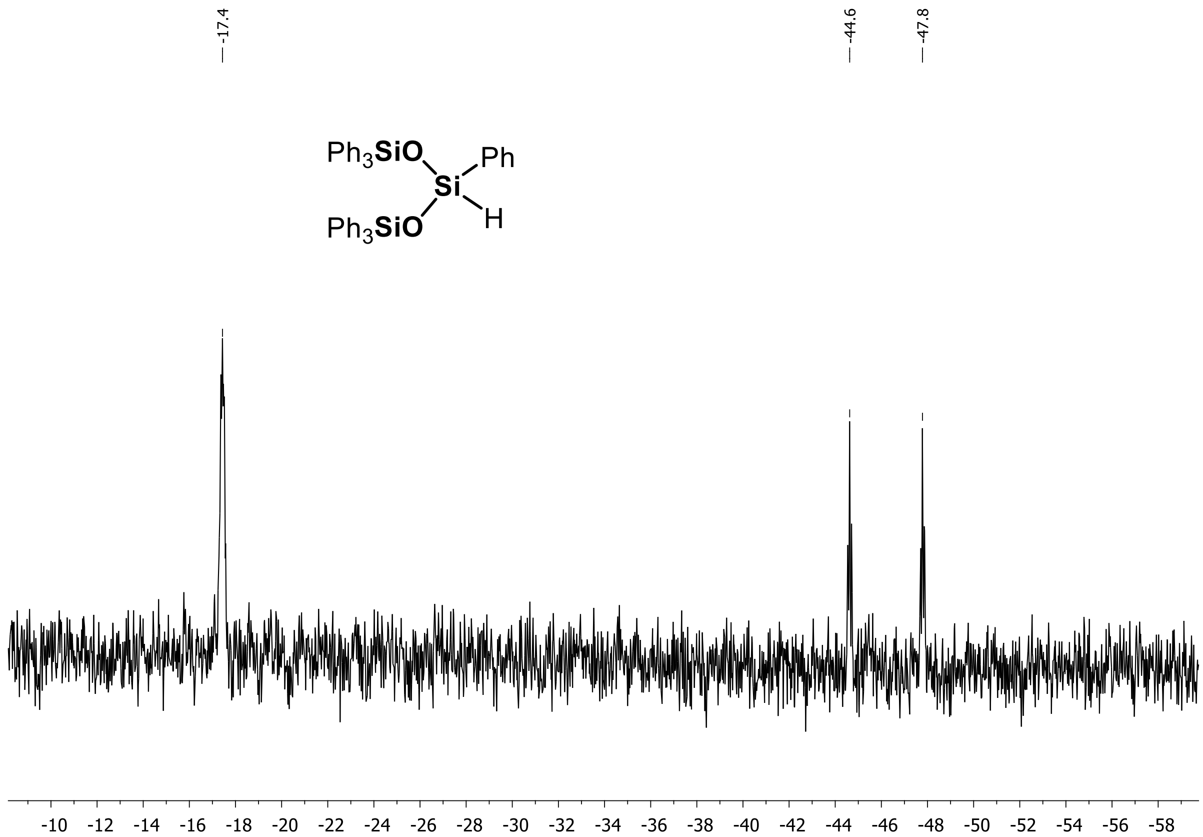

Figure S21. ^{29}Si NMR spectrum (C_6D_6, 298 K) of **6**.

2.5. Synthesis of Compound 7

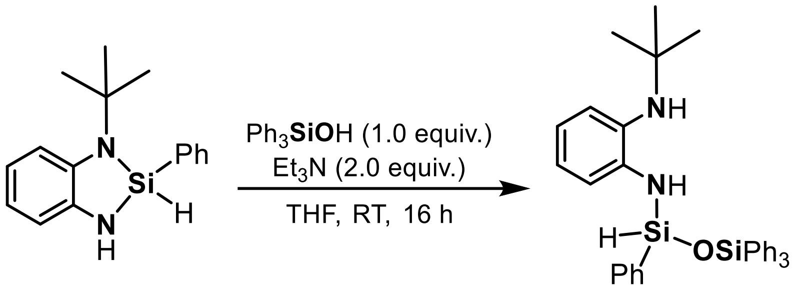

5 **7**

Compound **5** (130 mg, 0.48 mmol, 1.0 equiv.) and triethylamine (0.13 mL, 0.96 mmol, 2.0 equiv.) were dissolved in THF (10 mL). Triphenylsilanol (133 mg, 0.48 mmol, 1.0 equiv.) was added and the solution was stirred at room temperature for 16 hours. Next, all volatiles were removed *in vacuo*. The residue was dissolved in *n*-pentane (20 mL) and cooled to –80 °C, after which precipitation was observed. The solids were isolated via cannula filtration. Upon warming up to room temperature, the solids turned into a brown wax, which was layered with *n*-pentane (1 mL) and stored at 8 °C. Crystals suitable for single-crystal X-ray diffraction analysis were obtained after two weeks. The crystals were isolated via filter cannula and washed with *n*-pentane to afford compound **7** as a white crystalline solid (Isolated crystalline Yield: 8 mg, 0.01 mmol, 3%).

In an analogous experiment, hexamethylbenzene (0.5 equiv.) was added as an internal standard before purification and an aliquot was taken and analyzed via ^{1}H NMR spectroscopy, revealing an NMR-based yield of the reaction of 80%.

^{1}H NMR (400 MHz, C_6D_6, 298 K): δ = 0.98 [s, 9H, C(C*H*$_3$)$_3$], 2.03 [s, 1H, *t*Bu-N*H*], 5.71 [d, 1H, $^{3}J_{HH}$ = 3.0 Hz, Si-N*H*], 5.81 [d, 1H, $^{3}J_{HH}$ = 3.1 Hz, Si*H*], 6.67–6.72 [m, 1H, H_{Ar}], 6.77–6.83 [m, 2H, H_{Ar}], 7.05–7-16 [m, 8H, H_{Ar}], 7.16–7.21 [m, 3H, H_{Ar}], 7.73–7.78 [m, 8H, H_{Ar}]. **^{13}C{^{1}H} NMR** (101 MHz, C_6D_6, 298 K): δ = 29.8 [s, C(*C*H$_3$)$_3$], 53.4 [s, *C*(CH$_3$)$_3$], 117.0 [s, CH_{Ar}], 118.9 [s, CH_{Ar}], 125.7 [s, CH_{Ar}], 128.1 [s, CH_{Ar}], 128.2 [s, CH_{Ar}], 128.4 [s, CH_{Ar}], 130.3 [s, CH_{Ar}], 130.7 [s, CH_{Ar}], 133.1 [s, C_{Ar}], 134.3 [s, CH_{Ar}], 135.3 [s, C_{Ar}], 135.7 [s, CH_{Ar}], 135.8 [s, C_{Ar}], 144.2 [s, C_{Ar}]. **^{29}Si{^{1}H} NMR** (79 MHz, C_6D_6, 298 K): δ = −38.7 [s, *Si*H], −17.6 [s, *Si*Ph$_3$]. **^{29}Si NMR** (79 MHz, C_6D_6, 298 K): δ = −38.7 [d, $^{1}J_{SiH}$ = 240.6 Hz, *Si*H], −17.6 [s, *Si*Ph$_3$]. **CHN Analysis** $C_{34}H_{36}N_2OSi_2$: calculated: C 74.95, H 6.66, N 5.14, O 2.94, Si 10.31; found: C 75.00, H 6.53, N 5.10. **HR-MS(FD+)**, calculated m/z for $C_{34}H_{36}N_2OSi_2$ [M+H$^+$]: 544.23607; found: 544.23416.

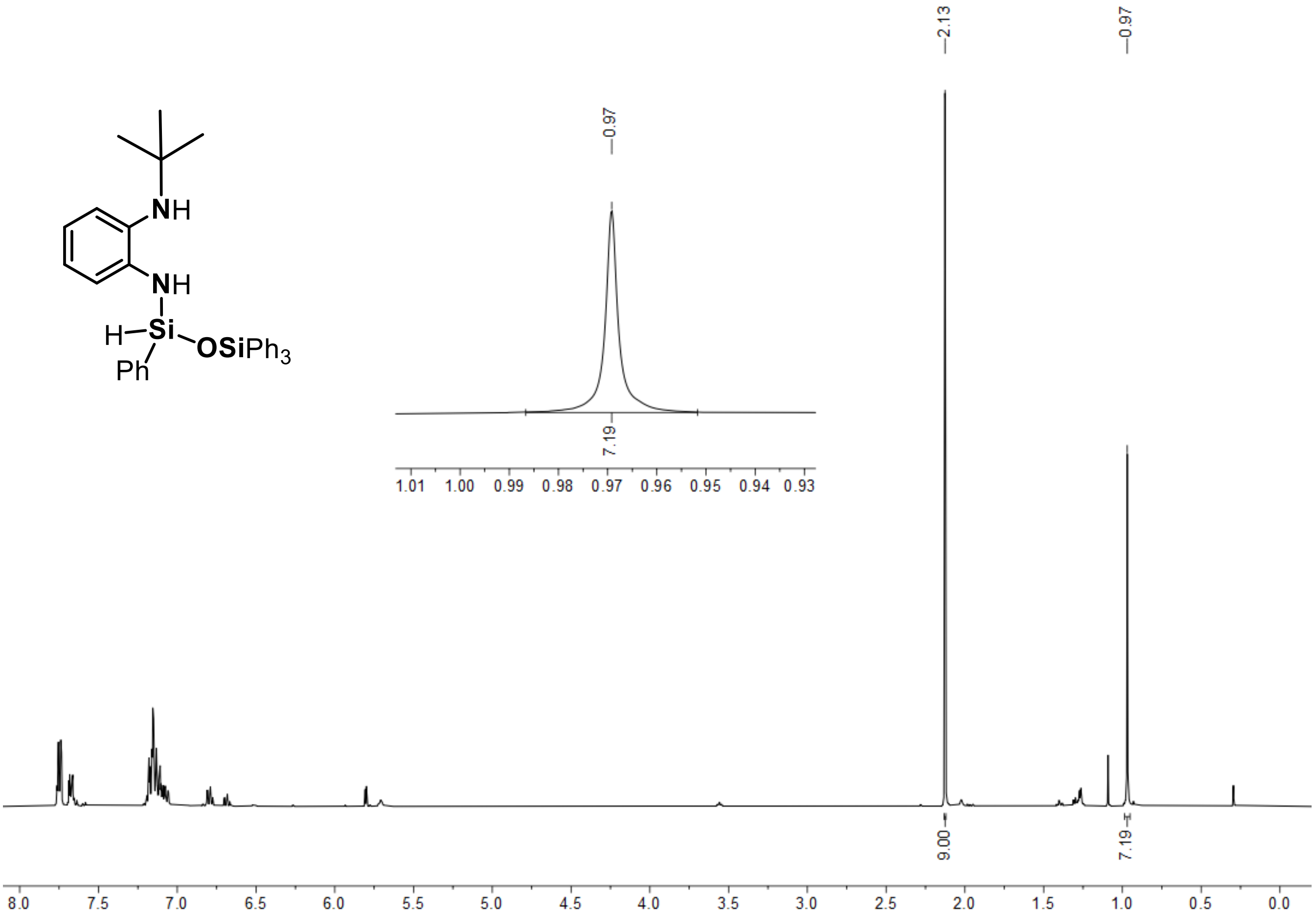

Figure S22. ^{1}H NMR spectrum (C_6D_6, 298 K) of the crude reaction mixture of compound **7** with HMB as internal standard.

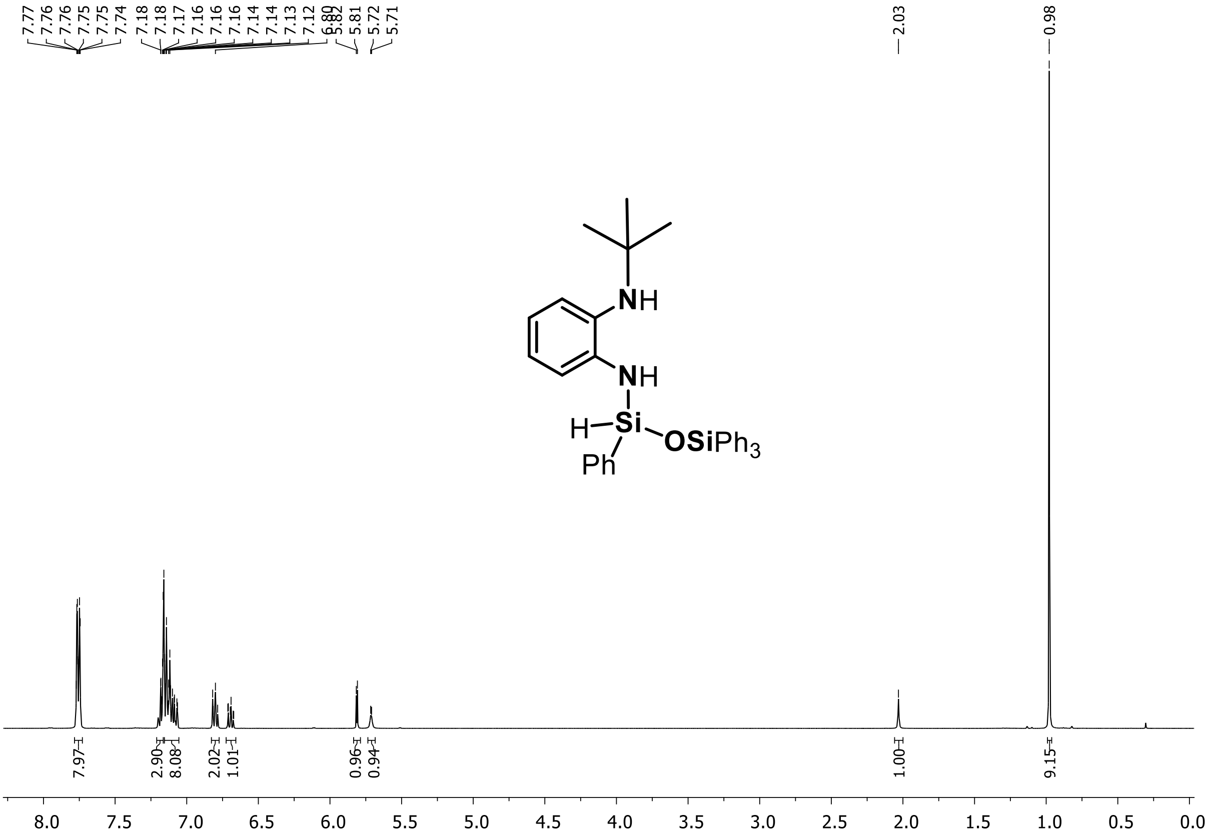

Figure S23. ^{1}H NMR spectrum (C_6D_6, 298 K) of **7**.

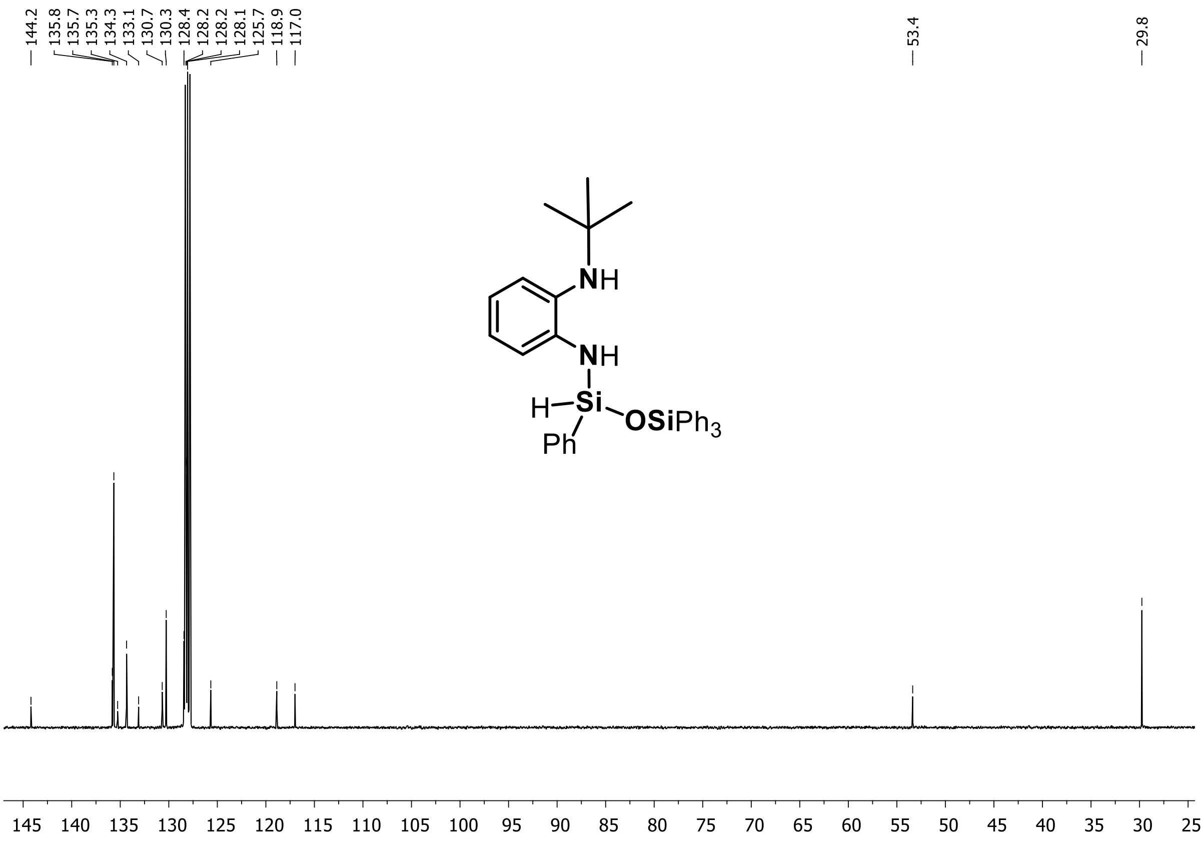

Figure S24. $^{13}C\{^{1}H\}$ NMR spectrum (C_6D_6, 298 K) of **7**.

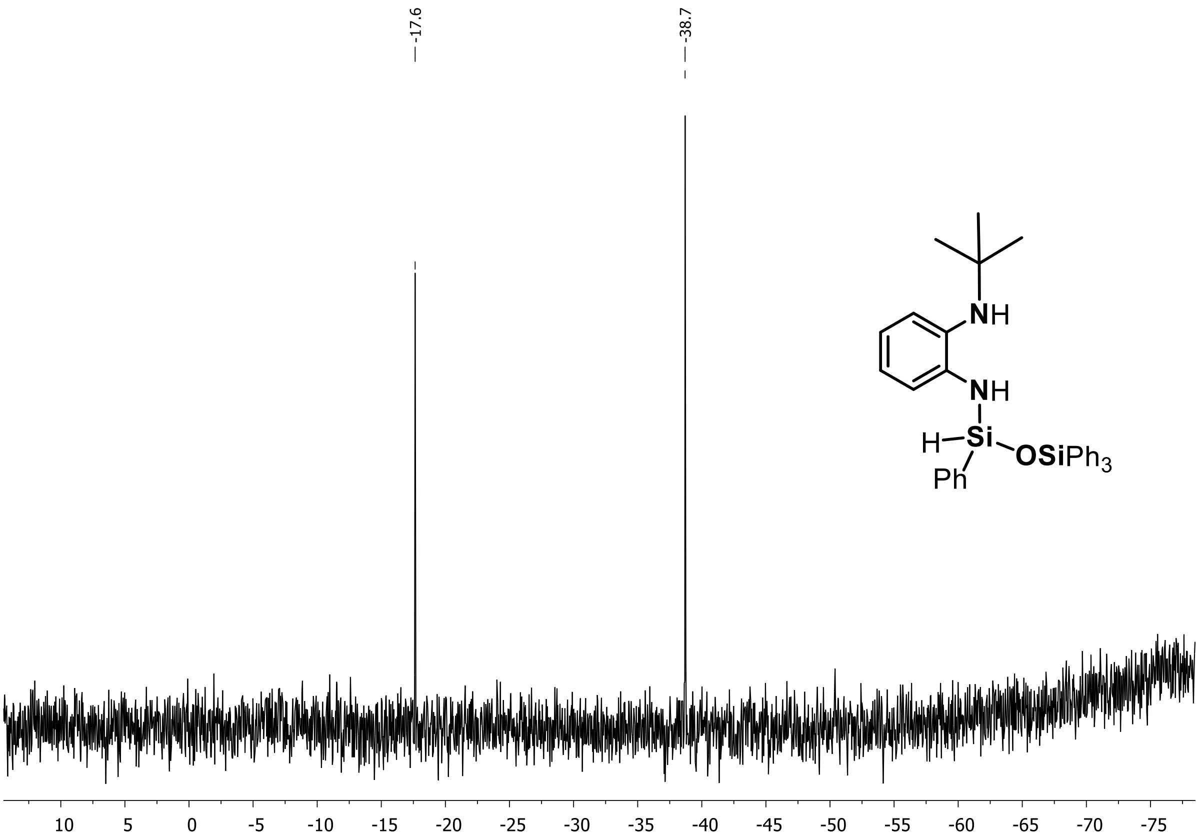

Figure S25. $^{29}Si\{^{1}H\}$ NMR spectrum (C_6D_6, 298 K) of **7**.

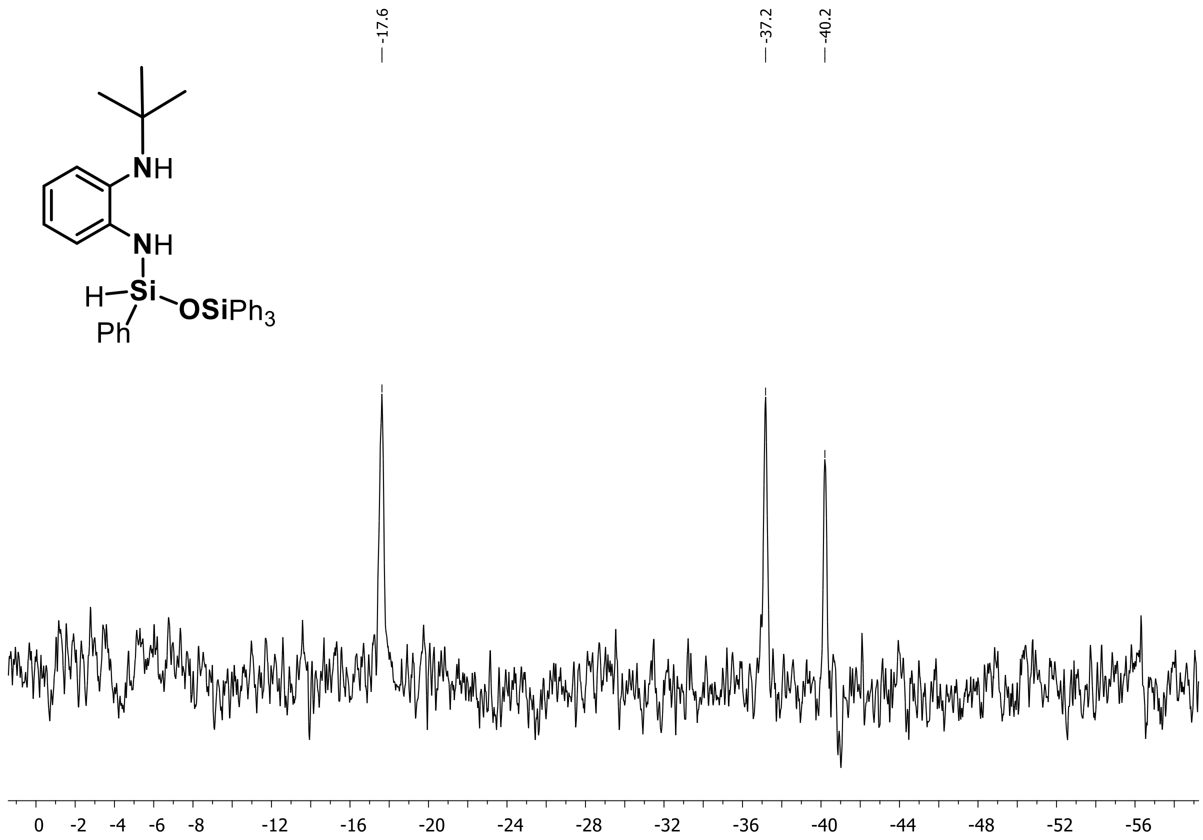

Figure S26. ^{29}Si NMR spectrum (C_6D_6, 298 K) of **7**.

2.6. Synthesis of Compound 8

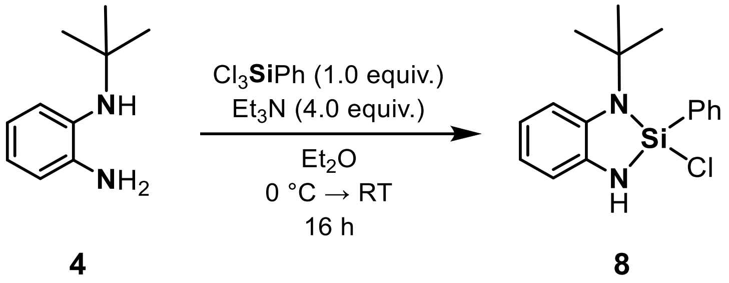

Compound **4** (1.64 g, 10.0 mmol, 1.0 equiv.) and triethylamine (5.58 mL, 40.0 mmol, 4.0 equiv.) were dissolved in diethyl ether (30 mL) and cooled to 0 °C. Trichlorophenylsilane (1.60 mL, 10.0 mmol, 1.0 equiv.) was added via syringe and the mixture was allowed to warm up to room temperature in 16 hours while stirring. The formed precipitate was filtered off via filter cannula. The filtrate was dried *in vacuo* to afford compound **8** as a brown wax (3.13 g, 10.0 mmol, >99%).

^{1}H NMR (400 MHz, C_6D_6, 298 K): δ = 1.35 [s, 9H, C(CH_3)$_3$], 3.45 [s, 1H, N*H*], 6.47–6.50 [m, 1H, H_{Ar}], 6.80–6.88 [m, 2H, H_{Ar}], 7.00–7.04 [m, 1H, H_{Ar}], 7.09–7.15 [m, 3H, H_{Ar}], 7.72–7.75 [m, 2H, H_{Ar}]. **^{13}C{^{1}H} NMR** (101 MHz, C_6D_6, 298 K): δ = 29.7 [s, C(CH$_3$)$_3$], 53.2 [s, C(CH$_3$)$_3$], 111.9 [s, CH$_{Ar}$], 114.0 [s, CH$_{Ar}$], 118.5 [s, CH$_{Ar}$], 118.5 [s, CH$_{Ar}$], 128.4 [s, CH$_{Ar}$], 131.4 [s, CH$_{Ar}$], 134.9 [s, C_{Ar}], 135.4 [s, CH$_{Ar}$], 136.4 [s, C_{Ar}], 137.7 [s, C_{Ar}]. **^{29}Si{^{1}H} NMR** (79 MHz, C_6D_6, 298 K): δ = −18.4 [s, *Si*]. **CHN Analysis** $C_{16}H_{19}ClN_2Si$: calculated: C 63.45, H 6.32, Cl 11.70, N 9.25, Si 9.27; found: C 63.07, H 6.56, N 9.25. **HR-MS(EI+)**, calculated m/z for $C_{16}H_{19}ClN_2Si$ [M+H$^+$]: 302.10005; found: 302.09939.

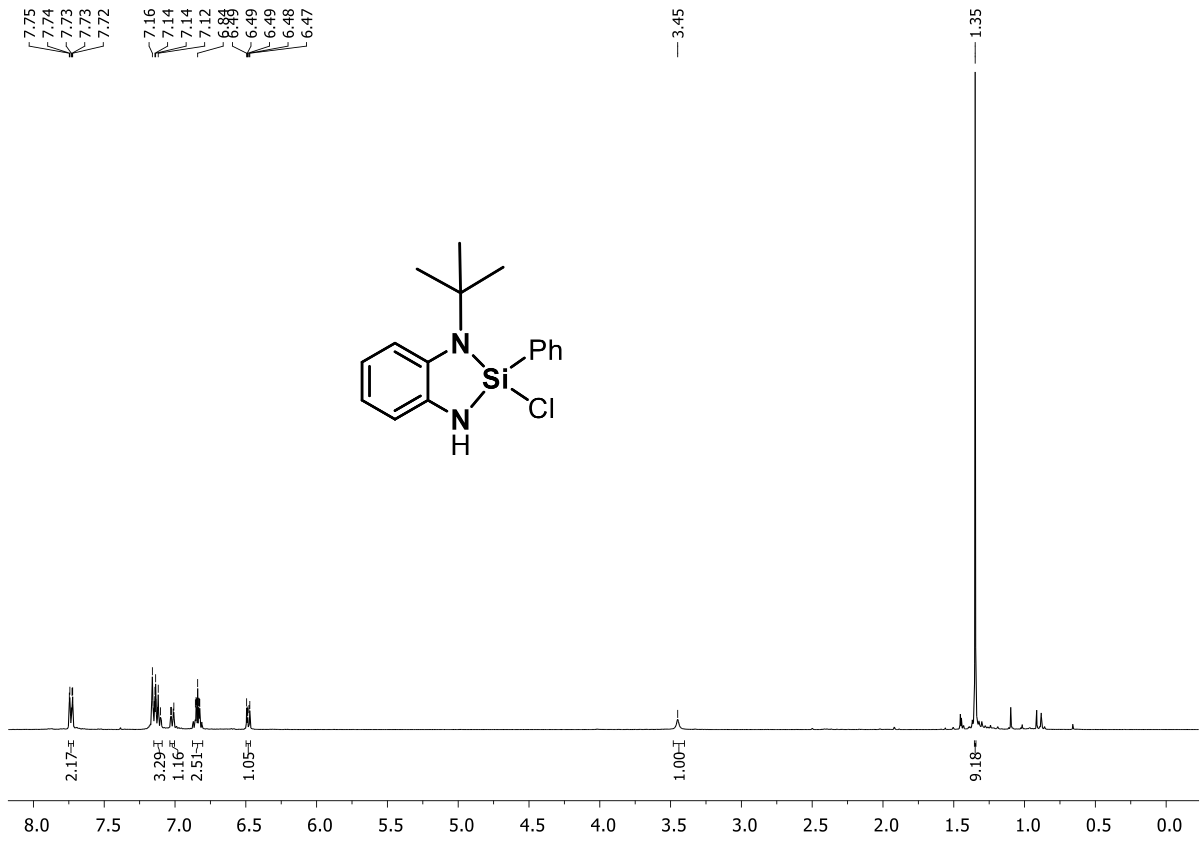

Figure S27. ^{1}H NMR spectrum (C_6D_6, 298 K) of **8**.

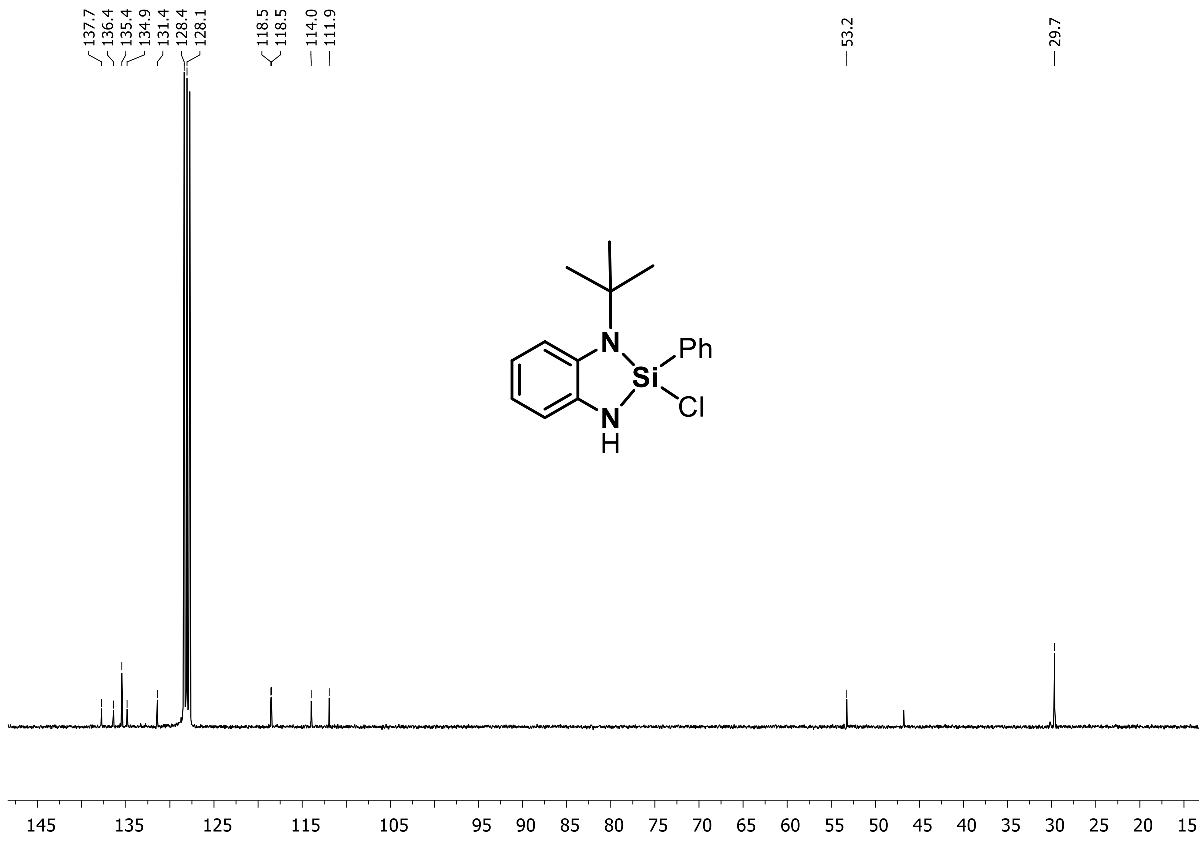

Figure S28. $^{13}C\{^{1}H\}$ NMR spectrum (C_6D_6, 298 K) of **8**.

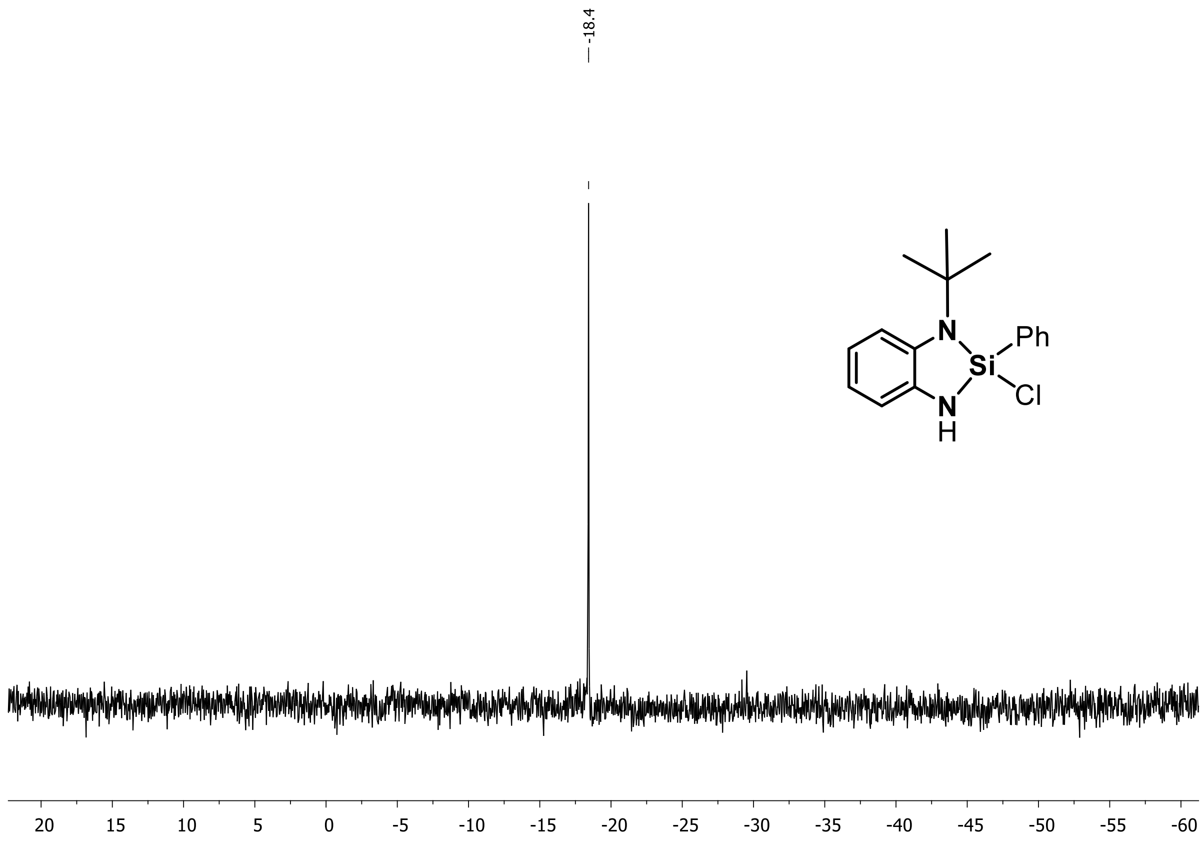

Figure S29. $^{29}Si\{^1H\}$ NMR spectrum (C_6D_6, 298 K) of **8**.

2.7. Synthesis of Compound 9

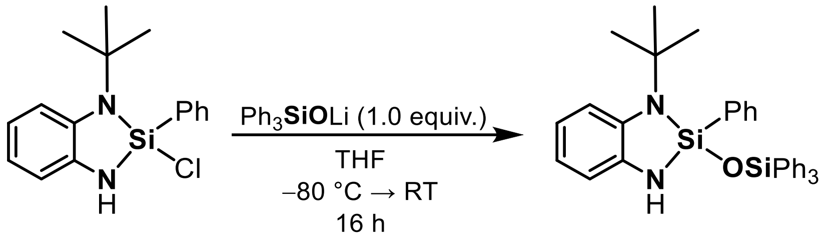

Triphenylsilanol (2.76 g, 10.0 mmol, 1.0 equiv.) was dissolved in THF (30 mL) and *n*-butyllithium (4.40 mL of a 2.5 M solution in hexane, 11.0 mmol, 1.1 equiv.) was added dropwise. The mixture was stirred at room temperature for 10 minutes, after which it was cooled to –80 °C. Compound **8** (20 mL of a 0.5 M solution in THF, 10.0 mmol, 1.0 equiv.) was added via cannula and the reaction mixture was allowed to warm up to room temperature in 16 hours. Then, all volatiles were removed *in vacuo*. The residue was washed with *n*-pentane (3 × 20 mL). The remaining crude product was dissolved in DCM (20 mL) and the remaining solids filtered off via filter cannula. The brown filtrates were dried *in vacuo*, resulting in a brownish solid. Crystals suitable for single-crystal X-ray diffraction analysis were obtained after recrystallization in *n*-pentane (2.36 g, 4.3 mmol, 43%).

^{1}H NMR (400 MHz, C_6D_6, 298 K): δ = 1.27 [s, 9H, C(C*H*$_3$)$_3$], 3.47 [s, 1H, N*H*], 6.31 [dd, 1H, $^3J_{H\text{-}H}$ = 7.4 Hz, $^4J_{H\text{-}H}$ = 1.4 Hz, H_{Ar}], 6.81 [td, 1H, $^3J_{H\text{-}H}$ = 7.5 Hz, $^4J_{H\text{-}H}$ = 1.0 Hz, H_{Ar}], 6.88 [td, 1H, $^3J_{H\text{-}H}$ = 7.6 Hz, $^4J_{H\text{-}H}$ = 1.4 Hz, H_{Ar}], 6.95–6.99 [m, 1H, H_{Ar}], 7.09–7.16 [m, 11H, H_{Ar}], 7.16–7.19 [m, 1H, H_{Ar}], 7.68–7.73 [m, 6H, H_{Ar}], 7.78–7.82 [m, 2H, H_{Ar}]. **^{13}C{^{1}H} NMR** (101 MHz, C_6D_6, 298 K): δ = 29.5 [s, C(*C*H$_3$)$_3$], 52.5 [s, *C*(CH$_3$)$_3$], 111.3 [s, CH_{Ar}], 113.1 [s, CH_{Ar}], 117.7 [s, CH_{Ar}], 117.8 [s, CH_{Ar}], 128.1 [s, CH_{Ar}], 128.3 [s, CH_{Ar}], 130.4 [s, CH_{Ar}], 130.6 [s, CH_{Ar}], 135.6 [s, CH_{Ar}], 135.6 [s, CH_{Ar}], 136.3 [s, C_{Ar}], 138.0 [s, C_{Ar}]. **^{29}Si{^{1}H} NMR** (79 MHz, C_6D_6, 298 K): δ = −40.0 [s, *Si*Ph$_3$], −17.0 [s, *Si*N]. **CHN Analysis** $C_{34}H_{34}N_2OSi_2$: calculated: C 75.23, H 6.31, N 5.16, O 2.95, Si 10.35; found: C 74.58, H 6.11, N 5.85. **HR-MS(FD+)**, calculated m/z for $C_{34}H_{34}N_2OSi_2$ [M+H$^+$]: 542.22042; found: 542.21889.

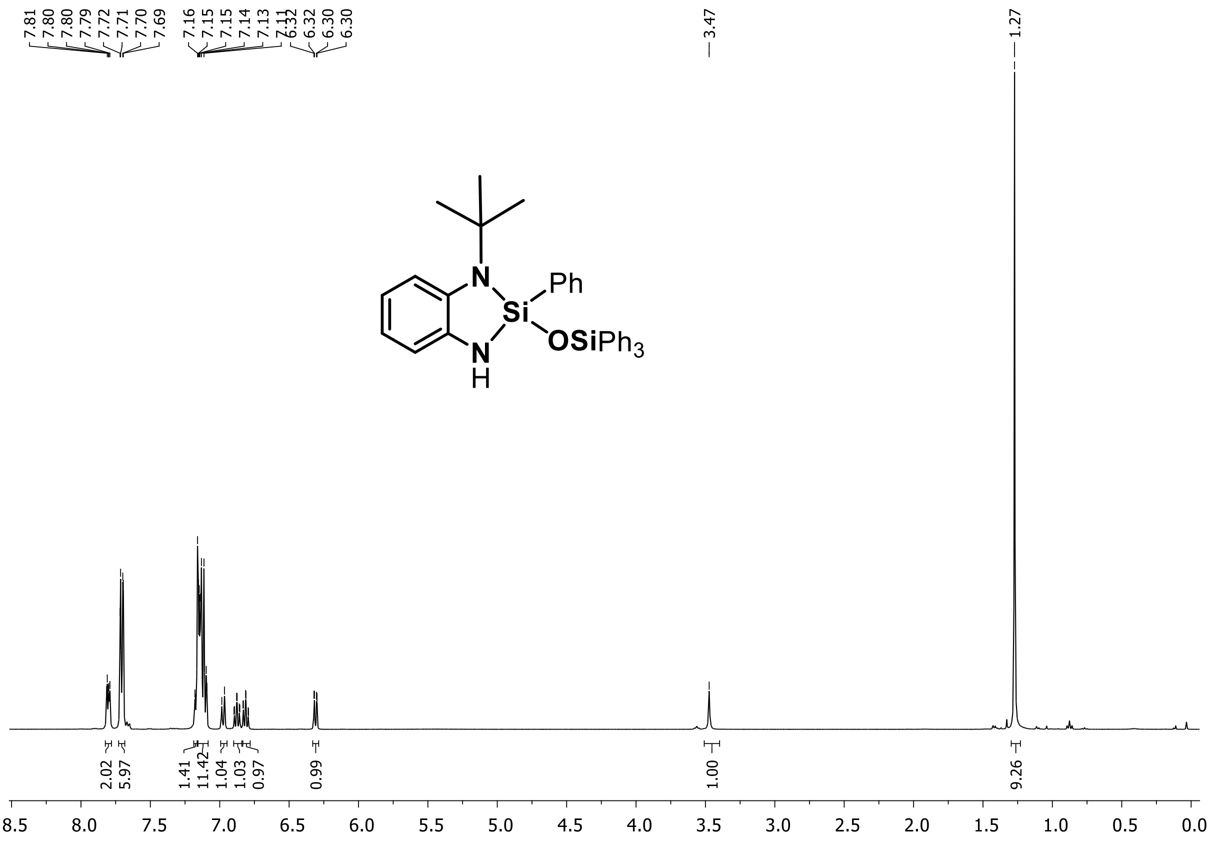

Figure S30. ^{1}H NMR spectrum (C_6D_6, 298 K) of **8**.

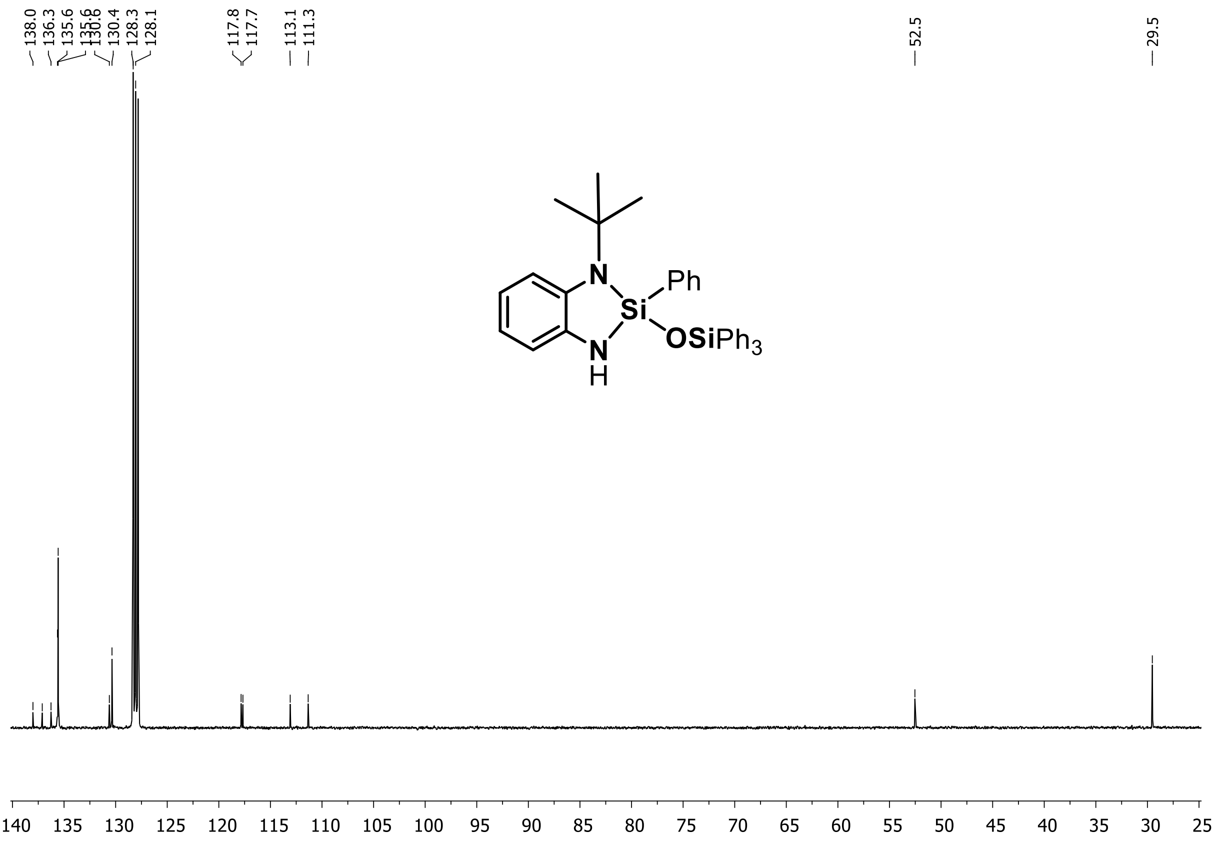

Figure S31. $^{13}C\{^{1}H\}$ NMR spectrum (C_6D_6, 298 K) of **8**.

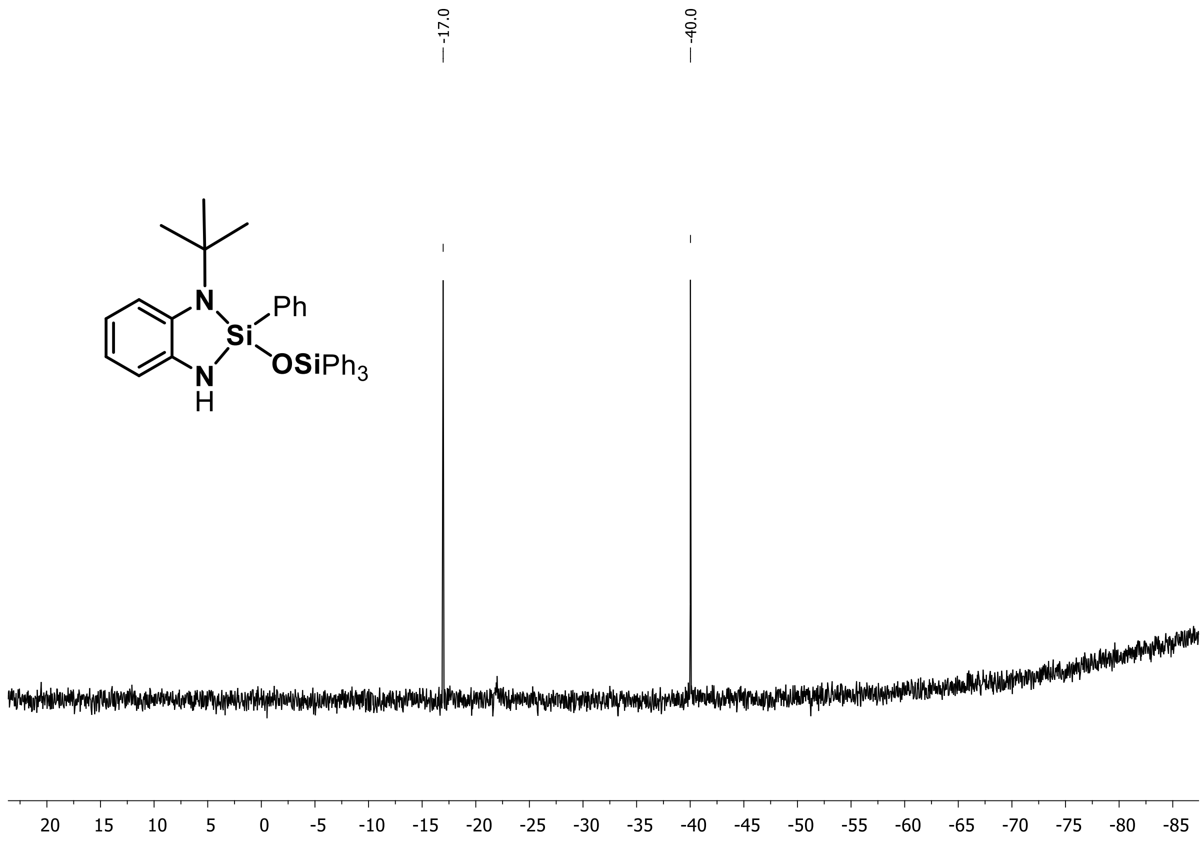

Figure S32. $^{29}Si\{^1H\}$ NMR spectrum (C_6D_6, 298 K) of **8**.

2.8 Synthesis of Triphenylsilanol-*d*

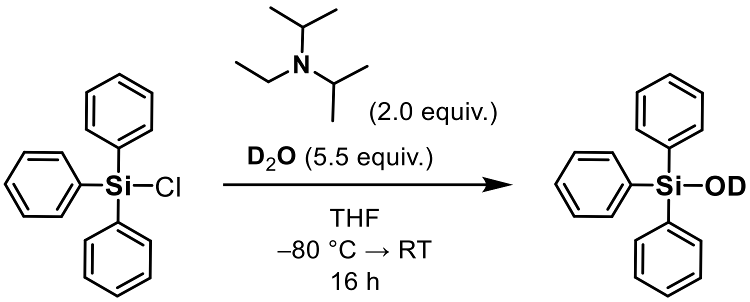

Chlorotriphenylsilane (1.47 g, 5.0 mmol, 1.0 equiv.) was dissolved in THF (15 mL) and cooled to –80 °C. *N*,*N*-Di-*iso*-propylethylamine (1.7 mL, 10.0 mmol, 2.0 equiv.) and D_2O (0.5 mL, 27.5 mmol, 5.5 equiv.) were added subsequently via syringe. The reaction mixture was allowed to warm up to room temperature in 16 hours. Then, all volatiles were removed *in vacuo*. The remaining crude product was extracted with diethyl ether (2 × 3 mL) and the remaining solids filtered off via filter cannula. The combined filtrates were dried thoroughly *in vacuo*. Triphenylsilanol-*d* was obtained as a white powder (946 mg, 3.4 mmol, 68%) with 3% non-deuterated triphenylsilanol as a byproduct.

^{1}H NMR (400 MHz, THF-d_8, 298 K): δ = 7.28–7.38 [m, 6H, H_{Ar}], 7.57–7.67 [m, 9H, H_{Ar}]. **^{2}H NMR** (61 MHz, THF-d_8, 298 K): δ = 6.08 (s, O*D*). **^{13}C{^{1}H} NMR** (101 MHz, THF-d_8, 298 K): δ = 128.5 [s, CH$_{Ar}$], 130.4 [s, CH$_{Ar}$], 135.9 [s, CH$_{Ar}$], 138.0 [s, C_{Ar}].

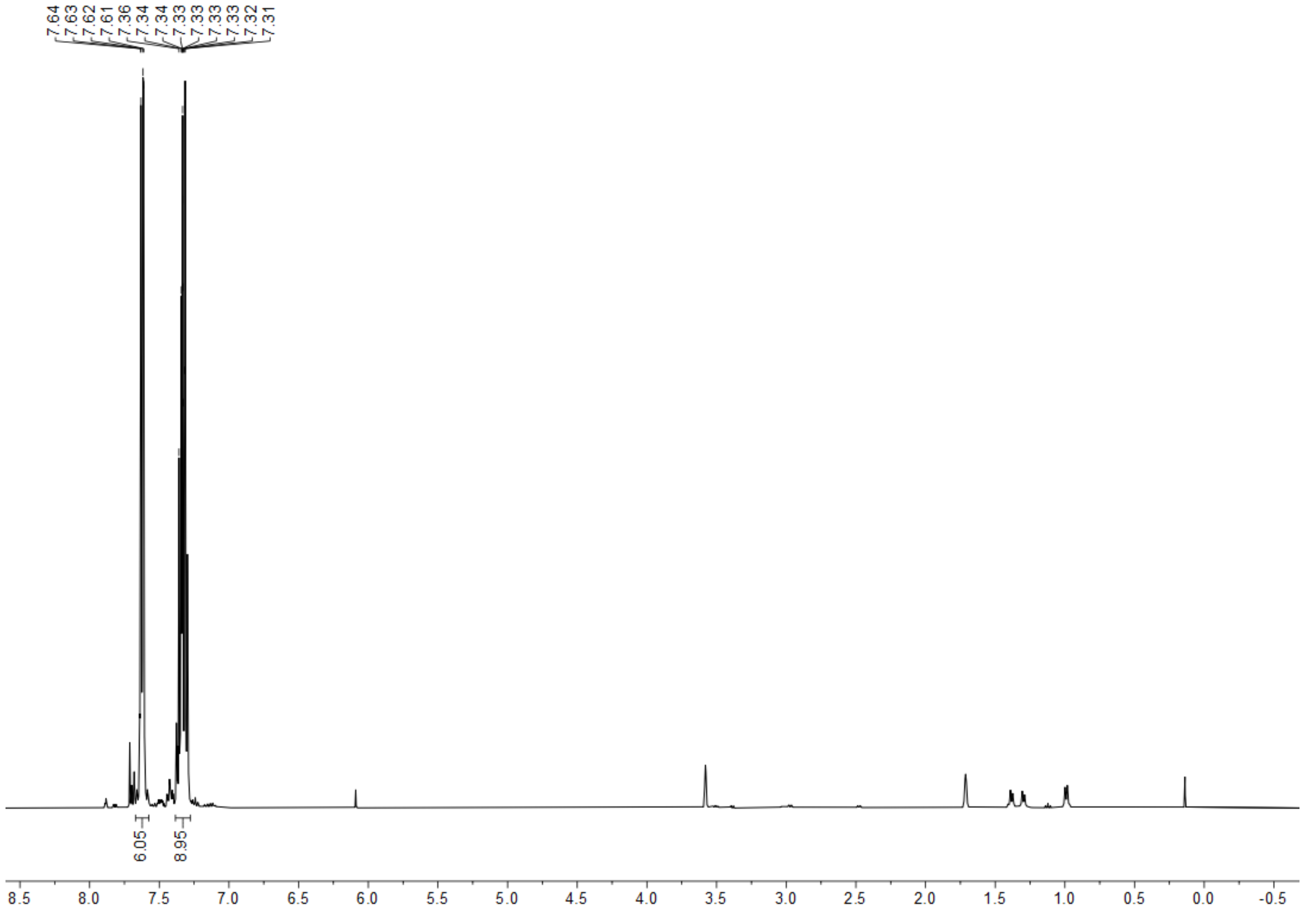

Figure S33. ^{1}H NMR spectrum (THF-d_8, 298 K) of triphenylsilanol-*d*.

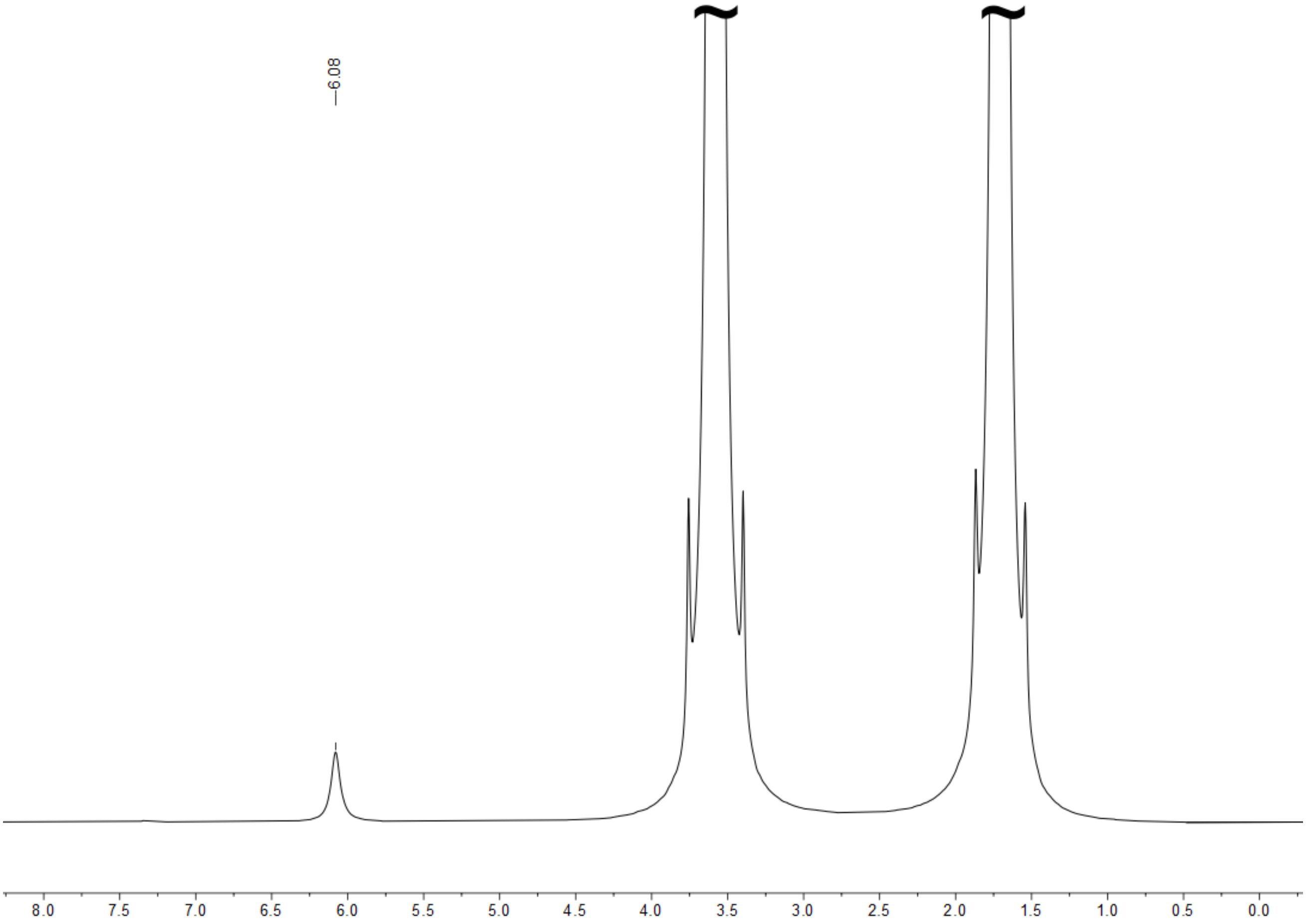

Figure S34. ^{2}H NMR spectrum (THF-d_8, 298 K) of triphenylsilanol-*d*.

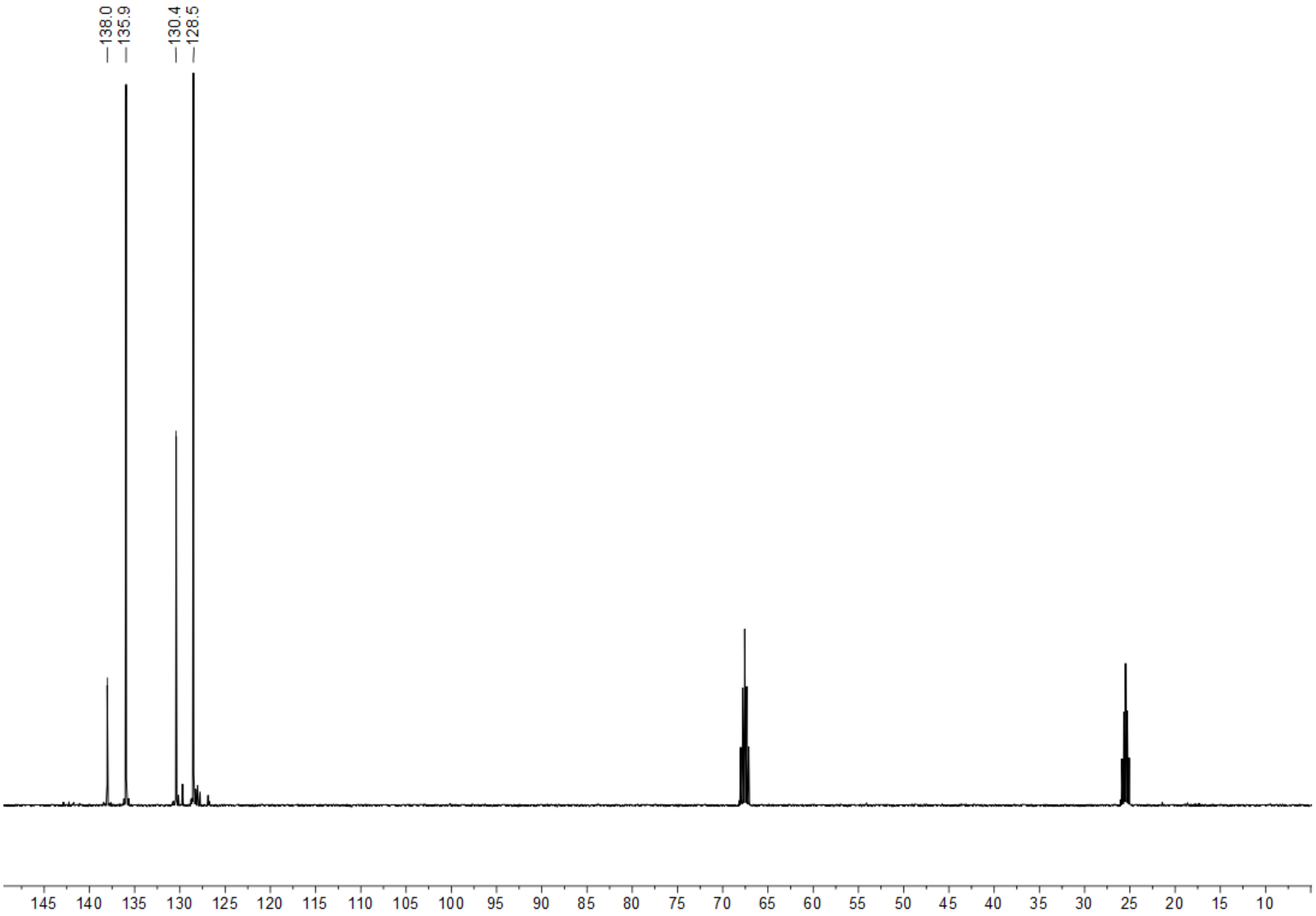

Figure S35. $^{13}C\{^1H\}$ NMR spectrum (THF-d_8, 298 K) of triphenylsilanol-*d*.

2.9 Reaction of Compound 2 with Triphenylsilanol-*d*

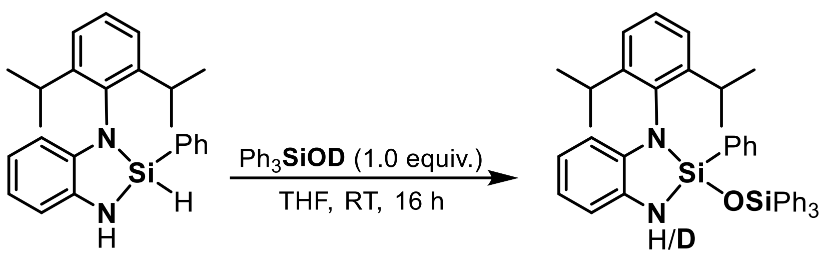

2 **3-*d***

Compound **2** (186 mg, 0.5 mmol, 1.0 equiv.) and triphenylsilanol-*d* (139 mg, 0.5 mmol, 1.0 equiv.) were dissolved in THF (10 mL) and stirred at room temperature for 16 hours. Next, all volatiles were removed *in vacuo*. The residue was washed with *n*-pentane (3 × 2 mL) to afford compound **3-*d*** as a white solid (187 mg, 0.29 mmol, 58%).

^{1}H NMR (400 MHz, C_6D_6, 298 K): δ = 0.31 [d, 3H, $^3J_{H\text{-}H}$ = 6.8 Hz, CH(C*H*)$_3$], 0.91 [d, 3H, $^3J_{H\text{-}H}$ = 6.8 Hz, CH(C*H*)$_3$], 0.93 [d, 3H, $^3J_{H\text{-}H}$ = 6.8 Hz, CH(C*H*)$_3$], 0.99 [d, 3H, $^3J_{H\text{-}H}$ = 6.8 Hz, CH(C*H*)$_3$], 2.83 [sept, 1H, $^3J_{H\text{-}H}$ = 6.8 Hz, C*H*(CH)$_3$], 3.62 [sept, 1H, $^3J_{H\text{-}H}$ = 6.8 Hz, C*H*(CH)$_3$], 3.84 [s, 1H, N*H*], 6.15 [d, 1H, $^3J_{H\text{-}H}$ = 7.6 Hz, CH_{Ar}], 6.47 [dd, 1H, $^3J_{H\text{-}H}$ = 7.5 Hz, $^4J_{H\text{-}H}$ = 1.1 Hz, CH_{Ar}], 6.68 [td, 1H, $^3J_{H\text{-}H}$ = 7.6 Hz, $^4J_{H\text{-}H}$ = 1.3 Hz, CH_{Ar}], 6.80 [td, 1H, $^3J_{H\text{-}H}$ = 7.5 Hz, $^4J_{H\text{-}H}$ = 1.2 Hz, CH_{Ar}], 7.00–7.13 [m, 13H, CH_{Ar}], 7.17–7.21 [m, 2H, CH_{Ar}], 7.61–7.65 [m, 6H, CH_{Ar}], 7.69–7.72 [m, 2H, CH_{Ar}]. **^{2}H NMR** (61 MHz, C_6D_6, 298 K): δ = 3.84 (br, N*D*).

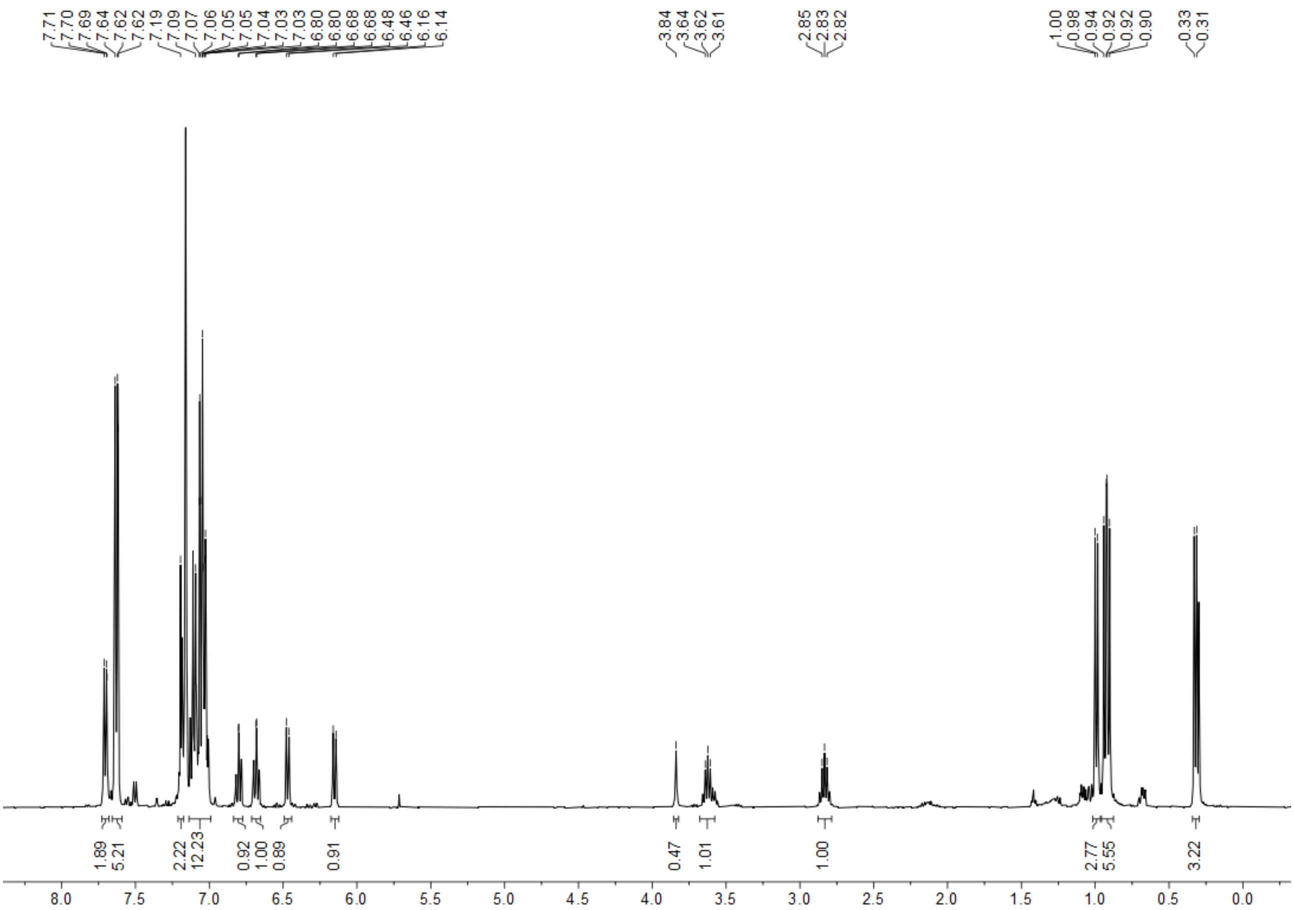

Figure S36. ^{1}H NMR spectrum (C_6D_6, 298 K) of **3-*d***. The N*H* signal at 3.84 ppm indicates a deuteration of 53%.

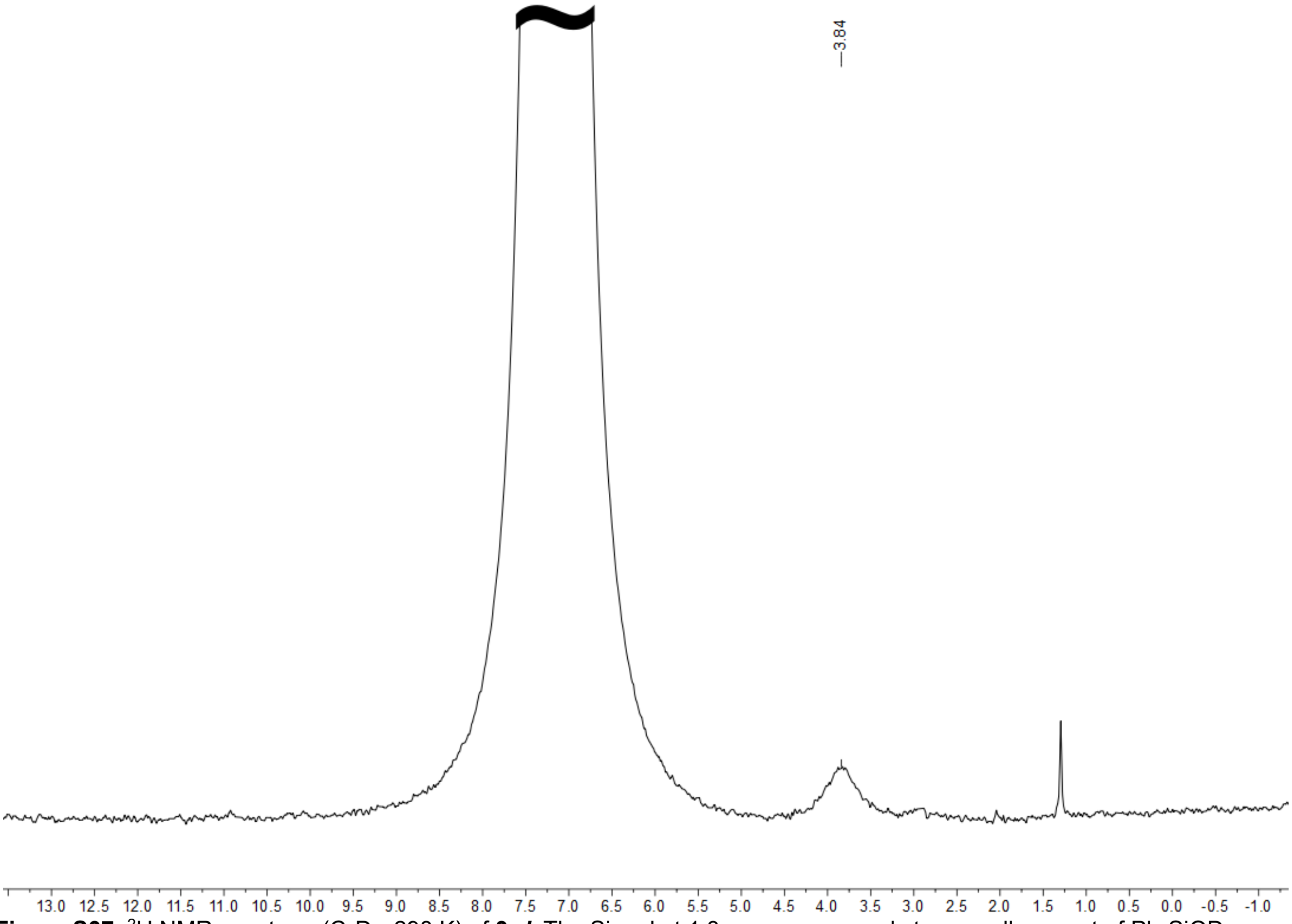

Figure S37. ^{2}H NMR spectrum (C_6D_6, 298 K) of **3-*d***. The Signal at 1.3 ppm corresponds to a small amount of Ph_3SiOD.

3. Mechanistic Investigations via NMR Spectroscopy

3.1. General Considerations

NMR spectra in this chapter were recorded on 600 MHz Bruker spectrometers (Avance III HD 600 MHz or Avance Neo 600 MHz) equipped with either an inverse ^{19}F-selective 5 mm TBI-F (^{1}H, ^{19}F, X) probe with z-gradient or an observe 5 mm Prodigy BBO (X, ^{1}H) probe with z-gradient. Temperatures were controlled with Bruker BVTE 3000 or Bruker BVTE 3900 temperature control units. Temperatures below 233 K were achieved via liquid N_2 evaporation. ^{1}H NMR spectra were referenced to the residual ^{1}H solvent signal of THF-d_8 (δ_{1H} = 1.74 ppm). ^{29}Si spectra were externally referenced to TMS (tetramethylsilane, δ_{29Si} = 0.0 ppm). ^{29}Si NMR spectra in figures were acquired without ^{1}H decoupling. Spectra were processed and analyzed with Bruker TopSpin v4.2.0, MestRe MNova v14.3.2 and Python scripts. Figures were created using Perkin Elmer ChemDraw Professional 2022, CorelDraw v24.0.0.301, MestRe MNova v14.3.2 and Python 3 scripts. Multipoint Baseline Correction (Smooth Segments Algorithm) was carried out for 1D ^{29}Si NMR spectra to remove the broad residual glass peak and obtain integratable data (an uncorrected spectrum is shown in Figure S34, top).

3.2. NMR-Measurement at 180 K

A sample of **2** in dry THF-d_8 was prepared in a medium-walled 5 mm J. Young NMR-tube under N_2 atmosphere and cooled to –80 °C using a cooling bath (*iso*-propanol / liquid nitrogen). Ph_3SiOH (5 equiv.) was added, the tube quickly shaken and transferred to a 600 MHz Bruker Avance III HD spectrometer with TBI-F probe, pre-cooled to 180 K via a liquid N_2 evaporator. After allowing the sample to thermally equilibrate, shimming was carried out manually and automatically (topshim), but sharp signals could not be obtained due to the high viscosity of THF-d_8 at this temperature. No starting material **2** could be detected in the ^{29}Si NMR spectra, indicating full conversion to **2a** / **2b**, whereas some of the excess Ph_3SiOH was still present based on the ^{1}H NMR (Figure S38) and ^{29}Si NMR (Figure S39). With the obtained data, it is not possible to determine how much free Ph_3SiOH is still present as there is significant signal overlap in the ^{1}H NMR and low signal-to-noise ratio in the ^{29}Si NMR-spectra.

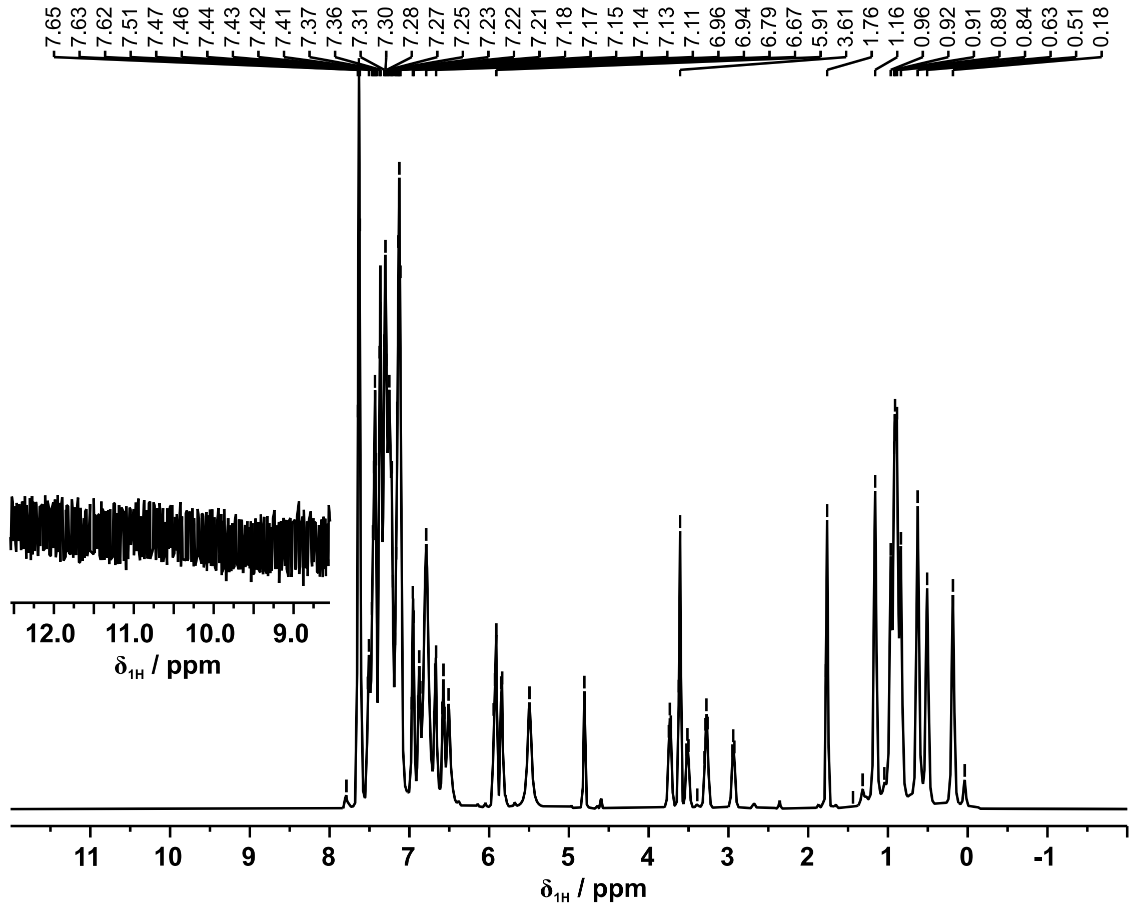

Figure S38. ^{1}H NMR spectrum (THF-d_8, 180 K, 600 MHz, 16 scans, exponential apodization of 2 Hz) of **2** and Ph_3SiOH (5 equiv.). All signals are broadened and singlets have a FWHM of approximately 15 Hz due to the high viscosity of THF at the low temperature. The region between 12.5 and 8.5 ppm is expanded to clarify the absence of sharp hydrogen bonded species under these conditions.

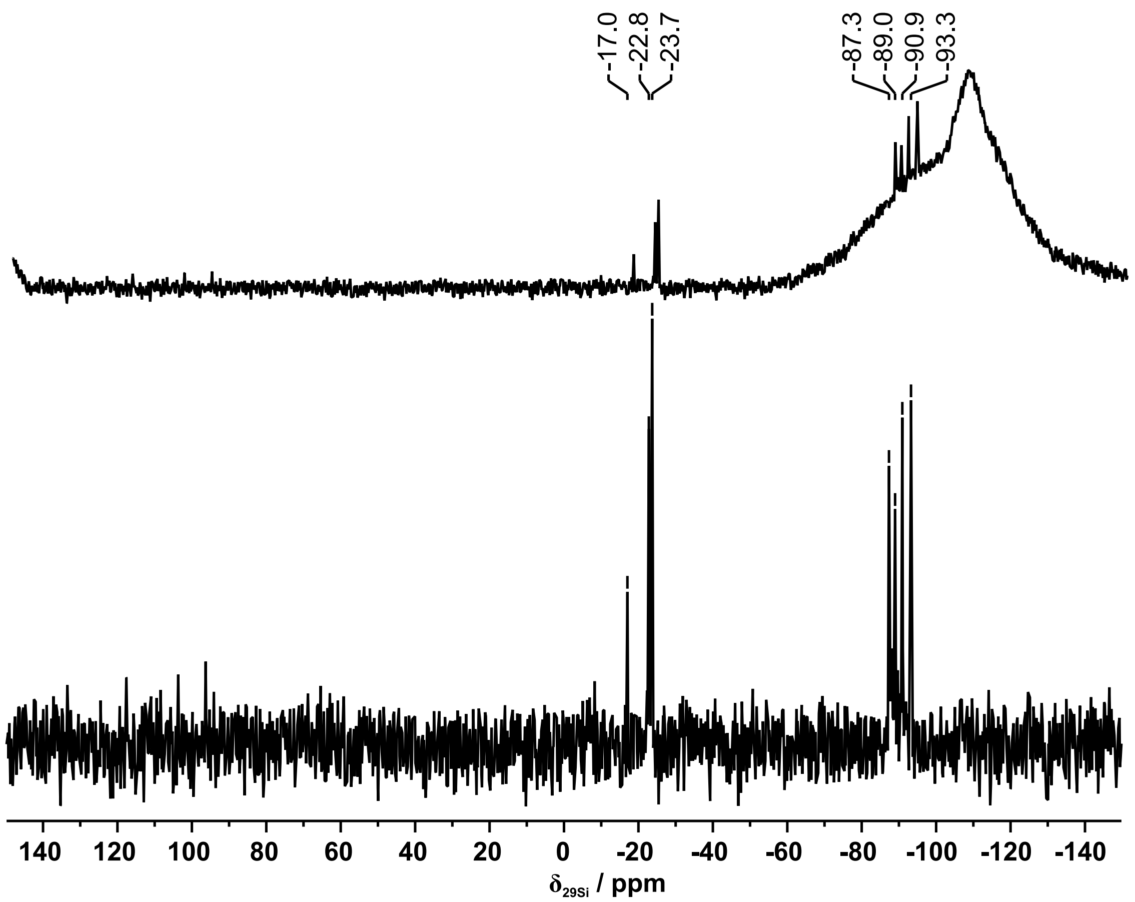

Figure S39. ^{29}Si NMR spectrum (THF-*d*$_8$, 180 K, 119.2 MHz, 256 scans, exponential apodization of 5 Hz) of **2** and Ph_3SiOH (5 equiv.). All signals are broadened and singlets have a FWHM of approximately 40 Hz due to the high viscosity of THF at the low temperature. The top spectrum is the originally measured spectrum without baseline correction, showing the large background signal originating from ^{29}Si in the NMR tube. The spectrum on the bottom was processed with advanced baseline correction to flatten the broad silicate background signal (see general considerations).

Since no changes of sample composition were noticed during several hours of keeping the sample at 180 K in the spectrometer, we rationalized that the nucleophilic addition of Ph_3SiOH to **2** had occurred fast in the short time (one minute) between mixing the reagents at room temperature and placing the sample into the cooling bath, thus "freezing" the chemical exchange equilibrium between **2a** and **2b**. From the observed ratio **2a**:**2b** of circa 1:1 and the absence of starting material, it can be concluded that the relative Gibbs energies ΔG of **2a** and **2b** are almost identical. Likewise, the reaction of **2** with Ph_3SiOH would be at least slightly exothermic (> 2 kJ mol^{-1}) if it is assumed that the formation is reversible (*vide infra* at room temperature). From a simple Boltzmann distribution model, a higher energy of 4.2 kJ mol^{-1} (1 kcal mol^{-1}) of the starting materials relative to **2a** and **2b** would result in only about 3% remaining **2** at 180 K, thus having a concentration below the detection limit under the applied measurement conditions.

As we argue in the main manuscript, the data can be very well explained by the presence of two anionic pentacoordinate silicates from the nucleophilic addition of silanolate Ph_3SiO^- to **2**, whereas **2a** is the initially formed isomer with axial N–Dipp and Ph_3SiO, and since attack anti to the N–Dipp group appears to follow the least sterically hindered pathway. Any formation of a pentacoordinate silicate species from addition of a nucleophile to the neutral, tetravalent silicon species is joined by a rehybridization from sp^3 to sp^2 on silicon. In this model, the equatorial substituent R–Si σ-bonds stem from one sp^2 orbital on Si with R each, while the axial Nu–Si–R is rather a 2e-3c-bond where Nu and R share the remaining unhybridized p orbital on Si with a lower bond order (0.5 in the symmetric case). A more thorough analysis applying MO-theory[111] to the related symmetric PF_5 molecule arrives at a formal

bond order of 0.7 for axial and $^5/_6$ for equatorial bonds. This reduction of bond order for both kinds of substituents is reflected by a stronger polarization of the bonds, which explains the downfield ^{1}H shifts of **2a** (5.91 ppm) and **2b** (4.81 ppm) relative to **2** (6.14 ppm). Since the weakening is less present on the equatorial positions, the ^{1}H NMR signal of **2a** is also less shifted. In contrast, the size of the coupling constant $^1J_{SiH}$ is strongly dependent on the amount of s-character of a bond (mainly Fermi contact contribution). As discussed above, the Si–H bond in **2** ($^1J_{SiH}$ = 233 Hz) is formed from a sp^3-orbital of silicon and the s-orbital of H with formal 25% s-contribution from silicon. The s-contribution is raised to 33% in **2a** ($^1J_{SiH}$ = 280 Hz) from a sp^2-Si orbital, while it is lowered to formally 0% in **2b** ($^1J_{SiH}$ = 197 Hz). A simple empirical approximation of the correlation of $^1J_{HSi}$ with the Si-s-contribution may be given with

$$^1J_{SiH} = \frac{(1 + s_{Si})}{2} \cdot 400\ Hz$$

whereas the number 1 originates from the 100% s-character of the H s-orbital in the bond and s_{Si} is the s-contribution from Si (p: 0; sp^3: $^1/_4$; sp^2: $^1/_3$; sp: $^1/_2$).

3.3. VT-Measurements from 180 K to 263 K: Berry Pseudorotation vs. Dissociative-Associative Mechanism

After being kept at 180 K for few hours, the sample from 3.2 was initially warmed to 193 K and then in steps of 10 K up to 263 K. ^{1}H NMR spectra were acquired at each temperature. The measurement of time-consuming ^{29}Si NMR spectra was omitted at the intermediate temperatures, since we wanted to observe the reaction in an early stage.

From the VT-measurement, a clear indication for chemical exchange being present between the two intermediates **2a** and **2b** (see main manuscript Figure 6 and Figure S40). At 180 K, the ^{1}H NMR signals **2a** and **2b** appeared already broad (the apparently sharper signals at 193 K can be attributed to the high viscosity of THF at 180 K), and could not be detected anymore at 213 K. At further increased temperatures, a new signal (**2ab**) appeared which was very broad at 223 K, then became narrower when approaching 263 K.

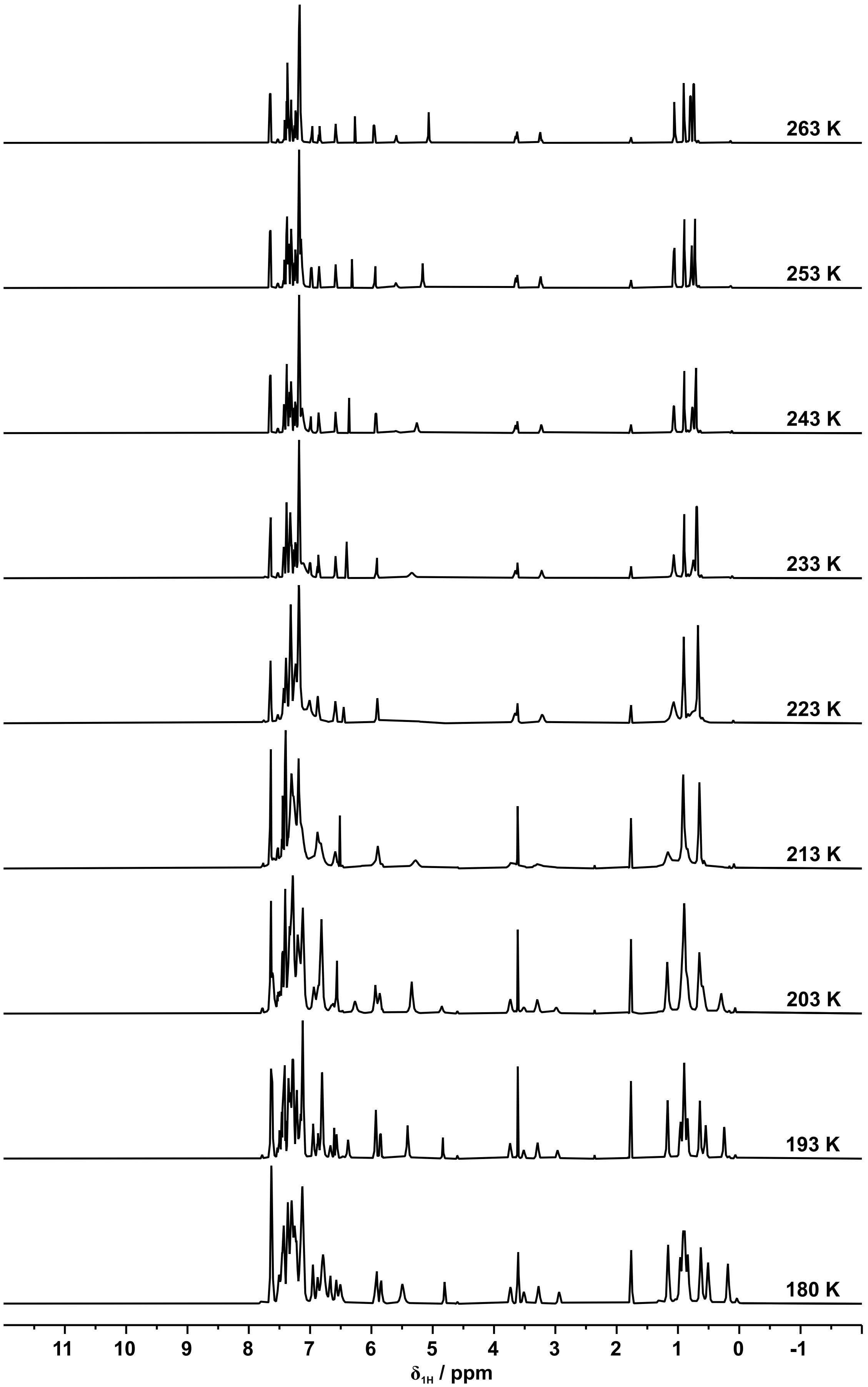

Figure S40. Full variable temperature ^{1}H NMR spectra (THF-d_8, 180–263 K) of the reaction of **2** with Ph_3SiOH (5 equiv.). For discussion refer to the main manuscript.

At first sight, there are two different mechanistic pathways, which could cause this exchange.[112] First would be the transformation of **2a** to **2b** and vice versa via a Berry pseudorotation (BPR) in which the phenyl substituent would remain as the equatorial, stationary ligand. In such a two-state system, the activation barrier $\Delta G^{\ddagger}_{BPR}$ can be determinined from the coalescence temperature T_c and the difference of Larmor frequencies Δv of both exchanging partners in the slow exchange regime. The rate at the coalescence temperature T_C for two (presumably) degenerate ground states is then approximately

$$k_{obs} \approx \frac{\pi\Delta\nu}{\sqrt{2}}$$

with a measured T_C = (223 ± 10) K and Δv = 654 Hz regime, $\Delta G^{\ddagger}$ was calculated via Eyring-Polanyi according to equation

$$\Delta G^{\ddagger} = RT_C \ln\left(\frac{k_B T_C}{h k_{obs}}\right)$$

to yield a value of $\Delta G^{\ddagger}_{BPR}$ = (41 ± 2) kJ mol^{-1} or approximately 10 kcal mol^{-1}, consistent with a fast process at room temperature. This value is similar to the reported values for compounds in which the presence of BPR as the only isomerization mechanism has been confirmed.[112]

On the other hand, BPR may not be the only pathway to obtain **2b**, at least at higher temperatures. Since we observed a slow exchange of starting material **2** and Ph_3SiOH with **2ab** at 298 K (see Section 3.4), it cannot be ruled out that **2b** may be directly formed from the starting materials from a nucleophilic attack opposite to the NH-nitrogen in **2**, although with a presumably higher activation barrier than the analogous formation of **2a**. Useful results from *in silico* studies have been hampered by the size and flexibility of the system under investigation.

3.4. Reaction monitoring at 298 K

In a separate sample with **2** and 5 eq. Ph_3SiOH in THF-d_8, it was possible to observe transient ^{1}H and ^{29}Si NMR signals of presumed pentacoordinate mixtures (**2ab**) at room temperature. The sample was warmed by hand and intensively shaken for five minutes prior to insertion to the magnet, thus a considerable amount of product was already present at t = 0 min, which marks the timepoint of the first spectrum that was measured. The sample was monitored by ^{1}H and ^{29}Si NMR spectroscopy for 12 hours.

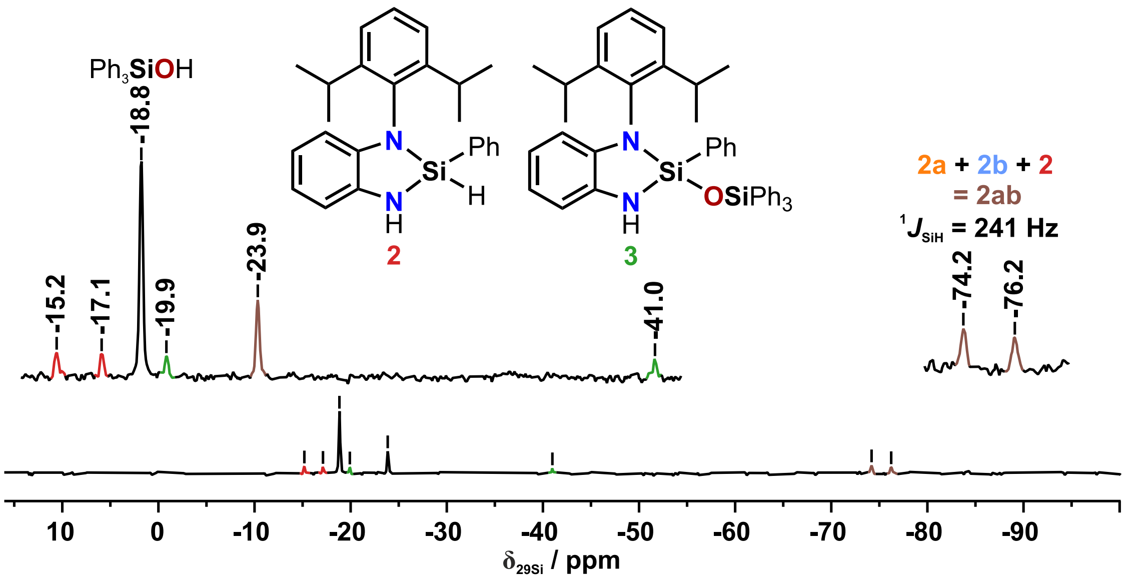

Figure S41. ^{29}Si NMR spectrum (THF-d_8, 180–298 K in 80 minutes) of the reaction of **2** with Ph_3SiOH (1 equiv.). The doublet around –75 ppm is proposed to originate from fast exchange of **2a** and **2b**, while **2** must also participate as exchanging partner based on the chemical shift (see below). For the sake of readability, this mix of isomers is defined as **2ab**.

In the first ^{1}H NMR spectrum, sharp signals corresponding to the starting material, intermediates **2ab**, and the product in a ratio of 1 : 3 : 1.3 were observed (Figure S41). Likewise, only a single set of signals was observed for a mixture of intermediates **2a** and **2b** at fast exchange (**2ab**) in the ^{29}Si NMR spectrum, in the presence of starting material and product (Figure S41).

Extended monitoring of the sample revealed that the time dependent concentrations of **2**, **2ab**, and **3** each follow a monoexponential function, resulting in full conversion to the final product **3** after 12 hours (for kinetics, see main manuscript Figure 7, and full ^{1}H NMR spectra in Figure S42). With an almost similar ratio of 1 : 3 for **2** and **2ab**. It can be inferred that the constant ratio of both species would originate from reversible exchange between **2** and **2ab** which is slow on the ^{1}H NMR time scale (two sets of sharp signals), but still much faster than the following irreversible elimination of H_2. From the integral ratios, the relative Gibbs energy Δ*G* of **2** vs. **2ab** was determined to be +2.7 kJ mol^{-1} (according to a Boltzmann distribution model). The sum over the integrals of **2**, **2ab**, and **3** remained constant over the full monitoring and no other intermediates could be observed in the spectra. Since Ph_3SiOH was used in large excess, a pseudo-first order reaction model was assumed. Since Ph_3SiOH was used in large excess, a pseudo-first order reaction model was assumed and a pseudo first order rate constant k_{obs} = (101 ± 4) · 10^{-6} s^{-1} was obtained under these assumptions.

Using the Eyring-Polanyi-Equation

$$\Delta G^{\ddagger} = RT_C \ln\left(\frac{k_B T_C}{h k_{obs}}\right)$$

a value of (95.8 ± 0.1) kJ mol^{-1} could be determined for the activation barrier Δ$G^{\ddagger}$ of dihydrogen elimination. Thus, the irreversible elimination renders the rate determining step in this reaction, as was also observed previously by Eaborn and Jenkins.[71]

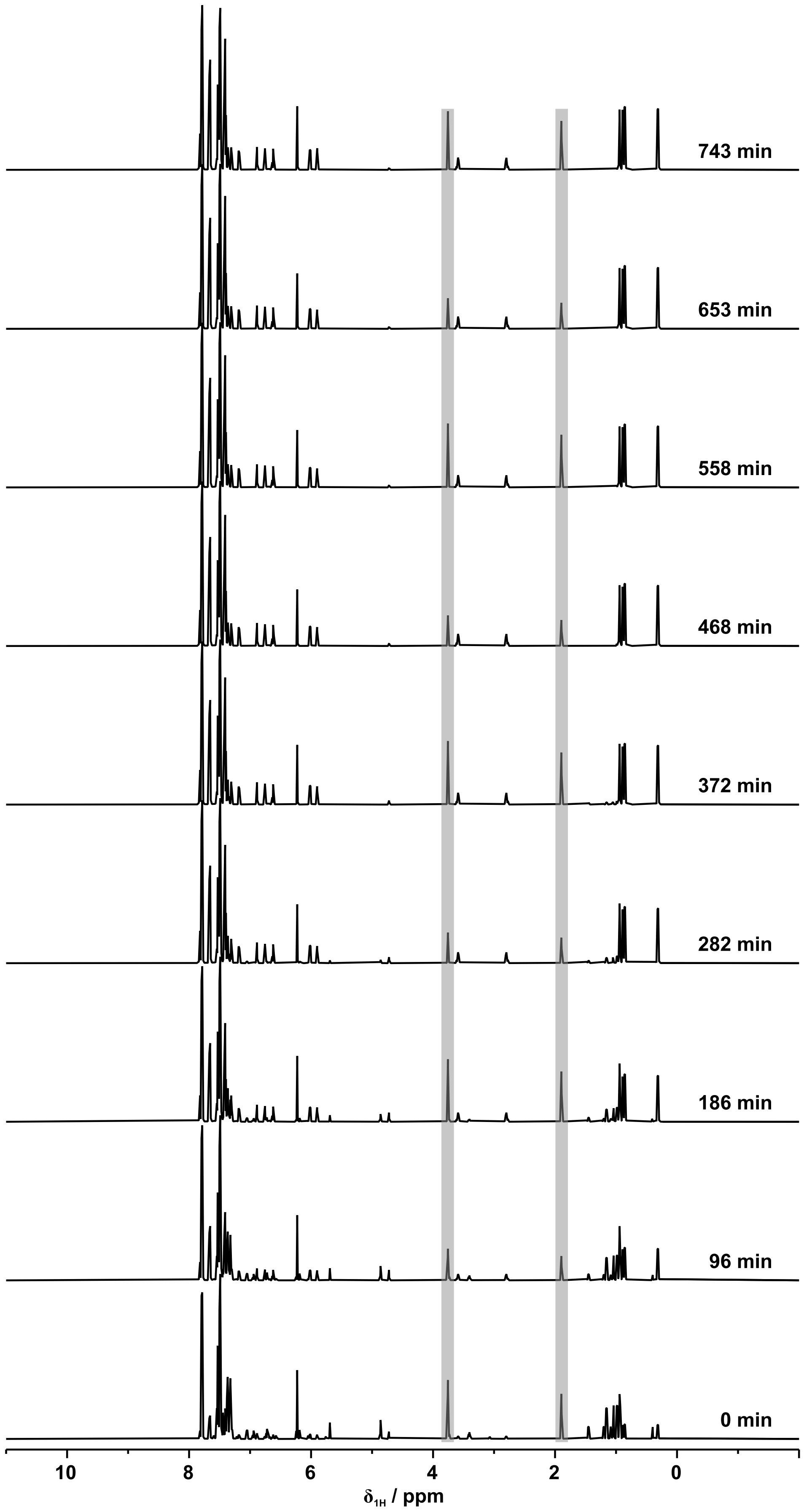

Figure S42. Full ^{1}H NMR spectra of the reaction monitoring (THF-*d*$_8$, 298 K) of the reaction of **2** with Ph_3SiOH (5 equiv.). Further discussion in the main manuscript. The inconsistent intensity of the solvent signals (THF, shaded in grey) is owed to the acquisition of ^{29}Si NMR spectra either without decoupling or inverse-gated ^{1}H decoupling.

3.5. Measurements at 233 K and 298 K Under Highly Basic Conditions

In order to gain more information on the charge of **2a** and **2b**, it was rationalized that they may be stabilized under basic conditions, since we propose an initial attack of the deprotonated silanol and negative charged intermediates. Observation of the same NMR signals would indicate a correct structural assignment.

Thus, an NMR sample containing **2** in THF-d_8 was prepared under N_2 atmosphere and cooled to –80 °C. Ph_3SiOK (2.5 equiv.) in THF-d_8 was added via cannula. It was slightly effervescent, indicating hydrogen evolution either by deprotonation of N-H in **2** by KH which could have been present as impurity from the preparation of Ph_3SiOK or formation of **3** from **2**. The sample was inserted into the NMR spectrometer, precooled to 233 K.

A magnitude-mode ^{1}H-^{29}Si-HMQC (Figure S43) indeed revealed the presence of two species with similar chemical shifts and $^{1}J_{1H\text{-}29Si}$ coupling constants as the ones previously found for **2a** and **2b** under neutral conditions (since the spectrum was not measured as phase sensitive, accurate values for ^{1}J could not be obtained, see also main manuscript). Further detected signals could be assigned to the starting material **2** and Ph_3SiOH / Ph_3SiOK. In contrast to the results described under 3.3, the two species could be clearly distinguished at 233 K, indicating a more hindered chemical exchange via Berry pseudorotation of both intermediates under these conditions.

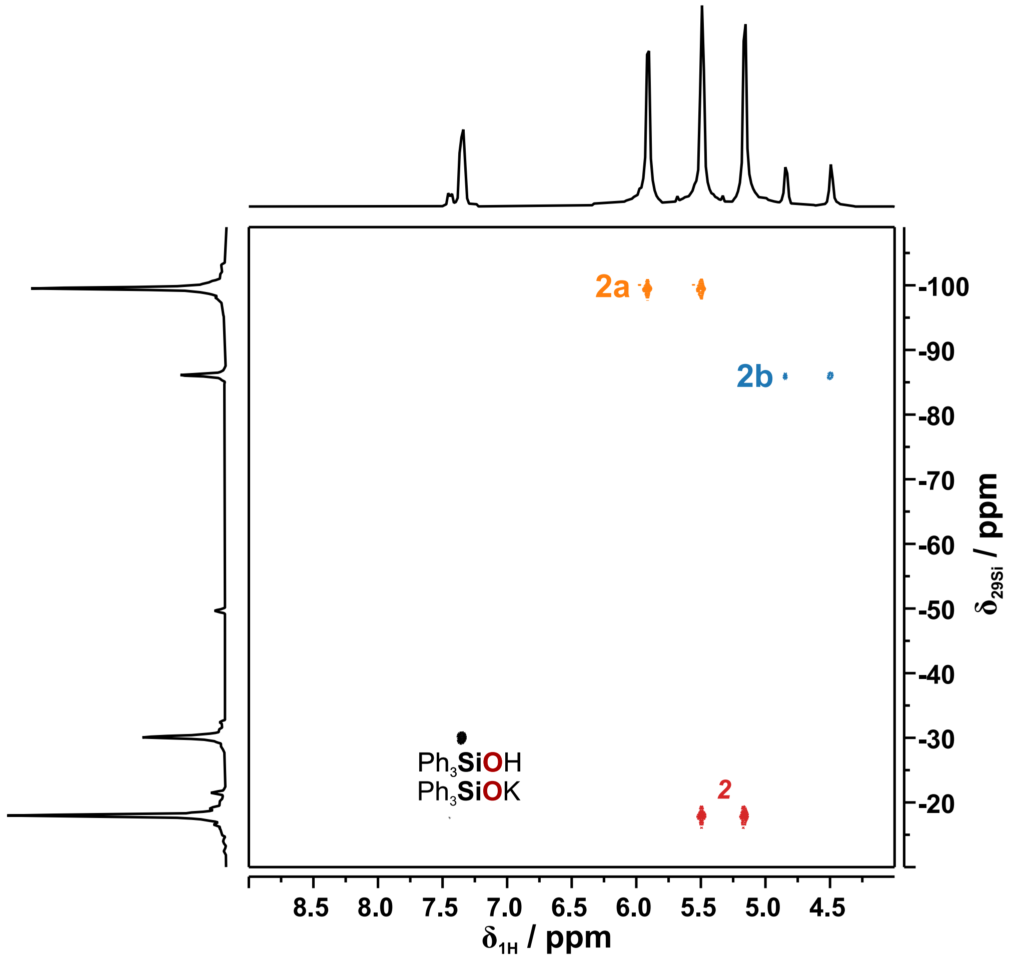

Figure S43. ^{1}H-^{29}Si HMQC (pulse sequence: hmqcgpqf, delay τ optimized for ^{1}J of 233 Hz) at 233 K of a sample of **2** with 2.5 eq. Ph_3SiOK in THF-d_8. Both species **2a** and **2b** could be detected.

The BPR barrier may be influenced by slight changes of the coordination sphere around the central Si in **2a/2b**, as is indicated by the presence of a multitude of new signals at around 10 ppm in the 1D-^{1}H NMR spectrum (Figure S 44). These new signals may originate from weak hydrogen bonds from a multitude of possible small

aggregates via e.g. O···H–N, O···H–O, N–H···N or even Si–H···O bonding motifs and hamper the isomerization process from **2a** to **2b** and vice versa. Likewise, they may also influence the absolute energy differences of **2a** and **2b**, which would explain the difference in observed ratios than under neutral conditions.

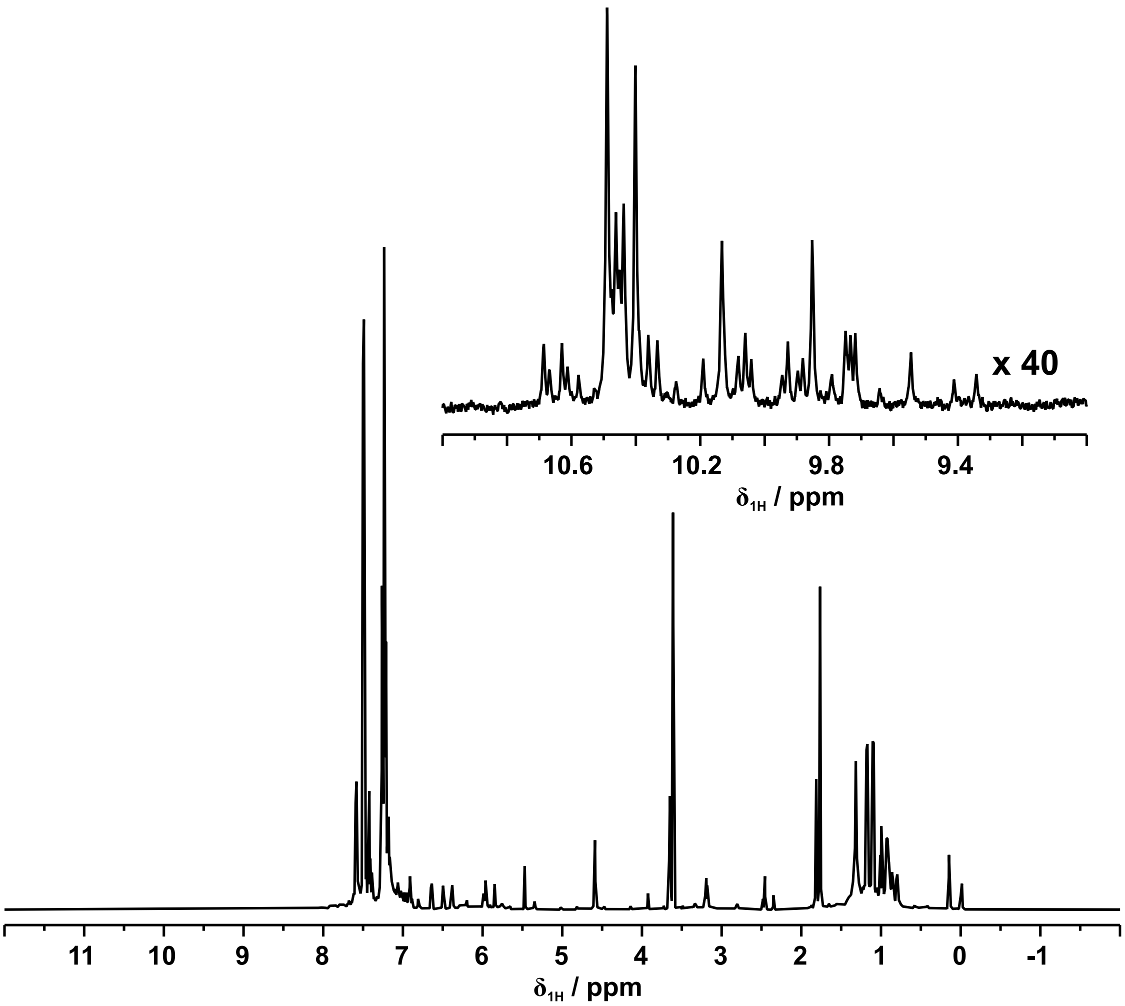

Figure S44. ^{1}H NMR spectrum of a sample of **2** with 2.5 eq. Ph_3SiOK in THF-d_8 at 233 K, showing many signals indicating the presence of a multitude of H-bonds or aggregated species at low temperature. Peak labels have been omitted for clarity.

When warming the sample to room temperature, the signal for **2a** completely vanished and only **2b** and the starting materials were detected in a ^{1}H-^{29}Si HMQC (Figure S45), overall indicating that under these conditions **2a** is the kinetically faster formed intermediate, while equilibration to **2b** would be thermodynamically driven.

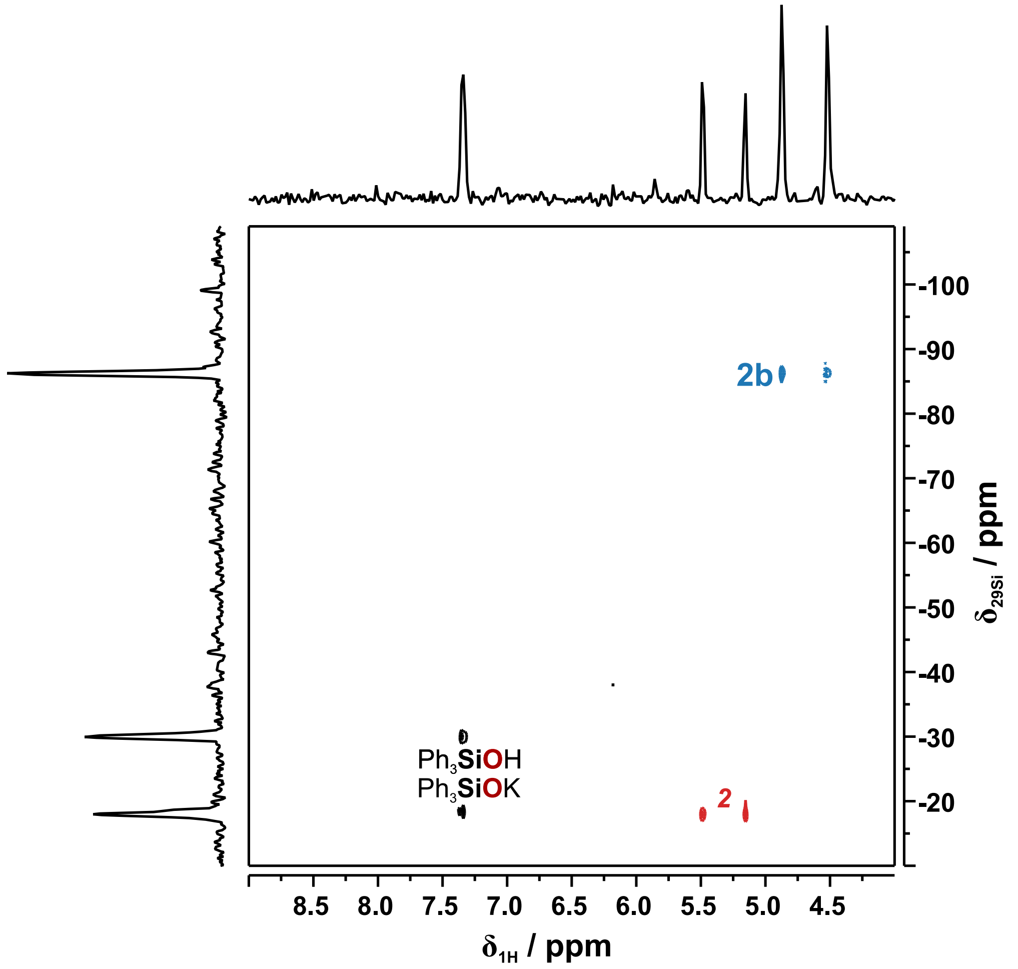

Figure S45. ^{1}H-^{29}Si HMQC (pulse sequence: hmqcgpqf, delay τ optimized for ^{1}J of 233 Hz) at 233 K of a sample of **2** with 2.5 eq. Ph_3SiOK in THF-*d*8 directly after being warmed from 233 K. Only the doublet assigned to **2b** could be detected at room temperature as intermediate.

The signals for **2** and **2b** had also vanished in another ^{1}H-^{29}Si HMQC, recorded just 4 hours later, with only the signals for Ph_3SiOH and silanolate observable (Figure S46). The ^{1}H NMR spectrum of this sample did not resemble the spectrum of the product **3** at the end of reaction monitoring under neutral conditions (Figure S47). Instead, a quite complex reaction mixture is observed which we assume to originate from many imaginable side reactions in this strongly basic environment.

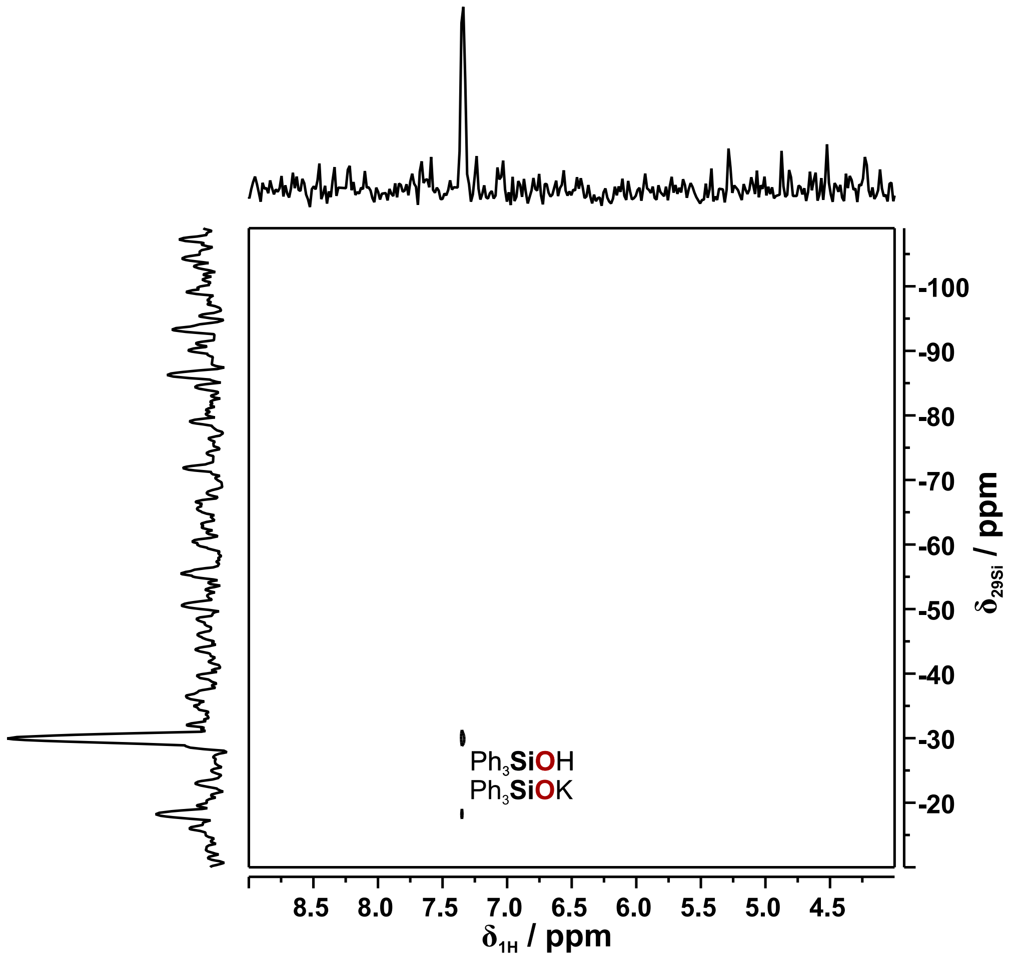

Figure S46. ^{1}H-^{29}Si HMQC (pulse sequence: hmqcgpqf, delay τ optimized for ^{1}J of 233 Hz) at 298 K of a sample of **2** with 2.5 eq. Ph_3SiOK in THF-d_8 four hours after being warmed from 233 K. No intermediates could be observed anymore, which may indicate degradation or precipitation under these conditions.

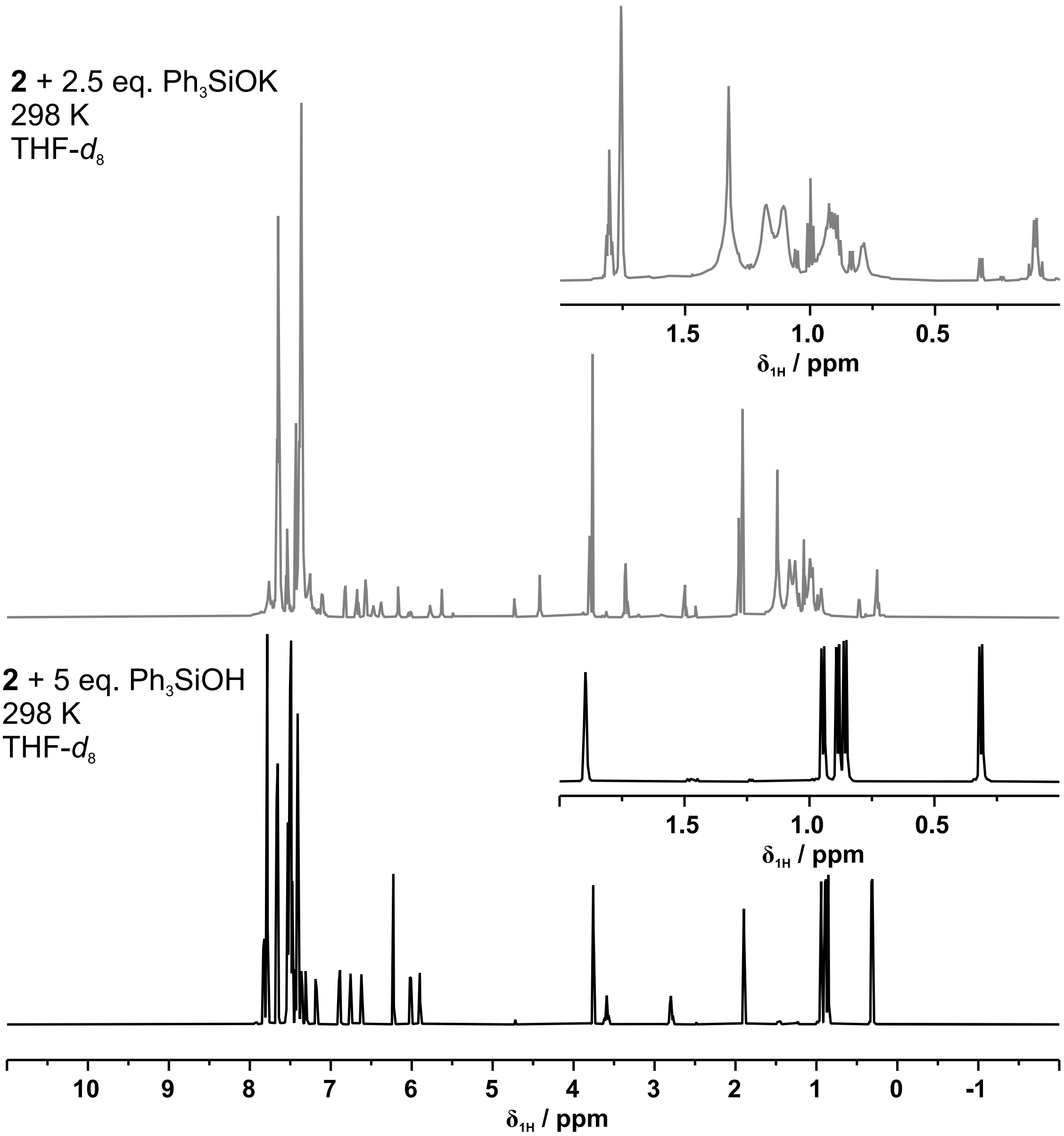

Figure S47. ^{1}H NMR spectra (THF-d_8, 298 K) at the end of reaction monitoring at 298 K (bottom, black) and ^{1}H NMR spectrum under basic conditions after 4 hours at room temperature (top, dark grey) with extensions of the methyl region from 0 to 2 ppm, indicating a very different and complex reaction outcome depending on reaction conditions. Signal labels have been omitted for clarity.

3.6. Measurements of 2 with Triphenylsilanol-*d*

In order to gain more knowledge about dynamic processes a separate sample with **2** and excess Ph_3SiOD in THF-d_8 was prepared in a medium-walled 5 mm J. Young NMR-tube under N_2 atmosphere and subjected to NMR-spectroscopic measurement.

Since only a very slow reaction was observed initially, the sample was stored at room temperature for 4 days before being measured again. At this point, the ^1H NMR spectrum resembled mostly the spectrum of the product **3** from earlier experiments with Ph_3SiOH (Figure S48). Interestingly, although the deuterated silanol was employed in this sample, H_2 was still the major detected isotopomer, followed by HD, with an integral ratio of approximately 80:20. D_2 was not detected by ^2H NMR.

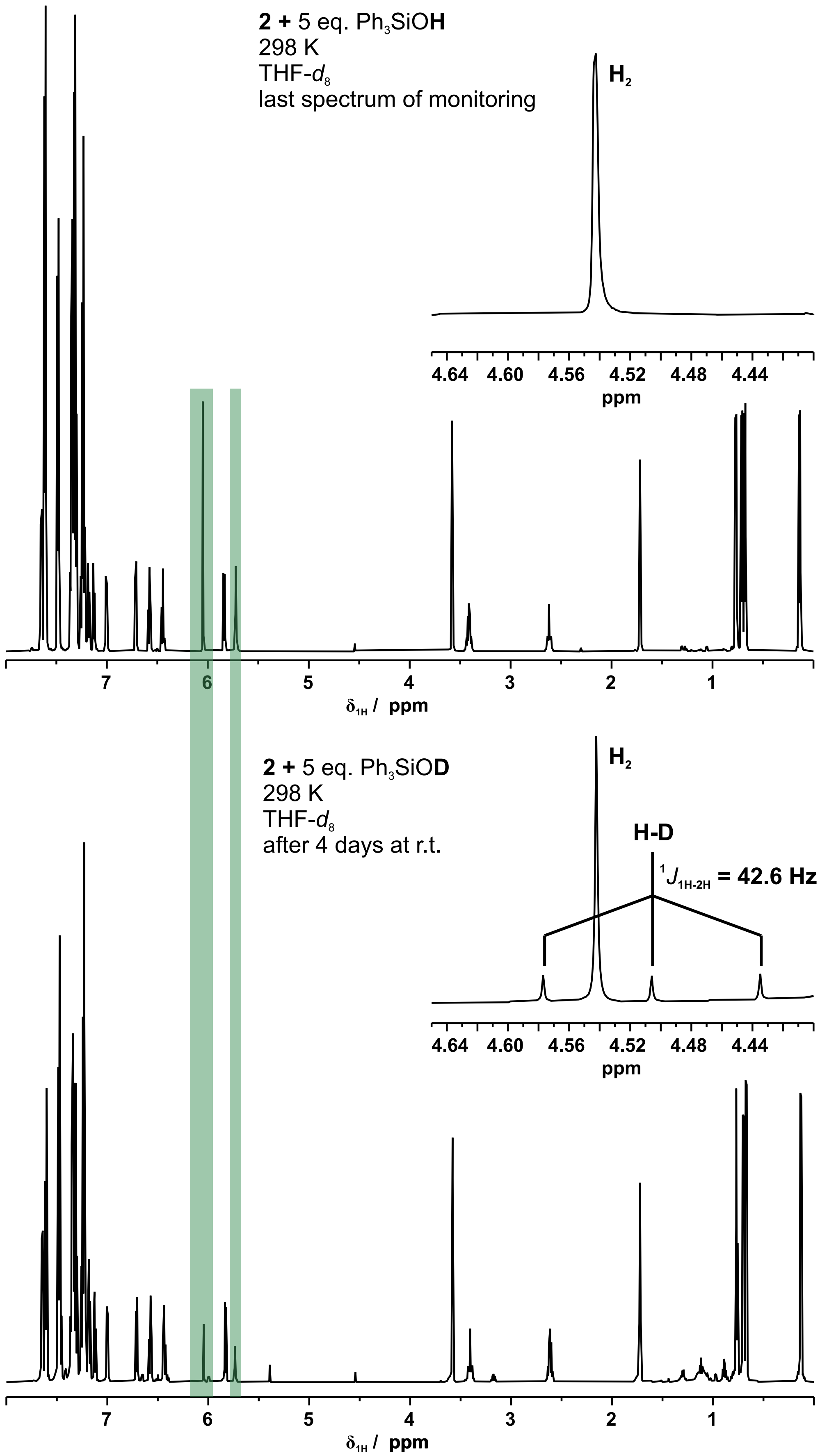

Figure S48. Reaction mixtures of **2** with excess of either Ph_3SiOH (top) or Ph_3SiOD (bottom). The areas marked in green indicate the N–H of **3** and O–H of Ph_3SiOH, which have a much-reduced integral in the experiment with triphenylsilanol-*d*, indicating the presence of deuterium exchange from Ph_3SiOD to ND.

This finding together with the experiment of Section 2.9 indicates the presence of a rapid exchange process between the NH group of **2** and Ph_3SiOD in solution.

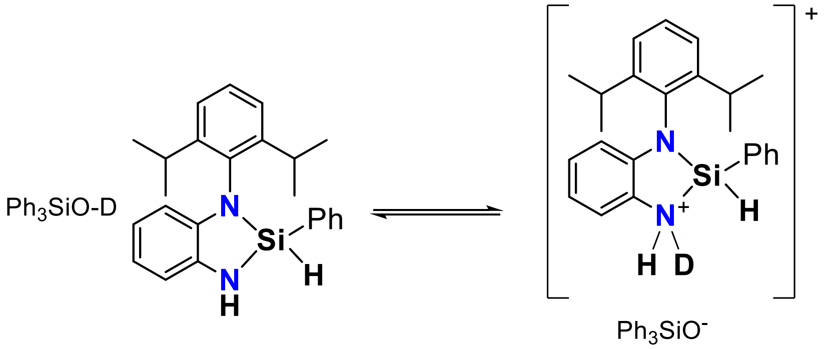

Scheme S1. Possible NH/OD exchange mechanism via a deuterated amine moiety.

3.7. Measurements of 5 with Ph_3SiOH and Et_3N

A sample of **5** in dry THF-*d*8 was prepared in a medium-walled 5 mm J. Young NMR-tube under N_2 atmosphere and cooled to –80 °C using a cooling bath (*iso*-propanol / liquid nitrogen). An excess of Ph_3SiOH and Et_3N was added and the tube quickly shaken and transferred to a 600 MHz Bruker Avance III HD spectrometer with TBI-F probe, pre-cooled to 180 K via a liquid N_2 evaporator. As in section 3.5, the role of the added base is to enhance the reactivity of the silanol and prevent the formation of protonated intermediates. No intermediates as in the case for the Dipp-substituted silane **2** discussed above were observed (Figure S49). Instead, full conversion to the ring opened compound **7** was found. This reveals a much higher lability of the N(Dipp)–Si bond in **5** and that pentacoordinate intermediates, if they are present at all in this case would be very short lived and directly undergo Si–N bond cleavage towards **7**. These findings show that changing of the ligand at nitrogen from Dipp in **2** to tBu in **5** heavily influences the mechanism, exchanging slow hydrogen evolution with formal substitution of Si–H with Si–$OSiPh_3$ towards a fast Si–N bond cleavage under addition of silanol under otherwise similar conditions. It remains experimentally elusive whether isomerization processes as for **2a**/**2b** occur also with the tert-butyl substituted silane. Computationally (see below), such isomerization is predicted to be rather unfavorable. Only a single pentacoordinate species would have been expected in the 2D ^{1}H–^{29}Si-NMR spectrum.

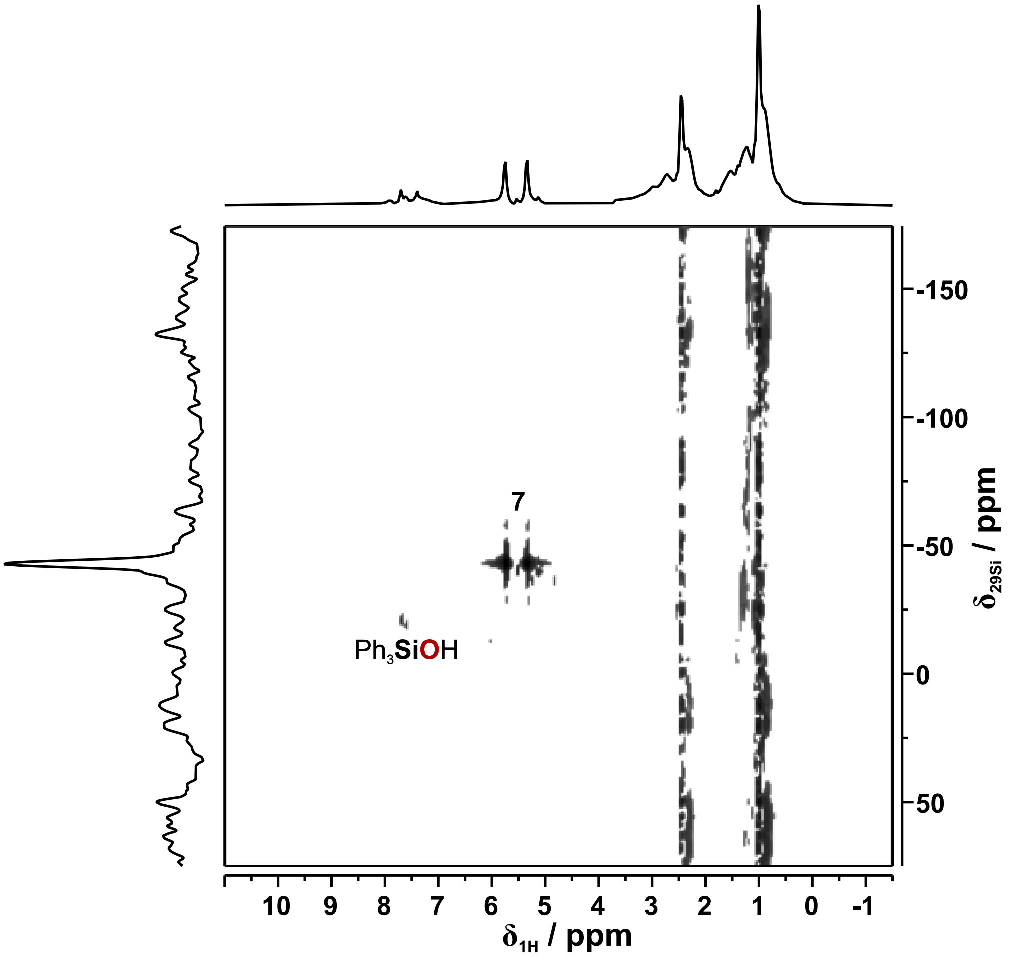

Figure S49. ^{1}H-^{29}Si HMQC (pulse sequence: hmqcgpqf, delay τ optimized for ^{1}J of 233 Hz) at 180 K of a sample of **5** with excess Ph_3SiOH and Et_3N in THF-*d*8 at 180 K. No intermediates could be observed. Instead, full conversion to **7** was found. The traces along 2.5 and 1 ppm in f1 originate from the excess Et_3N.

4. DFT Calculations

4.1. General Considerations

All quantum chemical calculations have been carried out using Orca 6.0.0.[113,114] Initial geometries were generated with Avogadro 4.2.1. Geometries and frequencies were calculated using the composite method PBEh-3c[97] (dispersion correction D3BJ[98,99] and gCP (geometrical counter-poise corection) to account for basis set superposition errors)[100] with improved resolution of identity approximation (chain of spheres) RIJCOSX,[115] which was successfully applied previously in literature for similar compounds.[88] Solvation was included via the conductor-like polarizable continuum model CPCM(THF).[101,102] To increase SCF accuracy, integration grid density has been increased with defgrid3. More strict thresholds for the geometry optimization have been set with the tightOpt and tightSCF keywords. All structures have been approved to be energetic minima by the absence of imaginary frequencies (< 0 cm^{-1}). Relative energies of isomeric structures were compared using the resulting final Gibbs energies G, relative to the lowest lying isomer ΔG at 298 K.

NMR parameters were calculated from optimized geometries using the PBE0 functional.[103] Chemical shifts and J coupling constants were calculated separately, using Jensen's NMR-optimized pcSseg-2[104] or pcJ-2[105] basis sets, respectively. Auxiliary basis sets were generated with the AutoAux keyword.[116] For NMR, the same solvation model was used as for geometries. Chemical shifts were always calculated for all atoms, while J coupling constants were only calculated for pairs of nuclei of interest. Absolute shieldings σ of ^{29}Si and ^{1}H were converted to chemical shifts δ by referencing TMS calculated with the same scheme, using the equation:

$$\delta_{calc} = \sigma_{calc} - \sigma_{ref}$$

An empirical value of 0.7 ppm was further subtracted from the calculated ^{1}H chemical shifts to account for systematic offset errors of the method.

4.2. Evaluation of the Method of Choice

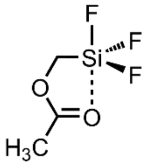

δ_{29Si} = -95 ppm (CD_2Cl_2)

Figure S50. Neutral, pentacoordinate silicon compound [$F_3Si(CH_2OAc)$] (**10**) used for benchmarking.

To determine a good calculation scheme, i.e. delivering correct results for NMR parameters at a reasonable computational cost, various methods were compared: For each method, a geometry optimization of TMS and [$F_3Si(CH_2OAc)$][117] (**10**) with additional calculation of analytical frequencies was conducted. NMR parameters were then calculated using PBE0 / pcSseg-2 for each geometry. Solvation was included both in the geometry optimization and NMR, when indicated (Table S1).

All calculations have been run with the same number of processes (nprocs = 12) on a workstation computer. The NMR calculations of TMS lasted on average 55 s, NMR calculation of the benchmark molecule on average 100 s.

The three common composite methods HF-3c,[118] B97-3c[119] and PBEh-3c[97] were tested, along with PBE,[120,121] PBE0,[103] B3LYP,[122-125] TPSSh,[126] ωB97X-D4[127,128] and M06-2X,[129-132] and varying size of Ahlrichs def2 basis sets.[133]

Table S1. Benchmarking various DFT schemes for computational cost and accuracy of TMS and **10**, followed by calculation of δ_{29Si} by PBE0 / pcSseg-2. The solvent for CPCM is dichloromethane.

DFT-Method	δ_{29Si} / ppm	$\lvert\Delta\delta_{calc-exp}\rvert$	t_{calc} TMS / s	t_{calc} **10** / s	d(Si–O) / Å
exp.[117]	–95				
HF–3c	–100.515	5.515	35	196	2.0142
HF–3c + cpcm	–102.949	7.949	43	216	1.9384
B97–3c	–82.133	12.867	60	247	2.2032
B97–3c+ cpcm	–87.709	7.291	87	409	2.0453
PBEh–3c	–90.129	4.871	68	230	2.0768
PBEh–3c + cpcm	–92.036	2.964	78	376	1.9846
PBE / def2-SVP D4	–87.354	7.646	86	169	2.1229
PBE / def2-SVP D4 + cpcm	–88.948	6.052	98	167	2.0316
PBE0 / def2-TZVP D4	–87.654	7.346	512	1640	2.1086
PBE0 / def2-TZVP D4 + cpcm	–91.642	3.358	353	1420	1.9847
B3LYP / def2-TZVP D4	–78.001	16.999	282	1703	2.3019
B3LYP / def2-TZVP D4 + cpcm	–90.161	4.839	341	1285	2.0245
TPSSh / def2-TZVP D4	–90.393	4.607	350	860	2.0854
TPSSh / def2-TZVP D4 + cpcm	–93.155	1.845	372	932	1.9790
ωB97X-D4 / def2TZVP	–85.727	9.273	348	1916	2.1478
ωB97X-D4 / def2TZVP + cpcm	–91.550	3.45	425	1624	1.9932
M06-2X / def2-TZVPP D3zero	–89.366	5.634	554	1063	2.0849
M06-2X / def2-TZVPP D3zero + cpcm	–92.460	2.54	627	1575	1.9770

Employing TPSSh / def2-TZVP with dispersion correction D4 and CPCM(DCM) for geometry optimization, followed by calculation of NMR via PBE0 / pcSseg-2 resulted in the smallest error from experiment. Nevertheless, PBEh-3c with CPCM(DCM) was chosen for further calculations, as the error is just slightly larger at only a quarter of computational time. Notably, B3LYP / def2-TZVP, a very popular calculation scheme, performed very poorly without implicit solvation, while also taking comparably long to compute, showing how important long-range, covalent interactions and inclusion of solvent are for calculating correct structures. In addition, the distances of the coordinated oxygen towards silicon in **10** are given in Table S1. A clear correlation can be drawn between d(Si–O) and δ_{29Si}, showing that NMR spectroscopy combined with computational modeling is a highly valuable tool to quantitatively determine atom distances in solution apart from the classical methods of NOE spectroscopy. The linear open chain conformer of **10** was also run through the calculation scheme and a δ_{29Si} of –60 ppm was found, in addition with a ΔG = +33 kJ mol^{-1} compared to the pentacoordinate conformer.

The accuracy of the NMR calculation method was further evaluated by calculating δ_{29Si} for the TMS / [$F_3Si(CH_2OAc)$][117] pair with the larger basis sets pcSseg-3 and pcSseg-4, using the geometries obtained by the PBEh-3c + cpcm(DCM) method (Table S2). While the deviation of experimental and calculated chemical shift

further decreased, it was found that the much longer required calculation times would not compensate for slightly better results and pcSseg-2 was used for all further calculations of chemical shifts.

Table S2. Benchmarking of varied sizes of Jensens pcSseg basis sets with the geometry obtained from PBEh-3c / CPCM(DCM).

DFT-Method	δ_{29Si} / ppm	$\Delta\delta_{calc\text{-}exp}$ / ppm	t_{calc} TMS / s	t_{calc} **10** / s
exp.[117]	–95			
PBE0 / pcSseg-2 + cpcm	–92.036	–2.964	54	104
PBE0 / pcSseg-3 + cpcm	–93.647	–1.353	455	537
PBE0 / pcSseg-4 + cpcm	–93.659	–1.341	1727	1829

Key parameters for the neutral starting material **2**, **5** and Ph_3SiOH as well as product disiloxane **3** have been calculated using the scheme described above including correction of δ_{1H} and are listed below in Table S3. The calculated NMR parameters are well in agreement with the experimental data. The coordinates of all optimized compounds are given at the end of the SI (Section 6).

4.3. Calculations of Compounds in the Systems Under Investigation

Table S3. Calculated and observed NMR parameters of **2**, **3**, **5** and Ph_3SiOH.

Compound	$\delta_{29Si\ calc}$ / ppm	$\delta_{29Si\ obs}$ / ppm	$\delta_{1H(SiH)\ calc}$ / ppm	$\delta_{1H(SiH)\ obs}$ / ppm	$^1J_{HSi\ calc}$ / Hz	$^1J_{HSi\ obs}$ / Hz
**2**	–15.1	–13.8	6.02	6.14	233	–233
**3**	–37.0, –12.7	–38.7, –16.6				
**5**	–19.3	–18.0	6.07	6.14	234	–234
Ph_3SiOH	–11.6	–16.0				

The calculated and experimentally observed NMR parameters and Gibbs energies of the six pentacoordinate, anionic isomers **2a-I** to **2a-IV** with equatorial Si-H and **2b-I** and **2b-II** with Si–H occupying an axial position are given in Table S4. The coordination geometry around the pentacoordinate isomers resembled a slightly distorted but clear trigonal bipyramidal mode, except for **2a-IV** which is better described as a basally distorted square pyramid in which the phenyl group is located on the apex.[96]

Table S4. Observed and calculated NMR parameters and Gibbs energies of pentacoordinate states **2a** / **2b**. All attempts to achieve an optimized geometry of a state resembling **2a-I** resulted in the geometry of **2b-II** (*).

Structure	N_{Ax}	R_{Ax}	$\delta_{29Si\ calc}$ / ppm	$\delta_{1H(SiH)\ calc}$ / ppm	$^{1}J_{HSi\ calc}$ / Hz	*G* / Eh	Rel. Δ*G* / kJ mol^{-1}
**2a$_{exp}$**	N–Dipp	Ph_3SiO	–92.1	5.91	280		
**2b$_{exp}$**	N–H	H	–87.8	4.81	197		
**2a-I**	N–Dipp	Ph	*	*	*	*	*
**2a-II**	N–H	Ph	–102.5	6.83	–213	–2385.03628234	22.72497168
**2a-III**	N–Dipp	Ph_3SiO	–98.7	5.66	–245	–2385.04447218	1.222563144
**2a-IV**	N–H	Ph_3SiO	–117.4	6.65	–214	–2385.03694935	20.97373826
**2b-I**	N–Dipp	H	–118.5	6.01	–177	–2385.03985244	13.35168127
**2b-II**	N–H	H	–100.0	4.76	–187	–2385.04493783	0

DFT calculations were also performed for conceivable neutral, *N*-protonated pentacoordinate derivatives of **2a** and **2b**. Protonation at N(Dipp) gave rise to six diastereomers (**2c-I–VI**), as the nitrogen atom becomes an additional chirality center, while protonation at NH generated three neutral, chiral isomers (**2d-I–III**) with *Si*-centered chirality but no additional stereogenic center at nitrogen. In all optimized structures, the protonated nitrogen occupies an axial position. Overall, 18 neutral and 12 anionic stereoisomers were identified as possible candidates in the reaction, with only one enantiomer calculated for each diastereomer (see Table S5).

All **2c** structures deviate strongly from pentacoordinate, zwitterionic geometries, instead resembling compound **7** with a partially broken, elongated N–Si bond and tetrahedral coordination at silicon. This is reflected in the calculated NH(Dipp)–Si Mayer bond orders (0.5 ± 0.1) and ^{29}Si NMR chemical shifts (–27 to –44 ppm), which are inconsistent with the experimental data; these isomers were therefore excluded.

In contrast, the NH-protonated isomers (**2d-I–III**) largely retained trigonal-bipyramidal coordination at silicon. While **2d-I** and **2d-III** exhibit calculated ^{29}Si NMR shifts closer to the experimental values at low temperature, their ^{1}H NMR shifts did not fit to observed values, and they were rather high in energy in comparison with the other possible isomers. **2d-II**, the protonated analogue of **2b-II**, matches the experimental NMR data most closely. Experiments with deuterated silanol (see Section 3.6) indicate NH protonation/deprotonation in the reaction mixture, suggesting that the proton can indeed reside on the amine function. Nevertheless, only **2d-II** is consistent with the observed results, whereas the calculated data for anionic intermediates, including energy differences and the **2a:2b** ratio, agree well with experimental data.

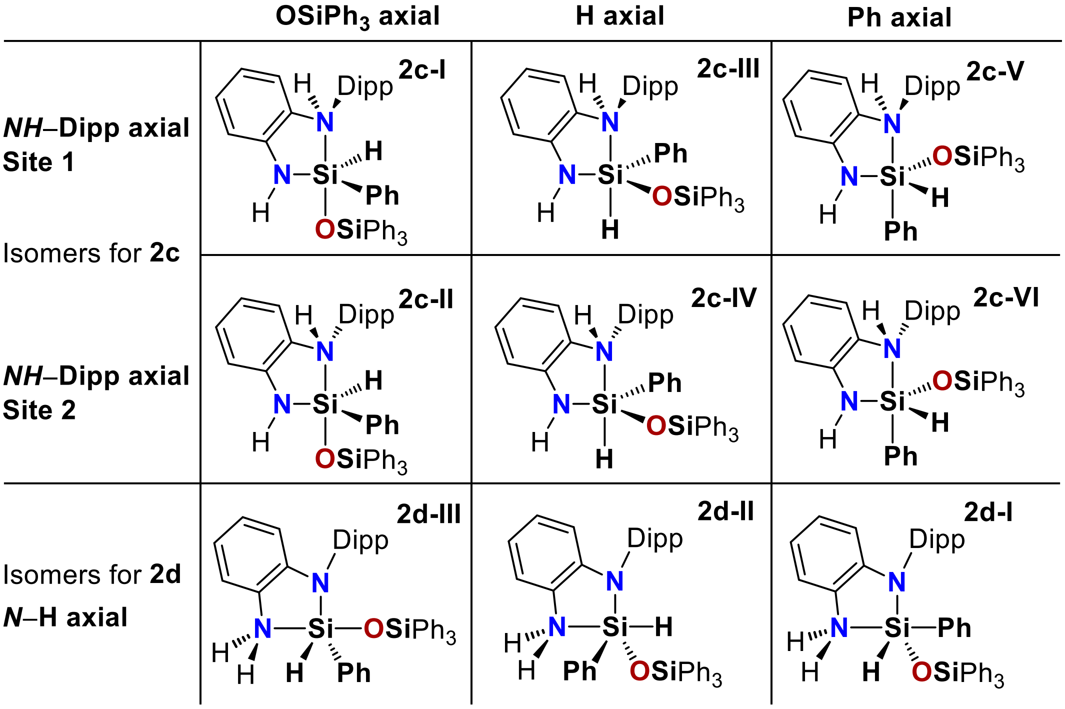

Figure S51. Structures of plausible neutral pentacoordinate silicon intermediates (formal charges are omitted for clarity).

Table S5. Observed and calculated NMR parameters and Gibbs free energies of zwitterionic pentacoordinate states **2c** / **2d**. The entry H–Site denominates the protonation site on N(Dipp): If H is in the entry, then protonation occurred from the site that it is syn with the equatorial H substituent on silicon.

Structure	N_{Ax}	R_{Ax}	H Site*	$\delta_{29Si\ calc}$ / ppm	$\delta_{1H(SiH)\ calc}$ / ppm	$^{1}J_{HSi\ calc}$ / Hz	*G* / Eh	Rel. Δ*G* / kJ mol^{-1}
**2c-I**	N–Dipp	$OSiPh_3$	H	–27.4	5.72	–243	–2386.21481587218	16.27679761
**2c-II**	N–Dipp	$OSiPh_3$	Ph	–43.8	5.53	–271	–2386.22101538085	0
**2c-III**	N–Dipp	H	Ph	–39.8	5.19	–225	–2386.21827947604	7.183112607
**2c-IV**	N–Dipp	H	$OSiPh_3$	–44.3	5.09	–214	–2386.21976471495	3.283620819
**2c-V**	N–Dipp	Ph	$OSiPh_3$	–44.5	5.89	–285	–2386.22022221774	2.08244816
**2c-VI**	N–Dipp	Ph	H	–40.4	5.82	–263	–2386.21724409587	9.901501173
**2d-I**	N–H	Ph	/	–74.2	5.82	–267	–2386.21658123382	11.64184416
**2d-II**	N–H	H	/	–75.2	4.59	–195	–2386.22010148288	2.399437292
**2d-III**	N–H	$OSiPh_3$	/	–96.8	6.94	–280	–2386.21751207862	9.197912998

Table S6. Calculated NMR parameters and Gibbs energies of pentacoordinate states originating from *t*Bu-Silane **5** as starting material.

Structure	N_{Ax}	R_{Ax}	$\delta_{29Si\ calc}$ / ppm	$\delta_{1H(SiH)\ calc}$ / ppm	$^{1}J_{HSi\ calc}$ / Hz	*G* / Eh	Rel. Δ*G* / kJ mol^{-1}
**5a-I**	N–*t*Bu	Ph	–106.6	6.41	–251	–2076.14665716	15.75456330
**5a-II**	N–H	Ph	–93.5	5.75	–218	–2076.13304031	51.50557574
**5a-III**	N–*t*Bu	Ph_3SiO	–109.8	6.60	–268	–2076.15265776	0
**5a-IV**	N–H	Ph_3SiO	–101.6	5.86	–255	–2076.13450038	47.67216487
**5b-I**	N–*t*Bu	H	–114.7	5.13	–130	–2076.14262062	26.35249100
**5b-II**	N–H	H	–98.8	6.02	–188	–2076.14585473	17.86134166

5. Single-Crystal X-Ray Diffraction Analysis

The crystals were selected and measured on a Gemini Ultra diffractometer equipped with an Atlas S2 CCD detector (**7**), XtaLAB Synergy R, DW system diffractometer equipped with a HyPix-Arc 150 detector (**6**), a GV50 diffractometer equipped with a TitanS2 detector (**9**) or a Supernova diffractometer equipped with either a TitanS2 detector (**1**) or an Atlas detector (**2, 3**). The crystals were kept at T = 123(1) K (**1**, **2**, **3**, **7**, **9**) or T = 100(1) K (**6**) during data collection. Data collection and reduction were performed with CrysAlisPro (Version 1.171.41.54a, Version 1.171.41.76a or Version 1.171.43.36a).[134] A numerical absorption correction using spherical harmonics as implemented in SCALE3 ABSPACK was applied. Using Olex2,[135] all structures except compound **2** were solved with ShelXT[136] and a least-square refinement on F^2 was carried out with ShelXL.[137] To enable a reliable analysis of the Si–H bond in compound **2**,[138] its structure was solved with NoSpherA2[139] with ORCA 5.0[113] of the refinement program olex2.refine.[140] All non-hydrogen atoms were refined anisotropically. Hydrogen atoms at the carbon atoms were located in idealized positions and refined isotropically according to the riding model. Figures were created with Mercury Version 3.10.3.[141]

Compound **1**: The asymmetric unit contains one molecule of compound **1**.
Compound **2**: The asymmetric unit contains one molecule of compound **2**. A RIGU restraint was applied.
Compound **3**: The asymmetric unit contains one molecule of compound **3**.
Compound **6**: The asymmetric unit contains one molecule of compound **6**.
Compound **7**: The asymmetric unit contains one molecule of compound **7**.
Compound **9**: The asymmetric unit contains one molecule of compound **9**.

Table S7. Crystal data and structure refinement of compounds **1**, **2**, and **3**.

Compound	**1**	**2**	**3**
CCDC number[90]	2478084	2478086	2478085
Formula	$C_{18}H_{24}N_2$	$C_{24}H_{28}N_2Si$	$C_{42}H_{42}N_2OSi_2$
M / g·mol^{-1}	268.39	372.589	646.95
T / K	123.01(10)	123.00(10)	123.00(10)
Crystal system	Triclinic	Monoclinic	Triclinic
Space group	$P\bar{1}$	$P2_1/c$	$P\bar{1}$
a / Å	10.3849(2)	10.0015(1)	9.5270(3)
b / Å	12.3450(3)	12.0551(2)	11.1155(4)
c / Å	14.6119(3)	17.7528(3)	16.8771(6)
α / °	110.103(2)	90	98.976(3)
β / °	91.635(2)	94.750(1)	94.087(3)
γ / °	114.796(2)	90	93.751(3)
V / Å^3	1563.86(7)	2133.09(6)	1755.54(11)
Z	4	4	2
Z'	2	1	1
ρ / g·cm^{-3}	1.140	1.160	1.224
μ / mm^{-1}	0.506	1.030	1.184
Crystal size / mm^3	0.29 × 0.15 × 0.13	0.24 × 0.20 × 0.09	0.17 × 0.09 × 0.06
λ / Å	1.54184	1.54184	1.54184
Radiation type	Cu K_α	Cu K_α	Cu K_α
θ range / °	4.261 – 67.010	4.440 – 76.110	4.039 – 72.926
Reflections, collected	27565	17365	19642
Reflections, independent	5528	4374	6828
Reflections with I > 2(I)	5115	4125	5924
R_{int}	0.0511	0.0195	0.0287
Parameters	414	496	432
Restraints	27	3	0
GooF	1.058	1.088	1.034
wR_2 (all data)	0.1258	0.0404	0.0906
wR_2	0.1225	0.0397	0.0857
R_1 (all data)	0.0500	0.0169	0.0416
R_1	0.0474	0.0155	0.0345
$\Delta\rho_{fin}$ (max / min) / e·Å^{-3}	0.32 / –0.32	0.24 / –0.12	0.36 / –0.30

Table S8. Crystal data and structure refinement of compounds **6**, **7**, and **9**.

Compound	**6**	**7**	**9**
CCDC number[90]	2478088	2478089	2478087
Formula	$C_{42}H_{36}O_2Si_3$	$C_{34}H_{36}N_2OSi_2$	$C_{34}H_{34}N_2OSi_2$
M / g·mol^{-1}	656.98	544.83	542.81
T / K	100.01(10)	123.01(10)	124(1)
Crystal system	Triclinic	Triclinic	Triclinic
Space group	$P\bar{1}$	$P\bar{1}$	$P\bar{1}$
a / Å	12.6336(6)	9.9562(2)	10.8425(4)
b / Å	13.1403(6)	11.9185(2)	12.0185(5)
c / Å	13.3843(4)	14.1045(3)	12.4936(4)
α / °	67.363(4)	72.173(2)	104.816(3)
β / °	62.497(4)	74.637(2)	92.098(3)
γ / °	68.033(4)	75.329(2)	109.754(4)
V / Å^3	1765.90(15)	1509.12(6)	1467.69(10)
Z	2	2	2
Z'	1	1	1
ρ / g·cm^{-3}	1.236	1.199	1.228
μ / mm^{-1}	1.508	1.282	0.952
Crystal size / mm^3	0.26 × 0.10 × 0.08	0.55 × 0.35 × 0.31	0.22 × 0.12 × 0.10
λ / Å	1.54184	1.54184	1.54184
Radiation type	Cu K_α	Cu K_α	Cu K_α
θ range / °	3.754 – 73.537	3.362 – 71.992	3.335 – 74.216
Reflections, collected	54619	36898	21102
Reflections, independent	6889	5848	7807
Reflections with I > 2(I)	5913	5506	6364
R_{int}	0.0399	0.0307	0.0425
Parameters	568	363	359
Restraints	0	0	0
GooF	1.058	1.033	1.044
wR_2 (all data)	0.1050	0.0842	0.1304
wR_2	0.1008	0.0827	0.1197
R_1 (all data)	0.0443	0.0333	0.0583
R_1	0.0374	0.0316	0.0464
$\Delta\rho_{fin}$ (max / min) / e·Å^{-3}	0.63 / –0.39	0.35 / –0.30	0.47 / –0.37

Compound 1

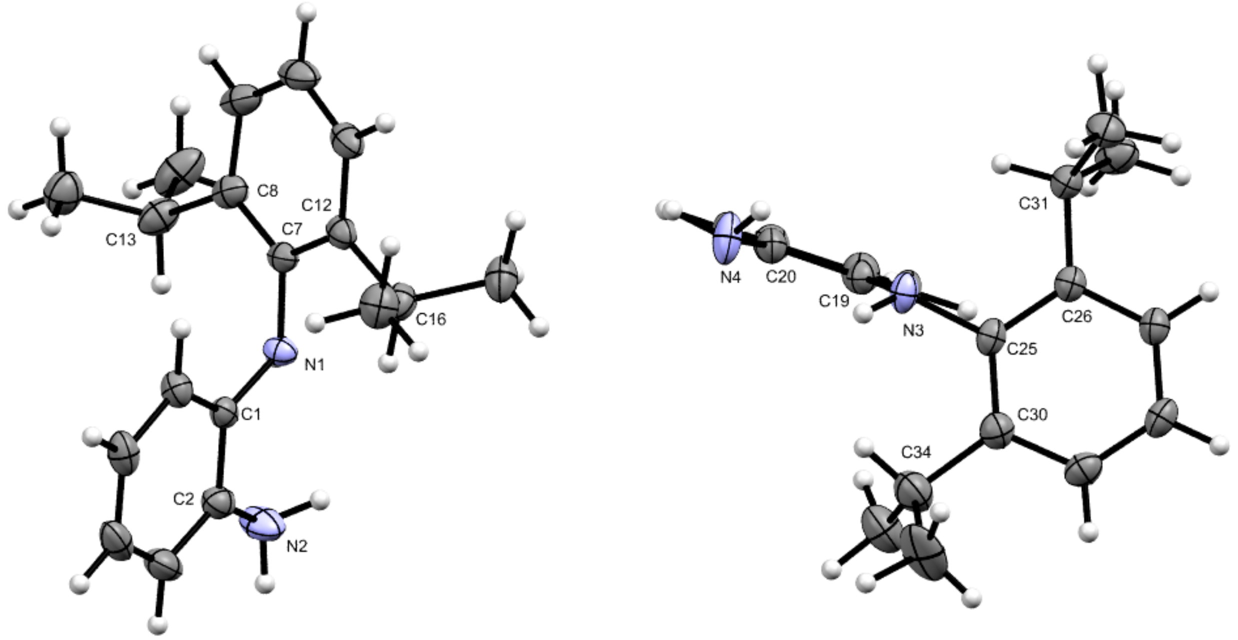

Figure S52. Molecular structure of compound **1** in the crystal (displacement ellipsoids of non-hydrogen atoms set at the 50% probability level).

Table S9. Selected bond lengths / Å and selected bond angles / ° of compound **1**.

Selected bond lengths / Å		Selected bond angles / °	
N1–C1	1.411(2)	C1–C2–N2	118.7(1)
C1–C2	1.410(3)	N1–C1–C2	117.5(1)
C2–N2	1.400(2)	C1–N1–C7	119.6(1)
N1–C7	1.433(2)	N1–C7–C8	120.8(1)
C7–C8	1.404(2)	N1–C7–C12	118.2(1)
C7–C12	1.406(2)		
C8–C13	1.521(2)		
C12–C16	1.517(2)		

Compound 2

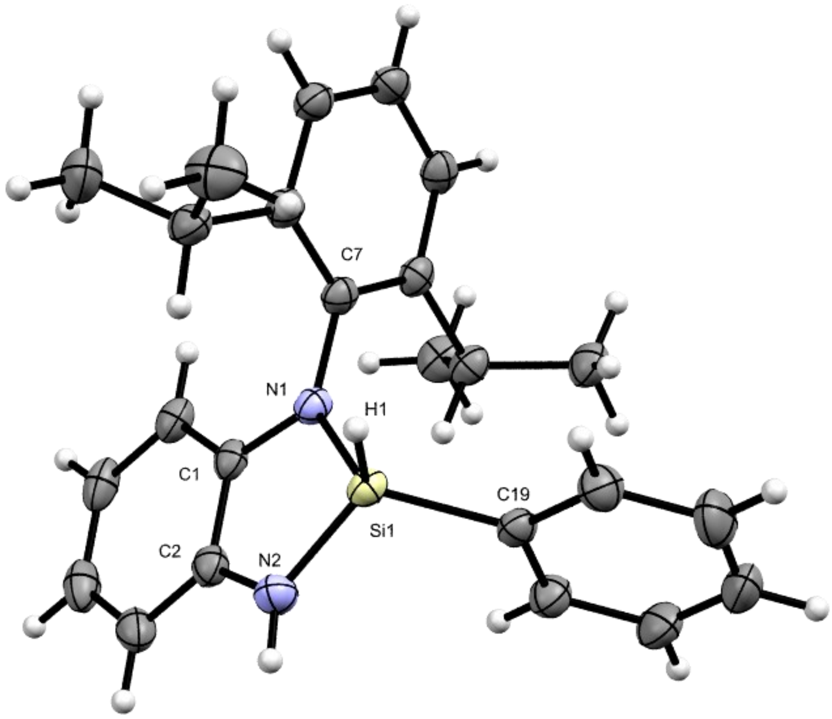

Figure S53. Molecular structure of compound **2** in the crystal (displacement ellipsoids of non-hydrogen atoms set at the 50% probability level).

Table S10. Selected bond lengths / Å and selected bond angles / ° of compound **2**.

Selected bond lengths / Å		Selected bond angles / °	
N2–Si1	1.7279(5)	N1–Si1–N2	91.32(2)
N1–Si1	1.7413(4)	N1–Si1–C19	117.01(2)
C19–Si1	1.8501(5)	N2–Si1–C19	113.62(2)
Si1–H1	1.546(7)	N2–Si1–H1	118.8(3)
C1–C2	1.4164(7)	N1–Si1–H1	110.1(3)
N1–C7	1.4263(6)	C19–Si1–H1	106.0(3)
C1–N1	1.4002(6)	C2–N2–Si1	112.25(4)
C2–N2	1.3956(6)	C1–N1–Si1	111.64(3)
		Si1–N1–C7	127.41(3)

Compound 3

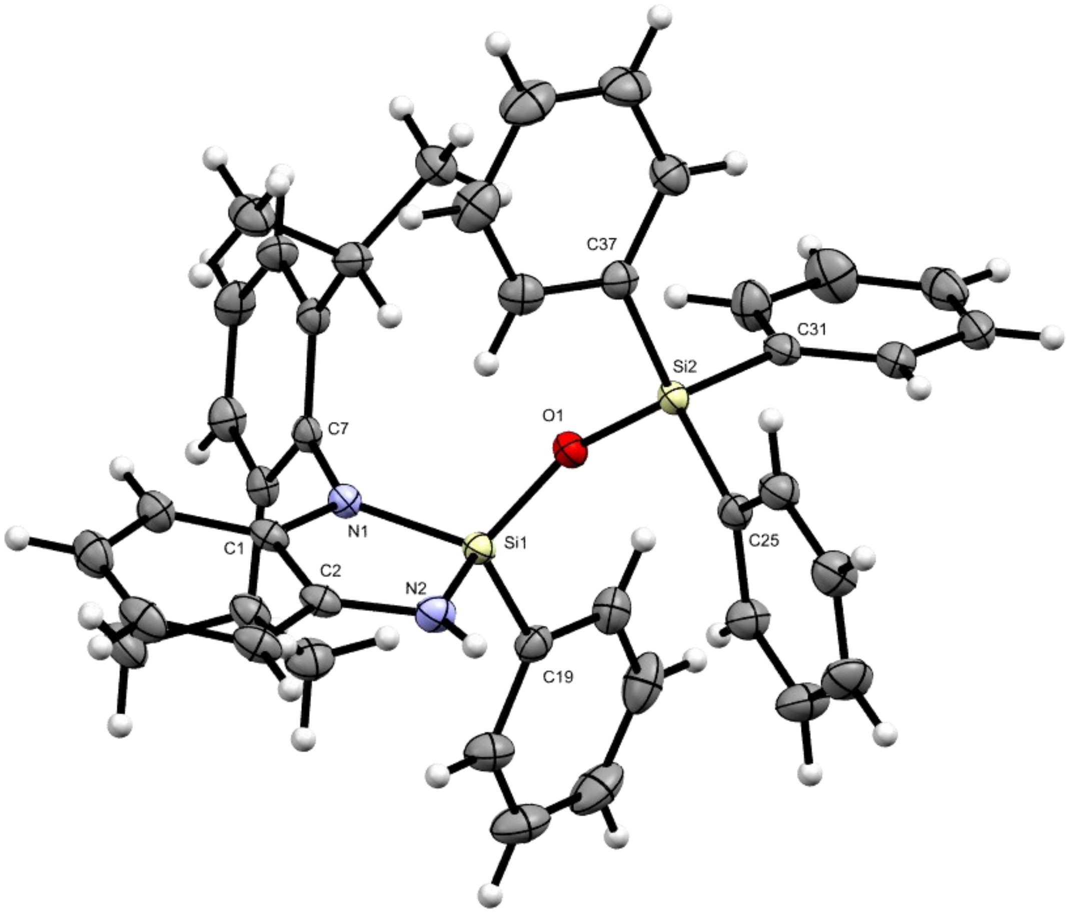

Figure S54. Molecular structure of compound **3** in the crystal (displacement ellipsoids of non-hydrogen atoms set at the 50% probability level).

Table S11. Selected bond lengths / Å and selected bond angles / ° of compound **3**.

Selected bond lengths / Å		Selected bond angles / °	
N2–Si1	1.724(1)	Si1–O1–Si2	147.82(7)
N1–Si1	1.733(1)	N1–Si1–N2	91.81(6)
C19–Si1	1.852(1)	N1–Si1–C19	113.14(6)
Si1–O1	1.618(1)	N2–Si1–C19	114.46(6)
C1–C2	1.415(2)	N2–Si1–O1	115.86(6)
N1–C7	1.438(2)	N1–Si1–O1	114.13(5)
C1–N1	1.405(2)	C19–Si1–O1	107.14(6)
C2–N2	1.398(2)	C2–N2–Si1	112.14(9)
Si2–O1	1.6420(9)	C1–N1–Si1	111.27(9)
Si1–C25	1.870(1)	Si1–N1–C7	128.30(9)
Si1–C31	1.866(1)	O1–Si2–C25	109.05(6)
Si1–C37	1.866(1)	O1–Si2–C31	106.88(6)
		O1–Si2–C37	108.90(6)

Compound 6

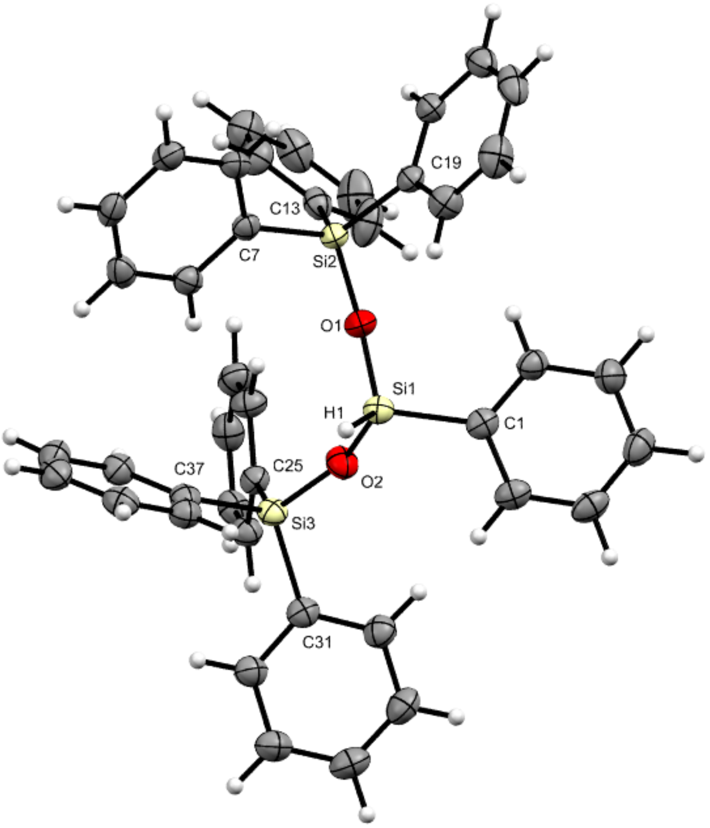

Figure S55. Molecular structure of compound **6** in the crystal (displacement ellipsoids of non-hydrogen atoms set at the 50% probability level).

Table S12. Selected bond lengths / Å and selected bond angles / ° of compound **6**.

Selected bond lengths / Å		Selected bond angles / °	
Si1–H1	1.42(3)	O1–Si1–O2	108.92(8)
Si1–C1	1.854(2)	O1–Si1–C1	108.90(8)
Si1–O1	1.598(1)	O2–Si1–C1	108.58(8)
Si1–O2	1.611(1)	O1–Si1–H1	109.9(9)
Si2–O1	1.623(1)	O2–Si1–H1	108.1(9)
Si2–C7	1.870(2)	Si1–O1–Si2	165.4(1)
Si2–C13	1.865(2)	O1–Si2–C7	109.71(8)
Si2–C19	1.865(1)	O1–Si2–C13	108.05(8)
Si3–O2	1.632(1)	O1–Si2–C19	109.06(8)
Si3–C25	1.863(2)	Si1–O2–Si3	151.9(1)
Si3–C31	1.863(2)	O2–Si3–C25	108.58(8)
Si3–C37	1.871(2)	O2–Si3–C31	106.21(8)
		O2–Si3–C37	109.89(8)

Compound 7

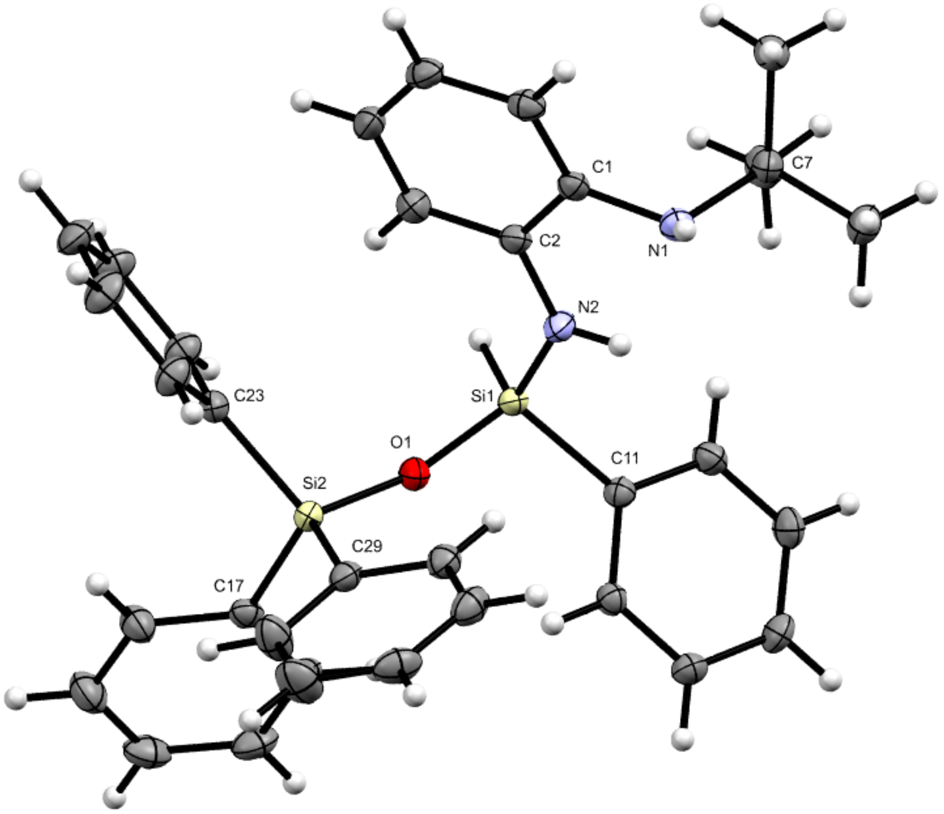

Figure S56. Molecular structure of compound **7** in the crystal (displacement ellipsoids of non-hydrogen atoms set at the 50% probability level).

Table S13. Selected bond lengths / Å and selected bond angles / ° of compound **7**.

Selected bond lengths / Å		Selected bond angles / °	
N1–C1	1.435(2)	C1–N1–C7	118.5(1)
C1–C2	1.408(2)	C2–N2–Si1	129.8(1)
C2–N2	1.399(2)	N2–Si1–O1	113.60(5)
N1–C7	1.491(1)	N2–Si1–H1	110.2(7)
N2–Si1	1.709(1)	O1–Si1–H1	106.7(7)
Si1–H1	1.37(2)	Si1–O1–Si2	162.46(7)
Si1–C11	1.861(1)	N2–Si1–C11	106.80(6)
Si1–O1	1.6218(8)	O1–Si1–C11	106.46(5)
Si2–O1	1.6239(8)	O1–Si2–C17	107.88(5)
Si2–C17	1.869(1)	O1–Si2–C23	109.99(5)
Si2–C23	1.868(1)	O1–Si2–C29	108.33(5)
Si2–C29	1.866(1)		

Compound 9

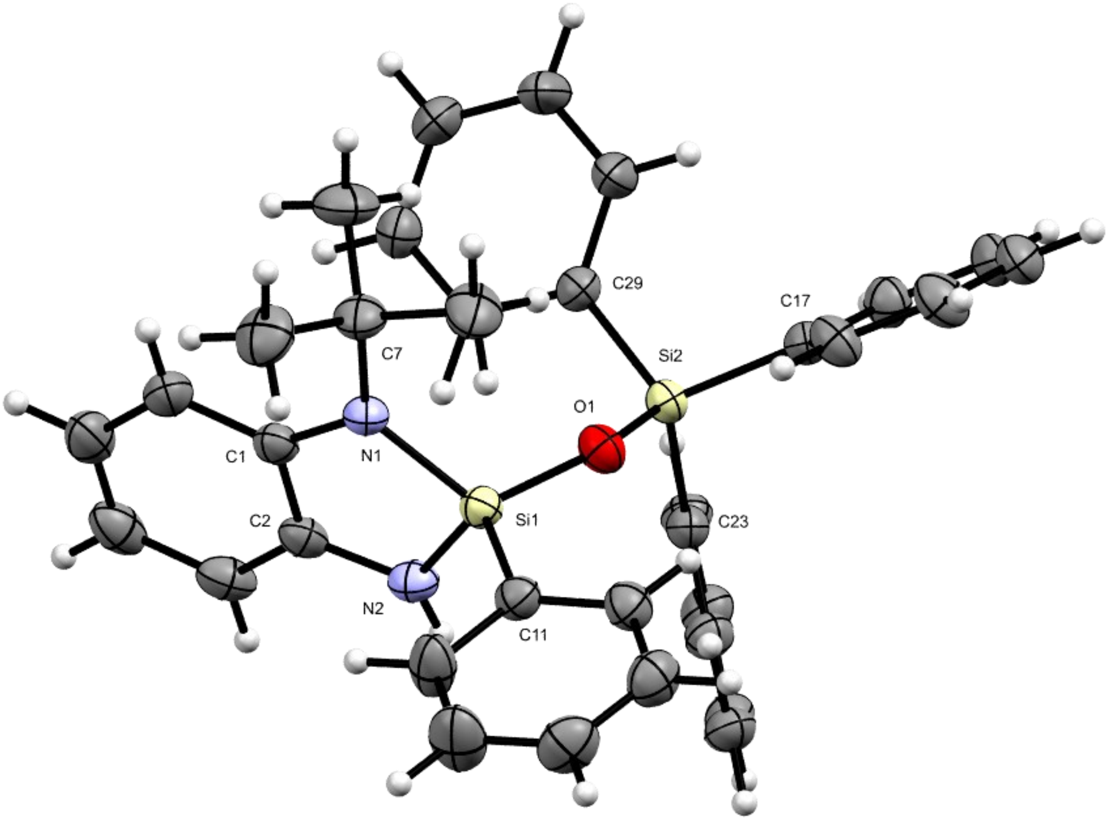

Figure S57. Molecular structure of compound **9** in the crystal (displacement ellipsoids of non-hydrogen atoms set at the 50% probability level).

Table S14. Selected bond lengths / Å and selected bond angles / ° of compound **9**.

Selected bond lengths / Å		Selected bond angles / °	
N1–C1	1.411(2)	Si1–O1–Si2	140.10(9)
C1–C2	1.415(2)	N1–Si1–N2	92.55(7)
C2–N2	1.400(3)	N1–Si1–C11	115.56(8)
N1–C7	1.485(2)	N2–Si1–C11	113.41(8)
Si1–N1	1.734(2)	N2–Si1–O1	113.78(7)
Si1–N2	1.719(1)	N1–Si1–O1	116.67(7)
Si1–C11	1.856(2)	C11–Si1–O1	105.02(7)
Si1–O1	1.625(1)	C2–N2–Si1	111.5(1)
Si2–O1	1.642(1)	C1–N1–Si1	110.7(1)
Si2–C17	1.862(2)	Si1–N1–C7	123.1(1)
Si2–C23	1.871(1)	O1–Si2–C17	105.93(7)
Si2–C29	1.867(2)	O1–Si2–C23	107.39(7)
		O1–Si2–C29	110.50(7)

6. Cartesian Coordinates of Optimized Structures

All values are given in Å (1 Å = 10^{-10} m).

TMS

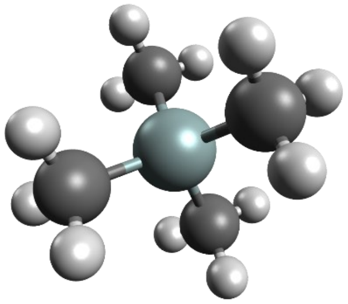

E = –448.3680001 Eh

Si	-1.25204444775515	-1.29371729416827	0.48788562973826
C	-1.45124745843267	0.28070463281701	-0.53246791386079
C	0.56267352076965	-1.51657844945836	0.95353396658244
C	-2.29219491394403	-1.16260380345586	2.05652320931856
C	-1.82769697670753	-2.77720630611132	-0.52585290472054
H	-1.98509701571068	-0.31221936205151	2.66830062094814
H	-3.35064274791420	-1.03316500589501	1.82269292114654
H	-2.19951988977227	-2.05966927787198	2.67170427979864
H	-2.87651538885142	-2.68050723074487	-0.81286029190591
H	-1.24509214824940	-2.88312077879913	-1.44297390746489
H	-1.72600321897977	-3.70734095708905	0.03652008165965
H	0.92965063024336	-0.67307735017667	1.54138454164381
H	1.19430413248157	-1.59790807228852	0.06686522876304
H	0.71306019361991	-2.42025013827494	1.54726871022468
H	-0.86203481932718	0.23750855140315	-1.45046130496702
H	-1.12529985656656	1.16066194858971	0.02536148081343
H	-2.49258959490359	0.44077889357562	-0.81835434771807

Compound 2

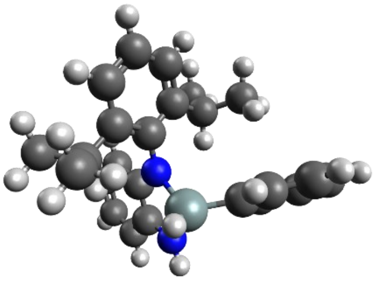

E = –1327.44257191 Eh

Si	-0.69695028882310	2.15354370538759	1.36023336968014
H	-1.18670353853125	2.61509588930181	2.68988246183902
N	-1.19418894700683	3.20908706323714	0.06005078477258
N	-1.53888832089583	0.81848588491572	0.62922129035393
C	1.13280959723246	1.87935506601806	1.55006537442615
C	1.83887120508963	2.50398037816688	2.57694179629979
C	3.21370965329773	2.35477120938591	2.69036894402211
C	3.89835973726527	1.56689701391146	1.77745571470558
C	3.20942708582751	0.92881145669711	0.75444606198165
C	1.83785937696868	1.08695832873941	0.64218125358914
C	-0.94170521176833	4.59821771618057	-0.04423547641331
C	-1.82933507774143	5.50241524174017	0.55720688082859
C	-1.55340353846203	6.86162618669199	0.46327314386948
C	-0.43714254948224	7.31775246758536	-0.21398529166950
C	0.42021367450125	6.41471609400562	-0.81517296760661
C	0.18622854416336	5.04569746293604	-0.74622970959771
C	1.13300806848341	4.09561667364256	-1.44497365566025
C	2.56225860593494	4.23718184721350	-0.92447798423614
C	1.07470762609317	4.28583360606297	-2.96101347956717
C	-3.07468857857531	5.04782885070033	1.28641773458918
C	-4.33635883131761	5.55588748256341	0.58880924495134
C	-3.04591145573364	5.46726706013981	2.75571793565838
C	-1.99440619984234	2.54392452780286	-0.86727643851986
C	-2.19346577197763	1.18923379834650	-0.54082648877466
C	-2.96791780577780	0.38882330777866	-1.35730673688769
C	-3.54815578649396	0.93337812947852	-2.50279225768061
C	-3.35272077853700	2.26421313087216	-2.82072231525348
C	-2.57176655875034	3.08006650334433	-2.00205504276296
H	-2.41947238012749	4.12362242163369	-2.24741797571312
H	-3.80662380416708	2.68247303664943	-3.70898944239464
H	-4.15516519886204	0.30530123835145	-3.14080593136992
H	-3.12495864134081	-0.65399683337183	-1.10882631260815
H	-3.11395156116139	3.95835474867903	1.25887776985118
H	-3.04546323639938	6.55274344572209	2.86864878997594
H	-2.15964855338667	5.08193360984294	3.26078347132669
H	-3.92232320794098	5.08415186271342	3.28074612567939
H	-5.22884507730743	5.17934333219655	1.09097092941215
H	-4.39152925439140	6.64596433553258	0.59312977449580
H	-4.37275225613311	5.22162377723099	-0.44785418172873
H	-2.22406943176474	7.57710172856426	0.92384991329157
H	-0.23785372775940	8.37966146145990	-0.27752263054707
H	1.28717553779811	6.78206991983889	-1.35087775172870
H	0.80935117762976	3.07583879343158	-1.23584058482688
H	3.21235680436167	3.49672300185693	-1.39252196473262
H	2.97851011602007	5.22254176684660	-1.14267417231129
H	2.61157222877248	4.08366095264927	0.15344282339022
H	1.72312750266954	3.56501098913813	-3.46171776989012
H	1.40310276825141	5.28417059968349	-3.25615302886510
H	0.06290497599955	4.14015449559910	-3.33923978980584
H	1.31116986437927	3.11661327581875	3.29972679506134
H	3.74974660024401	2.84979109516379	3.48953449474599
H	4.97051060250647	1.44702855097469	1.86387328763408
H	3.74425645940432	0.31068049317695	0.04512361274793
H	1.30764508466707	0.58885452148973	-0.16155990577755
H	-1.62976732710253	-0.12845270371915	0.95089950775033

Compound 2a-II

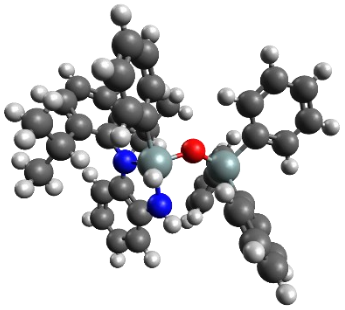

E = –2385.03628234 Eh

Si	0.00362236618938	0.00118720929576	0.00034688706657
H	0.01277446282380	0.01403263009894	1.53128592484283
N	-1.33290775259594	-0.40283191423964	-1.18664269743546
N	0.16767135251766	-1.86184219440763	0.06269189800719
C	-0.38667180611194	1.93086515851683	0.04557234549056
C	-0.86632279658899	2.48095247330531	1.23402419160702
C	-1.10674293216406	3.84363833126125	1.38919980491525
C	-0.85723779327450	4.71327645381163	0.33838641349482
C	-0.36007816221396	4.20212983716764	-0.85608400477781
C	-0.13085630587381	2.83962982607637	-0.98783300050190
C	-2.09501808961423	0.51810725534466	-1.94185164421946
C	-3.24751896404400	1.10099942303265	-1.38481405305256
C	-3.98396831341063	1.99560095921874	-2.15505275199323
C	-3.60993834017016	2.30279435153532	-3.45268445270589
C	-2.48671029901538	1.70812683194520	-3.99898130408975
C	-1.71480851322626	0.81359041520156	-3.26289489733771
C	-0.51267596021529	0.17182921633470	-3.92737775191648
C	0.46000138866471	1.20119121964769	-4.50416885900785
C	-0.94803179968626	-0.79614522176604	-5.03029936911724
C	-3.72403977764036	0.74600195407553	0.00806992711312
C	-4.73908832798494	-0.39939348947772	-0.04435274140694
C	-4.31986202691015	1.93146078496839	0.76406315839449
C	-1.58584448594335	-1.74838533804253	-1.40552893274372
C	-0.72243181758358	-2.58333862890066	-0.65328860682582
C	-0.87358342562998	-3.96534640873530	-0.72467716938255
C	-1.85816199510699	-4.52251089961312	-1.54557439736596
C	-2.69037617651821	-3.70598979974717	-2.28978204853515
C	-2.55654200043435	-2.31265813505387	-2.21485724762164
H	-3.22213675744441	-1.67996084301192	-2.78927228917246
H	-3.45263995687776	-4.13421304382396	-2.92844226791961
H	-1.96343253985547	-5.59958926291899	-1.59577062169253
H	-0.21865515802834	-4.60621141665701	-0.14423569045817
H	-2.86217858147037	0.39610125745698	0.57818764767361
H	-5.27742975934940	2.24817465863971	0.34609181750470
H	-3.64694852015612	2.78832718864654	0.76260577287525
H	-4.50275012390295	1.65304825173553	1.80337253539735
H	-5.05619960624616	-0.67750032702433	0.96300112851100
H	-5.62989255612823	-0.10311454426461	-0.60327543318328
H	-4.32264762081207	-1.28594503250646	-0.52056430228959
H	-4.87046589380122	2.45974673417569	-1.74105639148862
H	-4.19709073893706	2.99988820532479	-4.03750994047387
H	-2.20581105730377	1.94468971406163	-5.01885515307604

H	0.02864252540618	-0.40269592798467	-3.17552077158594
H	0.85397918390457	1.86710017024317	-3.73675523538999
H	1.31075175129652	0.69408119279469	-4.96108484228208
H	-0.00537830608986	1.81844702247418	-5.27471997731352
H	-0.07847266645589	-1.30056817624754	-5.45624250792377
H	-1.45734344514433	-0.27000330700400	-5.84101713322560
H	-1.62195714243868	-1.56176654040073	-4.64784420736397
H	-1.05532306933486	1.82176179562481	2.07699971139053
H	-1.48574512552544	4.22453257465272	2.33061516946887
H	-1.03829219450632	5.77556146179608	0.44818881445631
H	-0.14859623603485	4.86919917278136	-1.68383241360979
H	0.26250851949623	2.47890179757766	-1.92940931057327
H	0.70128248519807	-2.35115922180299	0.76457034027155
O	1.56062955899740	0.27598723004173	-0.66301497098799
Si	2.87481486438265	-0.60707378845592	-1.07175981583549
C	2.67337033639762	-1.67389448952063	-2.60999507307603
C	3.12344691427382	-1.22538978152524	-3.85405390064308
C	2.95443911135452	-1.98870358829500	-5.00244206656563
C	2.32780571016719	-3.22475183795344	-4.92710488320659
C	1.87710006124456	-3.69284740114356	-3.69951237687086
C	2.05328935544455	-2.92535406640307	-2.55702235794288
C	4.22293295863892	0.65527649268250	-1.45122774800120
C	5.54495207106653	0.24698078026209	-1.64763000448812
C	6.54748344460289	1.16051948422767	-1.94051708837703
C	6.24396324177274	2.51272496904148	-2.04272362919605
C	4.93779023753571	2.93963093518892	-1.84966404876539
C	3.93940242828009	2.01803019612961	-1.55620137427395
C	3.48029240083440	-1.68041561643943	0.35031848064513
C	3.22853232791892	-1.30318594031440	1.67260755756606
C	3.70907546066240	-2.05113558219880	2.73853765811689
C	4.45962105861407	-3.19598328943905	2.50218007887999
C	4.72475390283498	-3.58572793521778	1.19681526397636
C	4.23797531554214	-2.83340055910582	0.13494486223239
H	3.61499564114541	-0.26300514429615	-3.93619389072434
H	3.31075300567817	-1.61733338687496	-5.95516961123783
H	2.19151420610189	-3.82103589725749	-5.82056420419076
H	1.38527043779699	-4.65507543962709	-3.63094740584433
H	1.69427440206800	-3.30837022994369	-1.61136790673902
H	5.80444983445545	-0.80385095348069	-1.57093900067943
H	7.56480555079135	0.82005251682319	-2.08765637020849
H	7.02320303890640	3.22921450289034	-2.27014826423370
H	4.69460566723559	3.99211511627812	-1.92595400363157
H	2.92634823299363	2.37141068694977	-1.40589228698368
H	4.45051447011934	-3.16026922029468	-0.87675464677496
H	5.30740225984077	-4.47792664091163	1.00460458879213
H	4.83405774782062	-3.78196572328976	3.33196175858654
H	3.49681095827512	-1.74210509989727	3.75435071087274
H	2.63891466850893	-0.41721715382266	1.88002690038851

Compound 2a-III

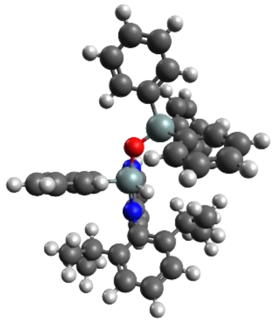

E = –2385.04447218 Eh

Si	-0.74637574740342	1.24313753055941	2.21869407358826
H	-0.89441243342969	2.20105638332518	3.38133663742639
N	-1.32523802338565	2.65293823922462	1.06620467173955
N	-1.74737279941922	0.26051459661575	1.10341061987920
C	1.09805928992805	1.17053585536150	1.77934325423444
C	2.07278008644646	1.46402049200488	2.73463822890532
C	3.42937832530936	1.38055741459397	2.44429204699107
C	3.84391471964605	0.98972849368273	1.17786696251434
C	2.89270567272957	0.69053852948141	0.21069612156022
C	1.53993461820029	0.78352064401175	0.51343305082297
C	-1.21841238724244	4.01584810896649	1.38942901549860
C	-2.13368145229622	4.59041506398267	2.29520686155767
C	-1.99876683700008	5.93320631502201	2.63392082750812
C	-0.99077779827294	6.71250301811237	2.09049056771211
C	-0.10798532586820	6.14927297314180	1.18575707078830
C	-0.20679299805477	4.80887014185582	0.82032127473219
C	0.75363840891195	4.25599389785687	-0.20900199850113
C	2.20732288143702	4.38328046848201	0.24132721408671
C	0.54886496295649	4.92708972649220	-1.56837186467330
C	-3.28856023889966	3.80204362191350	2.87461491171576
C	-4.61804289030136	4.26687930232329	2.27770354512481
C	-3.32925003214208	3.86158720139503	4.40074080507997
C	-2.15560706133920	2.27329402286600	0.05127822983227
C	-2.40338314492336	0.87640746090527	0.07406390455921
C	-3.25052659064216	0.30473397891800	-0.85982590048905
C	-3.85968699429176	1.10524481013511	-1.83562498826756
C	-3.62441225739739	2.46765444542726	-1.85845771116510
C	-2.77657845426233	3.05818300028672	-0.91240346668292
H	-2.60895033634081	4.12859750028912	-0.92970981790145
H	-4.09732966576360	3.08972812304373	-2.60860477640214
H	-4.51694108046710	0.65043853489732	-2.56633000607490
H	-3.44741809280376	-0.76136405708056	-0.82711692143504
H	-3.15466863883390	2.75770462331326	2.59219883043442
H	-3.51635667807717	4.87295659289734	4.76674601711998
H	-2.39174900194249	3.51224490720100	4.83472259095135
H	-4.12940225886949	3.22530864534359	4.78493050414691
H	-5.44066370944723	3.65099658157623	2.64779219545436
H	-4.83472402235649	5.30368326214469	2.54445208469595
H	-4.60903857450293	4.19318577660361	1.18979131443552
H	-2.69841952963223	6.38306964040854	3.32929612782014
H	-0.90000038038968	7.75614677212548	2.36488596471250
H	0.67000057197313	6.76608667754947	0.75004741284580
H	0.53931813108850	3.19375236210946	-0.32287866482945
H	2.87531511133400	3.93908868694838	-0.49880565521611
H	2.50390980299510	5.42694822680731	0.36715368322086
H	2.37296276149729	3.86423987244599	1.18518208312094
H	1.19884816354734	4.47492710172381	-2.32017365505502
H	0.78294856637091	5.99327596036562	-1.53002607599556
H	-0.48038372028847	4.82310528901170	-1.91343344346157
H	1.76953240239531	1.76473328807113	3.73262537891544
H	4.16321776889737	1.61669073443425	3.20568414412574
H	4.89946059030763	0.91956044633881	0.94588256427899
H	3.20672661843932	0.38378928479059	-0.77993186255783
H	0.81282648990617	0.54619013942660	-0.25606894868193
H	-1.94940068304504	-0.71342825230839	1.25860350645793
O	-0.66687148339309	-0.14616428958376	3.35134500655538
Si	-1.65830928271090	-0.74817474861184	4.48648370777743
C	-3.37619540772217	-1.12713480764934	3.80767189713138
C	-3.78541157997696	-2.42594449739030	3.50385865144003
C	-5.01958388780826	-2.67446206689590	2.91342795939661
C	-5.86913998850083	-1.61986021421385	2.61179817172411
C	-5.48273894374692	-0.31789666402451	2.90760392102825
C	-4.25185208772429	-0.08120706140436	3.50079966736184
C	-1.86306697003158	0.43922560231239	5.93593233837690
C	-0.80806131776845	1.28021706600917	6.30104333672213
C	-0.91575558379686	2.14506655892994	7.38096165271159
C	-2.08950751024461	2.18604232643692	8.12330583363135
C	-3.14831302663821	1.35664723099374	7.78099589526842
C	-3.03158570599788	0.49357994435907	6.69803002459138
C	-0.91011756060613	-2.34939817435937	5.13249992411358
C	0.18918414993205	-2.93935668471920	4.50715099282874
C	0.73516533048551	-4.12588226008673	4.98228993375152
C	0.18667712134420	-4.74615198928497	6.09614719199831
C	-0.90784321341258	-4.17451218083715	6.73344938611523
C	-1.44544854908526	-2.98839921781391	6.25393663590770
H	-3.13412128771164	-3.26326029439357	3.72743428603474
H	-5.31551000796203	-3.69109481164516	2.68667157626327
H	-6.82881520891502	-1.80974612292454	2.14795922119887
H	-6.13936951119622	0.51088094288673	2.67422680956462
H	-3.97048199696072	0.94219742545732	3.72438331806525
H	0.11061538439682	1.27106682016437	5.72598597729050
H	-0.08693981630930	2.79145044451937	7.64128117239506
H	-2.17830947961979	2.86314537022268	8.96348880643317
H	-4.06662793916250	1.38443073602724	8.35411440764690
H	-3.87385380402845	-0.13946059476875	6.44212831616388
H	-2.29667900050301	-2.55523774917484	6.76897775912966
H	-1.33921910610378	-4.65231258266345	7.60414856582467
H	0.61071354256448	-5.67023440011367	6.46838032734240
H	1.58959350876152	-4.56554812007259	4.48292901311467
H	0.63035811516830	-2.46743339310920	3.63750977389546

Compound 2a-IV

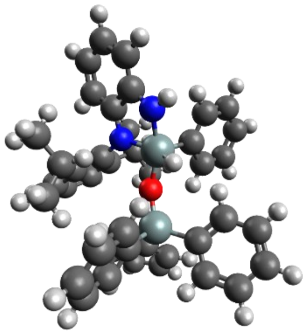

E = –2385.03694935 Eh

Si	-0.00896417215517	-0.00416173469969	-0.00290755859844
H	-0.01040134150087	-0.00389208989062	1.52862011367847
N	1.05358408337230	0.16098025046945	-1.51420018251838
N	0.81668064914947	-1.64527482495534	0.09027786360343
C	-1.77554421291551	-0.54090900147679	-0.55173416473592
C	-2.81112618362313	0.36974010545603	-0.79052665831257
C	-4.07734829848691	-0.03640594564210	-1.19203499279418
C	-4.35238413880622	-1.38528439419841	-1.37277680829049
C	-3.34692226352045	-2.31438752495041	-1.14552597783423
C	-2.08546113503317	-1.89218897108830	-0.74104996163821
C	1.40403941392942	1.32094455205188	-2.24227523404901
C	0.60340300345503	1.76905492428215	-3.30503217306003
C	1.03329192047243	2.84942574091134	-4.07133721192967
C	2.22930054612383	3.48844676890020	-3.80285568690184
C	3.00828662466979	3.05352238296992	-2.74366353979582
C	2.61738439583671	1.97699846900714	-1.95271414404505

C	3.52763164911312	1.52412833112582	-0.82923504604782
C	4.10694961318427	2.69128007908232	-0.03080355297795
C	4.66926664881503	0.65268762794376	-1.36025075464365
C	-0.71008509994081	1.10853154814732	-3.65804722907318
C	-0.63380431524998	0.39941007698747	-5.01110001348364
C	-1.86354536634658	2.11205604454277	-3.64349558229427
C	1.60131657315063	-1.02426626405047	-1.96579710433243
C	1.46075354674627	-2.07510777824706	-1.02324274060189
C	1.97617666664227	-3.33291123369998	-1.30962574090115
C	2.63204378406819	-3.56179823910336	-2.52401991911452
C	2.76604202991206	-2.53900861015268	-3.44522633079154
C	2.25301434772344	-1.26552636774080	-3.16517667125935
H	2.37776504698355	-0.46773262248112	-3.88707022381685
H	3.27062970967612	-2.71333066794694	-4.38742443446191
H	3.02887752639366	-4.54648952967301	-2.73924366103633
H	1.87383239076181	-4.13322407592034	-0.58484626118904
H	-0.92143489655108	0.35382285621418	-2.90183057140871
H	-1.76283657552857	2.85755687965051	-4.43518180510188
H	-1.91893917503340	2.64285726591227	-2.69263193233664
H	-2.81526253321314	1.59941990227319	-3.79426524402799
H	-1.57420604940923	-0.11052608696475	-5.22980219298971
H	-0.44463129006314	1.10490095442899	-5.82335267267594
H	0.16023619717145	-0.34775772365354	-5.02416610676619
H	0.42116412521018	3.19575339199090	-4.89633493761680
H	2.55262183809448	4.32384606859535	-4.41156753483181
H	3.94282688521958	3.56053070622519	-2.53591750966231
H	2.93471418627512	0.91505370867750	-0.14344788017759
H	4.63480270474070	2.31595002104576	0.84703096923561
H	4.82667068444486	3.26842296111476	-0.61461755152063
H	3.33065358290493	3.37110890430066	0.31890891484361
H	5.29596191217101	0.29948566903363	-0.53855632040240
H	5.30610298061380	1.22363428083865	-2.04036576245677
H	4.29760452558148	-0.21809365478580	-1.89753797720208
H	-2.63369822232448	1.43056836258364	-0.66909600642890
H	-4.84998158390415	0.70305194102491	-1.36598626314973
H	-5.33755345603367	-1.70760263553811	-1.68669080387206
H	-3.54385328872422	-3.37090623585919	-1.28339611352118
H	-1.32078947607633	-2.63995600137232	-0.57147163895415
H	0.69237608166399	-2.29751629775137	0.84791898737523
O	-0.24493565757727	1.73026697507249	-0.01529003472543
Si	-0.52289037949639	3.06076927872051	0.86785302922744
C	-1.01625845519681	4.44076137739442	-0.31262731784540
C	-0.09615781966322	4.88310544422125	-1.26827908430467
C	-0.43937304785757	5.84404014427477	-2.20822407204476
C	-1.71934639031312	6.38377476870389	-2.21417277191518
C	-2.65061401879820	5.95410910668662	-1.27912879101565
C	-2.29850313332303	4.99196012266703	-0.34018153512898
C	1.00758910246129	3.58512797997067	1.83334010212839
C	1.68506196339867	2.63274127410292	2.60122913823373
C	2.77728900181251	2.98146526101093	3.38147696001877
C	3.21893484709479	4.29866160592008	3.40872154839306
C	2.56011736261652	5.25978291547827	2.65554402596298
C	1.46352919590905	4.90294380959414	1.87904059856375
C	-1.87074555796376	2.87018637463550	2.17530874651289
C	-2.20195386709530	3.97890697683679	2.96201646168562
C	-3.15841717833938	3.89744137656409	3.96355057751502
C	-3.80463550309927	2.69147761777604	4.20679108197489
C	-3.48438266909547	1.57688847356834	3.44547195204328
C	-2.52717524669463	1.66867286186004	2.44150281503915
H	0.90280543028102	4.46076227874595	-1.29778398052500
H	0.29006204017030	6.16485005741172	-2.94130314287158
H	-1.99073600711236	7.13241924022222	-2.94784527788318
H	-3.65229076161236	6.36537223792984	-1.28220618519139
H	-3.04280041881370	4.66669621730773	0.37733091445999
H	1.35714315544558	1.59845446991785	2.58895941634808
H	3.28798426281179	2.22651448416948	3.96620353645472
H	4.07314522672171	4.57358327325029	4.01459253298879
H	2.89930310676901	6.28808137506854	2.67193514656804
H	0.96215874457185	5.66907938536890	1.29870940620845
H	-2.29106766052716	0.78442979994180	1.86460271495347
H	-3.97908299683495	0.63193007842724	3.63226744373571
H	-4.55061337553293	2.62184399607262	4.98842442252518
H	-3.39830605143505	4.77161609466326	4.55579015418559
H	-1.70332004351299	4.92861100249841	2.79591900462078

Compound 2b-I

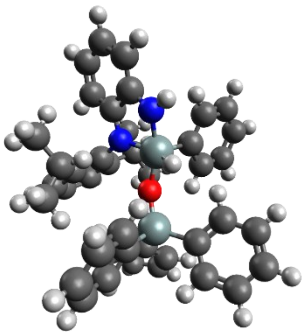

E = –2385.03985244 Eh

Si	0.00031749559939	0.01796421664862	1.54260762033000
H	0.00639224887665	0.01545796405735	-0.00495033517844
N	-0.27214048469139	0.73899162568389	3.26366226345276
N	-1.79586766075407	0.24312324769166	1.43842325055333
C	0.26572897774051	-1.84599269593314	1.86000871716440
C	1.49092412636265	-2.47816028176067	1.63860274769951
C	1.66033432926270	-3.84403179655127	1.83114938958177
C	0.59638042327770	-4.62241330887123	2.26593891776868
C	-0.63418817756226	-4.02020654780052	2.49658458523193
C	-0.79168604138019	-2.65553596871792	2.28852366349508
C	0.66384176628999	1.33104264326170	4.13863550502400
C	1.38217924595883	0.54928760871669	5.05765035897413
C	2.24891082128767	1.17157774755815	5.95386555632137
C	2.40208530320162	2.54582849867345	5.96589646085163
C	1.69217389767592	3.31400198721508	5.05781928656573
C	0.82891335693069	2.73131966536639	4.13563492948479
C	0.09362865448490	3.62751957830518	3.16175827170094
C	1.03364575034386	4.61515000483859	2.47015172164195
C	-1.04399688283770	4.38361984978266	3.85147317327935
C	1.23472658164073	-0.95330311865612	5.11812252604597
C	0.60909232726141	-1.40041086618841	6.43974722416187
C	2.57189204945655	-1.65445399998533	4.88291349322113
C	-1.58957693769323	0.79137869241387	3.65481471567853
C	-2.47906088165592	0.50646063511268	2.58736639209950
C	-3.84909896627415	0.53162729580289	2.79809629362801
C	-4.35675158387147	0.83548023669723	4.06684612941846
C	-3.49253201415993	1.11019465696425	5.11097536912876
C	-2.10690599044695	1.09243413270339	4.90714759901994
H	-1.43998958055969	1.31817945373942	5.73053402877224
H	-3.88129341728420	1.34167936643331	6.09500486357166
H	-5.42812653248626	0.84963373714956	4.22492035740418
H	-4.52420372738380	0.31970832229168	1.97618371797069
H	0.56250025993070	-1.25499070946358	4.31744059208250
H	3.28618796566112	-1.44507267712951	5.68224981158582
H	3.02083561514902	-1.34009822508441	3.94023647486068
H	2.43373473499531	-2.73609462439896	4.83736243200017
H	0.45413673986139	-2.48123117226762	6.44373070991979
H	1.24659873214738	-1.15561211678841	7.29223473663322
H	-0.35973321588715	-0.92525987504618	6.59967570840452
H	2.80450031743656	0.56936688822619	6.66398457732460
H	3.07140564420698	3.01671620308570	6.67521616899045
H	1.81895328600567	4.39025710870670	5.06662234483474
H	-0.34472857406451	2.99353437853236	2.38914293460025
H	0.50518109229214	5.14778349202519	1.67777191676966
H	1.42060925385162	5.36434752346150	3.16363857714712
H	1.88320638547918	4.10829572755897	2.01317197231821
H	-1.59384462657360	4.99187373028380	3.12979882739719
H	-0.65652739062079	5.05465267307720	4.62149198707415
H	-1.75036085989692	3.70081879870321	4.32185227932587
H	2.34182244792025	-1.89696095637497	1.30669671040562
H	2.62560570188344	-4.29997199787908	1.64511725522799
H	0.72309913088998	-5.68664778566354	2.42178459726155
H	-1.47324672682905	-4.61598893436515	2.83585931213671
H	-1.76462041962309	-2.21317998578897	2.47019168699034
H	-2.32383504318708	0.04893249271964	0.60300223468551
O	1.60233195767867	0.69362601887261	1.56746394865032
Si	2.63784172106676	1.23005394351520	0.42393868263730
C	1.97210183436449	2.75261665117073	-0.47472045213527
C	2.85518703682388	3.69964056648997	-1.00034517925755
C	2.39712528109553	4.82649636073107	-1.66959274668741
C	1.03340532510809	5.03134735996522	-1.83021249452764
C	0.13781463359381	4.10290484777291	-1.31834551271244
C	0.60457248538059	2.97924346948676	-0.64886936537820
C	3.07278205813552	-0.04861302506180	-0.89235171201206
C	2.43188079507691	-0.06466081992930	-2.13274773406750
C	2.72948990490576	-1.02885531046035	-3.08700985463463
C	3.68185524289402	-2.00222870849984	-2.81658004359270
C	4.33479631458106	-2.00366219042747	-1.59099892515329
C	4.03182356296168	-1.03451731686167	-0.64429810667173
C	4.23290907527932	1.67886355074965	1.31586926995322
C	5.44546994341000	1.82228978909985	0.63538536295853
C	6.61013460078179	2.17782084915607	1.30237657286554
C	6.58464298719205	2.39207114345688	2.67453759646282
C	5.39218802701977	2.24772029670747	3.37026455834105
C	4.22992918671021	1.89485199554262	2.69619883525203
H	3.92483343133177	3.57101821407941	-0.87999637512501
H	3.10453657145454	5.54629913794964	-2.06190262712950
H	0.67145823045469	5.90999538491369	-2.34905054946227
H	-0.92780800242829	4.25463563957463	-1.43657471761929
H	-0.11929057623401	2.27435155240946	-0.25850744696456
H	1.68655973351607	0.68762469332370	-2.36408609004269
H	2.21828682479982	-1.02104140488601	-4.04147671761924
H	3.91618596061727	-2.75503475150005	-3.55860967596538
H	5.08051642202739	-2.75805284103851	-1.37377149087524
H	4.55699155428216	-1.05067158993116	0.30458212795555
H	3.31070666240769	1.78605516111546	3.25828078866477
H	5.36367752822016	2.40784664748884	4.44101419792066
H	7.49156643227525	2.66619045916283	3.19869936254527
H	7.53758371682969	2.28252654483218	0.75343698773397
H	5.49107460874666	1.64512914225609	-0.43383728291291

Compound 2b-II

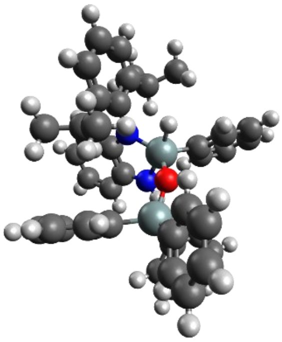

E = –2385.04493783 Eh

Si	0.00197880762673	0.00051874586426	0.00150769679542
H	0.01444850883941	0.00273598825010	1.54713909298273
N	-0.77870729593853	1.64812132079897	0.06146061752809
N	-0.24127493862806	0.24345777523147	-1.83670397662848
C	1.90618218189900	-0.26406690932349	-0.09267453911358
C	2.55137913202717	-0.88376397852675	0.98108174192791
C	3.92255965476463	-1.10447892041582	0.99097444221882
C	4.69865947341733	-0.67942279068661	-0.08040857138921
C	4.08804269033669	-0.04601843962049	-1.15454024247970
C	2.71065908313418	0.14699872586398	-1.15852769293289
C	-1.01559479773364	2.35847822860225	1.25545571214948
C	-2.22746887697390	2.18532237363243	1.94117453143698
C	-2.43607330696978	2.88736327110172	3.12524172633246
C	-1.47599789896708	3.75288134179776	3.62192353250863
C	-0.28697011506521	3.92493081083371	2.93274132629562
C	-0.03893616647969	3.23908415846531	1.74733580318733
C	1.27842685702342	3.44569048541679	1.03272093825857
C	2.44762908688208	2.94672156673232	1.88145613823816
C	1.47685843313863	4.90577951023143	0.62641560915289
C	-3.29845757164088	1.24255447612633	1.43927207496394
C	-4.62660310200696	1.96349591628767	1.21166952043332
C	-3.47010455636952	0.05782125156041	2.38989438730168
C	-1.10578830773823	2.25051362531205	-1.13487525016609
C	-0.77410341900263	1.42237583031432	-2.23723999438513
C	-1.03087596725277	1.87343164856356	-3.52719894477667
C	-1.61523834949657	3.12908438345670	-3.72743305840242
C	-1.94350898576528	3.93016035865571	-2.64885191706232
C	-1.68562165017503	3.48964600568381	-1.34257989057738
H	-1.93953789331868	4.12044350252099	-0.49895354797178
H	-2.39771327360032	4.90055465904402	-2.80505761718673
H	-1.80933867324444	3.46971235305772	-4.73735319068216
H	-0.77865352771815	1.24700161701105	-4.37608531638859
H	-2.97512362086534	0.84391789520898	0.47719734393290
H	-3.80928704032276	0.38052227547817	3.37659619639932
H	-2.53023472512741	-0.48078875957938	2.51671653810108
H	-4.20762228142999	-0.64480403827039	1.99790156686604
H	-5.36099920819722	1.27760148967451	0.78582504035481
H	-5.04287357339127	2.35686875022981	2.14134338177465
H	-4.50909714507344	2.79701513219859	0.51770813681384
H	-3.36360347267970	2.75506822665342	3.67068386981121
H	-1.65336129806865	4.29132264115571	4.54456148559038
H	0.46229847115162	4.60178638350696	3.32754557437955
H	1.26421999909735	2.85000224473027	0.11979307315120
H	3.38678257154794	3.03685130686373	1.33195273089948
H	2.54775544437676	3.51992626608637	2.80564307866725
H	2.31850525001499	1.89698538223451	2.14635045940291
H	2.40054001454299	5.02072711414372	0.05619745484306
H	1.54416556223742	5.56357476951372	1.49536953927099
H	0.65421503317326	5.25729553513992	0.00232124954913
H	1.96165604433137	-1.19380142275840	1.83807708151588
H	4.38853068590185	-1.59872892610594	1.83508951212723
H	5.77011698454937	-0.83739932873735	-0.07583345855285
H	4.68546216815494	0.29598563632740	-1.99129040665404
H	2.25403132590455	0.63437631042005	-2.01169684459361
H	0.08454176864054	-0.37768769599425	-2.56063277823577
O	-0.78657595027122	-1.53834370401648	0.00559467489247
Si	-1.73802515815017	-2.40746641905516	-0.99896447595283
C	-3.35042621670692	-1.55635857133417	-1.46767762549261
C	-3.42476481090640	-0.64689592482679	-2.52577072056822
C	-4.60514784825645	0.01977527087744	-2.82323220213871
C	-5.74594354177058	-0.21699831110406	-2.06752675186402
C	-5.69841976248405	-1.12422922426211	-1.01790801640940
C	-4.51214167845957	-1.78505478937346	-0.72573806240318
C	-2.19681608018775	-3.96798157026691	-0.04575711395498
C	-1.98532351980668	-4.07031621730629	1.33023000836034
C	-2.34903203638702	-5.21412162389284	2.03116042549194
C	-2.93285732484985	-6.28206919398369	1.36440538188082
C	-3.15103380455068	-6.20077276848014	-0.00541946393175
C	-2.78528042642881	-5.05490664676069	-0.69754001329660
C	-0.82238970028733	-2.93392491327759	-2.55717459282221
C	0.56995498252189	-3.06102502735285	-2.53687657145339
C	1.27062635598836	-3.48499682056734	-3.65767249824074
C	0.58823671274431	-3.79779300403229	-4.82638673033323
C	-0.79459085155273	-3.68354629362851	-4.86621656423993
C	-1.48875224228997	-3.25455822050298	-3.74179348485731
H	-2.54854209064816	-0.44460781599509	-3.12698529619166
H	-4.63191495706980	0.72689000616890	-3.64285187807606
H	-6.66794009030830	0.30273242225673	-2.29592327216090
H	-6.58438028960245	-1.31677740161314	-0.42585718762036
H	-4.49750324050431	-2.48974825764224	0.09782099990050
H	-1.52918263782201	-3.24777499358741	1.86766031439775
H	-2.17505765955505	-5.27128924920915	3.09845971148656
H	-3.21565689710833	-7.17460597691583	1.90814928331173
H	-3.60423033213538	-7.03082325087396	-0.53281964293697
H	-2.96212449051677	-5.01437246443584	-1.76726025722604
H	-2.56776582115236	-3.16341169743723	-3.79754476250268
H	-1.33283125893397	-3.92375838103805	-5.77452260557666
H	1.13237788365272	-4.12701850859263	-5.70252939125758
H	2.34945657269609	-3.56908317475134	-3.61971370772377
H	1.12284401762665	-2.81683336315058	-1.63667257747391

Compound 2c-I

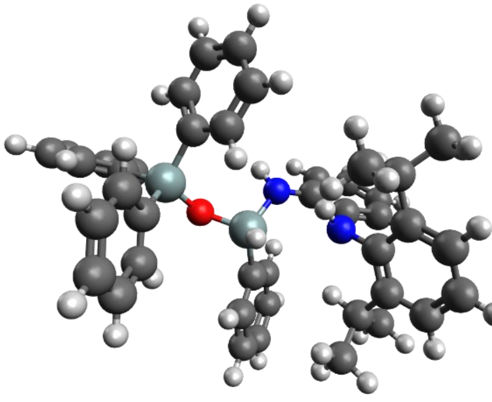

E = –2386.21481587218 Eh

Si	-0.12886900357306	-0.05736556156928	-0.67056848694766
H	0.49352962066307	-1.34895044947089	-0.28169262039851
N	-0.09933035498552	-0.49943519247854	2.34352950804874
N	-1.60469490495663	0.30534325487198	0.19499206225516
C	1.13335780086984	1.30341957714647	-0.55545463015785
C	2.46998802910823	1.03271177776502	-0.85498773211060
C	3.42784480015419	2.03556875701380	-0.81599915195957
C	3.06245019154361	3.32689017249809	-0.45926470887891
C	1.73856171185938	3.61279744385091	-0.15219719297246
C	0.78142946559427	2.60872661599207	-0.20623212425836
C	0.64027250603876	-0.99059834853077	3.45006251935171
C	0.14806002113565	-2.08188136642517	4.18691383395794
C	0.90462205184814	-2.55818447723219	5.25162021488529
C	2.09616071367300	-1.94765384809127	5.60861824220744
C	2.55615271923562	-0.86171703101894	4.88595937039114
C	1.84973660736117	-0.37189541617432	3.79046756222918
C	2.42165074402722	0.78477869859317	3.00035671796695
C	3.79925649686308	0.42952639108556	2.43990775170242
C	2.49291074801806	2.06211489717071	3.83754104817186
C	-1.15436592195381	-2.75662525512019	3.80654760971986
C	-1.93161849824371	-3.29552277604783	5.00466028817671
C	-0.89695622787495	-3.87562044999318	2.79276476099404
C	-0.98339242196074	0.57265858500599	2.52301392120869
C	-1.75480765122356	0.96692414504830	1.41472381889009
C	-2.67248814160543	2.00105514235689	1.56744346873523
C	-2.81689614376993	2.66434070711713	2.77893212406313
C	-2.02992988074299	2.29683946691679	3.85736594204942
C	-1.12679661935395	1.24839514817557	3.72924340528198
H	-0.54039022101649	0.94298097648488	4.58591154657626
H	-2.12403309258885	2.80719686323449	4.80681354959562
H	-3.53291021973724	3.47071693539915	2.86790624295401
H	-3.27152227978627	2.29829323446673	0.71420355031937
H	-1.80003218326533	-2.01276023192879	3.33151017931678
H	-0.26464315168278	-4.65114327106832	3.22885835893525
H	-0.39604760547272	-3.51278327325581	1.89276562191284
H	-1.83444123135388	-4.34151391706495	2.48427817767565
H	-2.91188500136189	-3.64639938298426	4.67999163069262
H	-1.42970079712668	-4.14026588066573	5.47856565465481
H	-2.08613170331870	-2.52598720919077	5.76157625682210
H	0.55632177295170	-3.40737797983228	5.82521441770922
H	2.66532263586669	-2.32095068464221	6.45033163560249
H	3.49039668103586	-0.39284652301216	5.17117497659697
H	1.76626310845741	0.97916798641379	2.15114604503882
H	4.18450186547815	1.25074361977113	1.83495741492588
H	4.52194570887159	0.23507044696638	3.23457012170192
H	3.75256078225070	-0.45558512011318	1.80426660400182
H	2.85658997998480	2.89236888819481	3.22947629032715
H	3.17301803227721	1.95021020293698	4.68436338718472
H	1.51395902147101	2.34124882810755	4.22787646574991
H	2.77482325330908	0.02528232730802	-1.11848731848875
H	4.45950642296003	1.80945255425535	-1.05329425270479
H	3.80909461555999	4.10969765872262	-0.41928858961538
H	1.45194095969506	4.61862759620061	0.12693196351823
H	-0.24722451645260	2.85108545421718	0.03192354409983
H	-2.39983518774700	0.43827699793704	-0.41648259974182
O	-0.62293304755128	-0.20119022483314	-2.25090429673486
Si	-1.16997386249585	-1.47329052784281	-3.17094947529669
C	-2.57937593507550	-2.33104311557770	-2.28367134473328
C	-3.90258500784924	-2.17871052908157	-2.70141208903745
C	-4.94733422826708	-2.77317561468829	-2.00500306145100
C	-4.68285005461100	-3.53160105720834	-0.87351721857023

C	-3.37251924399514	-3.69673809188091	-0.44239889709964
C	-2.33337873160723	-3.10219070815726	-1.14278048194231
C	0.23795773444992	-2.67561967978761	-3.43947204468295
C	1.55893823312498	-2.22150870150140	-3.48820594682498
C	2.60916758259984	-3.09911539664073	-3.71668861950286
C	2.35469998781804	-4.45128541173578	-3.90672786394618
C	1.04919381365390	-4.92086954529528	-3.86533105724479
C	0.00245614411048	-4.03880362786545	-3.63097979157111
C	-1.76384108500780	-0.74781539786825	-4.78666309950372
C	-2.24749811134307	0.56060163267052	-4.85896716320136
C	-2.72608700745187	1.07968203710215	-6.05438275033757
C	-2.72984201570497	0.29522900155740	-7.20002480665534
C	-2.25199575576009	-1.00742521973137	-7.14698638599551
C	-1.77247026825491	-1.52067557654962	-5.94983695051508
H	-4.12730682555345	-1.59071259940139	-3.58371641886697
H	-5.96658230430011	-2.64463025787773	-2.34610260140888
H	-5.49514239965765	-3.99607727194662	-0.32922377436132
H	-3.16167374495615	-4.29042721146205	0.43784797853717
H	-1.31709026181306	-3.25140480228161	-0.79297704346549
H	1.78026643223867	-1.16990218759225	-3.34427217690473
H	3.62596726560802	-2.72895566839608	-3.74666735671211
H	3.17282941108738	-5.13739530854851	-4.08476263397558
H	0.84630664314300	-5.97417455742707	-4.01064832917307
H	-1.00963163564749	-4.42553702927989	-3.59192827124142
H	-1.39534662363567	-2.53727189765978	-5.93022122406049
H	-2.24910771589118	-1.62135453647582	-8.03851049364687
H	-3.10185869734255	0.69920386359735	-8.13288868998145
H	-3.09480727220071	2.09677552630367	-6.09244389150435
H	-2.25066614003675	1.18957516389902	-3.97667996967665
H	-0.52466137883220	-1.23815317584970	1.79878984332344

Compound 2c-II

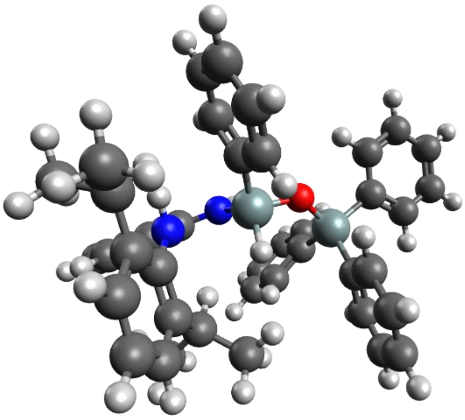

E = –2386.22101538085 Eh

Si	-0.05087327962174	0.06595607952698	-0.41318831335527
H	-0.96250003150127	-0.91424867445316	0.21803299725795
N	0.58604241794227	0.20954587472548	2.08592175729117
N	-0.15126439997282	1.76016844834021	0.03638525532245
C	1.68191525880900	-0.62143353148115	-0.52583768346570
C	1.87288275717697	-2.00418083317477	-0.50965420971210
C	3.14851532520420	-2.55074892732965	-0.55381487933304
C	4.25767237936527	-1.71709816265513	-0.60886363529715
C	4.08579853057793	-0.33892695036152	-0.62715417651283
C	2.80730572807444	0.20196439399475	-0.58751471212075
C	0.19453047378974	-0.83959289713669	2.95303903148615
C	-1.16400652134741	-1.04318049892547	3.24794435537651
C	-1.49787099621006	-2.06728045547505	4.13053655743127
C	-0.53877787490817	-2.90144743291266	4.67187216005257
C	0.78971009339697	-2.72550010688718	4.32237339381362
C	1.18340990816860	-1.70276628416105	3.46843063557168
C	2.65731828955768	-1.51040128697863	3.14762612152463
C	3.47838352531868	-2.79277790885497	3.24770732361575
C	3.26838731995621	-0.42436167834184	4.03760943612055
C	-2.29592047107676	-0.22463607676789	2.65374117787838
C	-2.97869739502712	0.64338176726329	3.71108001970341
C	-3.32335887850626	-1.11715944422123	1.95345113815847
C	0.23662532821435	1.55772838871093	2.38922539341306
C	-0.11958850317130	2.33933130327284	1.28430399366863
C	-0.44998577264004	3.67951743904205	1.49802142899730
C	-0.42878434383459	4.21249908881694	2.77791603143081
C	-0.07965198959998	3.42800239885913	3.86883152090883
C	0.25628322853992	2.09559812115054	3.66415867757860
H	0.52519493111571	1.46520494545933	4.50348840422432
H	-0.06718549830388	3.84509624271009	4.86662782903946
H	-0.69460821438468	5.25203467535844	2.92141394338170
H	-0.73798848508090	4.29888491864140	0.65662349887704
H	-1.89688590176048	0.44366692183504	1.89200315672366
H	-3.83710397453059	-1.77776233116343	2.65326872817065
H	-2.85982529726190	-1.73686153415785	1.18505768900458
H	-4.08599718266802	-0.50344710433174	1.47019922214013
H	-3.75936890849237	1.25485413199315	3.25456117670897
H	-3.44839729373854	0.03093385333847	4.48312745155187
H	-2.27111773012201	1.31405498673090	4.19736114875336
H	-2.53844119189615	-2.22457215287614	4.38761339466672
H	-0.82349367795742	-3.69432746669947	5.35117257844424
H	1.53165602994015	-3.39614816227337	4.73429385170779

H	2.75999776395535	-1.19602602342920	2.10336304085251
H	4.48330999724277	-2.61039005854715	2.86584296437890
H	3.58320241724073	-3.13969256382496	4.27679676579444
H	3.04045230981947	-3.59907707030918	2.65853825088318
H	4.31508160790349	-0.25777102379103	3.77800517211353
H	3.22476441925797	-0.72237623771011	5.08660427264796
H	2.75035491430808	0.53070916642809	3.94383140246620
H	1.01774633681338	-2.66921537471648	-0.45130825085283
H	3.27826636854587	-3.62540852176340	-0.53654449765292
H	5.25370020473705	-2.14014074959209	-0.63620908217673
H	4.94829563156048	0.31403538704662	-0.67009120509181
H	2.68856930160401	1.27973660548429	-0.59333341432953
H	-0.47717270048305	2.38373317952597	-0.68888901705335
O	-0.58275855515181	0.15722965440345	-2.00919038514399
Si	-2.08966936619661	0.29081298552163	-2.68266349761647
C	-2.90826429588247	1.87552140867531	-2.10027100260979
C	-2.97828757536488	3.00578690752066	-2.91602887690633
C	-3.50636058667322	4.20087533639168	-2.44251867765654
C	-3.97232840479095	4.28464697686270	-1.13808522071396
C	-3.91378920339019	3.17019297688176	-0.30994899251675
C	-3.38837321340384	1.98030614344899	-0.79064746614403
C	-3.13645342219420	-1.17419692427081	-2.16119108186859
C	-2.54563612333990	-2.42519264973931	-1.96067688040745
C	-3.30347571488374	-3.52374243143158	-1.58192714971215
C	-4.67442738630090	-3.39157656858817	-1.39981389257612
C	-5.28130119475529	-2.16008057424661	-1.60189293102566
C	-4.51617170518513	-1.06337900632878	-1.97815100892727
C	-1.86110618935413	0.32866306492359	-4.53785428749147
C	-0.61997482193352	0.59166767755984	-5.12011650475862
C	-0.47500830812437	0.64089745233584	-6.50070112974457
C	-1.57316921318697	0.42888980786970	-7.32269722940075
C	-2.81599246561135	0.16553374280111	-6.76083391020747
C	-2.95438382723927	0.11521525616607	-5.38119546049634
H	-2.61690608600038	2.95944191829970	-3.93691465096106
H	-3.55274723569660	5.06595357017250	-3.09161808997385
H	-4.38222766998196	5.21522409470622	-0.76670607784328
H	-4.27578927663511	3.22893837289962	0.70864435338276
H	-3.35351832669357	1.12207825203222	-0.12826932657232
H	-1.47694047035972	-2.55016622115471	-2.09393969689948
H	-2.82556025073074	-4.48249468847237	-1.42617411022034
H	-5.26713796977673	-4.24670746948526	-1.10103770887653
H	-6.34924172053291	-2.05159771554542	-1.46197167128172
H	-5.00754433784055	-0.10777614996862	-2.12116550841686
H	-3.93150624696159	-0.09888640947686	-4.96136948407015
H	-3.67486613817001	-0.00361070872728	-7.39765426384908
H	-1.46110834910820	0.46626878540011	-8.39875848102515
H	0.49580130228924	0.84370593753138	-6.93473643063335
H	0.24915335433454	0.75786483790525	-4.49534272989632
H	1.57617471078786	0.15890156417516	1.88726018980192

Compound 2c-III

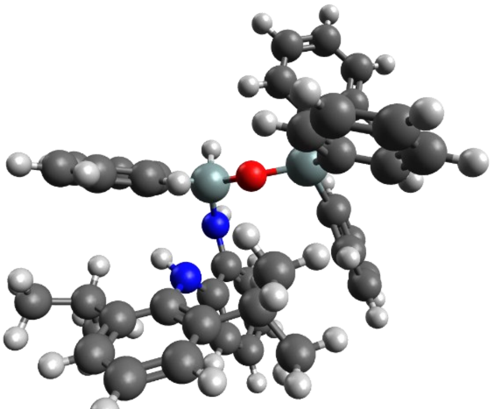

E = –2386.21827947604 Eh

Si	0.22290327224461	0.46878055228544	-1.02662718330455
H	0.63041306727677	0.81975822103634	-2.41276413614483
N	0.34623330225501	0.45649421805001	2.07930885507399
N	0.51970253906975	1.99530546059358	-0.23425243892552
C	1.27894172736972	-0.96920325498617	-0.47633212371465
C	0.75304639753684	-2.11278116566552	0.12802741347074
C	1.57973863096720	-3.15915249176392	0.51143747515321
C	2.94918185694919	-3.08033166810113	0.29320531348272
C	3.48935242714259	-1.95346496169847	-0.31129793610664
C	2.65773053801840	-0.90918919923050	-0.69317239853977
C	0.20924128354351	-0.40304997200067	3.19007179746696
C	-1.07230872020667	-0.74642480993999	3.64451427832819
C	-1.18222344396647	-1.58166895888598	4.75288830622178
C	-0.06314767841907	-2.10139133263551	5.37557600799657
C	1.19618018151584	-1.79302809267915	4.88628889073208
C	1.35985307448696	-0.94016640335585	3.80140130657469
C	2.75031666253955	-0.57461214742509	3.31447746109694
C	3.82210353377518	-1.59287536377736	3.69036790153824
C	3.14884483421947	0.81609679632708	3.81660915126884
C	-2.33920009633337	-0.29792093165652	2.94602144521467
C	-3.25914482408259	0.49771607658913	3.87176228595485
C	-3.07618075773093	-1.50660338456186	2.36763337736230
C	0.03279464662846	1.81953104096348	2.15607467067755

C	0.12537565851486	2.57374960128804	0.97262680815062
C	-0.15040396589450	3.93813719586070	1.02996560435794
C	-0.52902187044551	4.55442691176143	2.21318390909640
C	-0.63732092165209	3.80335783984476	3.37163183147871
C	-0.34461875174514	2.44683016578257	3.33809204840979
H	-0.39498611037588	1.86763951445733	4.25097066335691
H	-0.93034422041343	4.26573860181002	4.30493978177932
H	-0.74801343361021	5.61404239020524	2.21912662787570
H	-0.08277850498653	4.52253152891557	0.11951166603668
H	-2.07169121561205	0.34624274959239	2.10767800956681
H	-3.41211490018539	-2.18579356611588	3.15331050411493
H	-2.43891417892794	-2.07053156528262	1.68610363288444
H	-3.95548199016491	-1.18909479748448	1.80442710030158
H	-4.14325377869038	0.83811619879519	3.32947883769010
H	-3.60749799135564	-0.10873516931497	4.70968885067007
H	-2.76131257457588	1.37797981427974	4.27776475934282
H	-2.16531735284342	-1.84829933524611	5.12241565541280
H	-0.16855783505711	-2.75588182992887	6.23117765723706
H	2.06368978349201	-2.22034820254482	5.37085848815219
H	2.74657038297147	-0.55246526364550	2.21915306277927
H	4.75805250509098	-1.33621484298543	3.19303295521909
H	4.02114691988940	-1.60652927178518	4.76327300447246
H	3.54840069358408	-2.60216188757662	3.38123265081212
H	4.12575128235199	1.10186110264155	3.42282436775424
H	3.21202916140224	0.82213221221049	4.90638956736167
H	2.43457280702310	1.58458407512226	3.52200794778051
H	-0.31127271547140	-2.18781760199089	0.31504317861799
H	1.15588225464375	-4.03592213932418	0.98401923919883
H	3.59408309147738	-3.89589491183361	0.59439077299941
H	4.55575959865378	-1.88797422636855	-0.48496097966070
H	3.09646818388808	-0.03686805086239	-1.16606422279698
H	0.72357799291538	2.70672613543698	-0.92401183484180
O	-1.34971317197041	-0.03852356935844	-0.98839224404478
Si	-2.82379290435648	0.44837955775553	-1.58236956859057
C	-3.31958526730855	2.08542266683876	-0.82603374621861
C	-3.03587568155187	3.29798184888975	-1.45940994747886
C	-3.34937389591133	4.51017344150724	-0.86014608198241
C	-3.94719233102904	4.53132638260547	0.39229545130784
C	-4.22588827205644	3.33793031556641	1.04450860676157
C	-3.91538732277493	2.12963258360697	0.43729543780929
C	-4.03763253241136	-0.90401247945847	-1.14300953355821
C	-3.61336225633969	-2.23272579017081	-1.06598197408498
C	-4.51191221752801	-3.25641486120675	-0.80302559872969
C	-5.85716431918974	-2.96806550645234	-0.61311315023906
C	-6.29932406466439	-1.65489138949695	-0.69599557603358
C	-5.39572517737406	-0.63480574928408	-0.96302941806558
C	-2.72910979179216	0.58556598089003	-3.45039075499585
C	-1.77063681130861	-0.11531042273594	-4.18580631617034
C	-1.75904896721420	-0.07391089191789	-5.57356475278960
C	-2.71486804260827	0.66732988714709	-6.25443433090988
C	-3.68419097150849	1.36112331732225	-5.54234774415733
C	-3.68946758707022	1.31683859026662	-4.15523839878530
H	-2.56575160473154	3.30468828963828	-2.43602068727934
H	-3.12532214957534	5.43842790372369	-1.36996154037776
H	-4.19110136615917	5.47586588563968	0.86142284309715
H	-4.68584761783765	3.34722639606954	2.02440520609534
H	-4.15033259428097	1.20875125538461	0.95852919743566
H	-2.56686769373497	-2.47950016390245	-1.20242224911010
H	-4.16283323413185	-4.27935523015124	-0.74216130434837
H	-6.55897801712125	-3.76508983188118	-0.40320856351332
H	-7.34721104539920	-1.42462840513702	-0.55195517878008
H	-5.76179153629847	0.38387170506285	-1.02451997581220
H	-4.45786377223997	1.86371463019295	-3.62000781732701
H	-4.43571417143139	1.93687253285306	-6.06723566723917
H	-2.70665409977461	0.70176376598839	-7.33631722392092
H	-1.00434066364734	-0.62189271538024	-6.12308588535584
H	-1.01889658610615	-0.70949778654860	-3.68035424742869
H	1.21419727974727	0.30650625293768	1.58561113836124

Compound 2c-IV

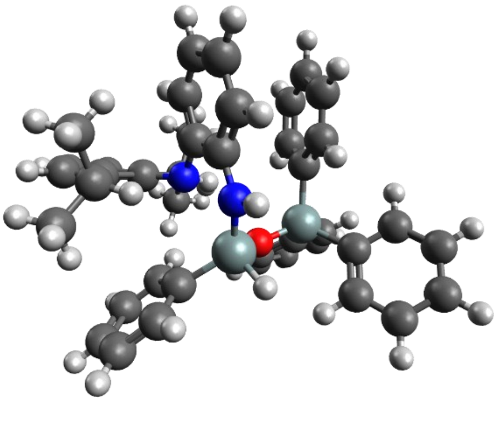

E = –2386.21976471495 Eh

Si	0.36977059917234	0.11880786674102	-0.94490215854164
H	0.89016613619066	0.22793534367801	-2.33867889755087
N	-0.43559079112499	0.51990249016969	1.80094815446895
N	0.74080656092614	1.73585821320814	-0.40606205915299
C	1.22610236341507	-1.38366210039479	-0.24162908704259
C	0.59901751031776	-2.31078525967318	0.59186562076787
C	1.25136523705986	-3.46737345944095	0.99362007939257
C	2.54729799255337	-3.72178969457020	0.56245177550145
C	3.18592514474957	-2.81485835761325	-0.27163688897386
C	2.52625712584872	-1.65943555093532	-0.66928518132867
C	-0.20285395473914	-0.20912487024911	2.98870350805253
C	-1.24754597561710	-0.92885422202812	3.59840889103972
C	-0.98865234229735	-1.59564303681409	4.79239448357998
C	0.26212636460379	-1.56380509371349	5.38152388143199
C	1.29074114389038	-0.89154225685524	4.74478731684899
C	1.09309760983969	-0.23181980644549	3.53744764590765
C	2.29064853764414	0.40039124001183	2.85594487345904
C	3.43961295779844	-0.59885938912906	2.71270491713922
C	2.77376955321384	1.65254410180923	3.58895551613214
C	-2.65962877594445	-0.97144282633578	3.03983147788654
C	-3.55163323607921	0.03872753021491	3.76166192953813
C	-3.27166874012132	-2.37043679015277	3.08339520300016
C	-0.19369431837255	1.92150028005551	1.82487097178125
C	0.41493327300833	2.49384930342747	0.70208965774173
C	0.70216104948941	3.86219329236936	0.72974003237014
C	0.37796358250441	4.69304129733948	1.82846592975075
C	-0.24923382443206	4.07114688248203	2.92919047583801
C	-0.53068025928640	2.71341736494406	2.91388821035591
H	-1.00336013163432	2.24985462710180	3.77165842711005
H	-0.51204070529204	4.67330204551295	3.78850816842781
H	0.61643022445777	5.69498142214416	1.82084290461114
H	1.18635458692550	4.31788799127021	-0.12661283466123
H	-2.65503977748575	-0.69876886306929	1.98099207320418
H	-3.45187301742432	-2.71375507094408	4.10269558895879
H	-2.62946899022405	-3.10105821316133	2.58956924063451
H	-4.23238062153753	-2.37019457041914	2.56717722838599
H	-4.55871232411851	0.03781044694495	3.34064709888134
H	-3.63017402811648	-0.20391647486698	4.82311793270483
H	-3.15753608090091	1.05191769700821	3.67683638192266
H	-1.78640652859994	-2.13864851052565	5.28349957963826
H	0.43790519900187	-2.07286846003586	6.32027769872771
H	2.27823230416305	-0.89591220417706	5.19043849149112
H	2.00404716851453	0.68496013907760	1.84390975067106
H	4.23981709027772	-0.16309069720138	2.11169500611003
H	3.87114161411019	-0.86986757558168	3.67787678223917
H	3.11255086105177	-1.51214268280456	2.21811410318622
H	3.61827804404285	2.10088045318252	3.06217118102745
H	3.10872405043220	1.40834006129697	4.59942330097668
H	1.99114805421212	2.40580718697459	3.66750804999468
H	-0.41576119681034	-2.14191948292698	0.92862548141334
H	0.74637592250178	-4.17371273524925	1.64036533870455
H	3.05564245534453	-4.62574381338104	0.87303115367805
H	4.19411439553776	-3.00835176205459	-0.61518980374541
H	3.03506147810781	-0.97122606507926	-1.33615910022178
H	1.19942067970989	2.28073560919854	-1.12315290583599
O	-1.25020319182676	-0.22126163820968	-0.98826346368454
Si	-2.70306733435242	0.37539610706525	-1.51477483906146
C	-3.20655701485091	1.86123097055711	-0.49532457433359
C	-2.63305393151273	3.11520917998585	-0.72569923869452
C	-2.98300705747084	4.21513856832419	0.04259397535966
C	-3.90674317991839	4.07718509753097	1.07048477811431
C	-4.48039844135499	2.83892382459517	1.32240381751496
C	-4.13418635006967	1.74358230955108	0.54216145883588
C	-3.94947226819855	-1.00070973938546	-1.28243225373702
C	-3.64055282375844	-2.17998733959973	-0.60386767461307
C	-4.59293988414943	-3.17737215654845	-0.43367558430242
C	-5.87304253583568	-3.00993855968319	-0.94172523541197
C	-6.19847726272441	-1.84290880525826	-1.62221015769688
C	-5.24337388527323	-0.85121001570224	-1.78965974454697
C	-2.58023150136263	0.84488270407804	-3.32321416962631
C	-1.76690546166162	0.10798906776764	-4.18922088413176
C	-1.70591184910434	0.40664299315727	-5.54282718940823
C	-2.46542576848094	1.44885271142768	-6.05873864478001
C	-3.28436498697722	2.18794747649756	-5.21665550498834
C	-3.33818130301459	1.88719119320333	-3.86192951650623
H	-1.90301356580874	3.24176393235281	-1.51711240916800
H	-2.52720294189628	5.17716498645950	-0.15182324301989
H	-4.17769330517708	4.93377130479534	1.67437544030928
H	-5.19857028095538	2.72532252571765	2.12436582448112
H	-4.59870929942481	0.78603534980538	0.74837404527605
H	-2.64703853993888	-2.33230636467419	-0.20005734452245
H	-4.33355691839401	-4.08480805076468	0.09669742380072
H	-6.61572183958760	-3.78642680959053	-0.81018329936136
H	-7.19504063803745	-1.70791100921086	-2.02269700494094
H	-5.51665112244963	0.05104031776828	-2.32683354374310
H	-3.98026764188136	2.47979768599052	-3.22048070837718
H	-3.87919580153953	3.00061491489055	-5.61354632867275
H	-2.41903586428040	1.68334076452167	-7.11451410434891
H	-1.06671419394434	-0.17372702220565	-6.19582286016117
H	-1.16907015399877	-0.71259675808419	-3.81000795241926
H	-1.35082400834623	0.32722729253999	1.42118811291685

Compound 2c-V

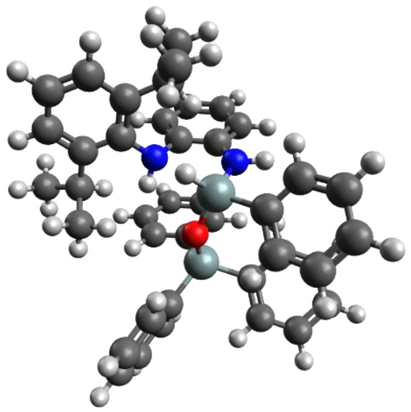

E = –2386.22022221774 Eh

Si	-0.43122411865510	-1.27167626931951	0.10080473973893
H	0.38648570202747	-1.97593574778942	1.10985439964968
N	-0.81606434033322	-0.07334760154432	2.58434074740031
N	-0.20540348627998	0.45591456968906	-0.06048393521675
C	0.12001546826184	-1.89768910182993	-1.58569733075037
C	-0.60790365891623	-2.86741843431150	-2.27837399508699
C	-0.18153954686941	-3.34488290802518	-3.51001925901506
C	0.99106264649176	-2.86173255607869	-4.07501301052303
C	1.73730961763902	-1.90657305123747	-3.39834947845261
C	1.30454702050298	-1.43510560857452	-2.16595767064494
C	-0.17097891475620	-0.48745196459249	3.77347176453884
C	-0.93505321602084	-0.95913515069175	4.85999828470660
C	-0.27516962201951	-1.38731330607371	6.00611620721768
C	1.10400093210939	-1.32608358402248	6.10772728777167
C	1.84096965762596	-0.85630145736470	5.03636209741914
C	1.23230608382490	-0.45183999968838	3.85208617927654
C	2.11864267986043	0.00082083746033	2.70947627188846
C	3.18920876291315	-1.03819301022905	2.37255387217150
C	2.75426322177683	1.36002403006848	3.00302437118489
C	-2.44625162598891	-1.04554235365800	4.78253676702939
C	-3.14590731050718	-0.88376620983832	6.12919987634816
C	-2.87209927726751	-2.35997966222013	4.12099328339484
C	-0.67129128066547	1.27976321295339	2.16800241150161
C	-0.37046619645822	1.50849764425398	0.82020252954962
C	-0.21668716206212	2.83000568539736	0.39072324202109
C	-0.35903142642405	3.88594708895896	1.27516471741073
C	-0.66057810641710	3.65282658992603	2.61061102234877
C	-0.81630947255684	2.34478321455267	3.04465368737108
H	-1.04585051146220	2.13904517949075	4.08372205834186
H	-0.77283243702083	4.47583344026294	3.30341880759409
H	-0.23031921396263	4.89883778568593	0.91547447825746
H	0.02208926093633	3.02605057640592	-0.64836018693845
H	-2.80633196576180	-0.21646493085722	4.16481732112111
H	-2.54343457167580	-3.21007886154078	4.72165162219976
H	-2.44760379516397	-2.48018447009821	3.12311426219681
H	-3.95819517116660	-2.41806497788093	4.02637733309593
H	-4.22296455677056	-0.80862131125381	5.97387869082321
H	-2.97674385889345	-1.73529078349374	6.78973283412126
H	-2.81919618068729	0.01797947211430	6.64802421455683
H	-0.84672263220638	-1.75992954674547	6.84599578144973
H	1.59971196898380	-1.64654845776992	7.01486113875600
H	2.92067000795128	-0.81648694794276	5.11661350853419
H	1.51267535930231	0.10847342532343	1.81328700665386
H	3.74691827331182	-0.72506938268551	1.48814786133764
H	3.91018901041321	-1.16948607337878	3.18085006491094
H	2.74337730477198	-2.01019324768648	2.15846967694429
H	3.35586546370062	1.69313908844698	2.15536366671656
H	3.40990261960815	1.30978986849921	3.87478103319901
H	1.99695114964296	2.12035455387076	3.19522255481782
H	-1.52964945409251	-3.25620539392158	-1.86317360958374
H	-0.76722170304176	-4.09163702472384	-4.03094009781561
H	1.32342792775267	-3.23002441877029	-5.03720755189851
H	2.65553178561406	-1.52870895182389	-3.82981749544221
H	1.90907067787839	-0.69304012515866	-1.65470811467571
H	0.05731719686428	0.76084893563768	-0.98760284871682
O	-2.01731573252743	-1.69985257949606	0.31256668442268
Si	-3.54683008505144	-1.25018960852985	-0.15379098592715
C	-4.03618041424652	0.33108390548524	0.72886921194253
C	-3.89365361244811	1.59292873940588	0.14696730373246
C	-4.21652012664040	2.74777473887689	0.84588525691874
C	-4.68950724875065	2.66309128897529	2.14806824442748
C	-4.84806093950360	1.41920124533452	2.74339877771239
C	-4.52810694622148	0.26824735432936	2.03631147324422
C	-4.68027271027991	-2.63639114807468	0.38532142476139
C	-4.18392126211036	-3.90767750377594	0.68019204064885
C	-5.03559408527844	-4.93870820668480	1.05459080416775
C	-6.40311297548818	-4.71522551814052	1.13636541771005
C	-6.91600012439061	-3.45815817739385	0.84386778966258
C	-6.05974066456488	-2.43084808869500	0.47336853305231
C	-3.61839900632628	-1.04295231050465	-2.01719048384538
C	-2.75775983265850	-0.17351745121230	-2.69529107416827
C	-2.79182427451286	-0.05692333409704	-4.07636026491823
C	-3.69176644150829	-0.81465584594668	-4.81457598899504
C	-4.55476367794353	-1.68368297871765	-4.16323681710539
C	-4.51574006475434	-1.79436870470776	-2.77890343320997
H	-3.53119174072775	1.68786845855851	-0.86900677642219
H	-4.09476053870332	3.71457275641226	0.37478758598296
H	-4.93850780641256	3.56319287803570	2.69527939528238
H	-5.22407179829457	1.34355402267988	3.75588566169232
H	-4.67608303477626	-0.69386750072266	2.51381643850678
H	-3.11978041130460	-4.10237266364355	0.62489032985295
H	-4.63123760775255	-5.91652181512029	1.28312951770558
H	-7.06785924772881	-5.51791478808901	1.42893894630353
H	-7.98145471756845	-3.27790138816510	0.90743305910092
H	-6.47843298771544	-1.45439062941411	0.25527432921434
H	-5.19393889016234	-2.48555336833559	-2.29283725374671
H	-5.25695937067877	-2.27968908229925	-4.73200892249428
H	-3.71771942523144	-0.72891525318723	-5.89334107817581
H	-2.11215979167251	0.62028072849093	-4.57747323513426
H	-2.03386861791801	0.41844074542673	-2.14793897957454
H	-1.78831877380830	-0.34768123323424	2.56433397686468

Compound 2c-VI

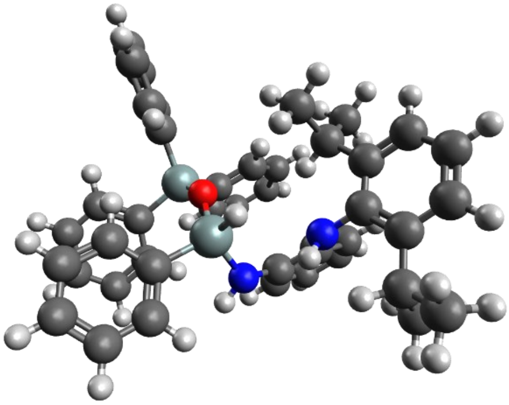

E = –2386.21724409587 Eh

Si	-0.71794538206144	-1.45112212412714	-0.05466829304033
H	-0.07006537326851	-2.32612168492574	0.94483287340541
N	0.43680175708887	-0.26737196447595	2.65752987668136
N	-0.09375257579988	0.18354781701652	-0.01011304149819
C	-0.34690170565165	-2.06859805421012	-1.78033417107998
C	-1.18143825317854	-3.00593796287483	-2.39326687306181
C	-0.91452918482883	-3.47696282803385	-3.67051543040310
C	0.20130565393091	-3.02089021089248	-4.35990225344304
C	1.05126837558147	-2.10057938304082	-3.76270928848642
C	0.77891056163050	-1.63333963004119	-2.48387157431571
C	0.71179460918229	-0.63434371147226	3.99120681013156
C	-0.35029967108428	-1.03153770319155	4.81688354373423
C	-0.06583472114110	-1.42061361899229	6.12204513364472
C	1.23344470918446	-1.43650030076328	6.59703767862679
C	2.27486330555413	-1.05967723638456	5.76483321819054
C	2.03881954670302	-0.64582340774829	4.45826419772960
C	3.18325997571464	-0.17661050965191	3.57978208913805
C	4.53419688846504	-0.77876981085433	3.95500620340337
C	3.26434112795862	1.35237551736968	3.58807186104315
C	-1.77389344996494	-1.11491006970125	4.30714867371124
C	-2.77236194589332	-0.46253325392771	5.26097835495815
C	-2.14922663354489	-2.57013539248514	4.02265316833335
C	-0.15445998239666	0.94040394537694	2.30504466286890
C	-0.41110195137020	1.16882605994484	0.94000327233380
C	-0.96187237811420	2.38545358585112	0.55390756034965
C	-1.29042747483994	3.36082135485662	1.48555505328425
C	-1.07876896369738	3.11350732261021	2.83202884539131
C	-0.50862146550972	1.91405022862688	3.23580251994367
H	-0.31841117727109	1.73870620829974	4.28717035177983
H	-1.34195709031862	3.85506384579976	3.57487410744368
H	-1.72863510872402	4.29375200024213	1.15698447349766
H	-1.15479175059766	2.55997467519965	-0.49878941735135
H	-1.83951455895266	-0.58072376553933	3.36006018644691
H	-2.14047576446182	-3.16953268986142	4.93507585877554
H	-1.45494722106918	-3.02444632587463	3.31466931261694
H	-3.15089220119719	-2.63065741044834	3.59145787736296
H	-3.77727423371496	-0.49117421956250	4.83646285438746
H	-2.81982348794498	-0.97407378094134	6.22335613053576
H	-2.51937286544100	0.58212788227819	5.44722020238209
H	-0.87069193351927	-1.73677897133712	6.77418990112001
H	1.43758830007605	-1.74988976402369	7.61278983150525
H	3.28577349275241	-1.08254425178763	6.14948418091545
H	2.99062181077152	-0.49118211755351	2.54986024367710
H	5.27809369207564	-0.50332117183079	3.20675743124886
H	4.89699426451988	-0.41188904273253	4.91636700311045
H	4.49526128312361	-1.86770045905815	4.00139368328797
H	4.05544309300979	1.69982124263899	2.92125394299492
H	3.48834290360502	1.71664093491534	4.59253935608069
H	2.33091239622388	1.81349802498442	3.26645575500576
H	-2.06224184960918	-3.37317152174846	-1.88015081416394
H	-1.58029479504013	-4.19621118151260	-4.12991003858095
H	0.40858689158477	-3.38369151438666	-5.35846887908721
H	1.92586627971387	-1.74517746899148	-4.29232273864404
H	1.46056325983594	-0.91818919666028	-2.03724109331796
H	0.00185270482283	0.58568712213494	-0.93451687518371

O	-2.33578648829443	-1.51967990935486	0.27929539849525
Si	-3.82753844064358	-1.05957087319788	-0.28910206162330
C	-4.37230512793490	0.50947013747409	0.56995683159559
C	-4.82594769442550	1.62957154179734	-0.12735392966673
C	-5.24764262452006	2.76954465144265	0.54626947340968
C	-5.22124823842751	2.80597868076812	1.93276482409667
C	-4.77756696552689	1.69833178518192	2.64510689225643
C	-4.36136783295155	0.56349997242405	1.96657167752469
C	-5.00120483968150	-2.44966093114831	0.14211291425121
C	-4.54454949306304	-3.74804315349069	0.37772981545526
C	-5.43193425046481	-4.77588565830985	0.66835397879753
C	-6.79535129390810	-4.52072633858863	0.72530801838161
C	-7.26801777604561	-3.23542068738102	0.49414034312826
C	-6.37615236227432	-2.21134650501118	0.20792867257672
C	-3.75449231619042	-0.80024979448586	-2.14118031081286
C	-2.90656813757397	0.16777021141106	-2.68734586060153
C	-2.77842400421605	0.31746768310522	-4.05973301481697
C	-3.50694532568425	-0.49907195390865	-4.91588599493299
C	-4.36438503052117	-1.45665030671157	-4.39335907591707
C	-4.48333955272824	-1.60467182344811	-3.01735705456302
H	-4.85421100768868	1.62081521941385	-1.21045781938844
H	-5.59572761350056	3.62892006866406	-0.01243646894231
H	-5.54702817888541	3.69409549635172	2.45898785545089
H	-4.75614731346482	1.72024235002044	3.72729299368157
H	-4.02975941644234	-0.29604719222849	2.53790847584528
H	-3.48398463035566	-3.96715266748392	0.34061878337195
H	-5.05919319669480	-5.77556348279409	0.85169129005601
H	-7.48818404734443	-5.32085994080953	0.95247345170691
H	-8.32997216830701	-3.03094120687481	0.54129728910638
H	-6.76235528207863	-1.21177534104954	0.04005760216219
H	-5.14893612298000	-2.36577994365434	-2.62721923741906
H	-4.93636865182868	-2.09260139990361	-5.05684908871256
H	-3.40697856781936	-0.38654012610244	-5.98786467477113
H	-2.11102408365229	1.06840130287754	-4.46265365059627
H	-2.32982693551086	0.81769415593886	-2.03766239314441
H	1.12294327872784	-0.57743807743424	1.98523655473772

Compound 2d-I

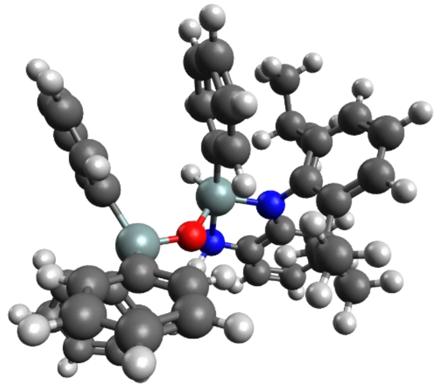

E = –2386.21658123382 Eh

Si	0.60991544613234	-0.85064378402374	0.51007986918777
H	-0.65903538060109	-1.59584472248037	0.25361210582219
N	1.73917207866464	-0.59913377230532	-0.85279490042465
N	1.39941339976071	-2.94200743669334	0.25411616147991
C	-0.19246100012761	0.87334914018495	0.67649559101660
C	0.43370856739820	1.97182754823341	1.26994347722457
C	-0.22575523650873	3.17821206493936	1.45195900053296
C	-1.54956829461019	3.31372459530707	1.04921033706076
C	-2.19981973216071	2.23428417854134	0.46992012661818
C	-1.52294605691629	1.03231160168518	0.29232435619599
C	1.90355886413374	0.67965131196632	-1.46429198650070
C	1.03241505973553	1.06562127788732	-2.49640207920284
C	1.25545888252509	2.28246126995096	-3.13352576315193
C	2.30791749613130	3.10082231296289	-2.76428675082487
C	3.14890166704016	2.71581195218262	-1.73561220133929
C	2.96389900273678	1.51053497329042	-1.06446885469146
C	3.92517675413930	1.13506693512230	0.04702068153132
C	4.25767505922764	2.31355369736973	0.96448577847976
C	5.22792669361455	0.55669531369366	-0.51252518405212
C	-0.11486421946694	0.19138547593511	-2.96085058887877
C	0.27090848234411	-0.60071629535606	-4.21302356063242
C	-1.39337335315746	0.98593524687514	-3.22573572321971
C	2.44725999222092	-1.64656456727870	-1.42458654161340
C	2.30387553217399	-2.91137463944379	-0.84881352485017
C	2.98242858598951	-4.01386281672615	-1.33359686582409
C	3.82513608486990	-3.88521248190554	-2.42975116367298
C	3.96466694979895	-2.63827387436001	-3.02272195194236
C	3.28970300177951	-1.52902511743452	-2.53482959886881
H	3.42354862318803	-0.57236367626030	-3.02080914624600
H	4.61139062187576	-2.51887950339269	-3.88267277058983
H	4.35708046206385	-4.74402502732802	-2.81569416021785
H	2.84508973641912	-4.97571844127622	-0.85378476514103
H	-0.34039328234771	-0.52921346672645	-2.17251903882920
H	-1.30768228440924	1.61664302291353	-4.11208373439230
H	-1.65512321420274	1.62290599400215	-2.38162992821883
H	-2.22492419074634	0.30132288167279	-3.39984853022122

H	-0.55300557431760	-1.24485969114149	-4.52583575556382
H	0.49953932683710	0.07270332181187	-5.04183418341484
H	1.14202419036187	-1.23206631738260	-4.04404175350922
H	0.60014610297253	2.59749757623696	-3.93581679369679
H	2.47015045692813	4.04132739228791	-3.27526789422247
H	3.96462002004140	3.36897948285426	-1.45247919331684
H	3.45365989182546	0.36245221233558	0.65775913013035
H	4.83548922434274	1.96399531460106	1.82181952133359
H	4.86548420117258	3.06556081235344	0.45918652722497
H	3.36510280410023	2.81117907557640	1.34401788710034
H	5.90068160895214	0.27976807023801	0.30129565293578
H	5.74251768293416	1.29367193421933	-1.13283766602309
H	5.05616244979130	-0.33086664177913	-1.11829418052522
H	1.45835666894456	1.88808776032540	1.60896524465475
H	0.28854483961338	4.01202689738596	1.91405824142703
H	-2.07047506479066	4.25193545051537	1.19356329097842
H	-3.23447352027730	2.32439741286412	0.16251383706977
H	-2.05527025518211	0.19466122188479	-0.14730841052892
H	0.61039924803830	-3.55350138007770	0.06920360336116
O	1.33614572801684	-0.96297051680990	2.02111459086047
Si	0.80215891080988	-1.07699606510142	3.57953422241104
C	-1.03247290435508	-0.72359837779964	3.69523728972564
C	-1.51346872973165	0.49886676245149	4.16682882859619
C	-2.87227038052442	0.78284179259370	4.16636923014236
C	-3.77685874565265	-0.15539813378762	3.68843142298258
C	-3.31887484699912	-1.37799608946825	3.21505967649117
C	-1.95953100128249	-1.65658608130450	3.22180410057653
C	1.78148834346987	0.12646534050581	4.62644131325677
C	2.82887022144397	0.87842210786298	4.09527556505290
C	3.56681785016836	1.74447328935227	4.89183957981416
C	3.26726866696863	1.87076623211411	6.24086001468374
C	2.23120233621059	1.12535143792654	6.79025745589946
C	1.49975756222316	0.26083988657123	5.98880499365194
C	1.15627164003615	-2.82199017951444	4.17724237722692
C	0.30775598779251	-3.49573529440231	5.05732023713663
C	0.61683188138024	-4.76847668697968	5.52100595996369
C	1.78596373799893	-5.39257879950441	5.10947471555228
C	2.64777993777558	-4.73669885941925	4.23945959376943
C	2.33505046927818	-3.46384165891822	3.78384544198211
H	-0.82219237261842	1.24910750299283	4.53166559565616
H	-3.22480097539640	1.73806973110592	4.53384117880688
H	-4.83666934058078	0.06533652500624	3.68446357010835
H	-4.02036026559509	-2.11311918008465	2.84174928383813
H	-1.62247011200097	-2.61753817634885	2.84862439261624
H	3.07975798667082	0.79020868449194	3.04581408991615
H	4.37486229167099	2.31985000769002	4.45749409164094
H	3.83942721134185	2.54609512577329	6.86409155231988
H	1.99469397104985	1.21827149201292	7.84253431816481
H	0.69685494786414	-0.31458120803279	6.43776819160088
H	3.03078818994522	-2.96401122372002	3.11783823787911
H	3.56431826135732	-5.21582000714855	3.91941542944711
H	2.02686316224101	-6.38555657127078	5.46689231784119
H	-0.05701177373520	-5.27355593414159	6.20130541781375
H	-0.61241058281696	-3.02810400118660	5.38815337408815
H	1.84156763657819	-3.29335395034239	1.09810698146801

Compound 2d-II

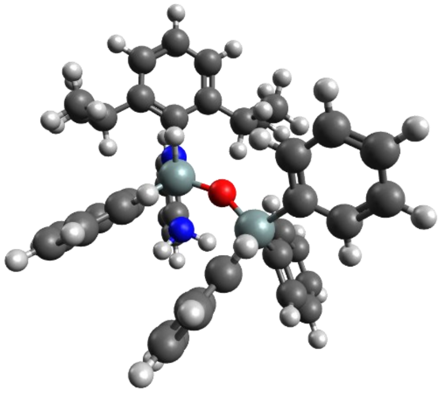

E = –2386.22010148288 Eh

Si	0.32667322426100	-0.30829151949438	0.43245216687172
H	0.34350532243106	1.18385755958676	0.21893829890261
N	1.46257750794484	-0.66727669076366	-0.88887982765393
N	0.53420313277095	-2.59648922568637	0.59000155787836
C	-1.51324461578676	-0.63018020548876	0.18376209771747
C	-2.41382817982259	0.12287639093842	0.93944167592253
C	-3.78579363813466	-0.02635213610473	0.78991578977058
C	-4.28743429740611	-0.91938924100532	-0.14765957753787
C	-3.40989855542064	-1.66007772102893	-0.92778041944092
C	-2.03875955321833	-1.51948980037336	-0.75514682044040
C	2.07016006858164	0.42351934079183	-1.57393328532235
C	1.43733430540195	0.98013317447597	-2.69336766381528
C	2.03950388732133	2.06381920410010	-3.32579388430938
C	3.24406065789181	2.57522189981388	-2.87352146176182
C	3.86818892399250	2.00099859653028	-1.77919942195848
C	3.29885558533448	0.91859295847151	-1.11527437749897
C	4.01407723358478	0.32179718132766	0.07685574808344

C	4.10142884286896	1.32436594244455	1.22731398132804
C	5.39781111835771	-0.20378852258393	-0.30400128073503
C	0.12131268445127	0.44951558819384	-3.21702494356552
C	0.20788002561268	0.07202582150867	-4.69543133377442
C	-1.00790753601303	1.44932389275535	-2.96823351554099
C	1.81369607903930	-1.94254427295972	-1.29016606800908
C	1.32589083813299	-3.00556347394848	-0.52573491157463
C	1.61838758767629	-4.31813941759408	-0.84323180434357
C	2.41209296089992	-4.60460500076921	-1.94748125754975
C	2.90547742191225	-3.55664093473883	-2.71171927421085
C	2.61730205394826	-2.23621240254965	-2.39484715665768
H	3.01592278553197	-1.43988524174372	-3.00864642905988
H	3.52711326858182	-3.76452105453431	-3.57329856661942
H	2.64176922998383	-5.62992993277821	-2.20341466973623
H	1.22588403820019	-5.11685007173799	-0.22513441616329
H	-0.12304223269243	-0.45993869603085	-2.66771927153314
H	-0.84216474085354	2.38061913175504	-3.51370400355797
H	-1.09480736645954	1.69093448384988	-1.90845767768961
H	-1.96418378460478	1.03698486926529	-3.29512973456076
H	-0.72865366652785	-0.38123360545940	-5.02436129649956
H	0.39008637536913	0.94108562311399	-5.32992512215784
H	1.00773629722662	-0.64768857508653	-4.87466328817558
H	1.55987384452217	2.51761453590124	-4.18499528824655
H	3.69802936943334	3.41983397611241	-3.37606844813532
H	4.81306513734608	2.40457054975595	-1.43448396641926
H	3.43398077248588	-0.52976458058897	0.43268964178950
H	4.55220094383969	0.85987911956146	2.10635176628857
H	4.71119887807598	2.18949023952858	0.96029840270164
H	3.11339532634499	1.68603921334874	1.51293528235974
H	5.86740744085046	-0.69017613030397	0.55282189443147
H	6.06140028662687	0.59915077246347	-0.62991472511533
H	5.33520618379142	-0.93567827790447	-1.11028807325209
H	-2.04437660261266	0.83761661581153	1.66674672178718
H	-4.46267446692780	0.55668221958710	1.40160546982677
H	-5.35655653989324	-1.03409276870044	-0.27310696691296
H	-3.79264588806963	-2.35058612396345	-1.66870987229214
H	-1.37748944699996	-2.11239675737640	-1.37895001680155
H	-0.37538368008109	-3.04792059664348	0.59458996956667
O	0.85371363102767	-0.30699977828311	2.01509149220053
Si	0.70309541670974	-0.74463258746325	3.59128097370398
C	-1.07712295708597	-1.15209062390001	4.00935892861778
C	-1.89939400569911	-0.20733992430265	4.62599741994270
C	-3.24401931637054	-0.46691710717325	4.85643753851731
C	-3.79039864329140	-1.68370515999710	4.47285464070292
C	-2.98741120037129	-2.64061844180485	3.86541873023730
C	-1.64494076714019	-2.37437020195243	3.63954343354672
C	1.28199264025899	0.70579208398264	4.62313004168970
C	1.30917232351478	2.00086558891961	4.10220921487017
C	1.71307529566982	3.07697838941352	4.88179068541806
C	2.09635060475654	2.87395530231801	6.20038113597746
C	2.07550372557369	1.59249675037222	6.73596539694320
C	1.67254121925780	0.52107334279395	5.95143640874171
C	1.78985098821179	-2.24276119935098	3.90318788574985
C	1.49283667082291	-3.17087167894491	4.90442394782394
C	2.32624573085407	-4.25419382686608	5.14924099458101
C	3.47417133678151	-4.43243801747537	4.38912358837024
C	3.78787222444255	-3.52192503813623	3.38872683129135
C	2.95474466014136	-2.43701256718615	3.15354892723598
H	-1.49056895997024	0.74907575536472	4.93130672405959
H	-3.86475664905180	0.27983620363379	5.33490406657933
H	-4.83860474161544	-1.88824865286917	4.64974614441157
H	-3.40810330639478	-3.59286657327438	3.56871859323314
H	-1.03575917563191	-3.13880056560874	3.17000231479224
H	1.01613336010370	2.17876637181340	3.07400283315293
H	1.72979015556281	4.07362336426976	4.45940545343790
H	2.41278091286548	3.71138698977681	6.80901270167885
H	2.37649713120709	1.42855608846515	7.76280385204487
H	1.67039539298822	-0.47278302350730	6.38610222569095
H	3.22085875581327	-1.73620730081222	2.36999441559517
H	4.68124099260509	-3.65576396357521	2.79228490302885
H	4.12217128811311	-5.27935432460074	4.57496636595198
H	2.07729428881268	-4.96168694799812	5.92979035037877
H	0.59605571395244	-3.05499520614750	5.50246788893429
H	0.98495041347358	-2.86673944294556	1.46032260427059

Compound 2d-III

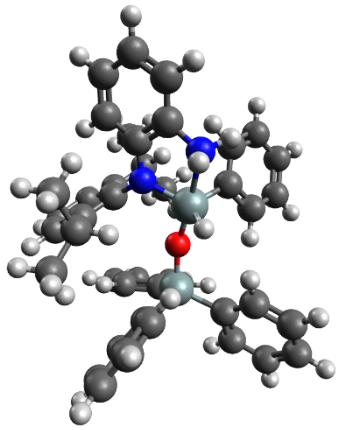

E = –2386.21751207862 Eh

Si	-0.04049646298494	0.06117539592282	-0.03070143647953
H	0.37431704065697	-0.12577085711942	1.39040967615678
N	1.14725279219867	0.09627979234031	-1.38392002405656
N	0.62036593536759	-2.04517465983066	-0.04482875318894
C	-1.77719223230090	-0.52627463942812	-0.53078810052688
C	-2.91013438502633	0.09917523169860	-0.00369925910068
C	-4.19483406340283	-0.27652011851184	-0.37524288682785
C	-4.38301379094826	-1.28068508971444	-1.31313573958694
C	-3.27530384233067	-1.90675557598025	-1.86747724141353
C	-1.99659340020805	-1.53801114414661	-1.47237863698073
C	1.50166474822008	1.29827833733020	-2.06734311397348
C	0.74402286306236	1.72569345776835	-3.16752550368247
C	1.15393214377267	2.86142363144647	-3.86022956099106
C	2.29003106954083	3.55313957567434	-3.48743908233367
C	3.03006539401340	3.12164982925123	-2.39947120099373
C	2.65619331463017	1.99563945665016	-1.67160197433840
C	3.51066850517150	1.53198666579917	-0.50800418424436
C	4.27669667859763	2.66711220691472	0.16671554323554
C	4.50453361147119	0.45006116115685	-0.94085238149138
C	-0.50116675854321	1.00319020001683	-3.63331929913316
C	-0.40556383131603	0.58538524785353	-5.10106055333742
C	-1.75091151674476	1.84986045545482	-3.39267311575055
C	1.57345006465245	-1.08417830912010	-1.97084421527283
C	1.30041336159244	-2.26789925639265	-1.28019284321726
C	1.65057134614453	-3.50701291315868	-1.77797696638507
C	2.30821207155320	-3.59825939757417	-2.99877433800666
C	2.60500648736484	-2.43144998273759	-3.68850287538285
C	2.24788824208107	-1.18574801843914	-3.19128497830637
H	2.49790857801507	-0.29803016186621	-3.75517629665406
H	3.12374357931101	-2.48446329498953	-4.63725900474888
H	2.58574882220005	-4.56339958590256	-3.39974559661863
H	1.41096126556016	-4.39882451444885	-1.21122800737879
H	-0.61282169934653	0.09159906665028	-3.04898654110200
H	-1.72698342102249	2.77303946637372	-3.97589825312649
H	-1.84878887916443	2.12124167517472	-2.34256878153004
H	-2.64692681585744	1.29671367388137	-3.68115927861991
H	-1.28064990602486	-0.00362666071775	-5.38098826471761
H	-0.36581904051572	1.44717995442906	-5.76960490684884
H	0.47891928434371	-0.02472676951232	-5.28696860451641
H	0.57240501974079	3.21114940534501	-4.70491848252368
H	2.59785064294674	4.43306798000372	-4.03818519773888
H	3.91468761569489	3.67664040359011	-2.11707945388231
H	2.84625685091035	1.09595928850637	0.24278725676986
H	4.73373383687722	2.30422956709860	1.08744876415550
H	5.08410129492299	3.04445572426592	-0.46394421597501
H	3.62871665945167	3.50282831297584	0.42914684665903
H	5.10464087803966	0.12441522253827	-0.08895185379373
H	5.18798682922075	0.83923297555306	-1.69861038902416
H	4.01477288930958	-0.42817261494464	-1.35581785226634
H	-2.80644464505658	0.90222049148770	0.71307970984726
H	-5.04832601568664	0.22502728415480	0.06340342178406
H	-5.38210808143257	-1.57007252367866	-1.61313265663275
H	-3.40435547422255	-2.68443660897082	-2.60962919248662
H	-1.16307557778180	-2.04765319808058	-1.94151834317060
H	-0.12948395894853	-2.71153479880752	0.11382719020618
O	-0.31622830786028	1.73032160068703	0.06302967592330
Si	-0.53120844367579	3.16868455766169	0.80621025107085
C	-1.08119618806023	4.40812440042799	-0.48604436215941
C	-0.14469302803639	5.02227469682090	-1.32042885937553
C	-0.54136718005157	5.85448981876106	-2.35812609461492
C	-1.89095000411639	6.08625003200143	-2.58416520824153
C	-2.83974178453037	5.47606621595525	-1.77410513704419
C	-2.43530072450793	4.64452395680993	-0.73902574014735
C	1.03349744467631	3.71900338796823	1.68956832710855
C	1.71572433863765	2.79211838313591	2.48391574301429
C	2.78488706590471	3.17413924341292	3.27959199611618
C	3.20196493226970	4.49902217166433	3.29206294197093
C	2.54079735511694	5.43455556847976	2.50953574224360
C	1.46293171034429	5.04633085282169	1.72238878807674
C	-1.80016334268588	3.04445831756926	2.19464891285380
C	-2.52951729257205	4.16280552303120	2.60662003229935
C	-3.38168518050911	4.10637681559666	3.70166760300037
C	-3.51511437761836	2.92471176878416	4.41761799352534
C	-2.78634070104957	1.80598561384399	4.03698827041874

C	-1.93750420736703	1.87064521383197	2.94054054044259
H	0.91412908030553	4.83920764095783	-1.17800640470109
H	0.20376482149201	6.31505715550398	-2.99422593336190
H	-2.20331097613622	6.73527214131819	-3.39240772881567
H	-3.89428486881016	5.64518245903750	-1.95104673144848
H	-3.19390063428357	4.16934005269345	-0.12784501284864
H	1.40837592477010	1.75169377045871	2.49051815702349
H	3.29663639825794	2.43875524205252	3.88757936500562
H	4.03808035991744	4.80032777614968	3.91021264196203
H	2.85942288844895	6.46931147375041	2.51545376269608
H	0.95061351308783	5.79836323936270	1.13375317285049
H	-1.37408340558820	0.98378623003433	2.67148490061335
H	-2.87775818009778	0.88265389913215	4.59485317494153
H	-4.17984341591637	2.87704761151007	5.27066089585310
H	-3.94057415315707	4.98554374906169	3.99623112710899
H	-2.43506180093461	5.10051422989012	2.07072826069453
H	1.25397644656541	-2.13800525341278	0.74466596548893

Compound 3

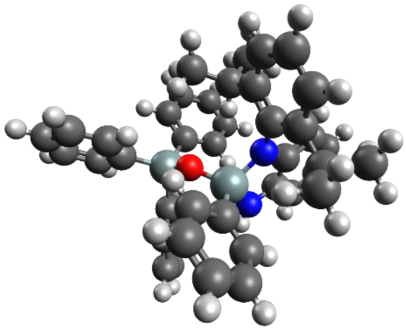

E = –2384.27037966 Eh

Si	-0.45879156455304	1.05777150761641	2.19918826345380
N	-1.29348544711471	2.15423577537668	1.12569246639798
N	-0.92475907636946	-0.29939246353326	1.22703539940024
C	1.36804084696601	1.32049341780118	2.36787635376038
C	1.90782168909185	2.08538540564599	3.40492254718591
C	3.27701137551841	2.29186459929971	3.49748080256752
C	4.13010463116636	1.72100261354430	2.56208676548385
C	3.61110050093629	0.94829514713889	1.53212043030928
C	2.24058920689195	0.75629079092406	1.43471793164363
C	-1.26911059568242	3.57229268857005	1.13623445372185
C	-2.21643973043169	4.27866776783333	1.89226259024988
C	-2.16849646207235	5.66993352106057	1.88343384176744
C	-1.21782598588795	6.34800838691249	1.14145803043610
C	-0.29832614921243	5.63815948123670	0.38868342047230
C	-0.30623670359251	4.24688213818115	0.36807142387274
C	0.69587182459626	3.52136300886587	-0.50304191962614
C	2.13283336001587	3.91681612603626	-0.16668069105575
C	0.39638601820158	3.75970549438899	-1.98418468988839
C	-3.29373863443448	3.58754852971375	2.69715676128171
C	-4.68443342998657	3.90322748146824	2.14569269932281
C	-3.19846412749776	3.94753511634689	4.17874706357105
C	-1.99510588728896	1.46060625041777	0.14299394119748
C	-1.78470565405617	0.06753808106065	0.20306345710865
C	-2.42381459191928	-0.77001877438391	-0.69338779446486
C	-3.26634440285507	-0.22415826065923	-1.66305191449240
C	-3.46671794151326	1.14455434231687	-1.72608886910512
C	-2.83216421065090	1.99582565279454	-0.81979767943855
H	-2.99445642543422	3.06534771571505	-0.86793571585837
H	-4.12035911060563	1.56417446379496	-2.47951126930514
H	-3.76222775465134	-0.87982380417390	-2.36681884632897
H	-2.26565353025308	-1.84056577796251	-0.64086581475473
H	-3.15320825157417	2.51125703809917	2.61250735919892
H	-3.38807756259138	5.00870332513248	4.35104809101067
H	-2.21208293281177	3.71518651477174	4.58047990363803
H	-3.93333249741550	3.38289858384674	4.75391131145631
H	-5.44434034799450	3.35006794176370	2.70053513440893
H	-4.92051250181038	4.96600834531549	2.22784155210563
H	-4.76829481074844	3.62058299897291	1.09558427630447
H	-2.89060987120952	6.23317760636919	2.46253459467300
H	-1.19633612270111	7.43036788209819	1.14504475890443
H	0.43590761294023	6.17786274681818	-0.19734110385473
H	0.60322414388527	2.45054920356043	-0.32045166717124
H	2.83437757690186	3.32498054005132	-0.75651038421398
H	2.32677932541077	4.96829200289441	-0.38626878956380
H	2.35741794366820	3.74324273164481	0.88600588001537
H	1.08662735206323	3.19052969123906	-2.60928088359326
H	0.50321704553679	4.81374303557438	-2.24791249624508
H	-0.61766381813065	3.45040265837182	-2.23939963931652
H	1.26006784254214	2.52717297358699	4.15268163983346
H	3.67935047512991	2.89269132350470	4.30292465095690
H	5.19867687162756	1.87675433856622	2.63768741172040
H	4.27358036680937	0.49824713042283	0.80402417234598
H	1.84839683452946	0.15713478772779	0.62126393294298
H	-0.72761726265003	-1.27674035803988	1.36939672111183
O	-1.05392800630842	1.06364398861775	3.73573647571211
Si	-1.98863614426208	0.03941625474900	4.65611319998625
C	-3.79224501687492	0.16863385426174	4.17474695858917
C	-4.20342752622699	-0.19513702401705	2.88821853051983
C	-5.52635729427171	-0.06466306010810	2.49362975043194
C	-6.47001586314409	0.42654384897442	3.38723188691778
C	-6.08378510637897	0.78537136271122	4.67080701610110
C	-4.75534088539665	0.65913913427061	5.05748657731419
C	-1.73280054808177	0.53867393492186	6.43806787867791
C	-1.09132594587045	1.72575705341659	6.79434096608431
C	-0.91791983048958	2.07111121435577	8.12844740823143
C	-1.38527473898281	1.23164520908993	9.12995024776071
C	-2.02370975889485	0.04442200746823	8.79434684248857
C	-2.19178182377958	-0.29673743355272	7.46018787788753
C	-1.36999810109411	-1.70619889471183	4.37111924118423
C	0.00666959886405	-1.95127137134611	4.32628176622224
C	0.50003571782123	-3.23030866029840	4.11466650505995
C	-0.37890039950094	-4.29412883443522	3.95189957229108
C	-1.74768003444860	-4.07238277174761	4.00318367138446
C	-2.23627532975435	-2.78825343322670	4.20986109132255
H	-3.48663759835462	-0.58683426888906	2.17617019012653
H	-5.81991433299652	-0.34560271772331	1.49024059705181
H	-7.50385493864257	0.52883403312442	3.08298342698862
H	-6.81562379080034	1.16768756880264	5.37081198364626
H	-4.47182183828227	0.95249415282300	6.06147274011263
H	-0.71661574504653	2.39366539842980	6.02829916191902
H	-0.41641257545853	2.99534003539040	8.38561688965193
H	-1.25030196349315	1.49968262865817	10.17000521052230
H	-2.38678487607471	-0.61555165076078	9.57169591924274
H	-2.68551002286883	-1.23202096017314	7.21798725483249
H	-3.30847970730394	-2.63350160049872	4.23813211796512
H	-2.43607064525672	-4.89839491837589	3.87823925881178
H	0.00354021405490	-5.29322134652710	3.78672622453295
H	1.56877125606655	-3.39839302027178	4.07850768989223
H	0.71205425280461	-1.13734913084363	4.45793800498067

Compound 5

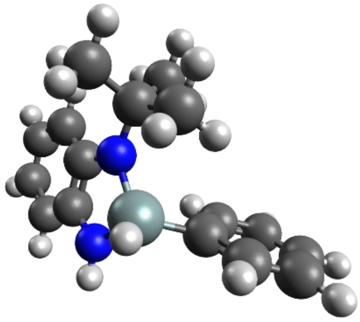

E = –1018.50293739 Eh

Si	-0.52822146003930	3.12876713423722	0.55558615033928
H	-0.85698125424877	3.94965631288489	1.75407689737578
N	-0.92127231644010	3.85439397265128	-0.99073620956569
N	-1.61708116493593	1.81282223305668	0.26705747470393
C	1.24474983739023	2.58890842075522	0.77978103157647
C	1.97356191795711	2.95158333301987	1.91063419055878
C	3.28881625267202	2.54036135719438	2.07553957750464
C	3.89278976909401	1.75898244683314	1.10231818574017
C	3.18162429071130	1.38794919593624	-0.03160811849504
C	1.86787969278197	1.79911438839416	-0.18882552200235
C	-0.39701269851026	5.13306731028583	-1.48567040918773
C	-1.80801568338223	3.04185731461895	-1.70856913573124
C	-2.20069062507576	1.89131719567383	-0.98669853213048
C	-3.07257486857862	0.96886956917907	-1.53120713660156
C	-3.57622082372866	1.16427966005115	-2.81415283472603
C	-3.19628069272056	2.27855355530248	-3.53357252399571
C	-2.31540835547796	3.21281418282662	-2.98870534087286
H	-2.04207514169374	4.06452772689397	-3.59138067785160
H	-3.57786324106396	2.43902155964194	-4.53294764356186
H	-4.25982476185107	0.44199476384102	-3.23942623216926
H	-3.35995570070971	0.09629320030215	-0.95664007071295
H	1.51010180521331	3.56654242494156	2.67465872878768
H	3.84196790917141	2.82926139230228	2.95978477857255
H	4.91882302614965	1.43751432465646	1.22622332057689
H	3.65350597364378	0.77743287955739	-0.79052091880694
H	1.32034425247284	1.50273317747626	-1.07660069818671
H	-1.79521035417495	1.00123493197983	0.83054685933045
C	0.47709595719333	5.75990117939772	-0.39533779671446
C	-1.53353348714071	6.12216149652543	-1.76845730545335
C	0.49269230911087	4.92079287502466	-2.71557654537406
H	1.34323707669424	5.14212998068741	-0.15124492916441
H	0.86164035163823	6.71944396103359	-0.74719955308160
H	-0.08683986120699	5.95511732782579	0.51906397245000
H	-1.12505047798368	7.08293754229143	-2.08553781040826
H	-2.12112211288491	6.28862721015769	-0.86480652004744
H	-2.21902345886012	5.78817996201409	-2.54317926328658
H	-0.03397932180106	4.46918028063633	-3.55326201296106
H	0.90103419446271	5.87262725674996	-3.05914454532875
H	1.32980324162203	4.26772696316193	-2.46465288109865

Compound 5a-I

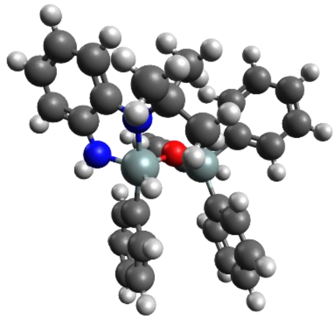

E = –2076.14665716 Eh

Si	-1.10284263298207	3.95231648346873	2.26138880280389
H	-1.72154930817831	5.16416731018755	2.91181369102800
N	-1.09985333077607	4.85505589809610	0.54028978968767
N	-1.90419003338466	2.67427981493880	1.33733209431454
C	-1.37708824341607	2.93825306639102	3.94601222328555
C	-1.92550170423679	3.58842211692859	5.05304852630743
C	-2.15757946353087	2.94493588152198	6.26394493863245
C	-1.84082800486768	1.60013779963028	6.40491529593256
C	-1.29746368660015	0.91776115272583	5.32342019079948
C	-1.07521446717865	1.58191634610368	4.12245528689416
C	-0.63025767108768	6.22006656223721	0.28234639205309
C	-1.55020998897942	4.06940707991550	-0.48094809702622
C	-2.03068541775969	2.80726741624043	-0.01907112435080
C	-2.55852866759847	1.86493487590292	-0.88144247930056
C	-2.62854565286736	2.12238760711701	-2.25535228887363
C	-2.16067630230559	3.33120623391260	-2.73009131519398
C	-1.62781201691586	4.29234309702108	-1.86274053615183
H	-1.28186334166290	5.21501555519220	-2.30209171671361
H	-2.20121595169240	3.55367322556579	-3.78985528154679
H	-3.04165202754539	1.38134557371066	-2.92804146270128
H	-2.91771321480874	0.92241742665681	-0.48136759169326
H	-2.17749160918766	4.64200733382458	4.96656115839569
H	-2.58319881143284	3.49073583174300	7.09820137827488
H	-2.01589404919729	1.08986930995022	7.34421280835724
H	-1.04627344009793	-0.13259531473628	5.41703646962360
H	-0.65001648317602	1.01378523395483	3.30137763890081
H	-2.24549409231690	1.82350273911348	1.75285750384498
C	-0.22700551521192	6.93958118112007	1.57821653593374
C	-1.74476364462485	7.08860360263072	-0.32978631453861
C	0.62522362433684	6.23101755032293	-0.60736399279770
H	0.56311329464642	6.41727607839617	2.11519313269266
H	0.15274063917497	7.93041021054481	1.32133519477427
H	-1.06821486992856	7.07530145851027	2.25527003075712
H	-1.38990649080290	8.10956519176076	-0.48630442766450
H	-2.59516198947866	7.13358601833737	0.35337158061678
H	-2.11324675828540	6.72354468654984	-1.28553307746085
H	0.47909632453599	5.75813774220977	-1.57597019456588
H	0.95725298391910	7.25546978665691	-0.79021709631612
H	1.43819228523242	5.70473315089356	-0.10598693325341
O	0.61068580680763	3.90856927703889	2.32142216437386
Si	1.92201205785393	3.65643091017689	3.25444527371091
C	2.52238187291237	1.88761413624331	3.04241202810688
C	3.68596827015858	1.44425137461933	3.67724684172472
C	4.16821915965823	0.15754008672744	3.48255532677874
C	3.49762995917657	-0.71187150954252	2.63138950202235
C	2.34993750371599	-0.28539865635676	1.97773262674352
C	1.87015419151338	1.00220637941268	2.18304373460125
C	1.65328071271655	4.10023910976149	5.05952365640585
C	1.20114013584212	5.38485531683069	5.37675359909914
C	0.98848452240827	5.77019686775114	6.69184468475882
C	1.22419218386675	4.87063365576651	7.72418285800741
C	1.67230343864209	3.59082816468925	7.43090695863194
C	1.88499139802438	3.21368687972911	6.11034381833755
C	3.31430337993828	4.74887706619260	2.59930272820054
C	3.65427804235276	4.67206209911367	1.24447821277243
C	4.68173656417862	5.43677434733106	0.71242456372745
C	5.40202249410645	6.29717063960970	1.53264628747352
C	5.08807631451930	6.38293360473052	2.88101923221291
C	4.05425918649073	5.61467819469303	3.40511798993778
H	4.23503679631458	2.11310974588286	4.33198194946103
H	5.06853050855077	-0.16580009041256	3.98971693030198
H	3.87219291728134	-1.71548440373595	2.47443547905120
H	1.82782323294843	-0.95477178598189	1.30560809214827
H	0.97457397301804	1.31877088416818	1.66128780717175
H	1.00427356900721	6.10161021089869	4.58644413356028
H	0.63550998067900	6.76972913440604	6.91256755956794
H	1.05674973396711	5.16631601272539	8.75216479677228
H	1.85207095619268	2.88266056642802	8.22997647036184
H	2.22099820449809	2.20443221241469	5.90432890224360
H	3.82567995680931	5.69869070842879	4.46101278226574
H	5.64660727134481	7.04903400973507	3.52670267562897
H	6.20515787118472	6.89544903267879	1.12125034050799
H	4.92237026261018	5.36222477630104	-0.34065783865226
H	3.10926730098056	4.00269872629612	0.58748109821959

Compound 5a-II

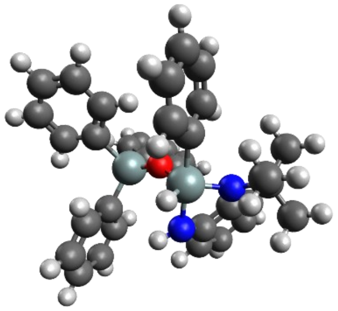

E = –2076.14665716 Eh

Si	-0.68577554891954	2.43421599657794	0.89897162805907
H	0.26451927490101	1.25810422442484	0.71322071947856
N	-1.19144589623068	3.67593403235211	-0.34185071266098
N	-2.05961302375111	1.41798733932874	0.11128706195017
C	0.74454523754651	3.46925626315936	1.77687782521465
C	0.54828454252226	4.74474871550650	2.31956501891082
C	1.51847232629821	5.38257058177057	3.08100781021566
C	2.73093327239588	4.74978261295552	3.33849816272090
C	2.95245567534249	3.48089996754895	2.82270245427976
C	1.96799098912032	2.86218214642001	2.05644376538515
C	-0.35132443640691	4.43222037181055	-1.29046388130286
C	-2.56230352379527	3.55893438238682	-0.57297299150675
C	-3.05092061775701	2.24929912582412	-0.30148618814779
C	-4.40662084347159	1.96954072630900	-0.46126349390035
C	-5.28646063221552	2.96762668370298	-0.88133303167076
C	-4.82075659833849	4.24754856474025	-1.12657933413442
C	-3.46107199333171	4.54092167023279	-0.96160187887711
H	-3.12237257053703	5.55351564920691	-1.13847821454956
H	-5.50093961343941	5.02988116970217	-1.43970365278594
H	-6.33812179369101	2.73704099720371	-1.00354947315199
H	-4.77237236741820	0.96919468574850	-0.25676775421325
H	-0.39455199822640	5.25513319934434	2.15166874630806
H	1.32940809653899	6.37082931718344	3.48453243935650
H	3.48962289321305	5.24051642575822	3.93583787705051
H	3.88962926361680	2.97271793425344	3.01911756004755
H	2.15884786685666	1.86608431233527	1.66496623420822
H	-2.37401504739570	0.54128058158472	0.50328859126880
C	1.07429856239619	3.86847149232186	-1.31279402701157
C	-0.26716513648299	5.92396787732086	-0.94222458035342
C	-0.87785408601126	4.27846894449869	-2.72672694310712
H	1.06975504384393	2.79500334151598	-1.51471216073050
H	1.64525109386173	4.35229876320983	-2.10752036181174
H	1.60628270121999	4.03691575801180	-0.38099520722576
H	0.36103487939463	6.45421466636684	-1.66177802256313
H	0.16641221644909	6.06593148346575	0.04718604247397
H	-1.24649771377250	6.40265324976120	-0.94985443058176
H	-1.85259430015908	4.73604325945736	-2.88323054219362
H	-0.18229988898370	4.75109395289178	-3.42240562766021
H	-0.95701111913038	3.22343563276847	-2.99685910176680
O	-1.55906474855884	2.57663884603132	2.38278298952949
Si	-2.51780656276545	1.92636859165858	3.51793368918140
C	-3.18268828201609	0.22776881974311	3.05212328141582
C	-2.31395537595885	-0.86610684890033	2.98278447050704
C	-2.76310364728840	-2.11945870317350	2.59233889066991
C	-4.10141439406478	-2.30517888542198	2.26675345674535
C	-4.98167775662928	-1.23477152710597	2.33599607431269
C	-4.52275524656607	0.01813656102112	2.72509716462126
C	-1.52354922893074	1.72486189324144	5.10317047883182
C	-1.93506000899850	0.85767140790099	6.11863038936779
C	-1.21086360046135	0.73101118399638	7.29675400945529
C	-0.04884243941991	1.46978656392346	7.47776196125031
C	0.38157072239084	2.33137992494947	6.47747837064767
C	-0.35050363003461	2.45733283547917	5.30379150868327
C	-3.97287365903412	3.07921536003724	3.82074756097861
C	-4.47998500355117	3.86313215328884	2.78059160794857
C	-5.57237687064237	4.69759998908800	2.97911514004271
C	-6.17964396501748	4.76586944352908	4.22588557372241
C	-5.68892414832405	3.99628359036995	5.27257477315510
C	-4.59630766237762	3.16397152718010	5.06762227865378
H	-1.26634653856077	-0.74127805299713	3.23532321661490
H	-2.07197294808342	-2.95138704642905	2.54164262751880
H	-4.45574456702894	-3.28185325972528	1.96215618872652
H	-6.02579263479263	-1.37405476715364	2.08521806798604
H	-5.22338367923090	0.84395252043237	2.76676416347523
H	-2.83202059855839	0.26117190818250	5.99006245440405
H	-1.54892819108760	0.05268680832222	8.07011849314295
H	0.52129283169049	1.37029157723004	8.39287324863606
H	1.29055170950333	2.90514007999346	6.60906199755664
H	0.00564678169751	3.12864117719881	4.53076644578551
H	-4.22401055975039	2.57844715014300	5.90104902948607
H	-6.15474846539411	4.04719671479431	6.24877118468385
H	-7.03043536583258	5.41710041451349	4.38236311342940
H	-5.94803151471845	5.29454199893693	2.15733326977073
H	-4.02765593765583	3.82179395075825	1.79629250404120

Compound 5a-III

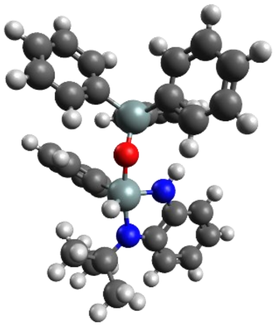

E = –2076.15265776 Eh

Si	-0.06932627492219	3.17443311287474	1.72750529783621
H	0.71632182401820	4.40298221866179	2.05992055218892
N	-0.06799905368592	3.61335798381299	-0.16171718382083
N	-1.81683511990934	3.06323950003358	1.47561782837690
C	0.93112826889721	1.55530917277347	1.54941063997989
C	1.97959868734588	1.22350412455388	2.41051437795275
C	2.65409351594863	0.01276521117976	2.31133154541933
C	2.27624566207523	-0.91546179124049	1.34910735404571
C	1.23408625872478	-0.61089185886234	0.48316465839865
C	0.58317228452838	0.61295852198793	0.58065253178121
C	1.10036767929892	4.01533909110500	-0.94977531463576
C	-1.29248534791026	3.46547317265429	-0.74987757752476
C	-2.30803501551398	3.14820676034064	0.20101954858829
C	-3.62468284088024	2.95879729259504	-0.17346033314180
C	-4.00241440980587	3.07486214911902	-1.51711254967890
C	-3.03929626838974	3.38008562944163	-2.45637859243780
C	-1.70193610205489	3.57225439603725	-2.08456199672515
H	-0.99860613823414	3.80416611146529	-2.86932127769738
H	-3.30658319384612	3.47335797066897	-3.50232257899531
H	-5.03499527118998	2.92484276882419	-1.80634143143614
H	-4.36215548714477	2.71766758555394	0.58507173062522
H	2.28166725347408	1.92128978989290	3.18265053783311
H	3.46821880353779	-0.21043324441653	2.99067819073611
H	2.79050599487821	-1.86568622049122	1.27384982404166
H	0.92856553569922	-1.32812594485949	-0.26931612166332
H	-0.21897530481347	0.83175260562180	-0.11520980077911
H	-2.47326952413931	2.82441126297071	2.19972203269613
C	2.34089463383026	4.18082165247485	-0.06058516573523
C	0.88561842320955	5.38882813571076	-1.61079573426311
C	1.47714381785873	2.95538026091891	-2.00067938503870
H	2.58605385948074	3.26941836174276	0.48389449185459
H	3.19642407331519	4.42258281281911	-0.69417744052388
H	2.22755593296469	4.98794297282604	0.66097382378987
H	1.78431791027282	5.69624133272976	-2.14995043088558
H	0.68406515119406	6.14044683743292	-0.84505855983680
H	0.05966745663948	5.41286565607849	-2.31785396380680
H	0.67150975735844	2.72633855027041	-2.69503240306227
H	2.33278056943906	3.29070395452865	-2.59124704491364
H	1.75678524232409	2.02397897126790	-1.50654900159566
O	-0.30301365718728	2.93096743611085	3.50983191692025
Si	-0.88685254203743	1.89605639851530	4.60668429834999
C	-1.77929132296708	0.39750984631964	3.88507382924124
C	-1.06515395573975	-0.67618673205650	3.34527992294383
C	-1.71640117837092	-1.76321982855368	2.77951244689999
C	-3.10439293453267	-1.79818179137251	2.73688289907185
C	-3.83428456969720	-0.74208982575942	3.26420389503445
C	-3.17416927103476	0.33998798496062	3.83347648732453
C	0.48259571365916	1.27020395206096	5.74119079349192
C	1.61083561012553	2.06069610739785	5.97556156473579
C	2.61690460407811	1.64595939693659	6.83725672001078
C	2.51256616191199	0.42367002194274	7.48945317204464
C	1.39903272648484	-0.37630634489824	7.27405229942960
C	0.39778324094920	0.04641547340785	6.40801072655845
C	-2.14225091718689	2.81223272842964	5.68017312115925
C	-2.71343577551313	4.00916653763286	5.24389170962613
C	-3.66030268306147	4.67823401422476	6.01046790641651
C	-4.05292689058245	4.16019914018083	7.23671536949038
C	-3.49509784203145	2.97138123017178	7.69094186935231
C	-2.55150643768577	2.30929499585359	6.91772571089359
H	0.01876446924590	-0.66522259020013	3.35600101535003
H	-1.13971594530409	-2.58092938173330	2.36553106921715
H	-3.61419231981571	-2.64443987001520	2.29370493155678
H	-4.91653227549315	-0.76100733653461	3.23399987568836
H	-3.76332384432632	1.15174288052591	4.24528929705300
H	1.71282293081569	3.01424047538087	5.47019097605322
H	3.48373615175952	2.27444042906715	6.99931527071280
H	3.29670389563412	0.09615581880662	8.16037673393707
H	1.31177392553538	-1.33152576877841	7.77658871895656
H	-0.45902327664617	-0.59834041242493	6.24689459879271
H	-2.12572232257906	1.38480030643756	7.29340485341370
H	-3.79483801450597	2.56261365463345	8.64790475989977
H	-4.78861422918828	4.68020592933827	7.83729257721465
H	-4.09013552164774	5.60486929055021	5.65076873620313
H	-2.41808285121314	4.42953000600502	4.28950899386799

Compound 5a-IV

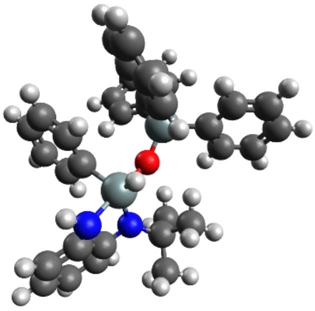

E = –2076.13450038 Eh

Si	-0.63753585694407	3.59494362853055	1.60522348321029
H	0.55156074746432	2.71912420883286	1.33890903650310
N	-1.21640802380017	4.95946880119206	0.56516713109135
N	-1.67658136329704	2.54981351996920	0.39830969649242
C	-1.76436336627597	3.08672996605364	3.06149871508841
C	-2.93072881248104	3.79195234513195	3.37885963899670
C	-3.75016226541176	3.41090811056564	4.43532412046443
C	-3.41884376316189	2.31056009326279	5.21560454445635
C	-2.26783828993678	1.59234916872309	4.91828506062956
C	-1.46165680882676	1.97521508065117	3.85270728588684
C	-0.45224829216355	6.11835742650529	0.06910072020331
C	-2.49627247007680	4.68019466725903	0.08842876256400
C	-2.74605488821411	3.28102438033083	-0.00663714403059
C	-3.99036648374014	2.83304029539529	-0.44577864548248
C	-4.99146777175287	3.74570949903507	-0.78406507392665
C	-4.76510917844729	5.10416545852912	-0.65599121479292
C	-3.52338611679355	5.56576900299290	-0.19946990940487
H	-3.38345975178481	6.62971599787161	-0.05905815859506
H	-5.54621006216215	5.81699414977411	-0.88911615914818
H	-5.95176262440645	3.38215897560950	-1.13017461102078
H	-4.17598449827391	1.76718491132246	-0.52481312172463
H	-3.20721923671505	4.66589512773468	2.79957955515252
H	-4.64553022491046	3.98042081395685	4.65509432582559
H	-4.04985645035601	2.01646832660531	6.04507904094042
H	-1.99482085227486	0.73297907894564	5.51939518696244
H	-0.56741784863219	1.39586360777284	3.64777039600263
H	-1.87367117780048	1.57342193309655	0.56837702856309
C	1.03416850277552	5.75205992157185	-0.04458761713901
C	-0.62316223600836	7.33732687254063	0.98177684002147
C	-0.86495800780555	6.50217748636475	-1.36216607102934
H	1.16482762317038	4.91272615969650	-0.73137948151484
H	1.59576521855479	6.59854119199310	-0.44503942811776
H	1.47737645574868	5.48706393891811	0.90855254600874
H	-0.03483904668434	8.18575559564236	0.62391514383082
H	-0.30498496260429	7.10506929200670	1.99668500382018
H	-1.66693521792857	7.65417724113851	1.02302361442202
H	-1.85702220926348	6.93953219210081	-1.43867318607688
H	-0.15999759196252	7.24248995441527	-1.74389717119774
H	-0.82903883378853	5.63621216196514	-2.02574670715988
O	0.31838776013268	4.67289266930658	2.69551329709648
Si	1.40237715894236	4.57867137166903	3.89595583223734
C	1.92534464045119	2.81454497154530	4.31750385431316
C	1.67845110163520	2.25596777451496	5.57229745831015
C	2.03083760225044	0.94353341965988	5.86287972649735
C	2.64418076241250	0.15988405077428	4.89577571950907
C	2.90709085002252	0.69694066148716	3.64151563364059
C	2.55105725563052	2.00864271111396	3.36118611098940
C	0.72500186057056	5.36254800822313	5.47239993388424
C	-0.64113184546921	5.34656614176367	5.76141343330391
C	-1.13363398666886	5.88312437398339	6.94427055260643
C	-0.26422919855037	6.45022107854044	7.86635630362135
C	1.09829486376580	6.47722698955900	7.59843063316104
C	1.58234853263299	5.93894375342711	6.41323441660876
C	2.96908754549627	5.54593197187126	3.46444242970117
C	4.25309743712768	5.03181648662350	3.65173227348672
C	5.38525682729092	5.78444815847113	3.35933841750654
C	5.25272914479723	7.07653174695676	2.87244712528948
C	3.98424338278382	7.61192080424364	2.68304578228510
C	2.86105410162201	6.85344958489400	2.97896169474057
H	1.19306150992450	2.84804057829612	6.33923459056178
H	1.82383735171010	0.53251111372032	6.84310482302980
H	2.91779315192034	-0.86408391872846	5.11718660811099
H	3.38756694032744	0.09248230708706	2.88234913325278
H	2.76061306324402	2.40561336327255	2.37446496024186
H	-1.33590842032830	4.90701474904407	5.05645535588042
H	-2.19765863578000	5.85852415087104	7.14494208939349
H	-0.64590232307802	6.87065323377337	8.78834440594744
H	1.78277306653743	6.91998313304226	8.31130118015498
H	2.64918031733630	5.97527916899570	6.22102785258359
H	1.88189083333084	7.29249306392050	2.82416015615835
H	3.87192312817888	8.62061538829605	2.30551726999670
H	6.13185481151752	7.66510372586402	2.64218955498902
H	6.36994526162797	5.36043785814416	3.51183600726061
H	4.38124018362684	4.02531477176677	4.03232220687270

Compound 5b-I

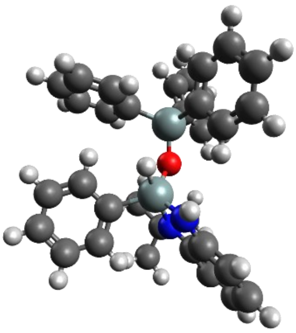

E = –2076.14262062 Eh

Si	-0.80924676369756	3.90869113632060	2.23158272231876
H	-0.55455555908189	2.97679555792428	3.47641610520976
N	-1.11553745977687	4.83630857093816	0.55182999057398
N	-1.29893202276511	2.49132330406871	1.30283115381024
C	-2.17193648998217	4.83982663600776	3.20578372114028
C	-3.42046377379199	5.10010546495992	2.63674817005256
C	-4.45386211643263	5.67393304536310	3.36890423409870
C	-4.25303739101780	6.02591389297292	4.69712628224635
C	-3.02206167708858	5.77094664711189	5.28896286705898
C	-2.01034254903433	5.16553658791129	4.55442156918022
C	-0.92302929948300	6.26847268859049	0.27500425983620
C	-1.48042899419205	3.98703857269215	-0.45848605877474
C	-1.60331730249072	2.63295283669110	-0.02276379637061
C	-1.98174331736634	1.61803579109374	-0.88135235498820
C	-2.25200157830659	1.89079967136811	-2.22694079658430
C	-2.12799196397536	3.18860882799398	-2.67852536594047
C	-1.74444975439683	4.22207609517957	-1.81472479853623
H	-1.66312331651140	5.20996067327239	-2.23955466303283
H	-2.32702141424486	3.42740866058172	-3.71653826803273
H	-2.54977461326357	1.09304958431797	-2.89570818353057
H	-2.06416316498166	0.60566689446549	-0.49992580349701
H	-3.59117710183577	4.85334050479211	1.59559550278363
H	-5.41505776195074	5.85120630987690	2.90109130821940
H	-5.05016497884487	6.48604601586966	5.26798279236912
H	-2.85421046530135	6.03216479380627	6.32716768841053
H	-1.07161671685930	4.94199453078917	5.04801922644925
H	-1.32197552243412	1.56692406766289	1.70008816058550
C	-0.53719532120666	7.06235606590208	1.53225829819563
C	-2.22210913715291	6.93236409832710	-0.22338751394286
C	0.22488029158012	6.49592776856082	-0.72549975038157
H	0.41645669541581	6.74875481904166	1.94847164528032
H	-0.44717503083709	8.11562955610366	1.25865418113766
H	-1.29386029644732	6.99412579960866	2.31132231234660
H	-2.03776045555510	7.97920977747993	-0.47413153771109
H	-2.97603301710274	6.91050583679831	0.56444250866252
H	-2.65609409936685	6.46036104577049	-1.10114009426978
H	0.07195980017650	6.01397709260773	-1.68841122836600
H	0.36334452433494	7.56298136466717	-0.91274701933689
H	1.15716114281137	6.10952292577477	-0.31230163098905
O	0.82242499783410	4.46223981625469	2.27520420570096
Si	2.02754076471560	4.17943711797787	3.34978930571042
C	2.59085526406362	2.38422628687391	3.34912204508712
C	3.60889519529159	1.97237810425268	4.21393628092034
C	4.08474697412058	0.66870895155265	4.19946328949350
C	3.55046428619170	-0.25348595733053	3.30844912661675
C	2.54200870581244	0.13686009362813	2.43833131306331
C	2.06849669548225	1.44288667651449	2.46133105710025
C	1.59604786319998	4.70436393944592	5.10264258103228
C	1.50836067732738	6.06413411005196	5.41299307143245
C	1.09093917011551	6.49189363380033	6.66560854133472
C	0.75463352158630	5.55968166862809	7.63879629533160
C	0.84234874062315	4.20365281845650	7.35352723344897
C	1.25704793013960	3.78451291717411	6.09614876609119
C	3.49745113547767	5.22046267316005	2.79598961476714
C	3.66943281895618	5.55447793278725	1.45076499683869
C	4.77655076821034	6.27430725668037	1.02151483892827
C	5.74261594090765	6.67313085929645	1.93566997583280
C	5.59433124384809	6.34756100467113	3.27728730741481
C	4.48249019129983	5.62958787211273	3.69819780425085
H	4.04325450202889	2.67690658881922	4.91571617257327
H	4.87156367950427	0.37194940414271	4.88154904230955
H	3.91942983278185	-1.27124907960147	3.29295464065336
H	2.12110626505755	-0.57672342612751	1.74108395826703
H	1.27516413165439	1.72438986294554	1.77955138583280
H	1.76143460185372	6.80771305682028	4.66503276910679
H	1.02446749273198	7.55062519811340	6.88215583383093
H	0.42667242461730	5.88922279931152	8.61658905453115
H	0.58097691122977	3.47287218757740	8.10846156861876
H	1.30585643973867	2.72232023555800	5.88701723542786
H	4.38289440656396	5.39332048277011	4.75190610233704
H	6.34258722539824	6.65646149085305	3.99641834517930
H	6.60617227292162	7.23593450213885	1.60444538860577
H	4.88461865673793	6.52477369034787	-0.02637627715810
H	2.92783624443355	5.25166171707669	0.72160129380640

Compound 5b-II

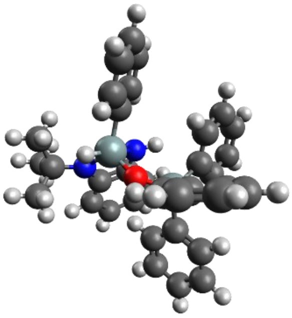

E = –2076.14585473 Eh

Si	-0.59748397188512	2.58268738644711	1.23686116498867
H	0.43278811168517	3.58396646387565	1.78646383346186
N	-1.00942641469494	3.69143843635800	-0.16728442025880
N	-1.90124404714004	1.51146128181420	0.44375564805094
C	0.68708478913405	1.14726171425327	1.32680761346020
C	1.73698000541421	1.26302624223843	2.24122893221202
C	2.67714970296591	0.25557730009128	2.41887247987025
C	2.60325383400719	-0.90078236411221	1.65260148588898
C	1.58233181480808	-1.03565123159384	0.72045848494759
C	0.63775083297928	-0.02663088445821	0.57004154765366
C	-0.13699416079212	4.72601523382499	-0.74001983205885
C	-2.33104038682410	3.54464844293831	-0.55359665883835
C	-2.83597067334859	2.25902381847619	-0.19839684727759
C	-4.14907028653375	1.91310473030785	-0.49478208041925
C	-4.99807572768009	2.83295121835593	-1.11453942545787
C	-4.53494529454190	4.10031748017233	-1.41395756182616
C	-3.21107419890593	4.45832234481766	-1.12311664350015
H	-2.89926913505016	5.47038935812516	-1.33869665061122
H	-5.19110374469945	4.83159767646338	-1.86975074678115
H	-6.01988724486677	2.55377511958800	-1.34158795315301
H	-4.51167103526972	0.92639481933161	-0.22693242535309
H	1.81898212781490	2.17234386913935	2.82919638284820
H	3.47019613135481	0.37308316371966	3.14774367747018
H	3.33735502170871	-1.68728928219247	1.77744936820290
H	1.52069855012661	-1.93028331112036	0.11218016147694
H	-0.15850134346159	-0.15536685726873	-0.15282528914811
H	-2.24676600043442	0.66311277580186	0.86705776341325
C	1.33736344600293	4.31487259442924	-0.59695993888579
C	-0.33745973870327	6.07839768127653	-0.04590914025015
C	-0.35394823566537	4.86368233549818	-2.25657119469679
H	1.50603716366861	3.32704091028865	-1.03093087872709
H	1.96727832677151	5.02702035886831	-1.13321279572623
H	1.66785484152573	4.28913724704925	0.43699019165440
H	0.32328956300509	6.84072682479882	-0.46507765101194
H	-0.11717171154400	5.98847852106966	1.01879992603088
H	-1.36189663047414	6.43928169274638	-0.14423545636862
H	-1.32344895620334	5.26360121451835	-2.53920424731012
H	0.40097192053966	5.53542594999827	-2.66850228313055
H	-0.23874571903283	3.89472399672684	-2.74609055754020
O	-1.48315360397216	2.76760959149503	2.71993186128105
Si	-2.55708808033772	2.08479143955957	3.73113855393037
C	-2.98537389817075	0.30370342195271	3.29704890440970
C	-2.00120916935453	-0.68651297600228	3.38021315570301
C	-2.26471255615022	-1.99628264418494	3.00633347545156
C	-3.52908486068292	-2.34480508331640	2.54667921093038
C	-4.52239892163472	-1.37917964407790	2.46407111623689
C	-4.24891926866347	-0.06812342285740	2.83568458693538
C	-1.80343126957385	2.06678511517919	5.45621805329671
C	-2.27272265169527	1.20374457863415	6.44966047536083
C	-1.73177385545805	1.21309295698686	7.72864619265902
C	-0.69853941568576	2.08806479760705	8.03707818280163
C	-0.21175946547662	2.94860575211708	7.06196147453435
C	-0.76101319092897	2.93601723186009	5.78640639141068
C	-4.14366422075578	3.09574752550681	3.74908460582957
C	-4.49108119972895	3.89359086195982	2.65664632946274
C	-5.67679677276793	4.61735811831209	2.64448987842785
C	-6.53886415601001	4.55895721692846	3.73087137910512
C	-6.20955631151710	3.77425599215398	4.82887858335243
C	-5.02349588805546	3.05277451809645	4.83352040501379
H	-1.00739762150602	-0.43409445027542	3.73380630361751
H	-1.48512315374018	-2.74480232817602	3.07134481252315
H	-3.73805177352258	-3.36603205841443	2.25402823354804
H	-5.50942545279874	-1.64526525251780	2.10710239528132
H	-5.03350477316571	0.67528310447854	2.75478966866350
H	-3.06869910521862	0.50206035563236	6.22434678671231
H	-2.11150673567466	0.53452166870103	8.48216543192605
H	-0.27113934416845	2.09567571481907	9.03182284823136
H	0.59840296727954	3.62830632637713	7.29476973027567
H	-0.36362044031469	3.60915818950547	5.03596666274558
H	-4.78348254569240	2.45184409731225	5.70395722246084
H	-6.87550046433382	3.72657206971679	5.68138696487028
H	-7.46295255349031	5.12323982934764	3.72374816711732
H	-5.92543257342132	5.22611035583682	1.78412955286581
H	-3.83768919847201	3.94883275708253	1.79394441973366

Ph_3SiOH

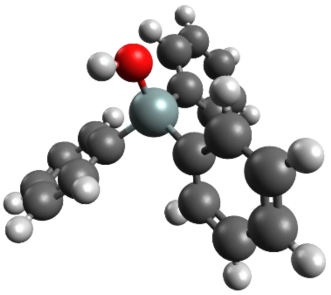

E = –1059.67113261 Eh

Si	0.47292799053135	3.72588256692999	-0.44550617650774
O	0.55906862334577	4.45625614430503	1.05576283001184
H	-0.24171821276121	4.39895987989528	1.58354873566089
C	0.85140511250483	1.90096600274106	-0.31058506689430
C	1.67937063514133	1.42674413273381	0.70898857908163
C	2.01903432028362	0.08470992323578	0.78688663794377
C	1.53959360062639	-0.80893428858508	-0.16053982483447
C	0.71842058255411	-0.35597246055658	-1.18290402440961
C	0.37778320025165	0.98671539579076	-1.25396468084501
C	1.75258155201898	4.59488566012306	-1.48492634053840
C	2.35038421510927	3.94568701536096	-2.56611866208664
C	3.25143501900800	4.60669328646534	-3.38678686831211
C	3.57315964286687	5.93298203519214	-3.13603362623752
C	2.99140631424163	6.59307108356911	-2.06321004125985
C	2.08889452387804	5.92873644029383	-1.24597092464883
C	-1.23595708181621	3.94295084239663	-1.17908532570256
C	-1.50238624767423	4.94354555085483	-2.11579061233369
C	-2.78193192336944	5.13196899546592	-2.61683731391538
C	-3.82241703691533	4.32135235086892	-2.18682903952493
C	-3.57860932639806	3.32099456640394	-1.25633471946919
C	-2.29714068906708	3.13494832081003	-0.76081983721293
H	3.70820153889643	4.08587641059015	-4.21821644766417
H	2.12023138196073	2.90576917503953	-2.76333457628346
H	4.27779010652145	6.45039878169828	-3.77429832678248
H	3.24498635666624	7.62557010020491	-1.86060184550889
H	1.64805954299705	6.44783281203328	-0.40480647546212
H	-0.69712557383882	5.57781441564937	-2.46428279393860
H	-2.11802366221159	2.33623502089029	-0.05035937450031
H	-4.38639048644160	2.68299006512688	-0.92192515837140
H	-4.82101484635516	4.46605665757962	-2.57839836181864
H	-2.96738843357818	5.90932835915589	-3.34658845513190
H	-0.27530695039724	1.32525282250692	-2.04901139386661
H	2.05698055717630	2.11635655811537	1.45364890839960
H	2.65865263152494	-0.26559847174092	1.58662252097845
H	1.80501606857484	-1.85657521288570	-0.10156720536408
H	0.33977695414433	-1.05031093825877	-1.92174471265033
